# Systems-Level Proteomics Evaluation of Microglia Response to Tumor-Supportive Anti-Inflammatory Cytokines

**DOI:** 10.3389/fimmu.2021.646043

**Published:** 2021-09-09

**Authors:** Shreya Ahuja, Iulia M. Lazar

**Affiliations:** ^1^Department of Biological Sciences, Virginia Tech, Blacksburg, VA, United States; ^2^Fralin Life Sciences Institute, Virginia Tech, Blacksburg, VA, United States; ^3^Carilion School of Medicine, Virginia Tech, Blacksburg, VA, United States

**Keywords:** microglia, cytokine, anti-inflammatory, ECM remodeling, tumor, proteomics

## Abstract

**Background:**

Microglia safeguard the CNS against injuries and pathogens, and in the presence of certain harmful stimuli are capable of inducing a disease-dependent inflammatory response. When exposed to anti-inflammatory cytokines, however, these cells possess the ability to switch from an inflammatory to an immunosuppressive phenotype. Cancer cells exploit this property to evade the immune system, and elicit an anti-inflammatory microenvironment that facilitates tumor attachment and growth.

**Objective:**

The tumor-supportive biological processes that are activated in microglia cells in response to anti-inflammatory cytokines released from cancer cells were explored with mass spectrometry and proteomic technologies.

**Methods:**

Serum-depleted and non-depleted human microglia cells (HMC3) were treated with a cocktail of IL-4, IL-13, IL-10, TGFβ, and CCL2. The cellular protein extracts were analyzed by LC-MS/MS. Using functional annotation clustering tools, statistically significant proteins that displayed a change in abundance between cytokine-treated and non-treated cells were mapped to their biological networks and pathways.

**Results:**

The proteomic analysis of HMC3 cells enabled the identification of ~10,000 proteins. Stimulation with anti-inflammatory cytokines resulted in the activation of distinct, yet integrated clusters of proteins that trigger downstream a number of tumor-promoting biological processes. The observed changes could be classified into four major categories, i.e., mitochondrial gene expression, ECM remodeling, immune response, and impaired cell cycle progression. Intracellular immune activation was mediated mainly by the transducers of MAPK, STAT, TGFβ, NFKB, and integrin signaling pathways. Abundant collagen formation along with the expression of additional receptors, matrix components, growth factors, proteases and protease inhibitors, was indicative of ECM remodeling processes supportive of cell-cell and cell-matrix adhesion. Overexpression of integrins and their modulators was reflective of signaling processes that link ECM reorganization with cytoskeletal re-arrangements supportive of cell migration. Antigen processing/presentation was represented by HLA class I histocompatibility antigens, and correlated with upregulated proteasomal subunits, vesicular/viral transport, and secretory processes. Immunosuppressive and proangiogenic chemokines, as well as anti-angiogenic factors, were detectable in low abundance. Pronounced pro-inflammatory, chemotactic or phagocytic trends were not observed, however, the expression of certain receptors, signaling and ECM proteins indicated the presence of such capabilities.

**Conclusions:**

Comprehensive proteomic profiling of HMC3 cells stimulated with anti-inflammatory cytokines revealed a spectrum of microglia phenotypes supportive of cancer development in the brain *via* microenvironment-dependent biological mechanisms.

## Introduction

Microglia cells represent the macrophage network population of the central nervous system (CNS). Microglia are found in the brain in a ramified form, i.e., having a small cell body with long branched protrusions which the cells extend and retract to inspect the environment for toxic and necrotic molecules, or for pathogens. The cells act as the surveillance system of the CNS, constantly patrolling the region and responding by activating a repertoire of cytotoxic factors that destroy the invaders (1, 2). Once a damage is detected, they rapidly transform into “reactive” ameboid mobile effector cells that can proliferate, phagocytose and interact with other immune cells *via* antigen presentation. Therefore, microglia play a crucial role in maintaining and restoring the homeostasis of the CNS. While serving as immune guardians, however, microglia have the ability to engage in distinct response programs which may be cytotoxic or immunosuppressive (3). Their routine function is disrupted in the presence of cancer. The “classical” M1 phenotype recognizes and attacks injuries, infections and damages to the CNS. The “alternative” M2 phenotype, on the other hand, dampens inflammatory responses and supports tumor progression (4). The polarization of microglia toward either phenotype is triggered by a specific concoction of cytokines. Stimulation toward the M1 phenotype occurs in response to bacterial endotoxin lipopolysaccharides (LPS) or interferons (IFN-γ), which render the microglia bactericidal and immunostimulant. In this subtype, there is an increased expression of STAT1 in the cells that further upregulates the expression of pro-inflammatory cytokines (IL-1β, IL-12, TNF-α) and of oxidative metabolites (iNOS) (5). On the contrary, the M2 phenotype is elicited in response to anti-inflammatory cytokines released from cancer cells that promote the development of tumor-supportive/pro-neoplastic microglia (6). In this case, the tumor cells in the brain “hijack” the pro-inflammatory nature of the microglia by sending out signals that promote tumor progression through a variety of processes, including matrix deposition and tissue remodeling (7–12). Chemoattractants such as CCL2 (or MCP1), secreted by cancer cells, can stimulate microglia *via* CCR2 activation, and recruit the microglia to the tumor microenvironment to support invasiveness through IL-6 expression (8). IL-6 activates an “M2” specific enzyme, Arginase-1 (ARG1) (9), that can alter the extracellular matrix composition to drive cancer progression. Additionally, anti-inflammatory cytokines such as IL-10, IL-13, IL-4 and TGFβ skew the microglia toward an M2 phenotype by increasing the intracellular levels of STAT3, which then negatively affects the expression of MHC-II, CD80, and CD86 (5, 10–12), and upregulates the expression of the macrophage scavenger receptor 1 (MSR1)/CD204 and of CD163 (5).

The brain offers a privileged, immunocompetent niche which is safeguarded from insults to the rest of the body. The blood brain barrier isolates the brain from the outside environment (13), thereby offering a sheltered destination site for circulating cancer cells (14, 15). Numerous reports have suggested that resident brain cells such as microglia support the survival and growth of metastasized cancer due to their ability to respond favorably to signals from the tumor microenvironment (16). Despite the prominence of these immune sentinels in the CNS and their role in the establishment of brain metastases, the interactions of microglia with the tumor cells and the acquisition of distinct anti-inflammatory properties is yet to be fully explored. The field is primarily limited by the inability to differentiate the brain microglia from the infiltrated bone-marrow derived macrophages (17). Many groups have reported similarities between the M2 activation of microglia and of tumor-associated macrophages (TAMs) that contribute to an immunosuppressive and tumor supporting milieu, however, the exact mechanisms of microglial plasticity, especially their re-programming during cancer metastasis, is still ambiguous to the scientific community (18). Moreover, due to ethical difficulties associated with the procurement of brain samples or isolation of primary cell cultures, studies focused on clarifying the role of microglia in the establishment of metastasized cancer have been mostly performed using rodent models (19, 20). The recent introduction of human microglia cell lines has been pivotal in overcoming these issues. In contrast to primary cells which have low renewing efficiency, bacterial plasmid SV40 large T-antigen transfected cells offer the ease of *in-vitro* culture and yield adequate amounts for sample analysis (21, 22). Easy upscaling is also supportive of large-scale omics projects, as demonstrated in a study in which IFN-γ and IL-4 were used to induce M1 or M2 polarization (23). To address the paucity of information in this field and advance the understanding of metastatic cancer cell development in the brain, in this study, we used mass spectrometry (MS) and proteomics to extensively characterize the human fetal brain-derived microglial cell line (HMC3) under basal and anti-inflammatory cytokine stimulation conditions. The HMC3 cells were stimulated with a cytokine cocktail known to originate from cancer cells, that was comprised of interleukins IL-4, IL-13, IL-10, growth factors TGFβ1/TGFβ2, and chemokine CCL2. These factors have been extensively studied in various animal and macrophage model systems, but much less in human microglia, and found to be key players in promoting tumorigenesis in the brain (5, 24–26). TGFβ is one of the most important anti-inflammatory cytokines that augments the action of IL-4 in promoting an M2 pro-tumor phenotype in a MAPK dependent signaling manner (27). IL-4 works in concert with IL-13 and IL-10 to execute anti-inflammatory roles that limit the production of inflammatory chemokines by downregulating the activity of STAT1 and NFKβ (28). Cell activation by IL-4 and IL-13 has been also shown to downregulate the expression of certain ECM remodeling matrix metalloproteinases (MMPs) (29), and to upregulate Arginase-1 (30) to lay the groundwork for collagen formation, tissue repair and cell growth. MCP1/CCL2, a chemokine, helps in recruiting monocytes to the site of tumorigenesis (31, 32). The proteomic data generated from stimulated and non-stimulated cells were explored for the presence of microglia-specific markers, cell surface proteins with immunological functions, and for quantitative changes in protein expression between cytokine-treated and non-treated cells. The results were assessed and discussed in terms of altered expression of specific protein sets and biological processes that are crucial for the suppression of immune activation and the formation of a tumor-supportive niche.

## Experimental Methods

### Materials

HMC3 cells, Eagle’s minimum essential medium and penicillin-streptomycin (PenStrep) solution were purchased from ATCC (Manassas, VA). Phenol red and glutamine free Minimum Essential Medium (MEM), L-glutamine, Dulbecco’s phosphate buffered saline (DPBS) and trypsin-EDTA were purchased from Gibco (Gaithersburg, MD). Fetal bovine serum (FBS) was supplied by Gemini Bio Products (West Sacramento, CA). Recombinant human IL-4, IL-10, IL-13, CCL2, TGFβ1 (HEK293 derived) and TGFβ2 (HEK293 derived) were acquired from Peprotech (Rocky Hill, NJ). Primary antibody [mouse monoclonal IgG anti-Vimentin (V9)], secondary antibody mouse IgG conjugated to CruzFluor488 (sc-516176), UltraCruz® blocking reagent, and UltraCruz® aqueous mounting medium with DAPI were purchased from Santa Cruz Biotechnology (Dallas, TX). The Proteome Profiler Human XL Cytokine array kit was bought from R&D Systems (Minneapolis, MN). Propidium iodide for FACS analysis was from Invitrogen (Carlsbad, CA). Cell Lytic™ NuCLEAR™ extraction kit for the separation of nuclear and cytoplasmic fractions of cells, trifluoroacetic acid (TFA), protein standards, phosphatase inhibitors [sodium orthovanadate (Na_3_VO_4_) and sodium fluoride (NaF)], ammonium bicarbonate (NH_4_HCO_3_), urea, dithiothreitol (DTT), ribonuclease (RNase) and Triton X-100 were procured from Sigma Aldrich (St. Louis, MO). Bradford reagent and bovine serum albumin (BSA) standards were provided by Biorad (Hercules, CA). Trypsin, sequencing grade, was from Promega (Madison, WI). Sample clean-up SPEC-PTC18 and SPEC-PTSCX pipette tips were bought from Agilent technologies (Santa Clara, CA). HPLC grade acetonitrile and methanol were from Fischer Scientific (Fair Lawn, NJ). Ethanol was obtained from Decon Laboratories (King of Prussia, PA). High purity water was prepared in-house by distillation of de-ionized water.

### Cell Culture

HMC3 cells were authenticated by short-tandem repeat (STR) profiling at ATCC. Three batches of cells, revived from liquid nitrogen, were used to produce three biological replicates of each control and stimulated cells. HMC3 cells were routinely cultured in EMEM supplemented with FBS (10%), in a water jacketed incubator at 37 °C and with 5% CO_2_. PenStrep (0.5%) was added to all culture media. For generating control cultures, the cells were either starved for 48 h in EMEM, or starved for 48 h and then released with EMEM supplemented with FBS (10%) for 24 h. For cytokine stimulation, the HMC3 cells were starved for 24 h in phenol red-free MEM with glutamine (2 mM), and then for another 24 h with MEM supplemented with a cytokine cocktail. Alternatively, cells starved for 48 h in EMEM were stimulated with EMEM supplemented with FBS (10%) and cytokines. The cytokines were added in concentrations that were proven effective in previously published works (23, 33, 34). The cytokine cocktail included TGFβ1 (20 ng/mL), TGFβ2 (20 ng/mL), IL-13 (20 ng/mL), IL-10 (20 ng/mL), IL-4 (40 ng/mL), and CCL2 (40 ng/mL).

### FACS Analysis

The cells were fixed using 70% ethanol and then stained with propidium iodide (0.02 mg/mL) in a PBS solution containing Triton X-100 (0.1%) and RNAase (0.2 mg/mL). The cells were incubated for 30 min at room temperature in the staining solution before being subjected to flow cytometry analysis (FACSCalibur, BD Biosciences, San Jose, CA).

### Immunofluorescence Microscopy

Immunofluorescent detection of vimentin was performed by fixing the cells with chilled methanol (-20°C, 15 min), blocking (1 h), incubating with primary antibody (2 ug/mL in blocking buffer, 1 h), and staining with the secondary antibody conjugated to the detection fluorophore (1 ug/mL in blocking buffer, 30 min). The cells were visualized with an inverted Eclipse TE2000-U epi-fluorescence microscope (Nikon Instruments Inc., Melville, NY). Images were acquired and processed with a Nikon’s NIS-Elements Advanced Research imaging platform, version 5.11.01.

### Cytokine Array

Quantitative assessment of 105 cytokines was performed using the Proteome Profiler Human Cytokine Array kit, in triplicate, in accordance with the manufacturer’s protocol. Conditioned medium from cells cultured under serum-free conditions was collected by centrifuging the medium at 2,000 rpm for 5 min, and used for incubating the cytokine membranes overnight, at 4°C. The membranes were treated the next day with the antibody cocktail for 1 h followed by the streptavidin-HRP conjugate for 30 min. The chemiluminescence signal was detected using a ChemiDoc™ Imaging System (BioRad, Hercules, CA) after an optimized exposure time of 10 min. The pixel intensity of each spot was acquired using Image Studio Lite 5.x (https://www.licor.com) and processed further with Microsoft Excel.

### Sample Preparation for MS Analysis

The cells were harvested by trypsinization at the end of each treatment and flash frozen at -80°C until further use. Cell fractionation into nuclear (N) and cytoplasmic (C) lysates was performed to allow for the generation and characterization of an enriched pool of nuclear proteins, and was carried out in accordance with the protocol described in the Cell Lytic™ NuCLEAR™ extraction kit. The lysis reagents were supplemented with phosphatase and protease inhibitors. A Bradford assay was performed to measure the concentration of the protein extracts. Protein extracts (500 ug) were denatured and reduced with 8 M urea and 5 mM DTT for 1 h at 57°C. The reduced proteins were digested overnight with trypsin at 50:1 protein-to-enzyme ratio at 37 °C. The reaction was quenched with glacial acetic acid, and the samples were subjected to C18/SCX clean-up. The protein digests were re-suspended in H_2_O:CH_3_CN : TFA (98:2:0.01) at a concentration of 2 μg/μL (35, 36), and frozen at -80°C until liquid chromatography (LC)-MS analysis was conducted.

### LC-MS Analysis

LC separations were performed with an EASY-nLC 1200 UHPLC system by using an EASY-Spray column ES802A (250 mm long, 75 μm i.d., 2 μm C18/silica particles, ThermoFisher Scientific) operated at 250 nL/min. Mobile phase A consisted of 0.01% TFA in H_2_O:CH_3_CN (96:4, v/v), and mobile phase B of 0.01% TFA in H_2_O:CH_3_CN (10:90, v/v). The solvent gradient was ~2 h long, with increasing concentration of solvent B according to the following steps: 7% B, 2 min; 7-30% B, 105 min; 30-45% B, 2 min; 45-60% B, 1 min; 60-90% B, 1 min; 90% B, 10 min; 90-5% B, 1 min; 7% B, 5 min. Mass spectrometry analysis was performed with a Q Exactive hybrid quadrupole-Orbitrap mass spectrometer (ThermoFisher Scientific). Nano-electrospray ionization (ESI) was induced at 2 kV, and the ion transfer capillary temperature was set to 250°C. Full mass spectra were acquired over a scan range of 400-1600 m/z, with acquisition parameters set to resolution 70,000, AGC target 3E6, and maximum IT 100 ms. The quadrupole isolation width was 2.4 m/z, and HCD (higher energy collisional dissociation) was performed at a normalized collision energy of 30%. Data dependent MS2 acquisition (DDA) was completed with a resolution setting of 17,500, AGC target 1E5, maximum IT 50 ms, and loop count of 20. Other data dependent settings included minimum AGC target 2E3 (with resulting intensity threshold of 4E4), apex trigger 1 to 2 s, charge exclusion enabled for unassigned and +1 charges, isotope exclusion on, preferred peptide match on, and dynamic exclusion of 10 s for chromatographic peak widths of 8 s. Validation of changes in the detection or expression of selected proteins was performed with targeted parallel reaction monitoring (PRM) at quadrupole isolation of 2 m/z, resolution of 35,000, AGC target (1-2)E5, and maximum IT 110 ms. LC separations for PRM were performed by using identical conditions as used for DDA-MS analysis, or, in the case of a few poorly defined chromatographic peak shapes, by using a fast, 20 min long, 0-90% solvent B gradient.

### MS Data Processing

Peptide-spectrum matches (PSM) and protein identifications were performed with the Sequest HT search engine embedded in the Proteome Discoverer (v. 2.4) software package (Thermo Fisher Scientific, Waltham, MA), and a target/decoy-based processing workflow of peptide precursor masses between 400 and 5,000 Da. The Sequest HT node searches were made against a *Homo sapiens* database, downloaded from UniProtKB (March 2019), containing 20,404 reviewed/non-redundant sequences. The workflow parameters were set up for the identification of only fully tryptic peptides with a minimum of 6 and maximum of 144 amino acids, maximum 2 missed cleavages, precursor ion tolerance of 15 ppm, and fragment ion tolerance of 0.02 Da. All **b/y/a** ion fragments were considered, with dynamic modifications on Met (oxidation) and the N-terminal amino acid (acetyl). The signal-to-noise (S/N) ratio threshold was set to 1.5. The target/decoy PSM validator node included concatenated databases, with input data filtered for maximum DeltaCn of 0.05 and maximum rank of 1, and strict/relaxed target PSM FDRs of 0.01 and 0.03, respectively. In the consensus workflow, the peptide group modification site probability threshold was set to 75, and the peptide validator node to automatic mode with strict/relaxed PSM and peptide target FDRs of 0.01/0.03 (high/medium). The peptide/protein filter node retained only peptides with at least medium confidence that were minimum 6 amino acids in length, and only proteins matched by rank 1 peptides. Peptides were counted only for the top scoring proteins. The strict/relaxed confidence thresholds for the protein FDR validator node were set again to 0.01/0.03, and the strict parsimony principle was enabled for the protein grouping node.

### Statistical and Bioinformatics Analysis of MS Data

For each of the 24 biological samples (cytokine-treated and control cells, serum-deprived and non-deprived, nuclear and cytoplasmic fractions, three biological replicates of each), three LC-MS/MS technical replicates were generated and combined during the database search process to produce one multiconsensus search file. The use of technical replicates enabled an increase in the number of identified proteins, in the confidence of protein identifications, and in the consistency and reproducibility of proteomic profiling results among biological replicates. A global multi-consensus report, compiled from the 72 MS-MS experiments performed for the 24 samples, was then generated by using Proteome Discoverer 2.4 and the results were aligned with the *Homo sapiens* database IDs for downstream analysis. Fold-changes (FC) for differentially expressed proteins between cytokine-treated and non-treated cells were assessed based on spectral count data, and statistical significance was calculated by performing a t-test. For each comparison, data normalization was performed based on the average of total spectral counts of the six samples taken into consideration, i.e., of the three control and three cytokine-treated biological cell replicates. The resultant normalization factor was used to normalize the counts of each individual protein in the six samples. Missing values in the proteomics data (i.e., proteins with zero counts) were handled by adding a spectral count of 1 to each protein. Differentially expressed proteins were then derived by calculating the Log_2_(Treatment/Control) spectral count ratios, and computing a two-tailed t-test for each protein. Proteins matched by two distinct peptide sequences with a 2-FC in spectral counts, i.e., Log_2_(Treatment/Control)≥0.9 or ≤(-0.9) with a p-value<0.05, were selected for differential expression analysis. These proteins were then interpreted in a biological context using bioinformatics tools provided by online data processing packages such as DAVID 6.8 (www.david.ncifcrf.gov) (37), STRING 11.0 (www.string-db.org) (38), KEGG (https://www.genome.jp/kegg/) (39) and Reactome (https://reactome.org) (40). Functionally related protein lists were created based on controlled vocabulary terms from UniProt (https://www.uniprot.org/) (41), and protein functionality was extracted either from the UniProt or GeneCards (https://www.genecards.org/) (42) databases. Biological pathways were created with BioRender (https://biorender.com/) and dendograms with RAWGraphs (https://rawgraphs.io) (43). PRM data analysis was performed with Skyline (https://skyline.ms) (44), by using a mass spectral library created from the DDA-MS raw files. Peptides with 5 transitions that displayed a dot product (*dotp*) score >0.9 were considered for validation.

## Results

Glioblastoma and brain metastasis model systems have shown that the immune response of microglia cells is greatly compromised in the presence of cancer cells that engage in a cross-talk with microglia to induce the growth and survival of the former. In this study, we hypothesized that the HMC3 cells, upon stimulation with anti-inflammatory cytokines (IL-4, IL-13, IL-10, TGFβ, CCL2) released from cancer cells, will show an increased expression of immuno-suppressive and tumor-supportive proteins and associated pathways. As resident immune cells of the brain, microglia cells have an extremely slow turnover rate throughout adulthood, making them one of the slowest dividing cells of the immune system (45). The “resting” and “activated” states of microglia were mimicked with HMC3 cells cultured in the absence and presence of FBS and cytokines, respectively. Serum deprivation was also used to provide a basal cell state level for enabling a more accurate observation of cytokine-stimulated behavior without interference from growth promoting factors from serum.

### Qualitative Assessment of Data

**Figure 1:**
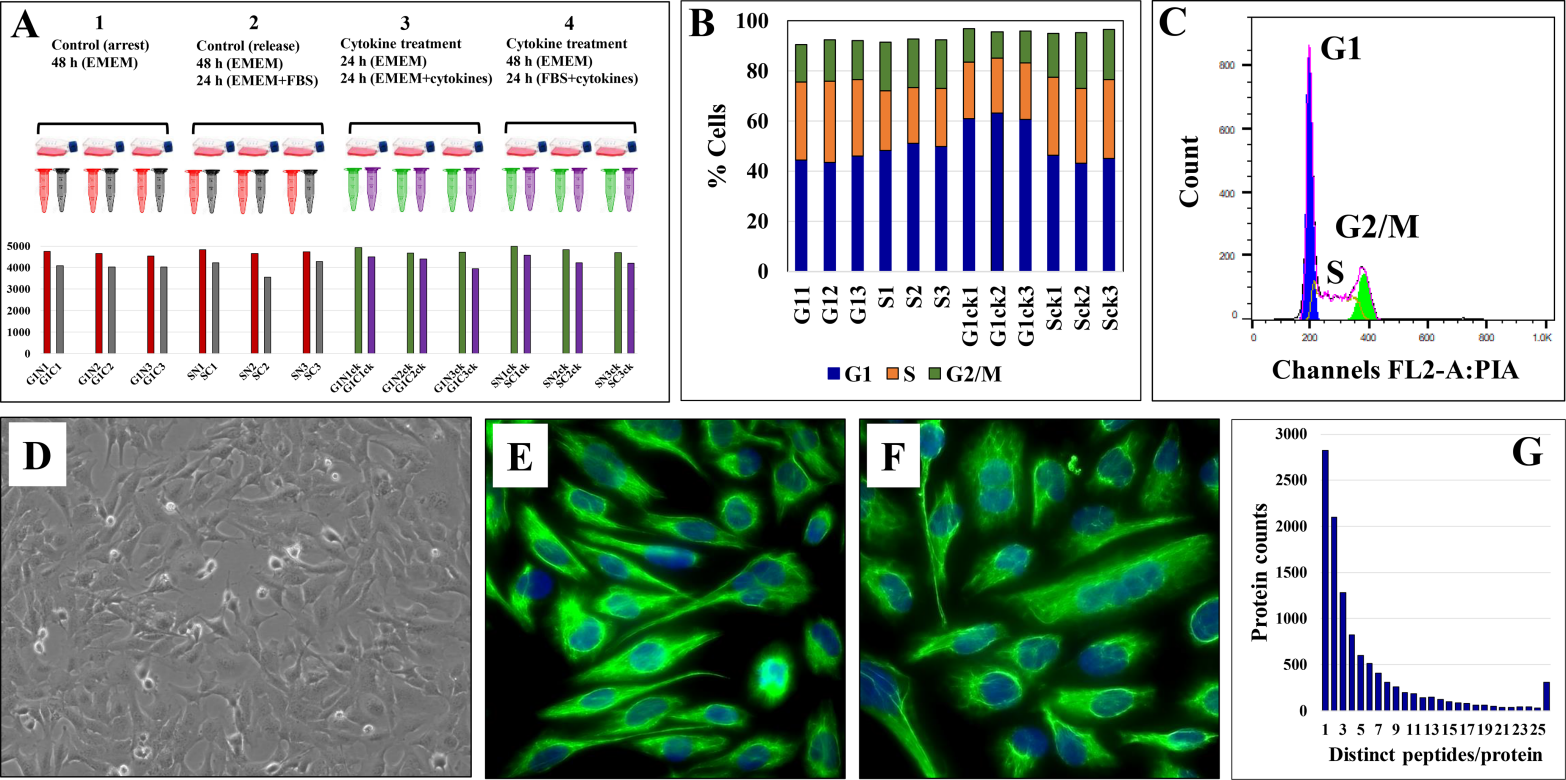
Evaluation of HMC3 cell culture protocol. **(A)** Experimental setup for cell analysis. Three biological cell replicates were cultured in serum-rich and serum-free media, in the presence and absence of cytokines. Cells were fractionated in nuclear (N) and cytoplasmic (C) fractions and analyzed by LC-MS/MS to yield ~4,000 protein IDs per 3 replicate/combined injections. **(B)** Stacked bar graph of HMC3 cell cycle distributions in the various experimental cell states. **(C)** Representative FACS analysis plot of HMC3 cells: G1 45-50%, S 22-30%, G2/M 10-20%. **(D)** Phase contrast microscopy image of proliferating HMC3 cells (20X magnification). **(E)** Immunofluorescence image of resting HMC3 cells stained for vimentin cytoskeletal filaments with XNAMEX primary antibody and secondary mouse IgG antibody conjugated to CruzFluor488 (green fluorescence); nuclei stained with DAPI (blue fluorescence). **(F)** Immunofluorescence image of activated HMC3 cells (same conditions as in E). **(G)** Histogram of protein counts distributed based on the number of matching unique peptides.

The experimental setup along with sample annotations are depicted in **Figure 1A**. Serum-deprived cells were denoted as G1 states, non-deprived cells as S states, and the cytokine-treated cells by a subscript “ck.” Serum deprivation was expected to generate a G1 cell cycle arrest-like state, while culture in the presence of FBS, a state typical to cells in the S-stage of the cell cycle. FACS analysis, however, revealed that the proportion of G1 and S cells did not change significantly during the different treatment regimens (~45-50% cells in G1 and ~22-30% in S; CV=3-11%; **Figures 1B, C**). Major morphological differences between serum-deprived and non-deprived cells were not observed either, fluorescently labeled cells for vimentin - a cytoskeletal mesenchymal type III intermediate filament protein in microglia - confirming a similar morphology for the two cell states (46) **(** **Figures 1D–F** **)**. As microglia cells were shown to upregulate autophagic behavior in response to serum starvation (47), the lack of cell cycle arrest was not unexpected. Oncogenic transformation as a result of primary cell transfection could have been an additional contributing factor (48, 49). Nevertheless, cytokine-treated cells in serum-deprived medium exhibited a larger proportion of G1 cells (~60%, **Figure 1B**), which, as will be discussed later, was an expected result of the addition of cytostatic TGFβ to the cell culture (50).

**Figure 2:**
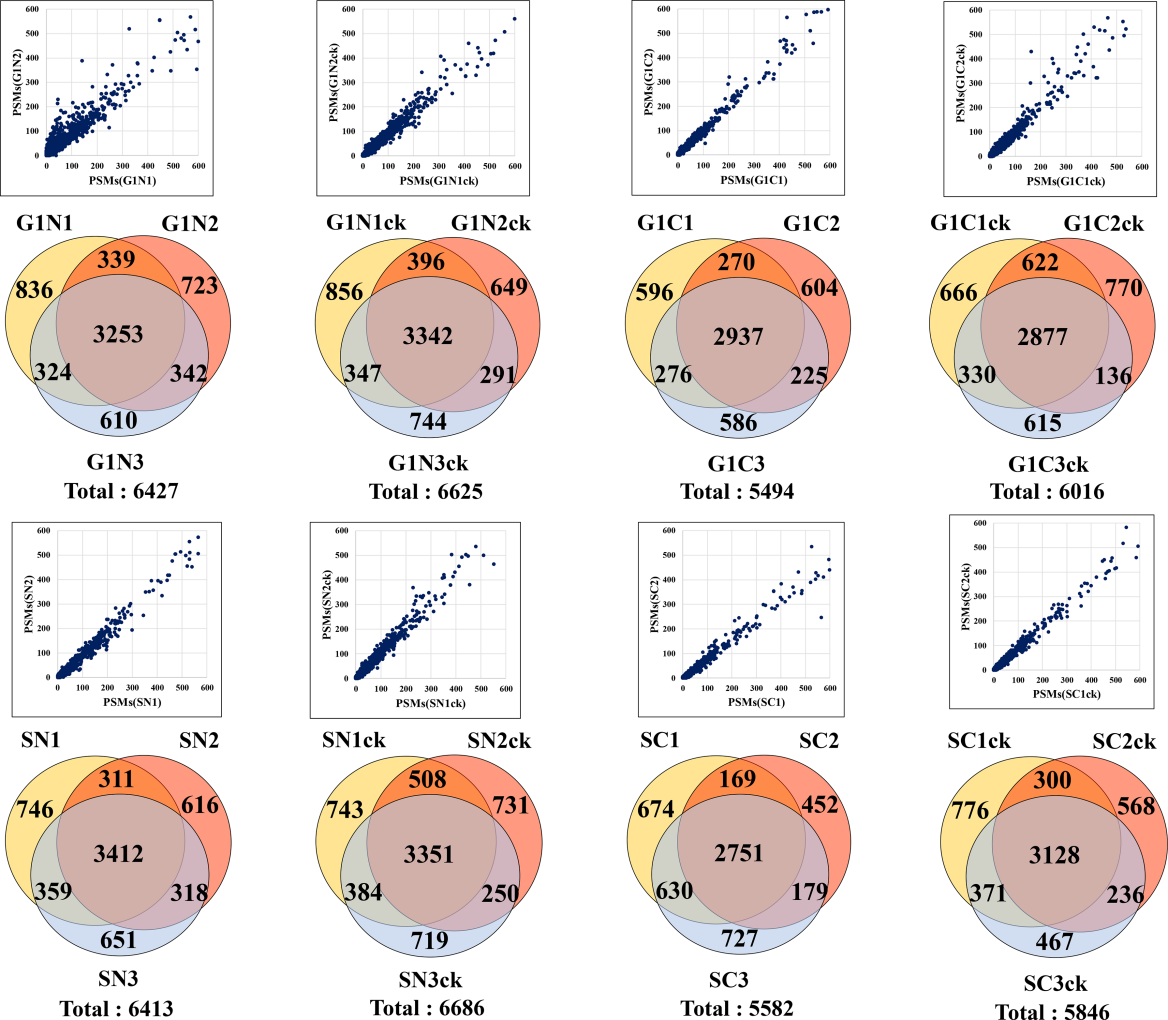
Reproducibility of protein identifications in microglia cells. Scatterplots show the correlation between the PSMs in two biological replicates, and the Venn diagrams show the protein overlaps in three biological replicates of G1, S and nuclear and cytoplasmic cell fractions.

The combined analysis of cytokine-treated (G1ck, Sck) and untreated (G1, S) HMC3 cells led to the identification of a total of 10,832 high (1% FDR) and medium (3% FDR) confidence protein groups, with an average of 4,463 (CV=8%) protein groups per each G1 or S cell state and nuclear or cytoplasmic fraction, and 6,136 (CV=7.6%) protein groups for three combined biological replicates of each cell state (**Figure 1A** and **Supplemental Table 1**). **Supplemental Table 2** provides a description of the gene abbreviations used throughout this study. As many as 75% of the proteins were matched by two or more distinct peptides (**Figure 1G**). Scatterplot correlation diagrams between the peptide spectrum matches (PSMs) of a protein in two biological replicates (Pearson correlation coefficient R>0.98), and Venn diagrams of protein overlaps in three biological replicates (~50% overlap), illustrate the reproducibility of protein identifications (**Figure 2**).

**Figure 3:**
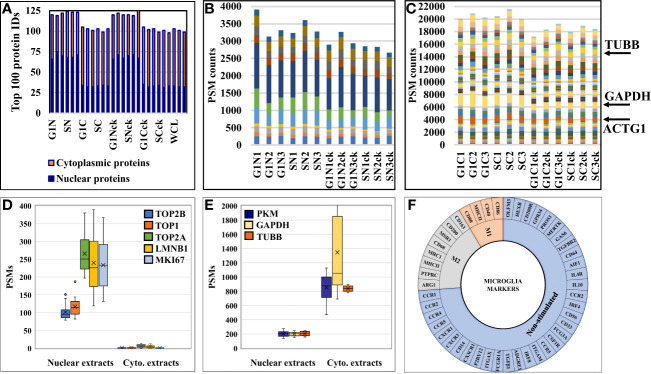
Charts representing the detection reproducibility of various protein marker categories in HMC3 cells. **(A)** Nuclear enrichment process outcome. The number of proteins with nuclear association in the top 100 most abundant proteins from the dataset is shown. Due to dual cellular component assignments, the sum of the nuclear and cytoplasmic proteins in the nuclear cell extracts exceeds 100. **(B)** Nuclear barcode proteins (11) displaying stable PSMs in the various nuclear enriched cell fractions (51). **(C)** Cytoplasmic barcode proteins displaying stable PSMs in the various cytoplasmic cell fractions. Beta tubulin (TUBB), glyceraldehyde 3-phosphate dehydrogenase (GAPDH) and cytoplasmic actin gamma 1 (ACTG1) are classical cytoplasmic marker proteins. **(D)** Abundance of nuclear markers in the nuclear and cytoplasmic cell fractions (topoisomerases-TOP1, TOP2A and TOP2B; lamin B1-LMNB1; and proliferation marker protein-MKI67). **(E)** Abundance of cytoplasmic markers in the nuclear and cytoplasmic cell fractions (TUBB, GAPDH, pyruvate kinase isozymes M1/M2-PKM). **(F)** Sunburst chart of microglia marker proteins associated with immune-activation and cytokine signaling processes.

The effectiveness of the nuclear protein enrichment step was assessed based on subcellular protein assignments provided by UniProt in the top 100 proteins with the largest number of distinct peptide counts (**Figure 3A**). On the average, the nuclear-enriched cell extracts contained ~70% nuclear proteins, *vs.* ~33% in the cytoplasmic fractions and whole cell/non-enriched lysates. The results were consistent throughout the entire dataset. Overlaps between the nuclear and cytoplasmic proteins were observed and expected to occur, due to the interference of the nucleocytoplasmic shuttling processes, linkage of the cytoskeletal to the nucleoskeletal proteins (52), and the association of various cytoplasmic components with the nuclear material (i.e., transcription factors, signaling molecules, gene modulators, etc.). The reproducible detection of a specific set of nuclear and cytoplasmic “barcode” proteins that were shown previously to be useful for data normalization and validation in quantitative proteomic experiments (51), as well as of a few proteins used as controls in routine biological studies (e.g, tubulin, actin, GAPDH), further corroborated the quality of the data and reproducibility of the nuclear/cytoplasmic separation process **(** **Figures 3B, C** **)**. The barcode proteins were shown to display a consistent number of spectral counts irrespective of the treatment performed on cells (51), and displayed similar behavior in these experiments, as well. In contrast, classical nuclear and cytoplasmic markers were identified in proportion to their abundance in the expected cellular fraction **(** **Figures 3D, E** **)**.

### Microglial Markers in HMC3

A first priority in analyzing the HMC3 proteomic data revolved around assessing whether the cells presented protein signatures characteristic of microglia function and/or M2 polarization. Microglia have been extensively characterized for their cell surface markers to define their physiology and distinguish them from peripheral macrophages in the brain. Over 50 proteins have been catalogued to define different states of human microglia, most of which being associated with immune-activation and cytokine signaling processes (22, 53) (**Figure 3F**). In this study, we identified 14 of these proteins, mainly associated with the plasma membrane, with known roles in regulating proliferation, differentiation, and immune function in cells of the myeloid lineage **(** **Supplemental Table 3** **)**. One of the most relevant proteins indicative of M2 polarization was the macrophage scavenger receptor MSR1 (CD204) (4) that was identified by three unique peptides (7 PSMs) in the cytokine-treated cells, and by only one peptide (1 PSM) in the non-treated cells. MSR1 is a cell membrane glycoprotein involved in host defense and microglia activation during neuroinflammation, and has scavenger activity in macrophages. Other markers such as the receptor-type tyrosine-protein phosphatase C (PTPRC or CD45) and arginase 1 (ARG1) (4) were identified in various cell fractions, but only by a single peptide and PSM. PTPRC has essential roles in regulating T- and B-cell antigen receptor and cytokine signaling processes, and ARG1 is a cytoplasmic enzyme of the urea cycle with roles in innate and adaptive immune responses and IL-4 signaling events (42). Markers of M1 polarization such as CD40, CD80, and CD86 (4, 11, 54), which are expressed upon treatment with pro-inflammatory cytokines, were either not detected or detected with very low spectral counts (e.g., MHCII/HLAII). On the other hand, proteins that have been shown previously to be highly expressed or unique to microglia over other immune cells were consistently observed in both untreated and cytokine-treated fetal microglia cells (55, 56). These proteins included the purinergic receptor (P2RY12), anticoagulant plasma protein (PROS1), receptor tyrosine kinase (MERTK), olfactomedin-like protein 3 (OLFM3), beta-hexosaminidase subunit beta (HEXB), and the Fc receptor-like proteins (FCRLs). Moreover, proteins that were part of a top ten adult microglia signature protein set, and that could not be identified in previously analyzed microglia cell lines (55), were identifiable in the human fetal brain-derived HMC3 cells [P2RY12, PROS1, MERTK, FCRLs, apolipoprotein E (APOE), sal-like protein 1 (SALL1), and integrin beta 5 (ITGB5)]. A few specific receptors, typically observable in non-stimulated or resting microglia cells, are worth additional comments. Remarkably, CD33, which belongs to the Siglec (sialic-acid-binding immunoglobulin-like lectin) family of molecules with roles in inhibiting immune cell activation and maintaining the cells in a resting state (57), was present only in the untreated HMC3/S cells but absent in the cytokine-treated cells. Enzyme-linked receptor proteins such as MERTK, PTPRC, colony stimulating factor 1 receptor (CSF1R), and transforming growth factor beta receptor 2 (TGFBR2) were present in both treated and untreated datasets. These receptors have various regulating roles in cell proliferation, differentiation, survival, apoptosis, and inflammatory responses. *Via* MAPK, STAT and TGFβ signaling are also involved in oncogenic cell transformation. Other markers included three G-protein coupled receptors, i.e., the adhesion G protein-coupled receptor E1 (ADGRE1) involved in cell adhesion and immune cell-cell interactions, and the C-C chemokine receptors type 4 and 5 (CCR4 and CCR5) with roles in chemokine signaling in macrophages. Also, the resting HMC3 cells were categorized by ATCC as negative for the GFAP (Glial fibrillary acidic protein) and positive for IBA1 (AIF1-Interferon gamma responsive transcript) markers. IBA1 was not detectable in the cells, but AIF1L (Allograft inflammatory factor 1-like), an AIF1 paralog, was upregulated in the serum-depleted/cytokine-treated cells. It was also interesting to note the substantial upregulation of GFAP in all cytokine treated cells. GFAP is a cytoskeletal protein, typically considered specific to astrocytes during the development of the CNS. Upon astrocyte activation, it was shown that GFAP expression is increased to inhibit inflammatory responses and reduce oxidative stress (58).

### HMC3 Surfaceome

**Figure 4:**
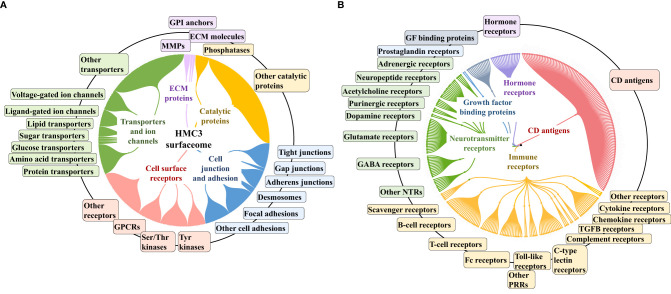
Dendograms representing the HMC3 microglia surfaceome. **(A)** HMC3 cell membrane proteins represented by receptors (Tyr and Ser/Thr receptor and nonreceptor kinases; olfactory, frizzled, taste, neurotransmitter and adhesion GPCRs), transport proteins (ligand and voltage-gated ion channels, transporters of small and large molecules), cell adhesion and cell-cell (tight junctions, gap junctions, adherent junctions and desmosomes) or cell-matrix (focal adhesion) junctions, and proteins with various catalytic functions (857 proteins with two distinct peptides, out of 1314 total cell membrane proteins detected; ~18% present in multiple categories). **(B)** HMC3 cell membrane receptors and CD antigens. A total of 283 proteins were assigned to signaling processes triggered by immune receptors (cytokine, chemokine, PRRs, complement receptors, Fc, T-cell, B-cell, and scavenger receptors), neurotransmitter receptors, hormone and prostaglandin receptors, glycan sensors (C-type lectin and Siglecs), and other growth factor binding proteins and receptors.

To gain an insight into the HMC3 cell surface proteome and its potential immunological implications, relevant categories such as receptors, cell surface antigens, transporters, and cell junction and adhesion proteins were extracted from the dataset by using UniProt keyword and GO controlled vocabulary annotations (**Figure 4** and **Supplemental Table 3**). From the total of 10,832 proteins, ~8,000 were matched by two or more distinct peptides, and could be assigned to the general nuclear (2,895), cytoplasmic (3,026) and cell membrane (977) cellular sub-fractions. The HMC3 surfaceome, represented by 857 proteins that could be mapped to specific cell membrane annotations, was captured in the dendogram shown in **Figure 4A**. This protein set represents ~23% of the UniProt annotated *Homo sapiens* cell membrane proteins (3,695), and could be assigned to all major categories, i.e., Tyr and Ser/Thr receptor and nonreceptor kinases, GPCRs, transporters, cell-cell and cell-matrix junctions, and to other matrix binding and interacting proteins comprised of GPI-anchored, matrix metalloproteases and scaffolding extracellular matrix glycoproteins.

**Figure 5:**
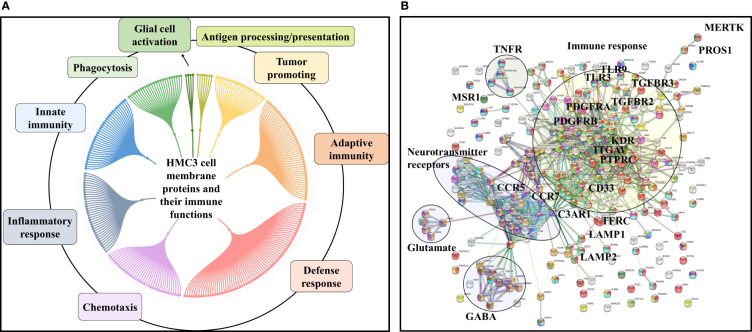
Immune response processes triggered by HMC3 cell membrane receptors and CD antigens. **(A)** Dendogram of major processes: innate and adaptive immunity responses, defense/inflammatory responses, chemotaxis, phagocytosis, secretion of immune modulators, antigen presentation, and tumor support. **(B)** STRING PPI diagram of the HMC3 receptors and CD antigens: red-immune system process, green-innate immune response; yellow-adaptive immune response, blue-inflammatory response, purple-chemotaxis; dark green-phagocytosis, light blue-defense response, dark yellow-chemical synaptic transmission, dark purple-glutamate receptor pathway, brown-gamma aminobutyric acid signaling pathway.

The entire landscape of specialized cell membrane receptors (growth factor binding, immune, neurotransmitter, and hormone receptors) and CD antigens that initiate and carry out various signaling functions was represented in the HMC3 cells. The different categories are highlighted in the dendrogram from **Figure 4B**, and the immune response processes that could be potentially triggered by the stimulation of cells *via* these receptors, as well as the complex protein-protein interaction (PPI) networks between these receptors, in **Figure 5**. Infectious agents, pathogens, damaged/apoptotic or cancerous cells in the CNS release a variety of factors that trigger an inflammatory response from the host. Additional release of chemokines provokes receptor mediated signaling processes that promote the targeted movement of microglia toward the site of pathogenesis (chemotaxis), recognition of specific molecules on the surface of the target, and initiation of an immune response that eliminates the perturbation source, cellular debris and neurotoxic agents (phagocytosis). Chemotactic migration of microglia and solicitation of an immune response is the result of a complex interplay between purinergic receptors, ion channels, neurotransmitters and Tyr kinase signaling (59, 60). Neurotransmitter receptors (glutamate, GABA, acetylcholine) modulate a variety of microglia functions, including activation, phagocytic clearance and polarization (61). GABA, acetylcholine and adrenergic receptors modulate and limit the release of inflammatory molecules and exert neuroprotective functions (60, 61). Stimulation of glutamate receptors has been shown to induce actin cytoskeleton rearrangements to promote chemotaxis and phagocytosis, and a separate group of the glutamate family of receptors modulate neurotoxicity or have neuroprotective functions (62). Adrenergic receptors have been shown to control M2 activation by negatively affecting the expression of inflammatory IL-6, NO and TNFα in the cells (60, 61, 63). Microglia use cell surface pattern recognition receptors (PRRs) such as toll-like receptors (TLRs) and C-type lectin receptors (CLRs) complexed with scavenger receptors (CD204, CD14, MERTK, AXL receptors) to detect and bind to specific pathogen-associated molecular patterns (PAMPs) on the surface of pathogens. Alternatively, microglia use TREM-2 receptors to recognize damage-associated molecular patterns (DAMPs) of apoptotic neural and other dead or damaged cells to initiate phagocytic clearance of the captured particles (64–66). Phagocytosis is further regulated by Fc, complement, or purinergic receptors (67). Together, these proteins are responsible for detecting extracellular cues and orchestrating the signal transduction process that triggers a corresponding cellular response (68, 69), and represent the focus of many drug target discovery efforts for therapeutic interventions. The interplay between the various receptor systems bridge innate and adaptive immune responses implicated in defense, inflammation and cytokine production, *via* the activation of various signaling pathways such as TLR, MAPK, ERK1/2, PI3K/AKT, JNK, JAK/STAT, and NFKB. These capabilities were all supported by the receptors identified in the HMC3 surfaceome.

### Comparative Assessment of Differentially Expressed Proteins

**Figure 6:**
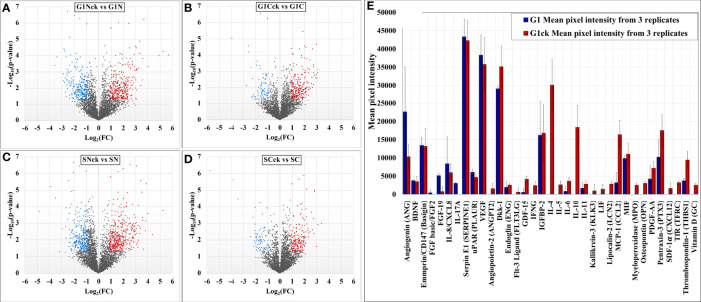
Volcano plots depicting changes in the spectral count of proteins associated with differentially modulated biological processes in cytokine-treated *vs* non-treated HMC3 cells **(A–D)**. Proteome Profiler cytokine array results generated from G1 and G1ck cell culture supernatants **(E)**.

**Figure 7:**
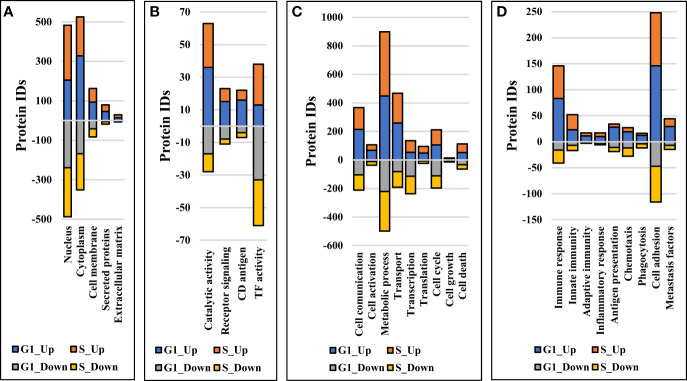
Stacked bar charts representing proteins that changed expression or activity level and could be associated with general biological categories related to: **(A)** Cellular location, **(B)** Receptor/catalytic or TF activity, **(C)** Biological processes, and **(D)** Immune response.

**Figure 8:**
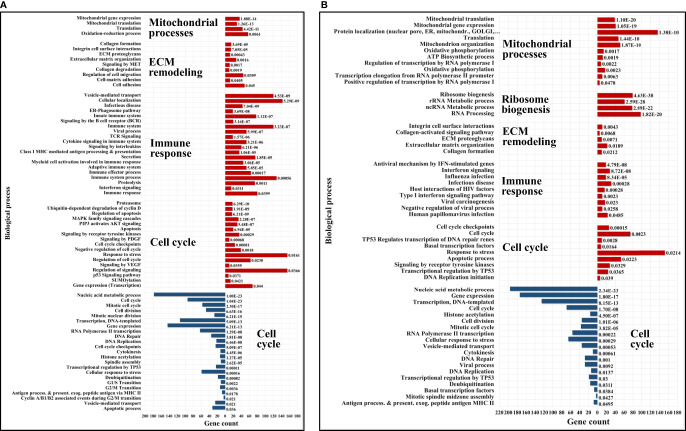
Bar charts of selected enriched up- (red) and down- (blue) regulated biological processes and pathways in cytokine-treated *vs* non-treated HMC3 cells. **(A)** G1ck *vs* G. **(B)** Sck *vs* S. Annotations are based on BP, KEGG and Reactome databases. Full lists of enriched categories are provided in **Supplemental Table 5**.

Comparative proteomic analysis was performed between cytokine-treated and non-treated cells, in the G1 and S cell states. The Volcano plots from **Figures 6A–D** depict the respective comparisons, i.e., G1Nck *vs* G1N, G1Cck *vs* G1C, SNck *vs* SN, and SCck *vs* SC, with up/downregulated biological processes encompassing protein counts of 433/307, 332/113, 484/374 and 260/102, respectively. Some cell membrane proteins were detected in the nuclear fractions due to co-precipitation during centrifugation. Only proteins identified with a minimum of two unique peptides that passed the statistical filtering criteria were considered for downstream bioinformatics analysis. Complementary quantitative results provided by the Proteome Profiler 105 cytokine array, acquired from the G1 and G1ck cell culture supernatants, were included in the bar chart from **Figure 6E**. The biologically relevant differences arising due to the treatment of cells with anti-inflammatory cytokines were explored using STRING, DAVID, KEGG, Reactome, UniProt and GeneCards bioinformatics identification, annotation and enrichment tools (**Figures 7** and **8**). **Supplemental Tables 4** and **5** include the full lists of proteins, enriched protein categories, and associated networks and pathways, and **Supplemental Table 6** provides a categorized list of the proteins that were selected for discussion. For a cohesive biological interpretation, enriched GO categories from the nuclear and cytoplasmic fractions of a cell state were combined and discussed collectively. In addition, categories of relevance to immune-response were explored individually (**Figure 7**). Up- and downregulated proteins and biological processes were observed in all cell states (**Figure 7A**), with observable impact from cell-surface-induced to transcription factor activities (**Figure 7B**), and output affecting cellular communication/activation, transcription/translation, transport, metabolism, cell cycle, growth, cell death (**Figure 7C**), and various immune responses (**Figure 7D**). General categories related to nucleic acid, protein or small molecule metabolism, as well as transport and signaling, encompassed large protein groups that were observable in all comparisons that were explored. PRM validation results for selected proteins in the biological replicates of the control and stimulated samples, displaying the changes in peptide peak areas associated with these proteins, were included in **Supplemental File 1**. As regulation of protein activity and function is often achieved at the post-translational level, the addition or removal of PTMs alters the detectability of proteins. This can be especially the case of proteins involved in signal transduction and cell cycle regulation, for which the observed changes may be representative of changes not just in expression level but also in activity. Specific categories that are discussed below were selected based on relevance to the cytokine treatment that was performed.

## Discussion

### Upregulated Biological Processes in Serum-Depleted/Cytokine-Treated Cells

**Figure 9:**
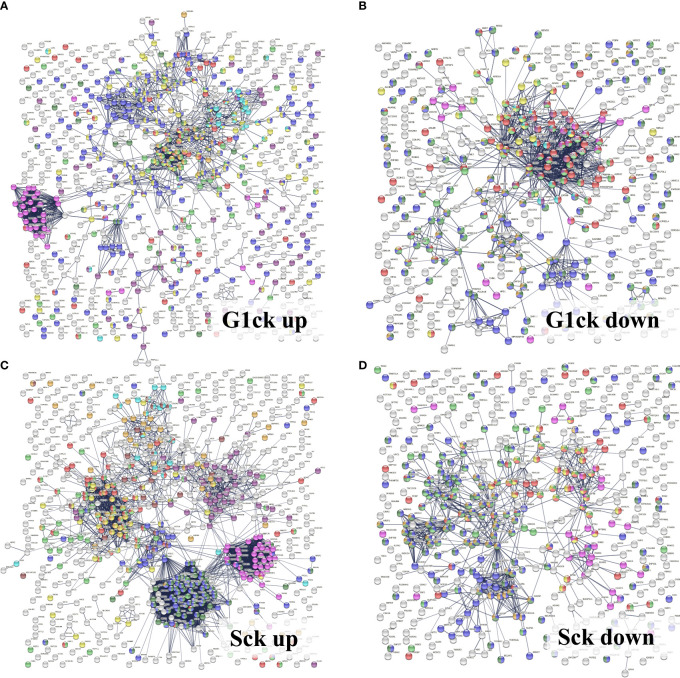
STRING PPI networks representing up- and downregulated biological processes in cytokine-treated *vs*. non-treated HMC3 cells. **(A)** Upregulated G1ck *vs*. G1 (728)/Details provided in **Figure 10**: Magenta-Mitochondrial gene expression (translation) (36); Purple-Oxidation reduction process (60); Yellow-Immune system process (127); Red-Regulation of cell cycle (63); Green-Proteolysis/phagosome pathway/FCERI mediated NFKB activation (74); Turquoise-ECM organization (27); Blue-Transport/vesicle mediated transport (228); Dark yellow-MAPK family signaling (33); Dark green-Regulation of cell migration (44). **(B)** Downregulated G1ck vs G1 (403): Red-Cell cycle (94); Green-Chromosome organization (82); Blue-Gene expression/transcription (144); Yellow-DNA repair (35); Dark green-Transcription DNA templated (113); Dark yellow-Transcription RNA pol II (40); Purple-M phase (23); Turquoise-S phase (13); Magenta-Membrane trafficking (24). **(C)** Upregulated Sck *vs* S (714): Magenta-Mitochondrial gene expression/translation (mitochondrial ribosomes) (41); Purple-Mitochondrial organization/oxphos, ATP biosynthesis, respiratory electron transport (51); Dark green-Ribosome biogenesis (79); Blue-RNA processing, rRNA/ncRNA (98); Yellow-Viral processes/Interferon signaling (51); Red-Cell cycle (74); Green-Chromosome organization (67); Turquoise-ECM organization (22); Dark yellow-PTMs (69); Brown-Signaling by RTK/cytoskeletal rearrangements, membrane organization, integrin cell surface interactions (28). **(D)** Downregulated Sck *vs* S (465): Red-Cell cycle (69); Green-Chromosome organization (77); Blue-Gene expression/transcription (174); Yellow-Mitotic cell cycle process (36); Dark green-Transcription DNA templated (125); Dark yellow-RNA polymerase transcription II; (56) Magenta-Membrane trafficking (35). See also **Supplemental File 2** for enlarged images and gene descriptions.

**Figure 10:**
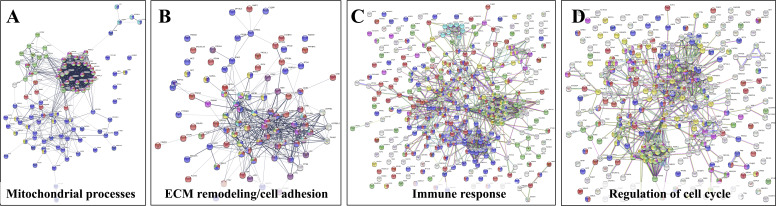
Detailed STRING PPI networks representing the upregulated biological processes in serum-depleted/cytokine-treated HMC3 cells (G1ck *vs* G1). The networks were built based on the selected enriched protein categories shown in **Figure 8**. **(A)** Mitochondrial gene expression and redox processes: Red-Mitochondrial gene expression (36); Blue-Oxidation-reduction process (57); Green-Translation (42); Magenta-Mitochondrial translation (30); Yellow-Fatty acid metabolic process; (9) Turquoise-Collagen formation (4). **(B)** ECM remodeling and cell adhesion: Red-Cell adhesion (48); Blue-Regulation of cell migration (45); Purple-Extracellular matrix organization (27); Yellow-Regulation of MAPK cascade (15); Magenta-Signaling by Met (11); Turquoise-Signaling by PDGF (9); Green-Signaling by VEGF (9). **(C)** Immune response: Red-Immune system process (128); Blue-Vesicle mediated transport (120); Green-Proteolysis (74); Magenta-Protein localization to the nucleus (19); Turquoise-Mitochondrial ATP synthesis coupled proton transport (8); Yellow-Cytokine signaling in immune system (53); White-Localization. **(D)** Regulation of cell cycle: Red-Regulation of cell cycle (63); Blue-Regulation of apoptotic processes (73); Green-Proteolysis (55); Yellow-Immune system (83); Magenta- Viral processes (39); White-response to stress/signaling processes/localization. Selected proteins associated with the PPI networks are provided in **Supplemental File 2**.

The upregulated processes in the serum-depleted/cytokine-treated cells were represented by a total of 728 proteins clustered into four broad biological categories, i.e., (a) mitochondrial gene expression and oxidation-reduction, (b) ECM remodeling and cell adhesion/migration, (c) immune response, and (d) regulation of cell cycle. **Figure 8A** provides a representative selection of these biological processes, **Figure 9A** a STRING PPI network of the whole protein set, and **Figure 10** a breakdown of the PPI network into the above four categories. The following discussion of biological processes is based on these networked interactions.

#### Mitochondrial Gene Expression and Oxidation-Reduction

Cells produce energy by oxidizing their carbon fuels in the TCA cycle and generate a proton-motive force by repeated oxidation-reduction cycles to generate ATP in a process known as oxidative phosphorylation (OXPHOS). This process occurs in the mitochondria. Noticeably, the cytokine-treated HMC3 cells showed an upregulation of mitochondrial gene expression reflected by proteins involved in mitochondrial transcription, mt-RNA processing and translation, and an increased expression of 25 mitochondrial ribosomal (MRP) protein subunits including 18 large and 7 small (**Figure 10A**). These results were complementary to the identification of various protein complex subunits implicated in mitochondrial and cytoplasmic oxidation-reduction reactions, oxidative phosphorylation, cellular response to oxidative stress, cell detoxification, and lipid/organophosphate/organonitrogen compound metabolism. It is now well established that IL-4 treated M2 microglia are characterized by lower glucose consumption by switching to oxidative phosphorylation which is fueled by higher rates of fatty acid oxidation (FAO) (70). Accordingly, an upregulation of mitochondrial enzymes that are involved in FAO was observed. In addition, the reactive oxygen species (ROS) generated during oxidative phosphorylation were balanced by an upregulation of mitochondrial and cytoplasmic oxidoreductases which protect the cells during oxidative stress.

#### ECM Remodeling and Cell Adhesion/Migration

Increased mitochondrial respiration in response to IL-4 is correlated with switching the arginine metabolism from citrulline and nitric oxide production in M1 microglia to arginase-catalyzed production of ornithine and polyamines for collagen synthesis in M2 microglia (27, 30, 71). Subsequently, the prolyl-hydroxylase enzymes (P3H1, P3H3, P4HA2) which are key to collagen synthesis showed an increased expression in the cytokine treated cells. Indeed, HMC3 showed a substantial upregulation of collagens, collagen receptors (ITGB1/ITGA3, DDR2), and cell-collagen adhesion modulators such as the TGFβ induced protein (TGFBI). Collagen deposition and cell-collagen interactions has been linked to cell invasiveness, and in cancer cells, to enhanced metastatic abilities (72). The integrin ITGB1/ITGA3 heterodimers are receptors not just for collagen, but also for fibronectin, laminin and thrombospondin, and along with the ITGB4/ITGA6 receptors for laminin mediate invasiveness (42). TGFBI, that binds to ECM and integrins, on the other hand, has been shown to inhibit cell adhesion (42). Likely the result of co-stimulating the cells with TGFβ (27), MAPK signaling and regulation which is central to numerous cellular pathways, was noticeably altered in the cells. Along with MAPK, signaling by c-MET, PDGF, and VEGF were tightly interconnected to the upregulated processes related to ECM remodeling, collagen formation, cell-cell/cell-matrix adhesion and cell migration (**Figure 10B**). Together, the cell adhesion molecules, the cell surface proteins and receptors, and the cytoskeletal proteins and intracellular signaling effectors orchestrate cytoskeleton organization and remodeling, leading to enhanced cell migration. A deeper look at the extracellular enzymes (LOX, CTSB, CTSD), growth factors (FGF1, NGF), cytokines (AIMP1), and ECM molecules detected in the cells highlighted their role in the extracellular matrix dynamics. ECM reorganization, increased deposition of collagen and glycoproteins such as fibronectin (FN1) and tenascin (TNC), which were substantially upregulated in the treated cells, result in increased rigidity and tensile strength of tissue and favor tumor growth, progression and invasion (73, 74). ECM remodeling molecules (CTSB, CTSD, SPARC, SERPINE1) support tumor invasion and metastasis by interacting with, degrading and/or re-shaping the extracellular matrix (75). LOX expression, responsible for the oxidative deamination of Lys residues in collagen precursors, collagen crosslinking and increased matrix stiffening, was correlated with increased tumor adhesion and migration (73, 76), but also with possible roles in tumor suppression (42). Proteases (CTSB, CTSD) that participate in protein degradation and turnover activate tumorigenic invasion and metastatic pathways by cleaving signaling peptides, growth factors and hormones in the intra- and extracellular environment (77).

β-catenin (CTNNB1), an important downstream effector of canonical Wnt signaling, was found to be consistently upregulated in all cytokine treated fractions, but mostly in the nuclear ones, pointing toward Wnt signaling activation. It has been well documented that TGFβ also induces the activation of Wnt ligands (78), and SMAD proteins of the TGFβ pathway engage in a crosstalk with Wnt signaling proteins to release CTNNB1 from the GSK3β mediated destruction complex, stabilize its function, and regulate gene expression (79). Recent studies have also shown that Wnt ligands produced by cancer cells use β-catenin signaling to stimulate immunosuppressive TAMs that support tumor growth and metastasis, and to upregulate the production of anti-inflammatory IL-4 and TGFβ (80). However, both positive and negative regulators of the Wnt pathway were present alongside β-catenin, advocating for a complex method of β-catenin activation in these cells.

IL-4 stimulation of macrophages was shown to induce the expression of E-cadherin which is stabilized by β-catenin to regulate cell adhesion processes (81). E-cadherin was not detected in the cells, but the cytoplasmic fractions were highly enriched in the adherence junction cadherin modulators CDH2, CDH4 and CDHR3. Increased expression of cadherins boost homophilic cell-cell adhesion between cells, impeding therefore single cell migration, and facilitating grouped migration and cell motility on cadherin substrates. Cadherin molecules can also activate intracellular signaling pathways and alter the actin cytoskeleton *via* the various catenins (α, β and p120) that they bind (82). In addition, the overexpression of focal adhesion proteins such as integrins, their kinase regulator ILK, of talin modulators of integrin activation (TLN1/TLN2), and of vinculin (VCL), indicated that integrin mediated signaling was highly correlated with the ECM processes and involved in transducing the extracellular cues into biochemical intracellular signals that alter cytoskeleton organization and migratory capabilities. Integrins such as ITGAV serve as receptors for vitronectin (VTN), tenascin-C (TNC), thrombospondin (THBS1), fibronectin (FN1), fibrillin and laminin proteins, together augmenting cell adhesion and signaling.

Enriched protein clusters that regulate chemotaxis or phagocytosis (e.g., TLR, P2X, P2Y, Fc or CCRX receptors) were either not identified, or identified with very low counts. Nevertheless, several receptors, cell adhesion and migration proteins in the stimulated cells could be associated with chemotactic processes (PDGFRB, VGFR1, DPYSL2, CRMP1, MET, PALLD, ITGAV, BCAR1, FYN, L1CAM). In addition, the detection of AIF1L (albeit with low counts), a paralog of AIF1 with roles in inflammation, macrophage activation, migration and induction of phagocytosis (41), pointed to the presence of such propensities. Overall, the combined effect of the upregulated proteins clearly demonstrated the induction of a microglia phenotype with enhanced ECM-remodeling and migratory capabilities, that simultaneously can also direct the formation of a tumor-supportive ECM niche in the vicinity of the microglia cells (73, 83).

#### Immune Response

Proteins representative of immune reactions could be associated with innate, adaptive or inflammatory responses *via* cytokine signaling, class I MHC mediated antigen processing and presentation, chemotaxis, cell adhesion/migration, and vesicle mediated transport (**Figure 10C**). One of the mechanisms by which the type I and type II cytokine receptors signal is *via* the JAK/STAT pathway. The signal transducers and transcription activators STAT1 and STAT3 are known to get activated by interleukins and interferons (84, 85), and complementary and conflicting roles of STAT1 and STAT3 on cell cycle have been also documented. STAT1 negatively regulates cell proliferation, elicits anti-viral and anti-tumorigenic immune responses, and promotes apoptosis. STAT3 promotes cell survival, G1/S transition and proliferation, motility and immune tolerance (42, 86). It has been shown, however, that increased expression in STATs does not necessarily correlate with increased activity (87). JAK/STAT signaling pathways play critical roles in both innate and adaptive responses, with IL-4/IL-13 and IL-10 being known for downregulating NFKB and STAT1 activities, limiting the production of inflammatory cytokines, and driving an anti-inflammatory macrophage phenotype (28, 88). In response to IL-10 stimulation, STAT3 alone can mediate various cellular responses and inhibit the transcription and expression of pro-inflammatory cytokines (89), however, previous reports have suggested that in certain circumstances IL-10 can also activate STAT1 mediated signaling to instigate an inflammatory response (87). These complex trends were observable in the present dataset, as well, and manifested themselves by the induction of M2a (*via* IL-4/IL-13) and M2c (*via* IL-10) polarized populations of cells with tumor-promoting activities that encompassed type II inflammatory/Th2 responses in M2a cells, and, matrix deposition, ECM remodeling and suppression of immune responses in M2c cells (28). Interestingly, STAT1 which is typically associated with M1 polarization was found to be consistently upregulated in all cytokine-treated cells, but mostly in the G1Nck cell fractions, while changes in the activity of STAT3 were observable only in the G1Nck fractions. These changes appeared to be rather affected by the dephosphorylation of some of its matching peptides, including one that induces transcription upon phosphorylation at Y45 (90). Nonetheless, a protein that mediates STAT3 degradation was also present (TMF1). STAT6 that is involved in initiating cytokine-initiated STAT signaling, was detectable, but with too low counts to support conclusive assessments.

Along with STAT proteins, additional proteins participating in cytokine, integrin, MAPK, Wnt and TGFβ signaling (TAB1, ZFYVE9, SMAD, PAK1) were highly interconnected in orchestrating the same cytoskeleton re-organization, ECM remodeling, adhesion, migration and endocytic processes. TGFβ signaling was further represented by the early endosomal zinc finger motif containing protein ZFYVE9 that recruits SMAD2/SMAD3 to the TGFβ receptor and mediates the subcellular localization and transcriptional activity of SMADs. The concomitant downregulation of the SMAD nuclear interacting protein SNIP1, who’s overexpression has been shown to inhibit the formation of SMAD4 complexes and TGFβ signaling, was also reflective of TGFβ activation. NFKB did not emerge as being activated in the serum-depleted/cytokine treated cells, but changes in the counts of MYD88 (Myeloid differentiation primary response protein), an innate immune signal transduction adaptor involved in toll-like receptor signaling, was suggestive of altered NFKB signaling.

Class I MHC mediated antigen processing and presentation of ubiquitin-proteasome degraded proteins was represented by the HLA class I histocompatibility antigens (HLA-C Cw-8α and Cw-14α chains), aminopeptidases that trim HLA class I-binding precursors for presentation by MHC class I molecules, and proteins with roles in folding (TCP1, CCT2, CANX), ER associated degradation (DERL1), activation of innate response pathways (MYD88), and degradation of proteins in the lysosomes (CTSL) (91). The proteasome components and ubiquitin conjugating or ligating enzymes (UBE2K, UBE2Z, TRIMs/E3 ligases) were amply overexpressed, and part not just of the antigen processing cluster but essentially of all global innate/adaptive response processes pertaining to cytokine, Fc epsilon receptor, CLR, MAPK and NFKB pathways. Of relevance was the finding that components of the immunoproteasome that have essential functions in processing class I MHC peptides were present in both control and treated cells along with subunits of the constitutive 20S core (92). The immunoproteasome low molecular weight proteins (i.e., LMP2/PSMB9, LMP7/PSMB8 and MECL-1/PSMB10) were present concurrently with their standard proteasome counterparts (i.e., PSMB6, PSMB5 and PSMB7) and the proteasome activator complex subunits 1, 2 and 3 of the 11S regulator of the immunoproteasome (PSME1, PSM2 and PSME3). Such immunoproteasome components can be induced by pro-inflammatory cytokines, but in the case of HMC3 cells appeared to be expressed at the basal level.

The immune cluster included one additional overarching hub that comprised vesicular transport, viral and cellular localization proteins. Vesicular mediated transport plays a key role in a variety of immune related functions such as endocytosis of apoptotic cells and pathogens, exocytosis, cytokine secretion and antigen presentation. Main drivers of intracellular membrane trafficking, exocytosis and secretion (small Rab GTPases, GTPase activating ARFGAP1/3, EXOC4), Golgi vesicle transport (COPI coat and AP-4 complexes), fusion of vesicles to the target membranes (NAPA, STX12, YKT6) and transmembrane trafficking (TMED2/7/9/10), were all markedly overexpressed. Viral transport activities were mediated through integrin, proteasome and nuclear import processes. Several proteins such as GBP1, PML, SP100 nuclear antigen, and the mitochondrial antiviral signaling protein MAVS were present to mount defense responses. Representative proteins of the receptor-mediated endocytosis included DNAJC13, CANX, TFRC, LDLR and SPARC.

As the observed chemokines were detectable by only one peptide and one or two spectral counts, changes in chemokine expression could not be measured with confidence. Nevertheless, chemokines CCL22, CCL23, CXCL12, and CXCL6 were observable only in the cytokine treated cells, and CXCL1 and CCL25 in both control and treatment. CCR4, the chemokine receptor for CCL22, was also detected only in the cytokine treated cells, while receptors CCR5 and CCR7 only in the control. With roles in immune surveillance and inflammation response, CCL22 is a chemoattractant for activated T-lymphocytes, NK cells, monocytes and dendritic cells. Likewise, CXCL12 (SDF1) is an attractant for T-lymphocytes and monocytes, and CXCL1 and CXCL6 for neutrophils. CCL22 was shown to be part of the M2a macrophage repertoire induced after IL-4/IL-13 stimulation (28), with suppression of adaptive immunity in TAMs (7). CCL22 secretion also enables the attraction of pro-tumor immunosuppressive cells *via* interactions with CCR4 receptors on the surface of Th2/Treg cells, while autocrine generation of CXCL12 was shown to support tumor growth and metastasis (42) and modulate the differentiation of monocytes into a phenotype with immunosuppressive and proangiogenic characteristics (93). Similar was the case of the macrophage scavenger receptor M2 marker MSR1 (CD204) that displayed increased spectral counts in the serum/cytokine treated cells, but did not meet the stringent criteria that were utilized for considering protein upregulation. It was interesting, however, to observe the upregulation of the complement C1q binding protein (C1QBP), a multifunctional protein that is involved in mediating and controlling a very large number of cellular processes including transcription regulation, RNA processing, CDKN2A-mediated apoptosis, protein synthesis in mitochondria, mitochondrial OXPHOS, adhesion and chemotaxis. This protein can be found in various cell compartments, and when acting as a receptor, it can bind C1q molecules to inhibit the activation of the complement C1 complex, and therefore immune reactions (42). Its ability to interact with viral and bacterial proteins, makes it a critical regulator of anti-bacterial and anti-viral inflammatory responses (94).

Altogether, the analysis of the immune signaling cluster revealed that the cells exhibited a strong response to the cytokine treatment, manifesting mainly a tumor-supportive M2a/c phenotype that displayed pronounced collagen deposition and ECM remodeling processes, type II inflammatory responses, production/upregulation of angiogenic factors and processes (FGF1, PDGFRB, VGFR1), upregulation of protease inhibitors (SERPINE1), suppression of adaptive immunity (CCL22), expression of tumor growth and survival chemokines (CXCL12) but not of typical inflammatory cytokines (IL-1, IL-6, IL-12, TNF-α) (7), upregulation of scavenger markers (MSR1, LRP1), and display of mostly endocytic but not phagocytic activities. Simultaneously, STAT1 upregulation and the presence of pro-inflammatory components (MYD88, AIMP1) were observable. Anti-proliferative effects induced by TGFβ and possibly STAT1 were clearly manifested, as evidenced by the analysis of cell cycle regulating components (see below). The impact of adding CCL2 to the stimulating cocktail of cytokines was not clear. On one hand, CCL2 can elicit immunosuppressive effects and support tumor progression by promoting increased invasion through angiogenesis (95), while on the other, it can induce the production of pro-inflammatory cytokines in macrophages to augment their survival (96).

#### Cell Cycle Regulation

On a background of response to stress, a cluster of functionally altered proteins were representative of various aspects of cell cycle and check point regulation, G1/S or G2/M transition, and apoptotic processes (**Figure 10D**). Representative examples included proteins with functional roles in cell cycle arrest and negative regulation of growth (cyclin-CDK complex inhibitors CDKN1A/CDKN2A, GADD45GIP1, PML, ILK, TSG101, PHB, CEBPA), transcription activation inhibition (TMF1), epigenetic repression (HDAC2), general RNA polymerase II transcription inhibition (HEXIM1), and regulation of apoptotic processes (PML, SP100, STAT1/STAT3, HTRA2). Shared components of a complex network of signaling pathways provided the scaffold for communication between these biological processes (i.e., MAPK, RAF/MAP, PI3K/AKT, c-MET, VEGF, PTEN, Notch, NFKB, Wnt, CLR, TCR and B cell receptor pathways). Proteasomal degradation, as mainly represented by various 26S proteasome subunits and ubiquitin conjugating enzymes, was common to most signaling pathways. The upregulated proteasome components were associated with the nuclear fraction, mirroring prior findings that have shown that proteasome recruitment at the nuclear pore complex facilitates the regulation of transcription (97, 98) and cell cycle progression by catalyzing the degradation of key cell cycle regulatory proteins. The above processes were expected to occur as a result of TGFβ activities that induce G1/S cell cycle arrest *via* the expression of cycD/E-CDK4/6 inhibitors, and by making use of the ubiquitin-proteasome pathway (99). STAT signaling activated by cytokines has been also described to upregulate the expression of proteasomal subunits (100), and IL-4 induced STAT1 activation was shown to result in cell growth inhibition (101).

### Downregulated Biological Processes in Serum-Depleted/Cytokine-Treated Cells

The downregulated processes in the serum-depleted cells related to gene expression, transcription initiation by RNA polymerase II Mediator, cell division, and associated metabolic processes (**Figures 8A** and **9B**). This was evident from the genes and proteins that control G1/S or G2/M transition (CCNA2, CCNB1, CDK1, PLK1, and AURKB/AURKC), spindle assembly, DNA repair and replication, and cytoskeleton organization. The simultaneous upregulation of HEXIM1 (a general RNA polymerase II transcription inhibitor) and of the p16 (CDKN2A) and p21 (CDKN1A) inhibitors of cycD-CDK4/6 and cycE-CDK2 complexes corroborated these results. The cytokine treated/serum-depleted cells also had the highest proportion of G1 cells, which was anticipated based on earlier reports that have shown that TGFβ and IL-10 induce anti-proliferative effects and an anti-inflammatory response in microglia cells (50, 102, 103), and that TGFβ mediates cell cycle arrest in G1 by the induction of CDK inhibitors (99). The lower abundance of cyclin A2 (CCNA2), of G2-specific cyclin B1 (CCNB1), and of the nuclear proliferation marker MKI67 in the cytokine-treated cells was supportive of this result. In addition, the downregulation of a transcription factor regulating the expression of some immune and inflammatory response genes (CEBPB), of a scaffold protein involved in transcription regulation and innate immunity (PQBP1), and of several cell adhesion (VCAM1), cytoskeleton organization, transport and motility proteins with known chemotactic or phagocytic activity (e.g., SRC, ELMO2, PTPRG), was also observed.

A cytokine array analysis used for exploring the secreted proteins in the supernatant of serum-free cultured cells corroborated several findings from the cellular extracts (**Figure 6E** and **Supplemental File 3**). The upregulation of thrombospondin 1 (THBS1) and of the soluble transferrin receptor (TFRC) was confirmed by the cytokine array, and a few proteins showed a roughly constant level of abundance with both MS and microarray detection (MIF, BDNF). SERPINE 1 and basigin (BSG), which were detected in the cell extracts with increased counts, did not show a change in abundance in the cellular supernatant. An additional set of low abundance proteins that were either undetectable by MS, or detectable by few spectral counts and not quantifiable, were detected by the array as faint spots. Among these, the components with increased abundance could be classified into anti-inflammatory cytokines (GDF15), cytokines with dual inflammatory and anti-inflammatory function (IL-6, LIF, IL-11, LCN2, THBS1), and inflammatory cytokines and factors (IL-5, IFN-γ, OPN, MPO). Various other components such as proteins with cytokine activity (FLT3LG), growth factor activity (PDGFAA, ANGPT2, ENG, DKK1), chemokines (CXCL12/SDF1α, OPN), endocytic factors (TFRC), proteases (KLK3), and vitamins (D) were also observable. The components with decreased abundance on the array included pro-inflammatory cytokines (IL-17A and IL-8), a fibroblast growth factor (FGF-19), and angiogenin (ANG). Altogether, the upregulated proteins on the array represented pathways such as JAK/STAT, PI3K-AKT, MAPK, TGFβ and focal adhesion, and confirmed the presence of both pro- and anti-inflammatory trends pinpointed by mass spectrometry.

### Upregulated Biological Processes in Serum/Cytokine-Treated Cells

Quantitative profiling of the nuclear and cytoplasmic fractions of the serum/cytokine-treated cells resulted in 715 proteins representing biological processes similar to the ones described for the serum-depleted cells. Correlated, however, with the addition of FBS, the aberrant increase in ribosome biogenesis, one of the most energetically expensive biological processes (104), was unique to the serum-supplemented cells. **Figures 8B** and **9C** depict these changes, and relevant findings are discussed below.

#### Mitochondrial Gene Expression and ECM Remodeling

Mitochondrial expression, oxidative phosphorylation and ECM remodeling processes were represented mostly by the same proteins as in the serum-depleted cells. Two mitochondrial protein hubs emerged, one comprising mitochondrial ribosome proteins matched to mitochondrial gene expression and translation, and the other to mitochondrial organization and OXPHOS. As most mitochondrial proteins are encoded by nuclear DNA and then translated on cytosolic ribosomes, the mitochondrial inner membrane (TIMM) and outer membrane (TOMM) translocase complexes that import proteins into mitochondria were correspondingly upregulated (105). An increased expression of collagens, integrins and ECM molecules that participate in cell adhesion and migration correlated with increased integrin signaling and cytoskeletal rearrangement (106). In addition, three noteworthy proteins, peroxidasin (PXDN) which is involved in ECM synthesis, alpha-2-macroglobulin (A2M) which is an inhibitor of proteases such as collagenase and of inflammatory cytokines, and disintegrin/metalloproteinase domain-containing protein 17 (ADAM17) which is responsible for the proteolytic release of cell surface proteins, were observed. The induction of TGFBI and TGFB1I1 adhesion and migration modulators was observable, and downstream effects of TGFβ signaling with roles in regulating actin cytoskeleton dynamics, cell migration and angiogenesis were manifested *via* the upregulated RHOA, ROCK1, SMAD2 and its coactivator SNW1 proteins.

#### Immune Response

The immune response clusters were less distinct in the serum/cytokine treated cells, most likely due to the dominating cell-cycle related processes induced by the addition of serum. Nevertheless, proteins involved in signal transduction pathways that link the cell membrane receptors to adhesion, cytoskeleton organization, transport and intracellular signaling could be associated with antiviral innate and interferon type I responses (NFKB1, TRAF3, PML, SP100, STAT1/2, ISG15/20, MX1/2, IFIT1, SAMHD1, HLA-A, HLA-C, TRIMs) (**Figure 9C**). Intermingled with cell cycle and nuclear import/export, notable was the higher level of interferon signaling that was evident from the activation of the interferon-induced proteins (IFIT1, ISG15, ILF3, MX1, MX2, TRIM22). The signal transducers STAT1 and STAT2 which mediate IFN-γ signaling were also present with increased spectral counts in the cells. In the same time, however, PARP14, an ADP-ribosyltransferase which had been proposed to have roles in suppressing pro-inflammatory IFN-γ/STAT1 macrophage activation, while also promoting anti-inflammatory activities *via* IL-4/STAT6 modulation (107), was upregulated, as well. HLA-C class I histocompatibility antigens were complemented here by HLA-A antigens (A-24α and A-31α) and by a downregulated HLA-DOA, which is a modulator of HLA class II histocompatibility antigen presentation pathways (42). Its expression is inhibited by IL-10 (12).

The multifaceted NFKB signaling enterprise was also better represented in the serum/cytokine-treated cells, as evidenced by the upregulation of TRAF3 (TNF receptor associated factor)-a protein involved in NFKB activation, of NFKB1 (p105 or p50)-a protein that promotes the transcription of inflammatory genes, and of its inhibitor (NFKBIE)-a protein that prevents NFKB from translocating into the nucleus and activating transcription. The common heterodimeric complex partner of NFKB1, RELA or p65, was slightly upregulated in the cytoplasmic fractions, without passing though the 2-fold change criteria. NFKB1 is activated in response to a variety of stimuli, and can act as both a transcriptional activator or repressor as a function of the type of complexes that it forms. Previous reports suggested that the ECM laminin or fibronectin, along with certain phagocytic processes, can also trigger NFKB activation *via* integrin-mediated signaling (108). However, the NFKB1/RELA complex could not be detected in the nucleus, suggesting that the pathway was not fully activated. Nonetheless, REL (or c-REL), which is one of the transcription factors for HLA-A (12), could be observed with increased counts in the nuclear fraction of cells. Interesting to note was also that an NFKB-activating protein (NKAP) that induces activation upon inflammatory cytokine stimulation, was depleted in the nuclear fractions of both G1 and S cytokine-treated cells.

Despite the observed alterations in these pathways, the production of classical pro-inflammatory cytokines did not occur. Only one TNF protein, C1QTNF1 (Complement C1q tumor necrosis factor-related protein 1), was detected with increased counts. Notable was also the upregulation of NLRC4 (NLR family CARD domain-containing protein 4)-a component of the inflammasome, of ANKRD17 (Ankyrin repeat domain 17)-a protein that mediates innate antibacterial pathways *via* NOD1/NOD2 pattern recognition responses, and of the multifunctional C1q complement binding protein C1QBP which has roles in driving ribosome biogenesis, protein synthesis in mitochondria, and assembly of the mitoribosome (42).

Altogether, while elements of interferon and NFKB signaling were observed in the serum-depleted cells, the molecular differences brought about by the addition of serum appeared to have contributed to augmenting these processes in the serum/cytokine-treated cells. Either canonical NFKB signaling activated by TLR stimulation leading to the expression of certain uncharacteristic IFN signaling and mediator proteins, or autocrine signaling through cytokine receptors followed by activation, may have resulted ultimately in a spectrum of microglia responses. Inhibitory effects induced by the anti-inflammatory cytokines, lack of a strong enough signaling input to meet the necessary thresholds, negative feedback loops or cross-talk with other signaling pathways, appeared to have antagonized ultimately a full activation process.

#### Cell Cycle and Ribosome Biogenesis

Upregulated cell cycle processes were sustained by proteins indicative of G1/S transition and DNA replication initiation (MCM licensing factors, PRIM1), mitotic prometaphase proteins, components of the nucleopore complex, and RNA polymerase transcription initiation and termination proteins (POLR2s, GTFs 2F/2E/2H, ERCC3). Cyclin H (CCNH)-an activator of CDK1/2/4/6, and cyclin Y (CCNY)-a positive regulator of Wnt signaling pathway, were both present with increased counts in the nuclear fractions of cytokine treated cells. Many of the above proteins have roles in both positive and negative regulation of gene expression, and the presence of negative regulators of cell cycle (PML, WEE2, E2F7, CTNNB1, PPP2R5B, ILK, THBS1, EZH2), of regulators of TP53 activity (PML, TP53RK), and of apoptotic proteins (AIFM1, MST4, STK24, MTCH1, SH3GLB1), underscored complex functional, and possibly opposite trends in the dataset. Some cell cycle proteins were also representative of immune response and antiviral processes. For example, SAMHD1 which is a regulator of DNA end-resection at stalled replication forks is also involved in antiviral defense responses, and the interferon-induced antivirals GTPases MX1 and MX2 have roles in cell death and cell-cycle progression, respectively.

Ribosome biogenesis is considered a hallmark of cell growth and division. The nuclear machinery that mediates DNA transcription and ribosome synthesis prior to mRNA translation was heavily represented by general transcription factors (GTFs), RNA polymerase II subunits (POLR2s), nucleolar ribosome biogenesis proteins (UTPs), nucleolar regulator of RNA polymerase I (NOLC1), and nucleolar proteins involved in pre-rRNA and rRNA processing (RRPs). The cytoplasmic translation initiation factors (EIF1AX, EIF2B2, EIF2D) complemented this group. It is known that anabolic processes such as ribosome biogenesis and protein synthesis are driven by mTORC signaling in growing cells (103). It has also been shown that mTORC1 negatively regulates autophagy, and positively affects oxidative metabolism through changes in mitochondrial DNA content (109). Along these lines of evidence, the activation of two mediators of the mTOR pathway, RPTOR and LAMTOR1, which upon sensing nutrients activate mTORC1 and promote cell growth, was observed. In response to IL-4 stimulation, LAMTOR1, a component of the Ragulator complex, was also shown to be essential for suppressing inflammatory innate immune responses and polarizing macrophages towards an M2 phenotype (110). As shown, however, by the cell-cycle clusters that were downregulated in the cells and that were indicative overall of impaired cell proliferation (see below), the overexpression of ribosome biogenesis proteins appeared to be rather related to mitochondrial expression, ECM and migratory processes than to cell cycle progression.

### Downregulated Biological Processes in Serum/Cytokine-Treated Cells

Similar to the serum-depleted cells, the serum/cytokine-treated cells showed a downregulation of processes involved in gene expression, transcription regulation, cell cycle, DNA repair, vesicle transport/intracellular trafficking, and cytoskeleton organization (**Figures 8B** and **9D**). Representative proteins included mainly transcription initiation factor TFIID subunits (TAF1), general transcription factors (GTFs), Mediator subunits of RNA polymerase II transcription, RNA polymerase subunits (mainly I), regulators and activators of transcription (NFIB-Nuclear factor IB, HMGB2, RPS6KA5, BCLAF1, and THRAP 3), and replication factors (RFC1/3). These proteins were complemented by components of the E3 ubiquitin-protein ligase complexes (APC, BCR, SCF) that mediate the ubiquitination and degradation of target proteins (cyclins, CDKs), thereby controlling cell cycle G1/S transition and exit from mitosis. The downregulation of cyclins A2 (CCNA2) and B2 (CCNB1), as well as of cyclin T2 (CCNT2)-a subunit of the positive elongation transcription factor-confirmed the suppressed status of these cells. Further, the identification with lower nuclear counts of CDT1, a DNA replication licensing factor with roles in generating the pre-replication complex, was indicative of its possible degradation and impaired proliferation. Moreover, PLK1 (Polo-like kinase), a Ser/Thr kinase which increases in expression level during mitosis, was downregulated in the nuclear fractions of cytokine-treated cells, also suggesting repressed proliferation.

MHCII class antigen presentation was affected by the downregulation of HLA-DOA, vesicle-mediated protein transport, and microtubule-dependent and Rho-mediated signaling. Downregulated proteins involved in adhesion, cytoskeletal re-arrangement, motility/migration (ENG, PIK3CB), phagocytosis (ELMO2, DOCK1), and intracellular trafficking and endocytosis (RAB13, RAB5A, DNM1, DNM3, kinesins) are anticipated to impact processes related to chemotaxis and phagocytosis. On a global level, changes in the activity of proteins with roles in the transcription regulation and activation of inflammatory genes (RPS6KA5), in the activation of signaling cascades involved in cell proliferation/survival and motility (PIK3CB), in proliferation/migration and PRR signaling (HMGB2), TGFβ signaling (ENG and SMAD4), and cell-cell recognition and adhesion processes (VCAM1), are expected to affect innate immunity and inflammatory responses. Overall, the results confirm the role of TGFβ in cell cycle arrest, cytokinesis, suppression of immune responses, and the development of a cancer-supportive microglia phenotype. The downregulation of CD33 (Myeloid cell surface antigen) that inhibits immune cell activation (57), and that requires PI3K to exert its suppressive effects, suggests the presence of both immune-activating and deactivating trends. Likewise, it was interesting to note the downregulation of TLK2, a Ser/Thr kinase with roles in chromatin assembly and transcription, that is also a negative regulator of autophagy induced by amino acid starvation (42).

### Tumorigenic Phenotype of Microglia Activated by the Anti-Inflammatory Cytokines

**Figure 11:**
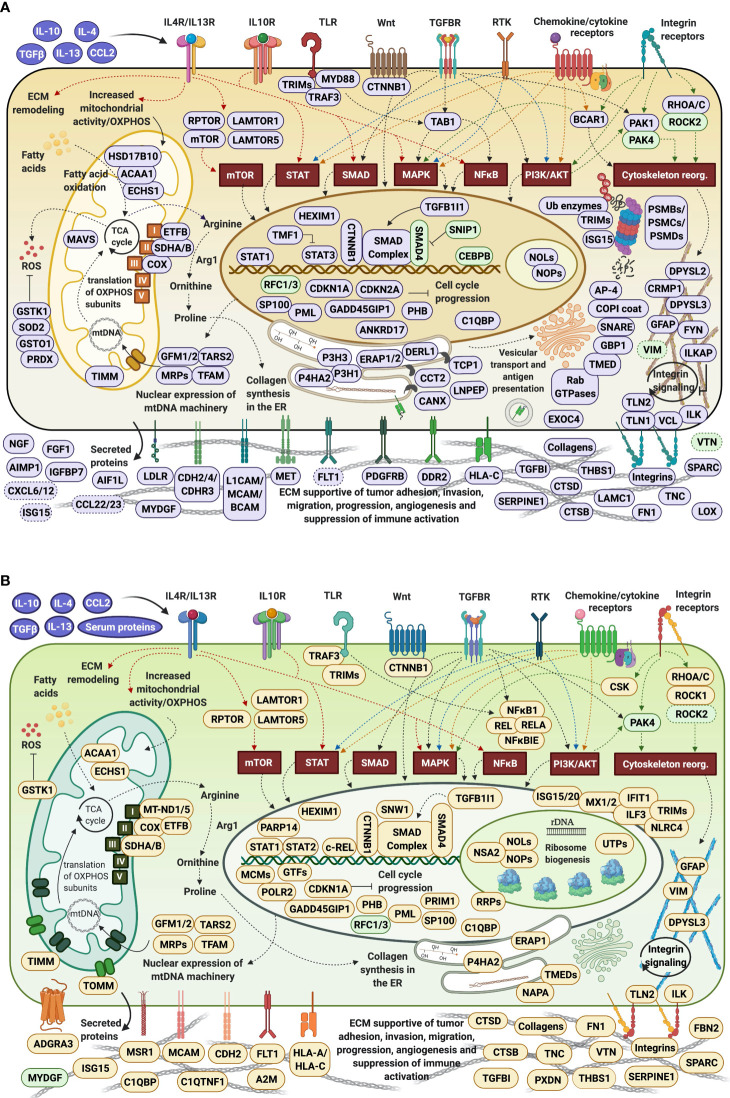
Protein landscape supportive of pro-tumorigenic HMC3 cells. **(A)** Cytokine-treated/serum-depleted cells. **(B)** Cytokine/serum-treated cells. Proteins were placed in the cellular compartment in which they exert their main function. Proteins highlighted in purple or amber displayed increased spectral counts, while proteins highlighted in green displayed decreased spectral counts in the cytokine-treated cells. Arrows indicate established relationships between receptors and their activated pathways.

The biological processes that were altered in the cytokine-treated HMC3 cells uncovered numerous mechanisms by which the microglia could support tumor progression in the brain. These processes were supported by (a) dynamic changes in the composition of the ECM that foster tumor attachment and invasion, (b) increased expression of proteins that facilitate angiogenesis, (c) secretion of cytokines and growth factors that promote tumor growth, and (d) expression of proteins that support the recruitment of cells that suppress immune activation. **Figure 11** summarizes the findings of this work by illustrating the protein landscape that could sustain and drive pro-tumorigenic functions in the microglia cells.

The key determinants of the tumor-supportive microglia phenotype were the proteins of the MAPK, STAT, TGFβ, NFKB, PI3K, mTOR and integrin signaling pathways. Remodeling of the ECM matrix emerged as a pivotal outcome of the altered signaling networks, as evidenced by the increased production of collagens, collagen stiffening enzymes, and proteases and protease inhibitors that alter the intercellular matrix to ultimately support tumor adhesion and invasion. Together with collagen, additional matrix proteins such as fibronectin, vitronectin and tenascin facilitate the entrapment of growth factors and cytokines responsible for the transmission of signals to- and within the cells to trigger the expression of genes that control protein synthesis, survival, migration, and angiogenic processes in cancer progression. Complementary functional roles point to competencies for macrophage recruitment to the site of tumorigenesis and M2 polarization (e.g., serpine 1) (111), as well as suppression of immune activation by limiting the infiltration of T-lymphocytes (e.g., tenascin) (112). In response, collagen sensors in cancer cells (i.e., DDRs) can initiate metastatic migratory and invasion programs (72, 113). Finally, integrin-mediated signaling has diverse roles in supporting tumor progression *via* processes that range from angiogenesis to adhesion, survival, motility and microglial recruitment (73, 74, 106). By bridging the ECM with the intracellular environment, integrin receptors have the ability to cause changes to the actin cytoskeleton (76) to promote the migration of cells in the surrounding environment.

The immunosuppressive and tumorigenic characteristics of microglia were further augmented by the secretion of growth factors such as NGF which can counter inflammatory responses (114), MYDGF which increases viability of cancer cells (115), FGF1 which supports angiogenesis and tumor growth and invasion, and of cytokines CCL22 and CXCL12 which attract pro-tumor immunosuppressive cells (7). Through an increased expression of MHC class I molecules, the microglia have demonstrated diminished immune activation against tumor-cells and induced expression of angiogenic factors and cytokines (116). Moreover, the anti-tumor inflammatory activation of microglia was directly affected by reduced cell proliferation that was linked to TGFβ and IL-10 signaling mechanisms. Altogether, the upregulated biological processes in HMC3 suggest that the microglia cells favor the formation of a tumor-supportive niche when they are subjected to an anti-inflammatory microenvironment *in-vitro*.

## Conclusions

Proteomic analysis of HMC3 cells treated with anti-inflammatory cytokines exposed a vast array of altered biological and immunological functions. These processes distinguished themselves more clearly in the serum-depleted cells. Altogether, the combined effect of IL-4, IL-13, IL-10, TGFβ and CCL2 resulted in the induction of a spectrum of M2a/c microglia phenotypes with capabilities for inducing matrix deposition, ECM-remodeling and angiogenic processes, and overall, a tumor-supportive niche that facilitates growth and invasion. Mitochondrial gene expression and respiration were highly upregulated processes, resulting in altered arginine metabolism and collagen synthesis. As evidenced by the upregulation of certain receptors, cytokines and endocytic activities, the stimulated cells were inclined to rather exhibit an immunosuppressive response, even though pro-inflammatory defense elements were observable. MAPK signaling was central to all cell communication pathways, and STAT components were involved in mediating several biological outcomes. The negative impact of TGFβ on cell cycle progression was clearly manifested through the altered activity of proteins involved in gene expression, G1/S and G2/M transition, cell division, and apoptosis. NFKB signaling manifested itself mainly in the serum/cytokine-treated cells but it did not emerge as being fully activated. It was accompanied, however, by several proteins representative of interferon signaling. Altogether, the study underscores the complex network of interactions that are activated in stimulated fetal microglia cells, and demonstrates the benefits of proteomic profiling for gaining insights into the many, still unknown, environment-contingent mechanisms that drive cancer progression. The immunosuppressive landscape enabled by co-operative microglia points to possible novel therapeutic targets that could guide future drug discovery efforts.

## Data Availability Statement

The mass spectrometry raw files were deposited to the ProteomeXchange Consortium via the PRIDE partner repository with the following dataset identifiers: PXD023163 and PXD023166.

## Author Contributions

SA performed the experiments. SA and IL analyzed the data and wrote the manuscript. IL coordinated the work. All authors contributed to the article and approved the submitted version.

## Funding

This work was supported by an award from the National Institute of General Medical Sciences (Grant No. 1R01GM121920) to IL.

## Conflict of Interest

The authors declare that the research was conducted in the absence of any commercial or financial relationships that could be construed as a potential conflict of interest.

## Publisher’s Note

All claims expressed in this article are solely those of the authors and do not necessarily represent those of their affiliated organizations, or those of the publisher, the editors and the reviewers. Any product that may be evaluated in this article, or claim that may be made by its manufacturer, is not guaranteed or endorsed by the publisher.
