# Supplementary material for: Systems-Level Proteomics Evaluation of Microglia Response to Tumor-Supportive Anti-Inflammatory Cytokines
Source: Front Immunol. 2021 Sep 9;12:646043. doi: 10.3389/fimmu.2021.646043 (PMC8458581; doi:10.3389/fimmu.2021.646043)

Supplemental File 1

PRM validation of changes in protein detection and expression

Systems-Level Proteomics Evaluation of Microglia Response to Tumor-Supportive Anti-Inflammatory Cytokines

Frontiers in Immunology 2021, doi: 10.3389/fimmu.2021.646043

Category 1

Mitochondrial/Redox proteins

Peroxiredoxin-5, mitochondrial (PRDX5)

ETDLLLDDSLVSIFGNR, Charge 2, m/z 953.99432

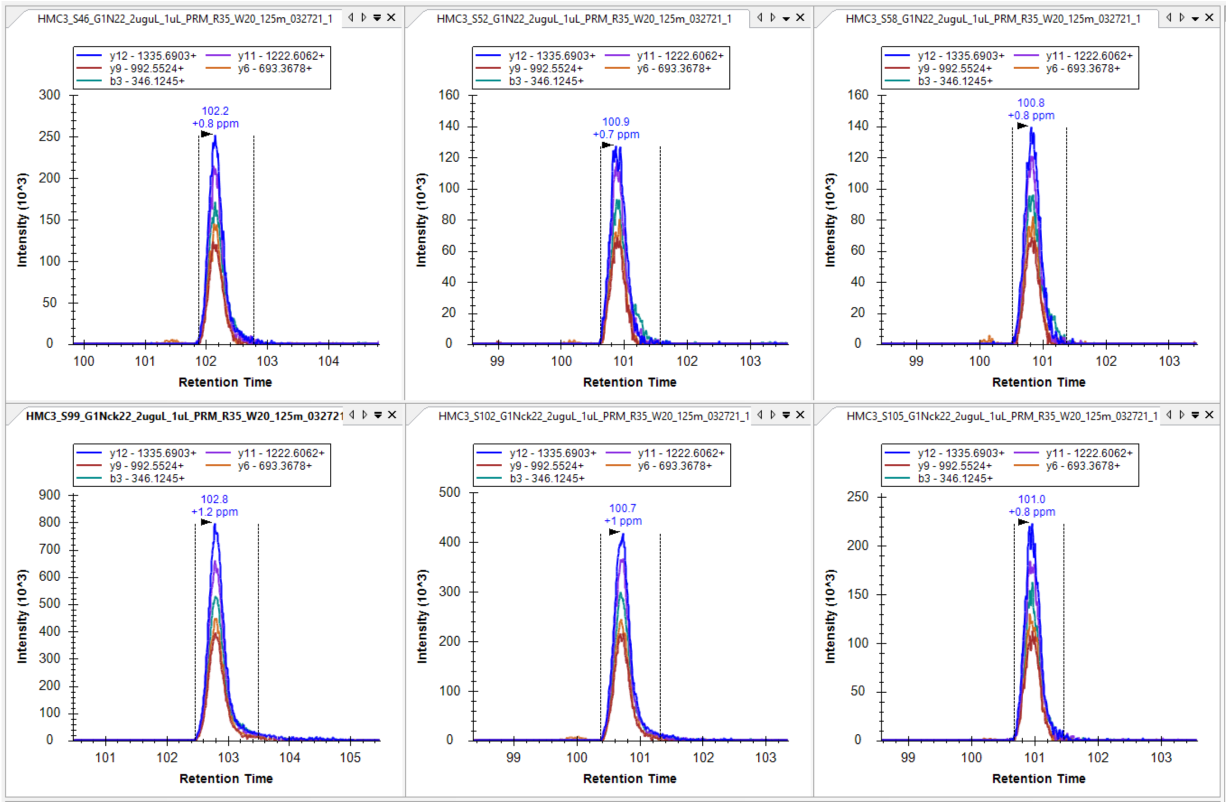

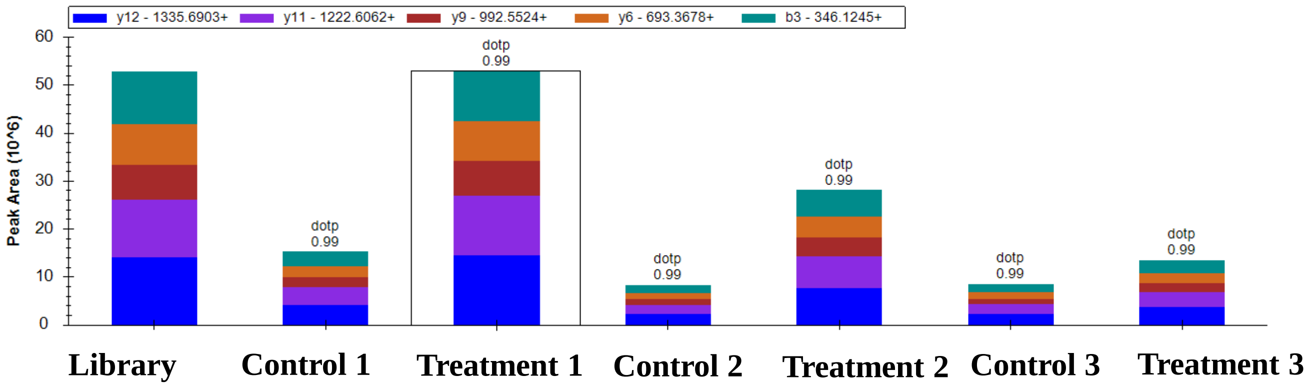

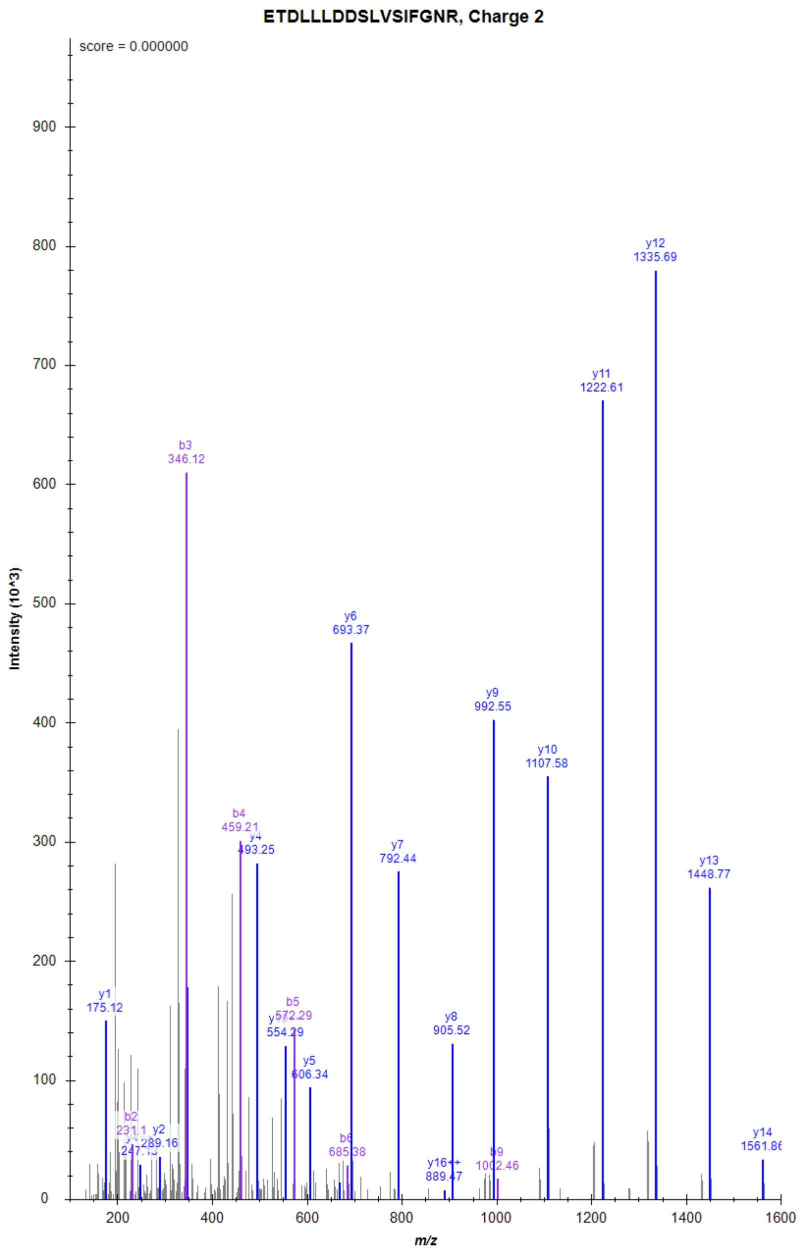

Peroxiredoxin-5, mitochondrial (PRDX5)

VGDAIPAVEVFEGEPGNK, Charge 2, m/z 914.45776

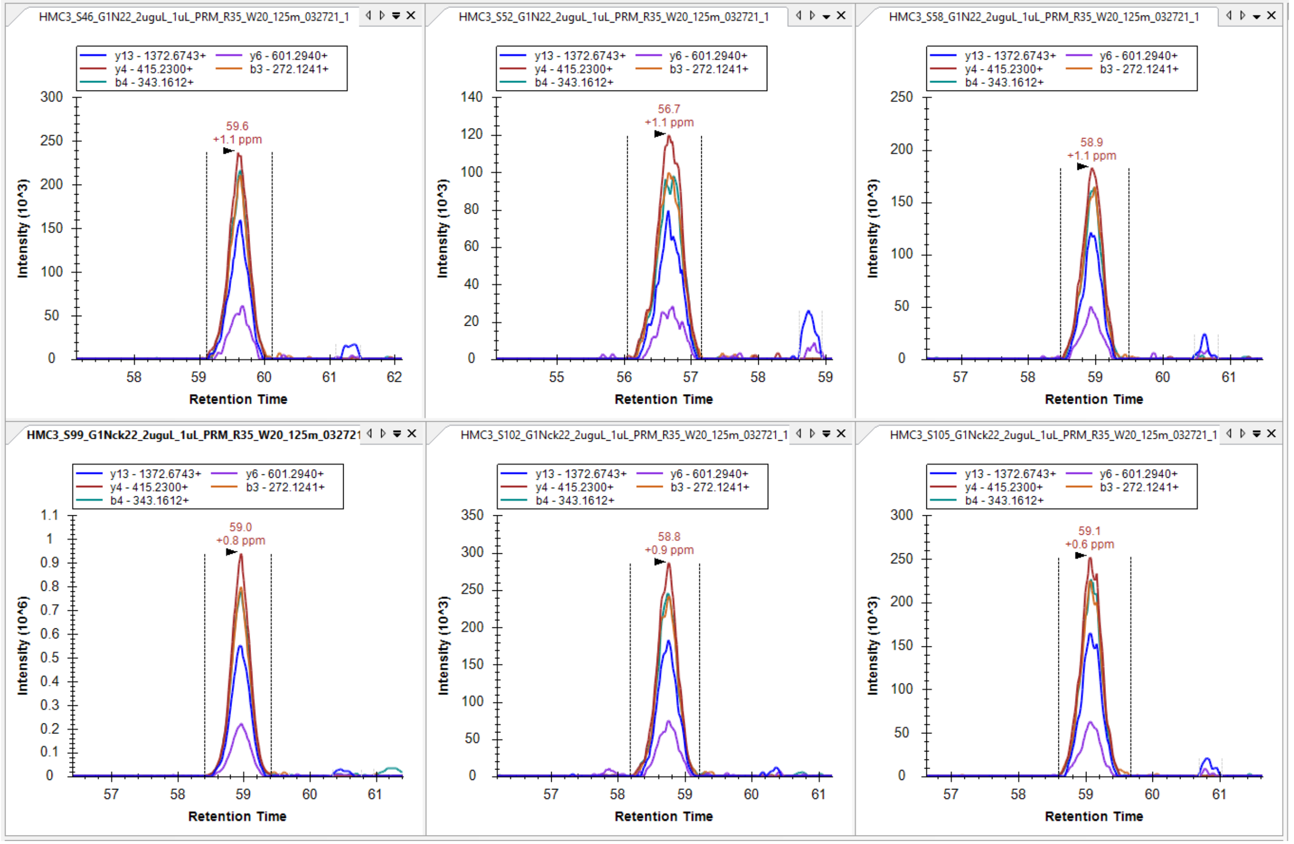

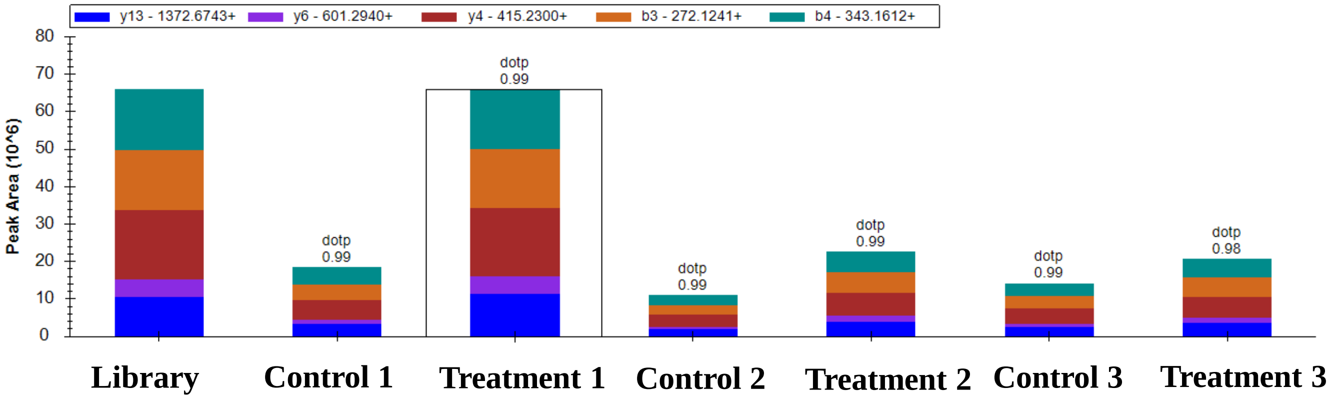

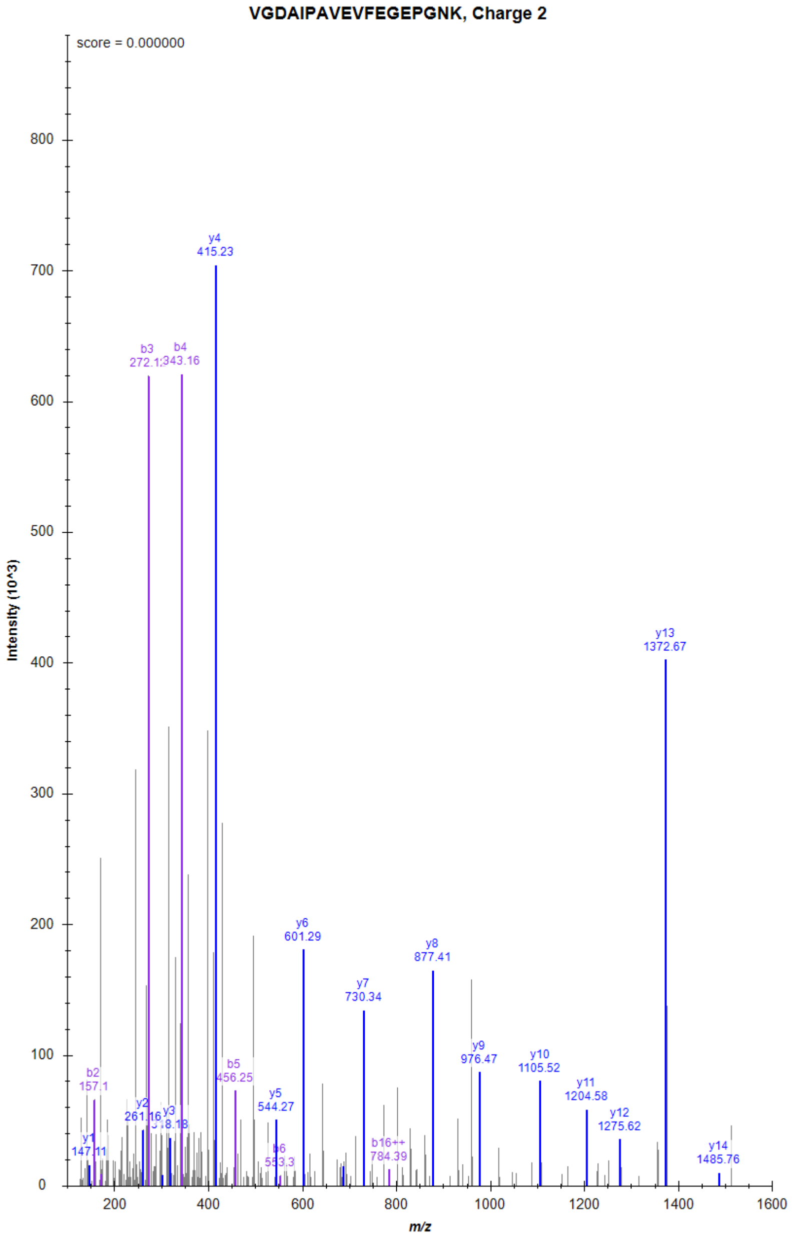

Thioredoxin-dependent peroxide reductase, mitochondrial (PRDX3)

DYGVLLEGSGLALR, Charge 2, m/z 731.89948

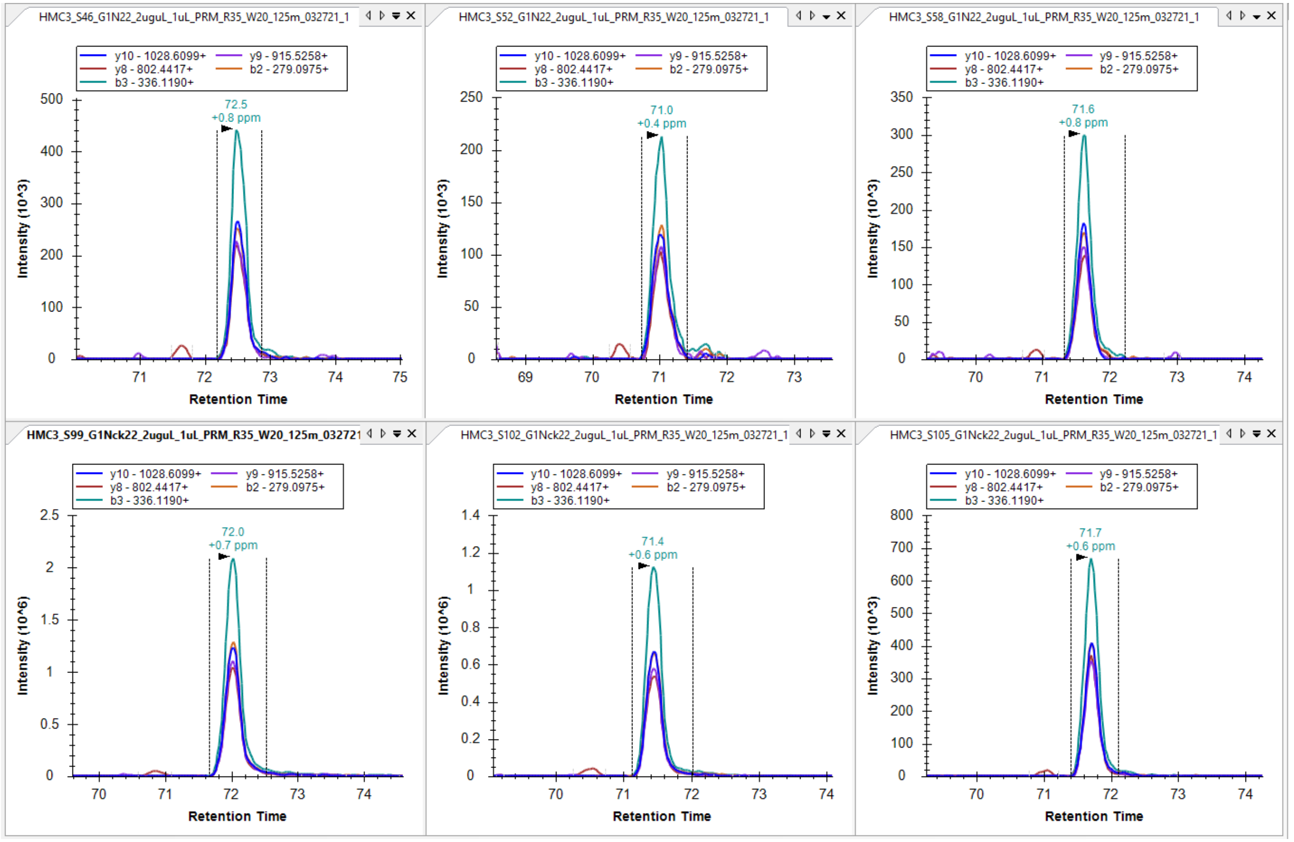

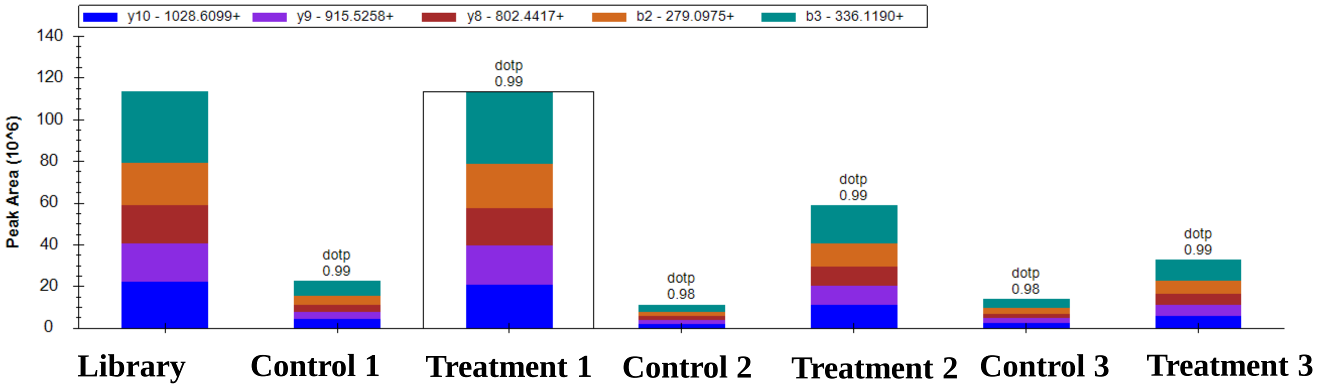

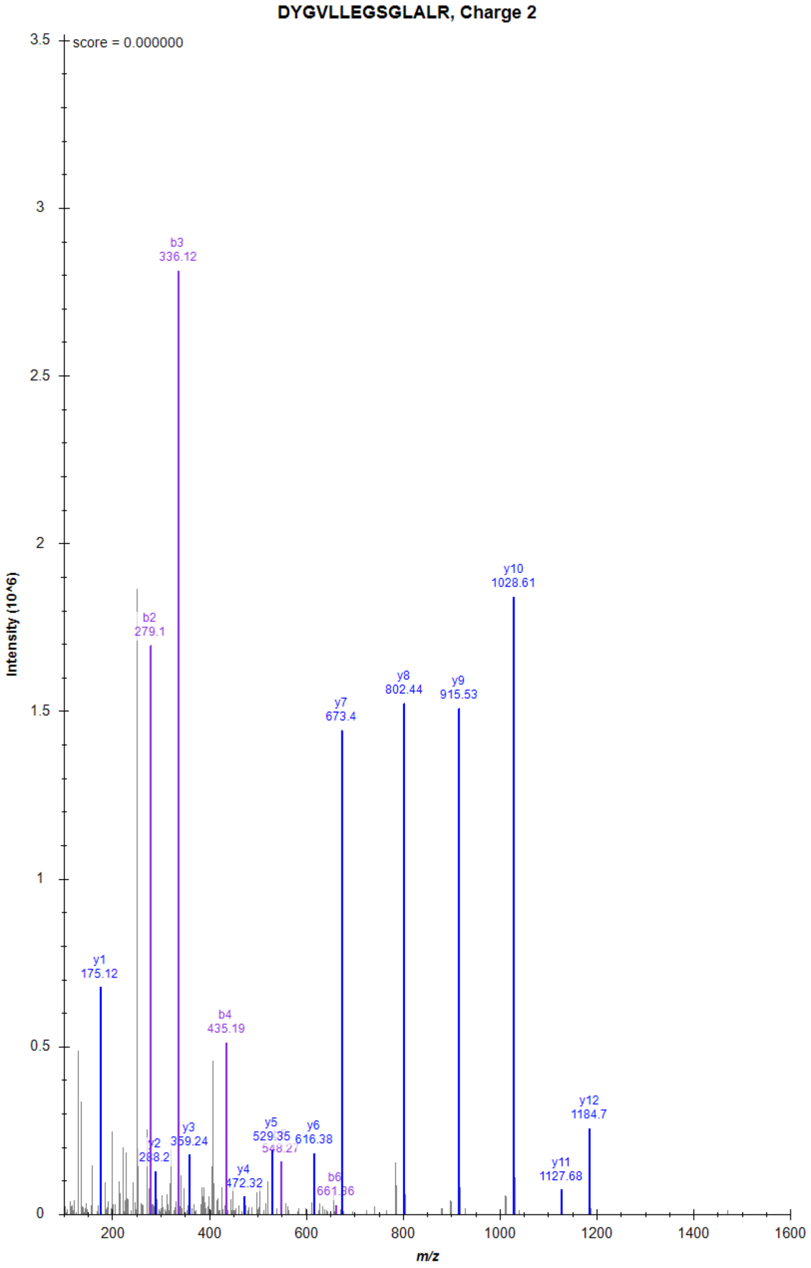

Thioredoxin-dependent peroxide reductase, mitochondrial (PRDX3)

HLSVNDLPVGR, Charge 2, m/z 603.83228

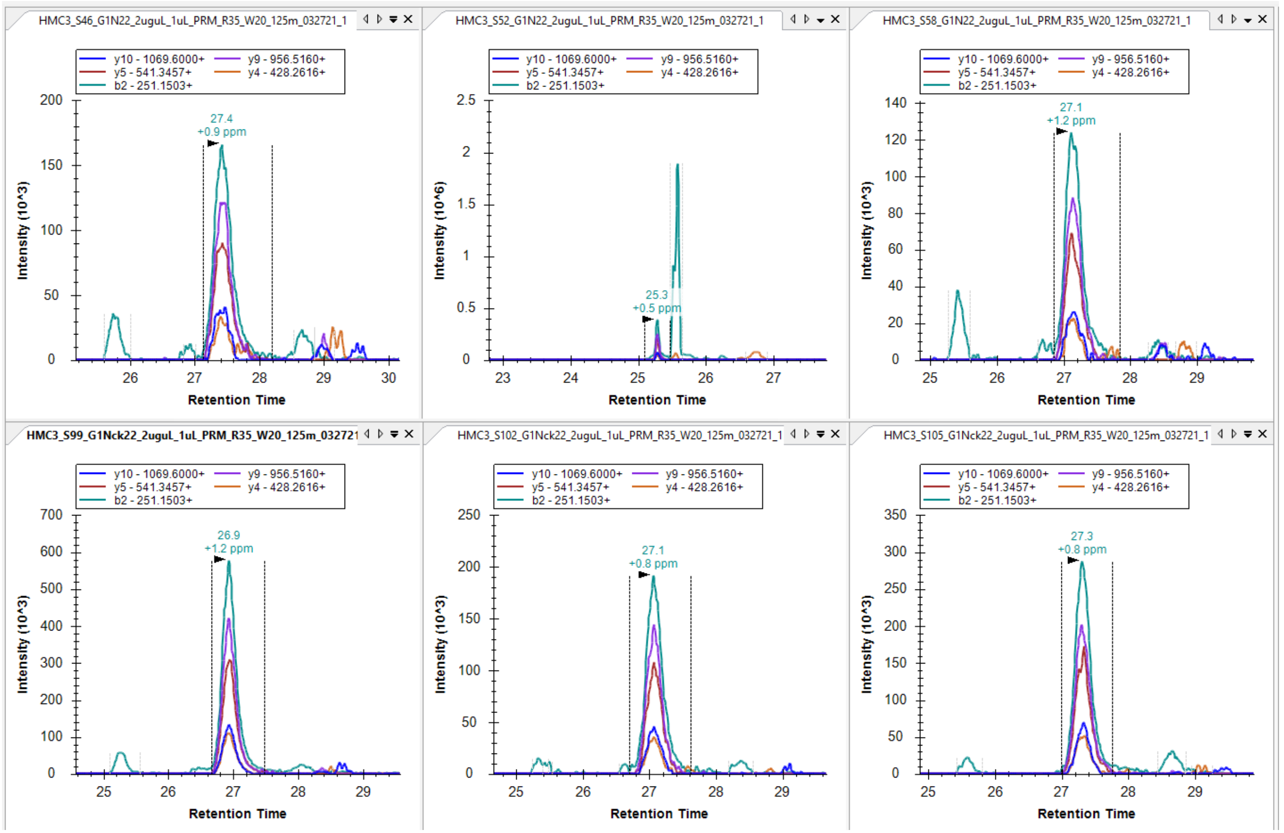

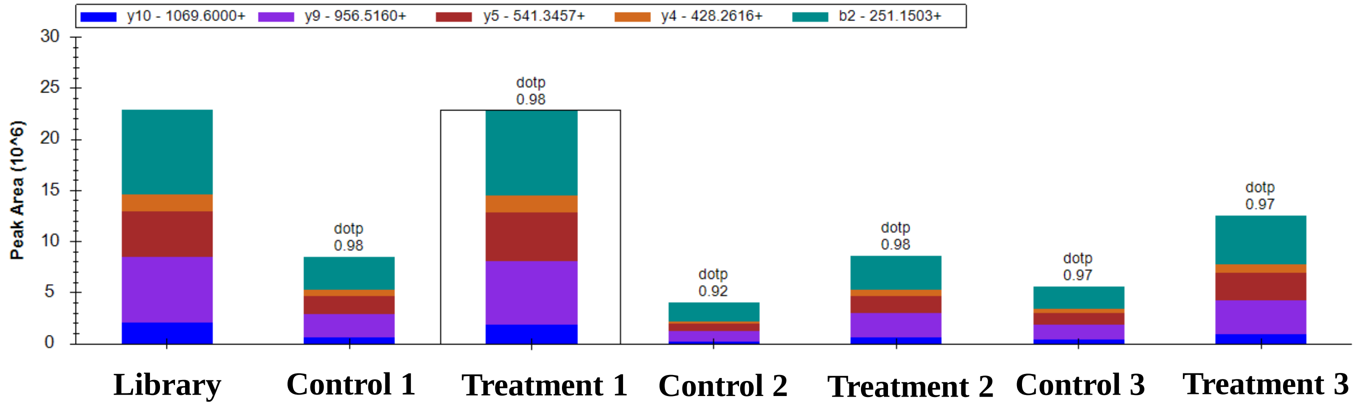

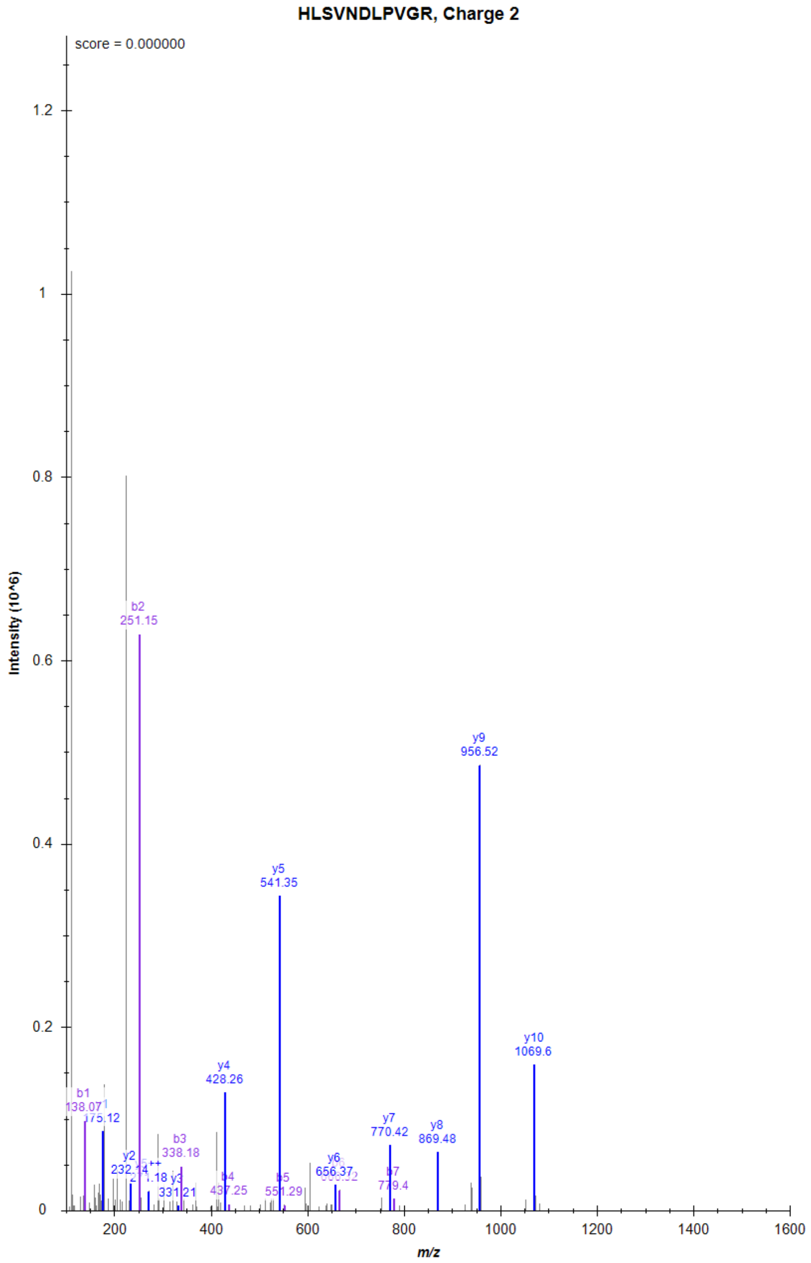

Prolyl 4-hydroxylase subunit alpha-2 (P4HA2)

TAELLQVANYGVGGQYEPHFDFSR, Charge 3, m/z 900.10327

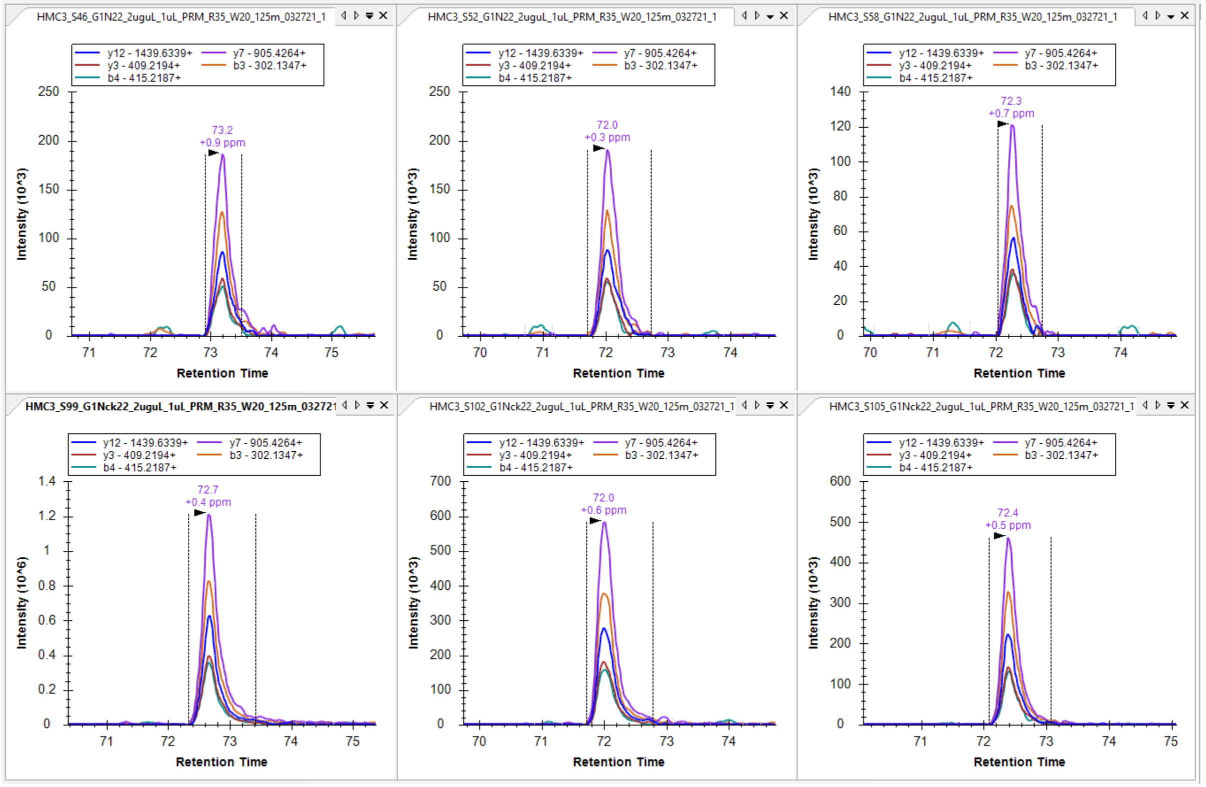

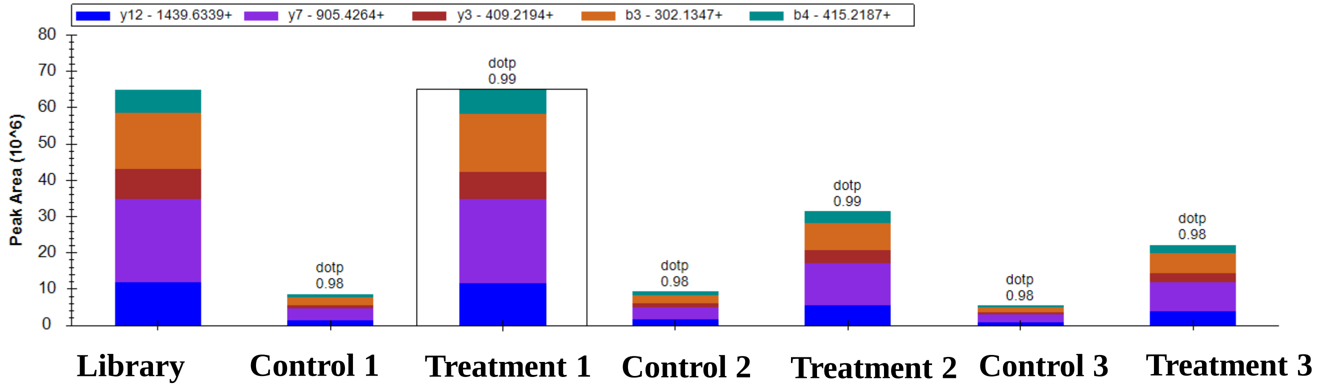

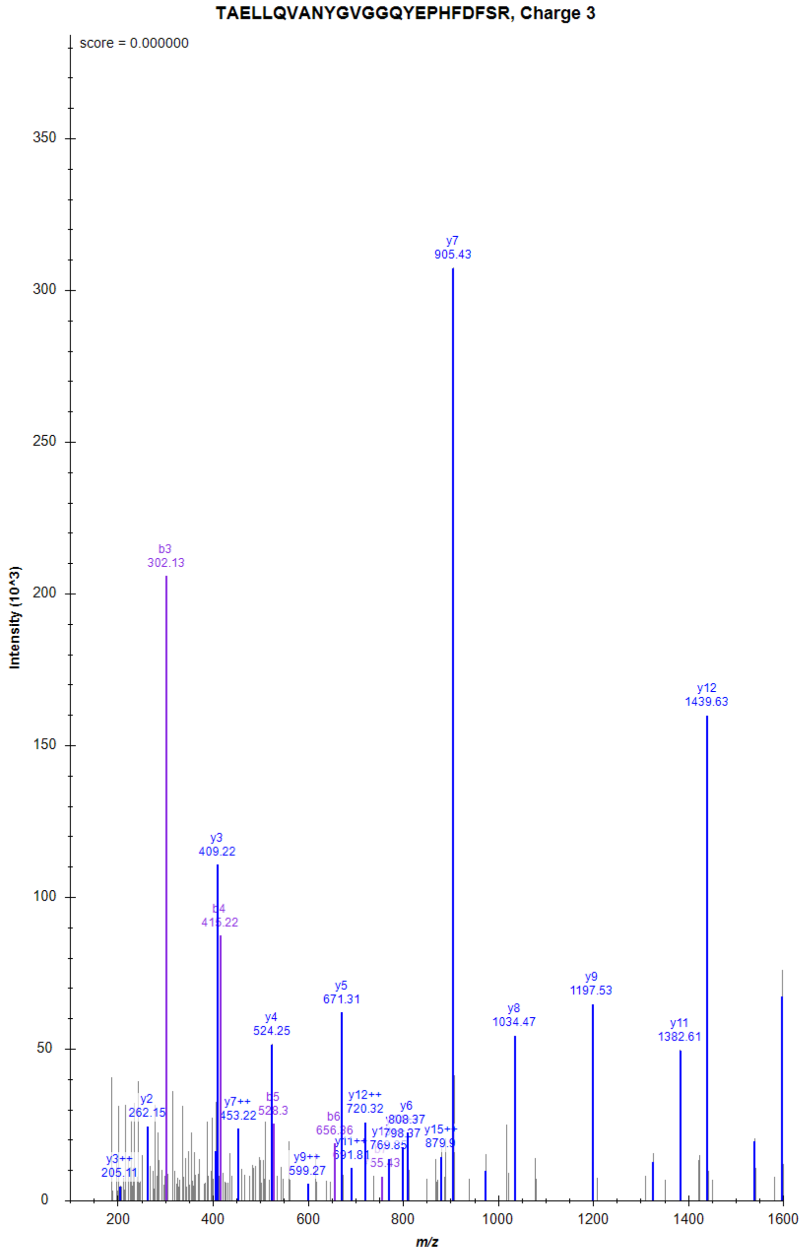

Prolyl 4-hydroxylase subunit alpha-2 (P4HA2)

SQVLDYLSYAVFQLGDLHR, Charge 3, m/z 742.05157

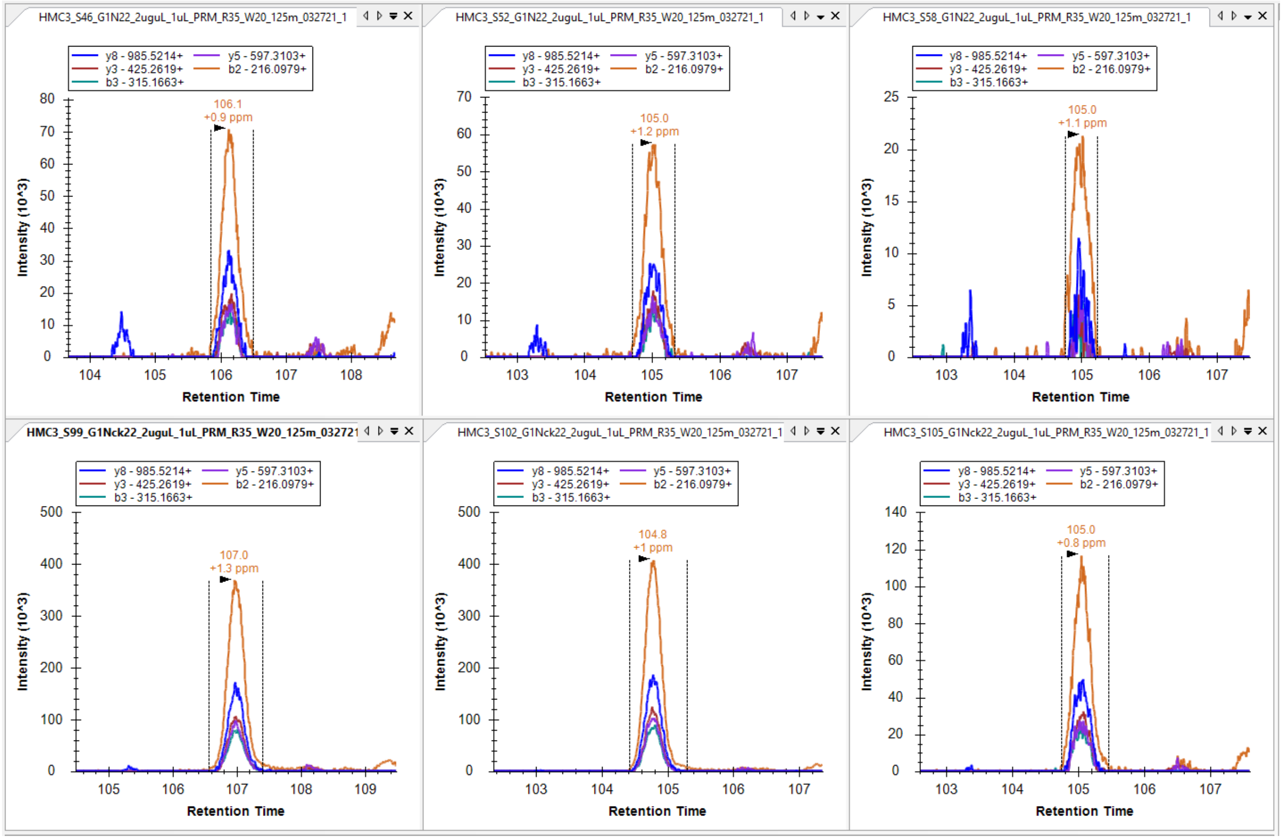

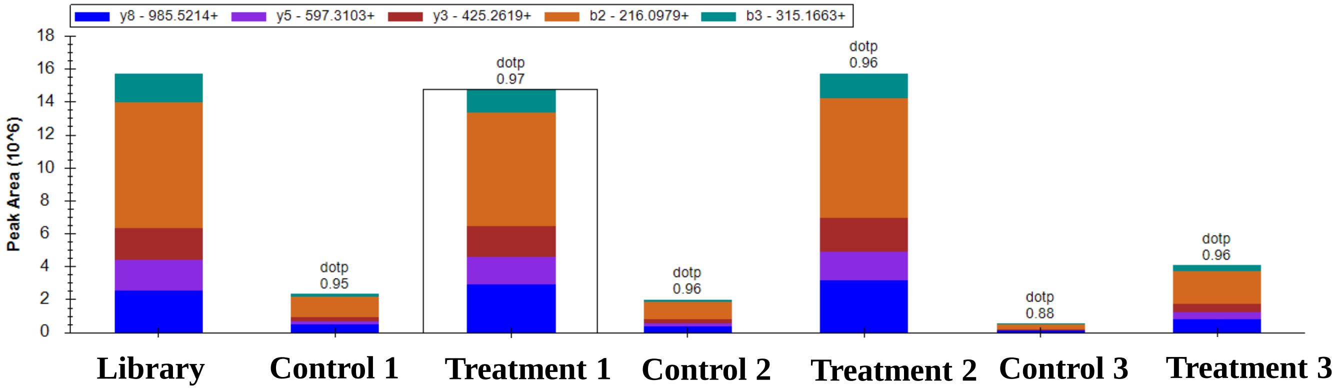

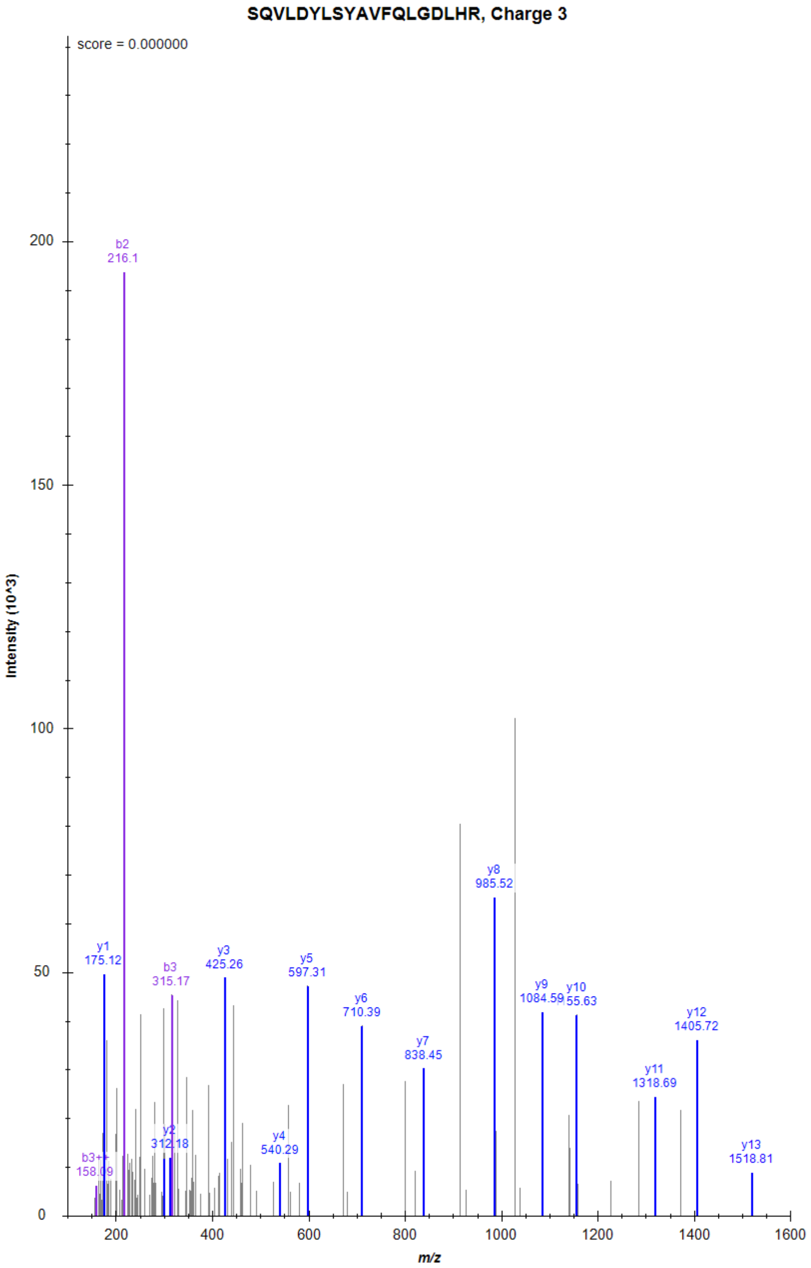

39S ribosomal protein L41, mitochondrial (MRPL41)

EMVPEFVVPDLTGFK, Charge 2, m/z 854.43652

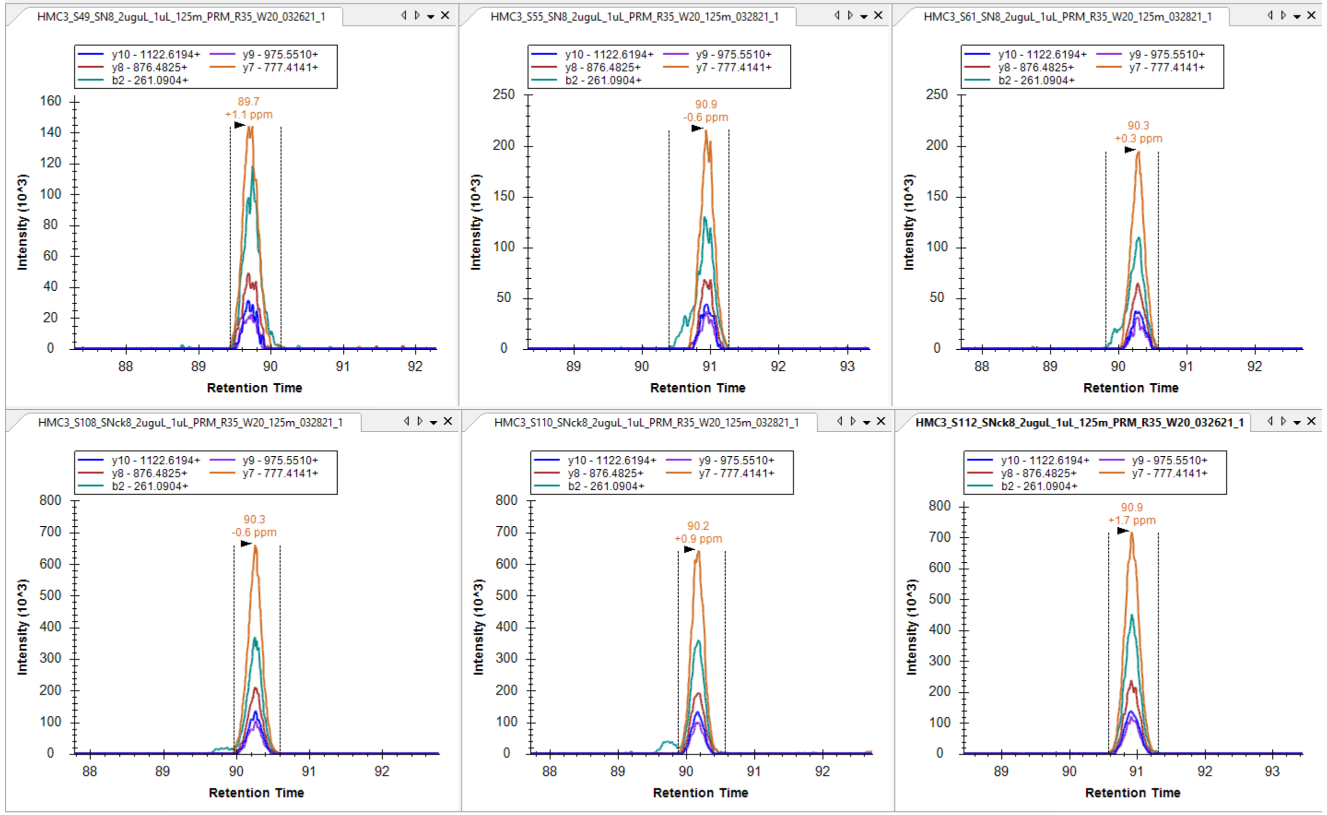

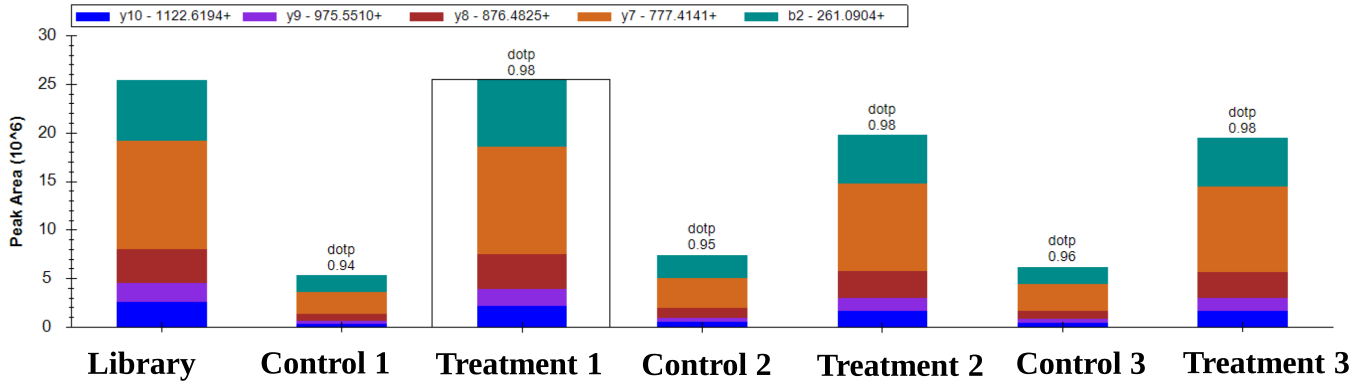

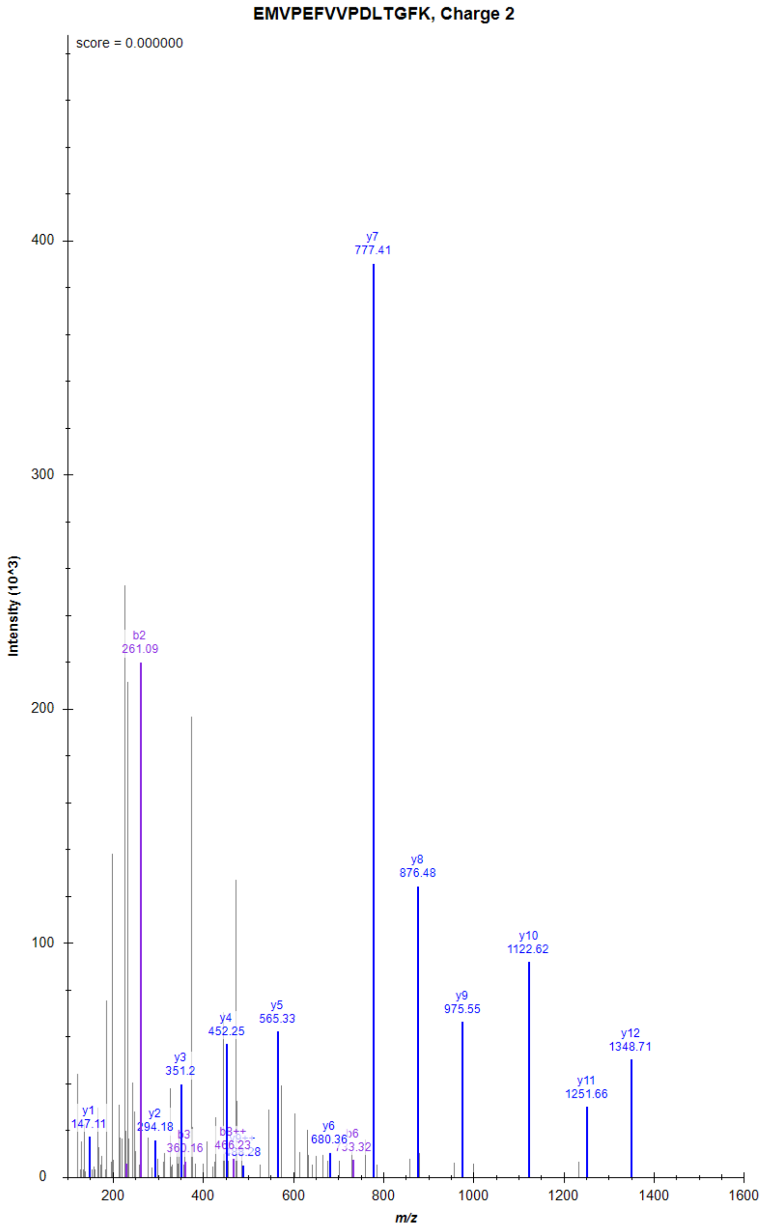

39S ribosomal protein L47, mitochondrial (MRPL47)

NMLLTLEQEAK, Charge 2, m/z 645.341

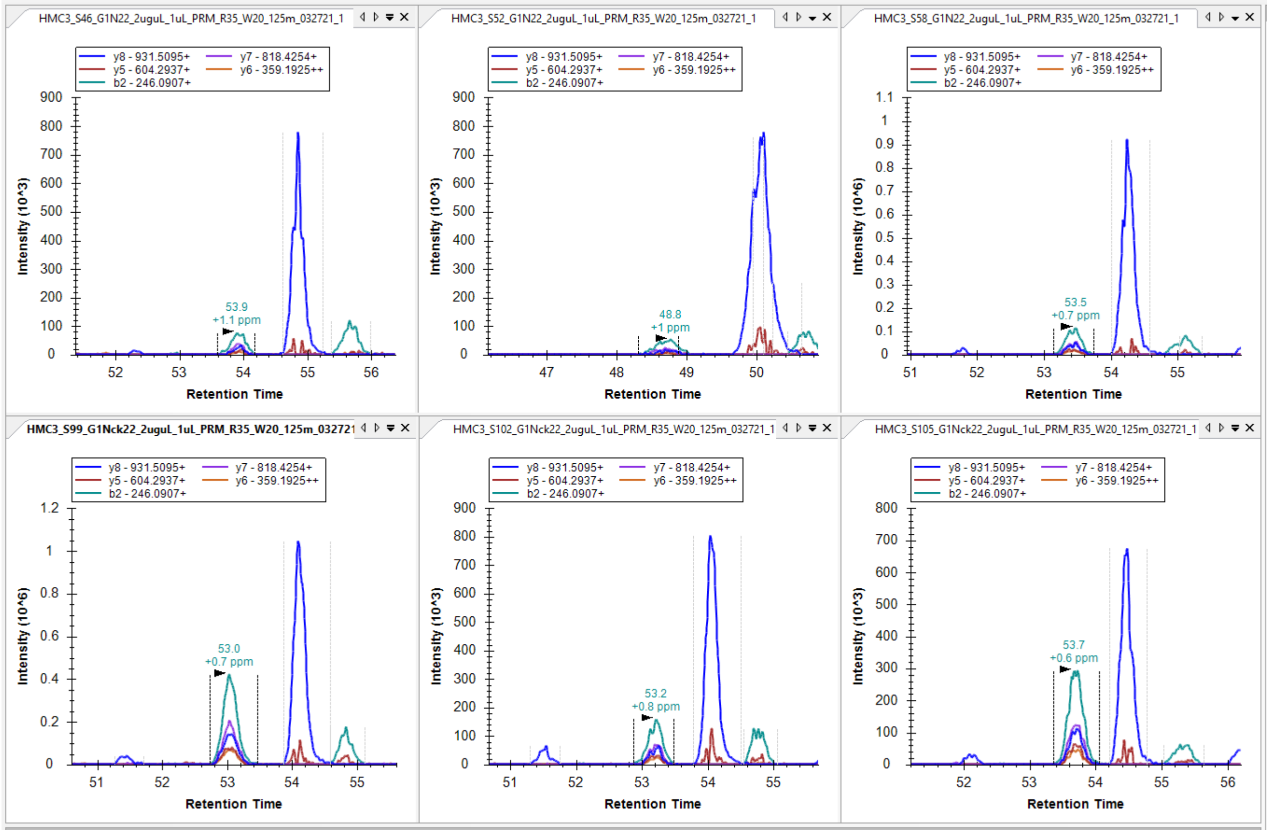

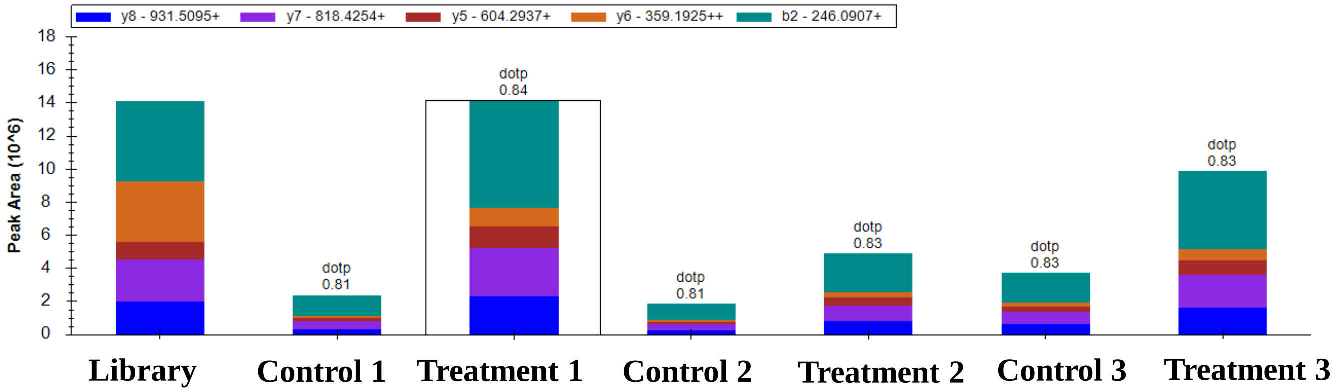

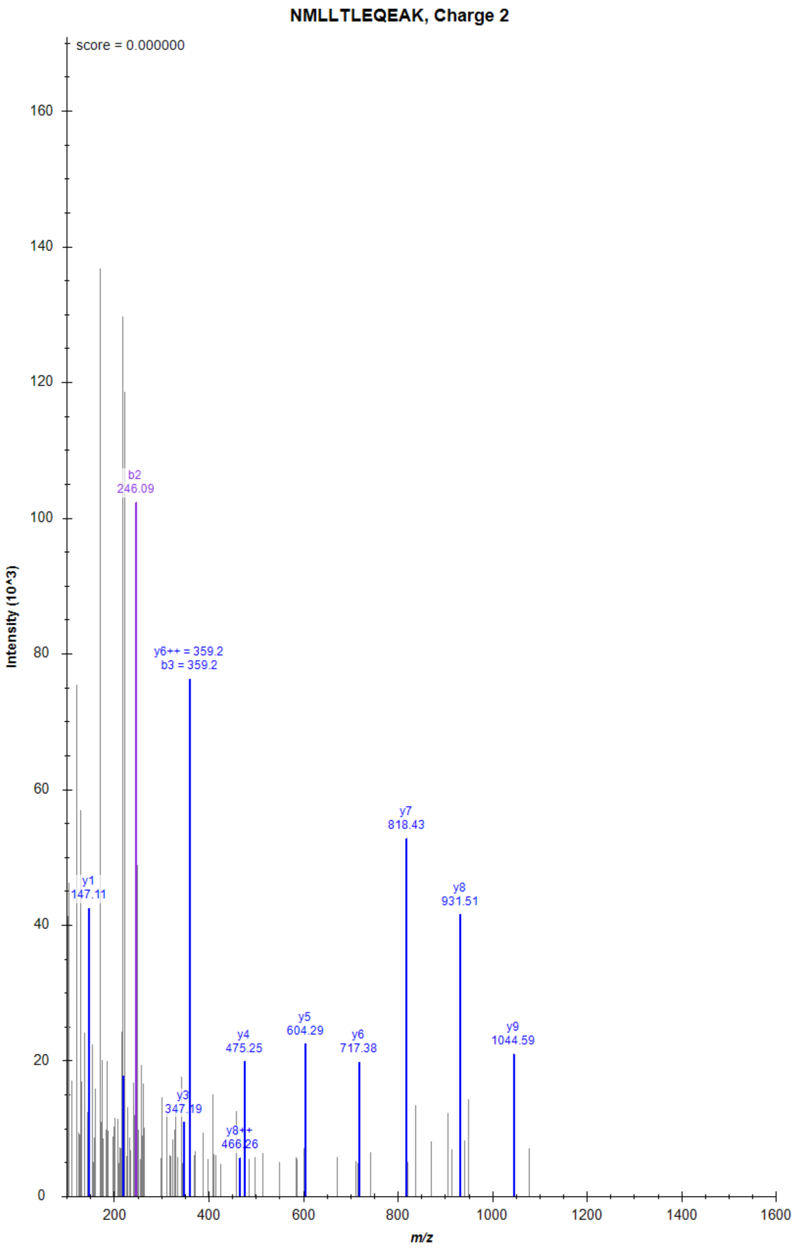

Glutathione S-transferase kappa 1 (GSTK1)

YGAFGLPITVAHVDGQTHMLFGSDR, Charge 3, m/z 897.1084

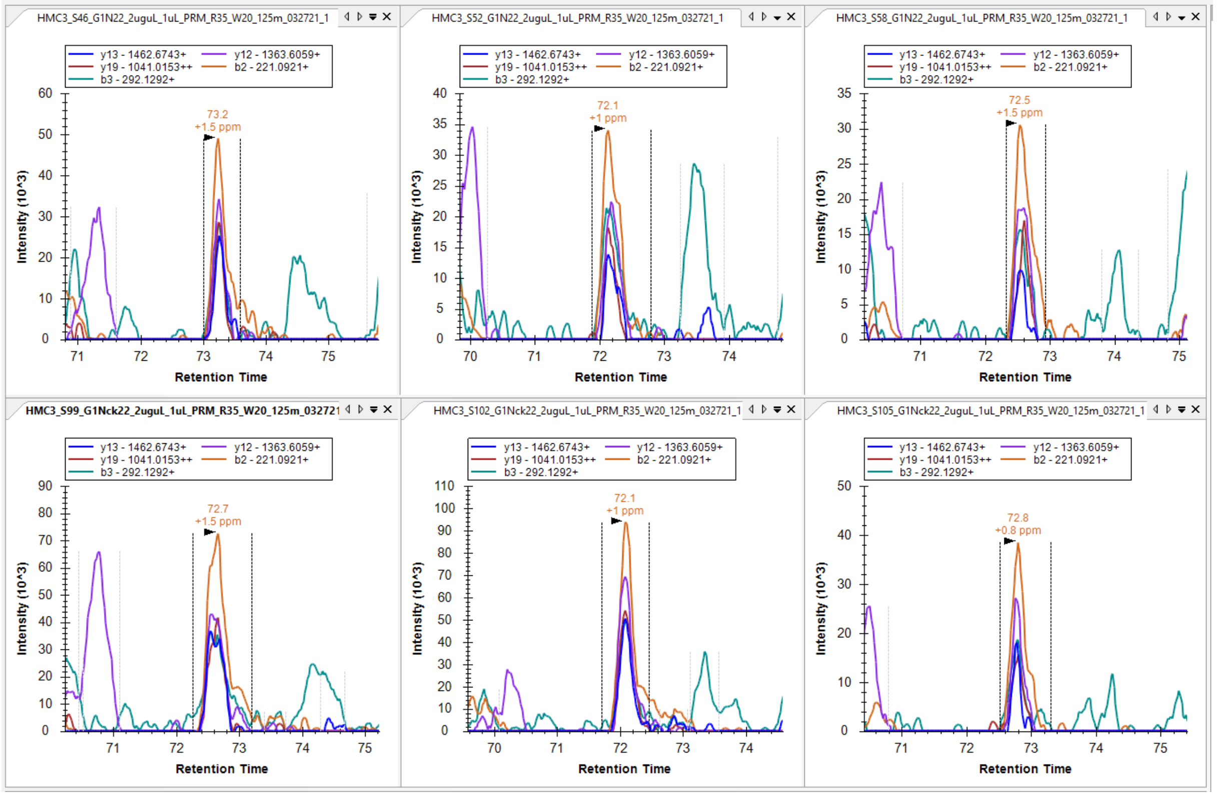

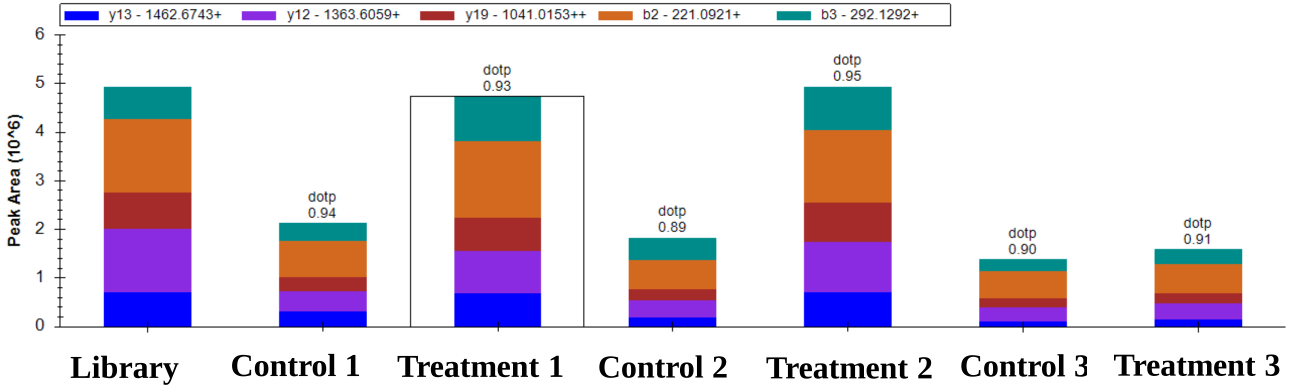

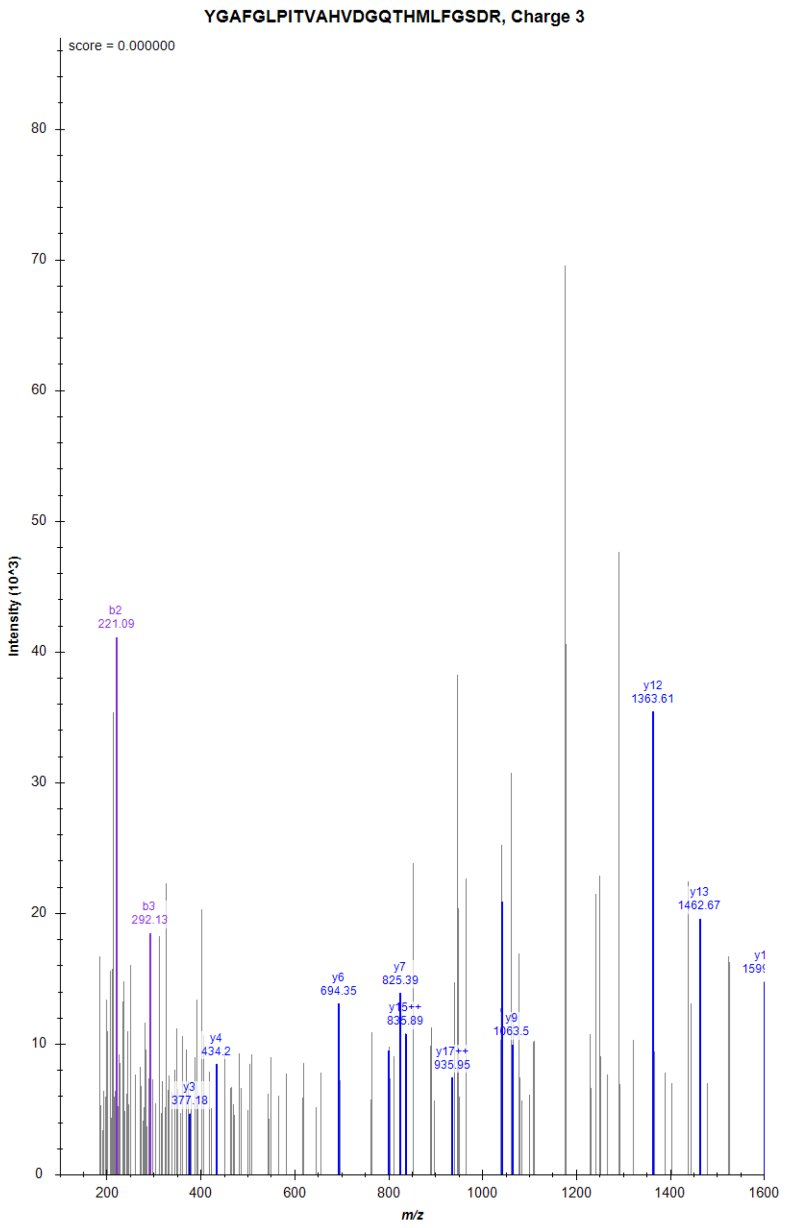

Glutathione S-transferase kappa 1 (GSTK1)

MELLAHLLGEK, Charge 2, m/z 627.35199

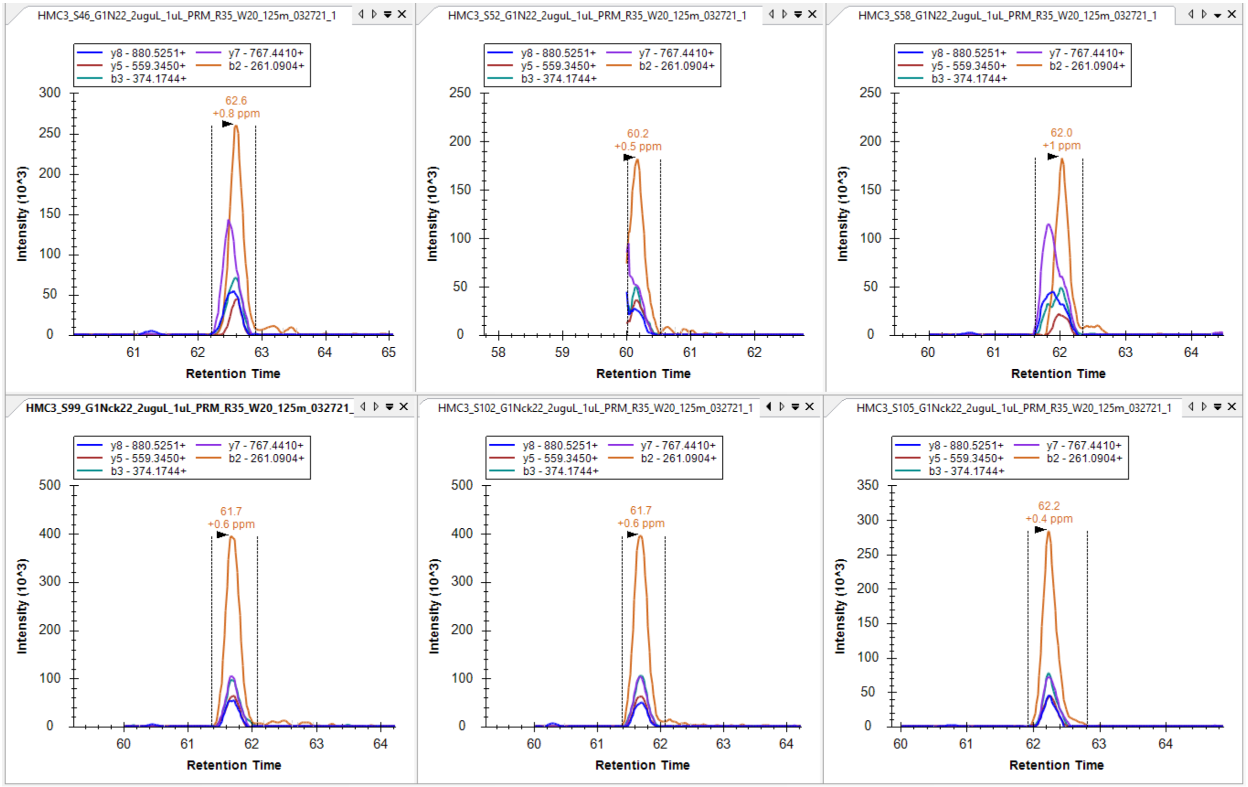

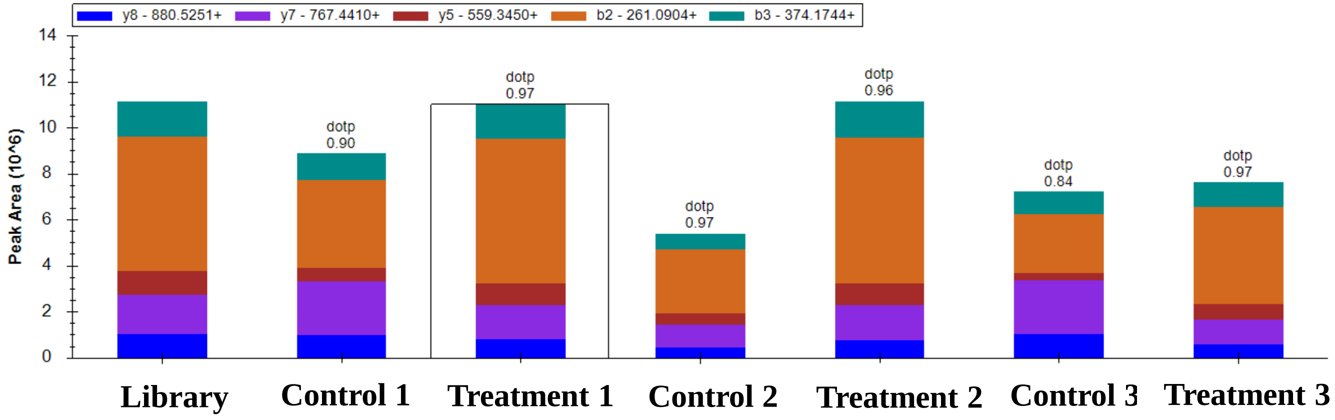

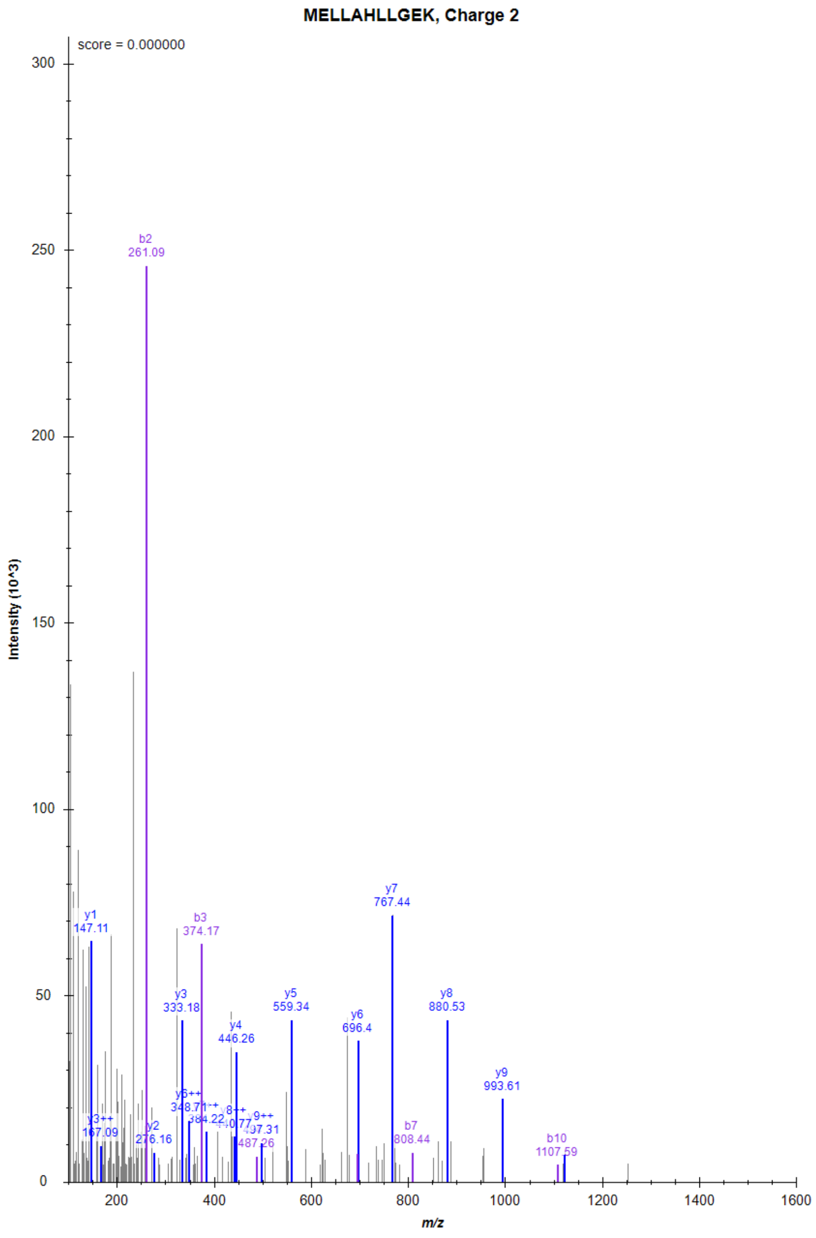

Category 2
Extracellular Matrix proteins

Collagen alpha-1(I) chain (COL1A1)

VFCNMETGETCVYPTQPSVAQK, Charge 3, m/z 811.37177

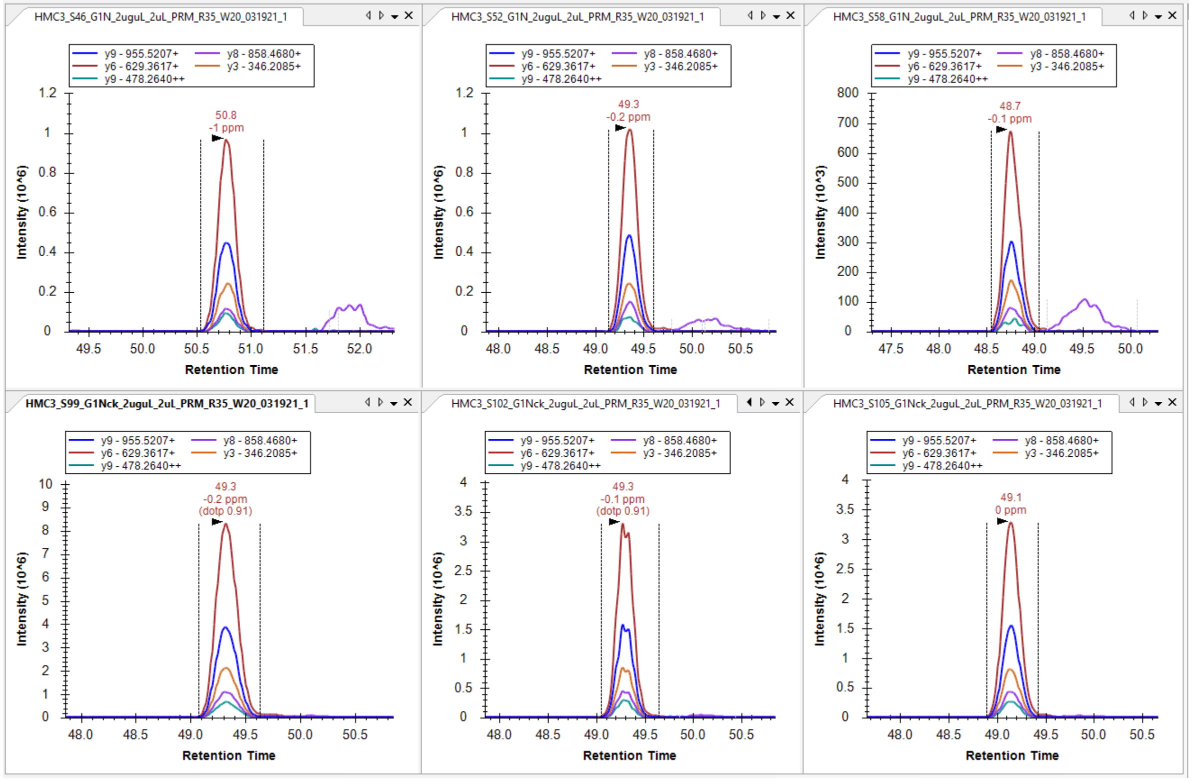

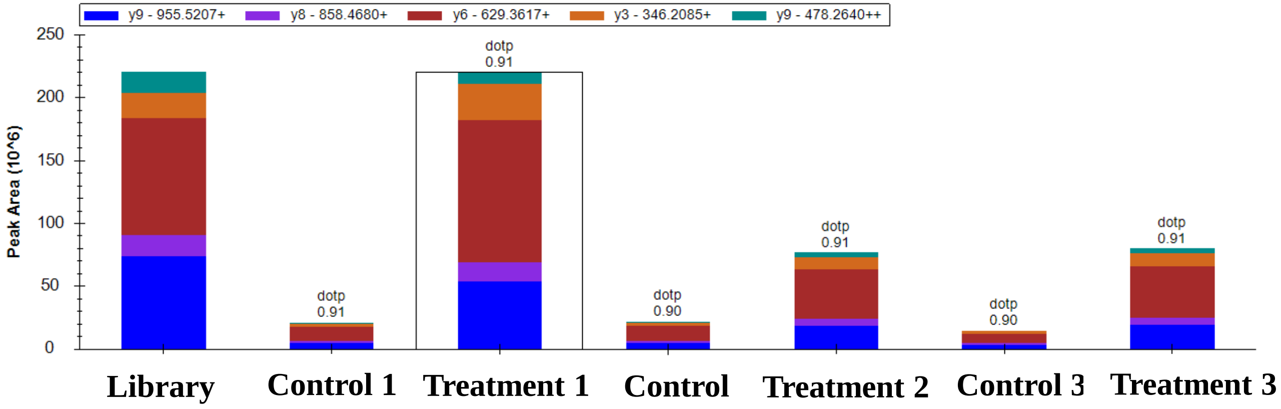

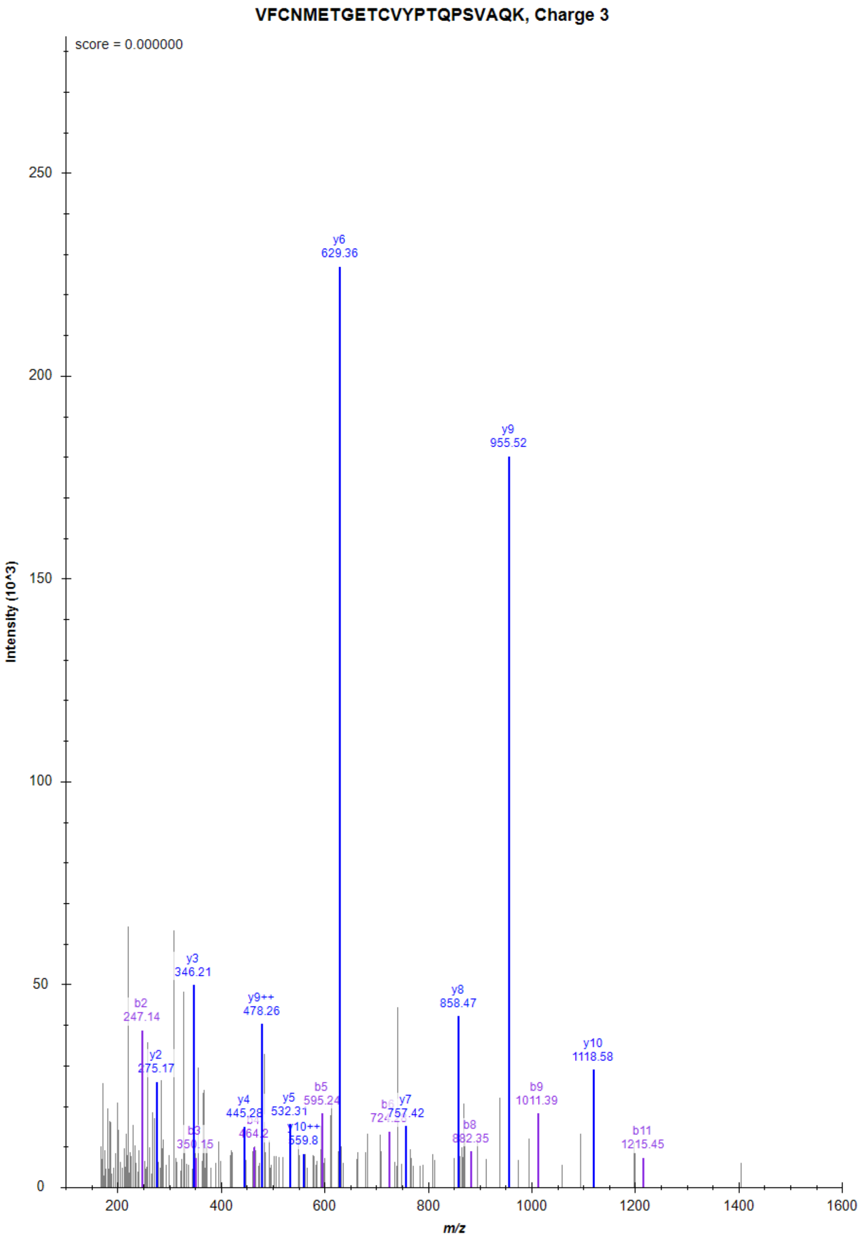

Collagen alpha-1(I) chain (COL1A1)

SGEYWIDPNQGCNLDAIK, Charge 3, m/z 674.98071

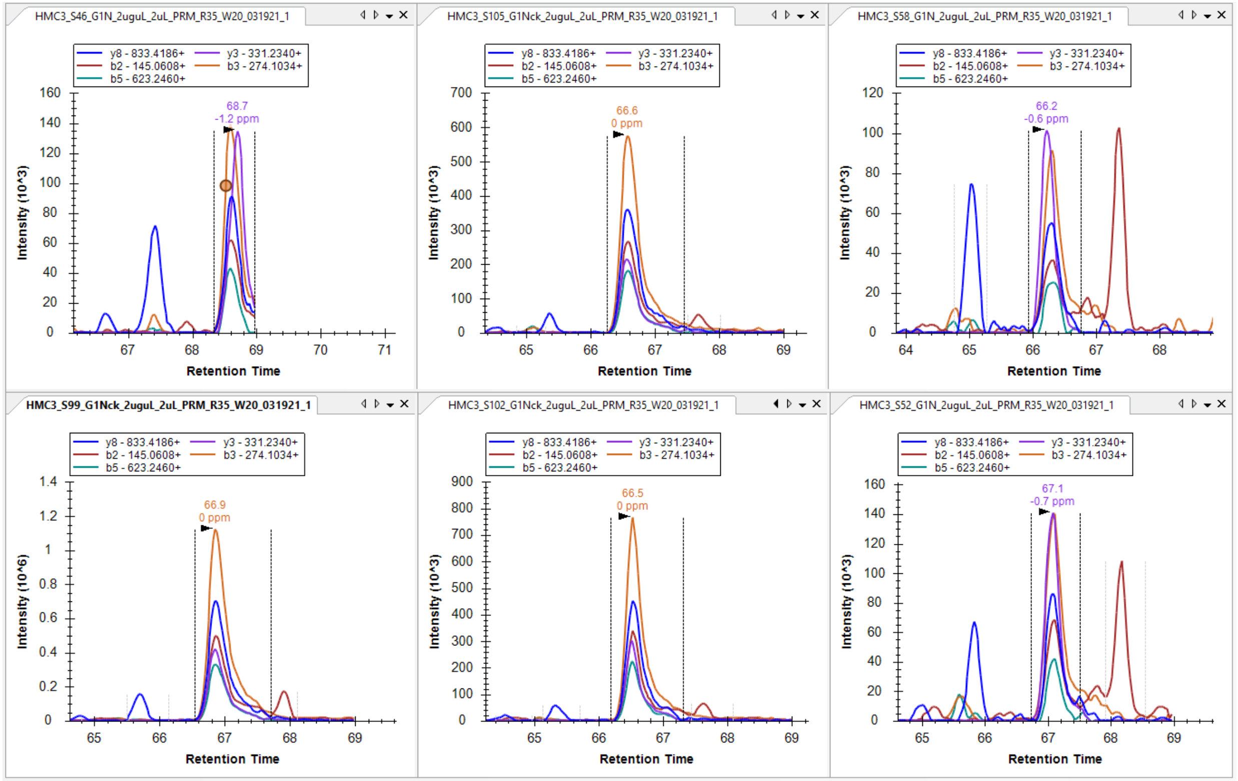

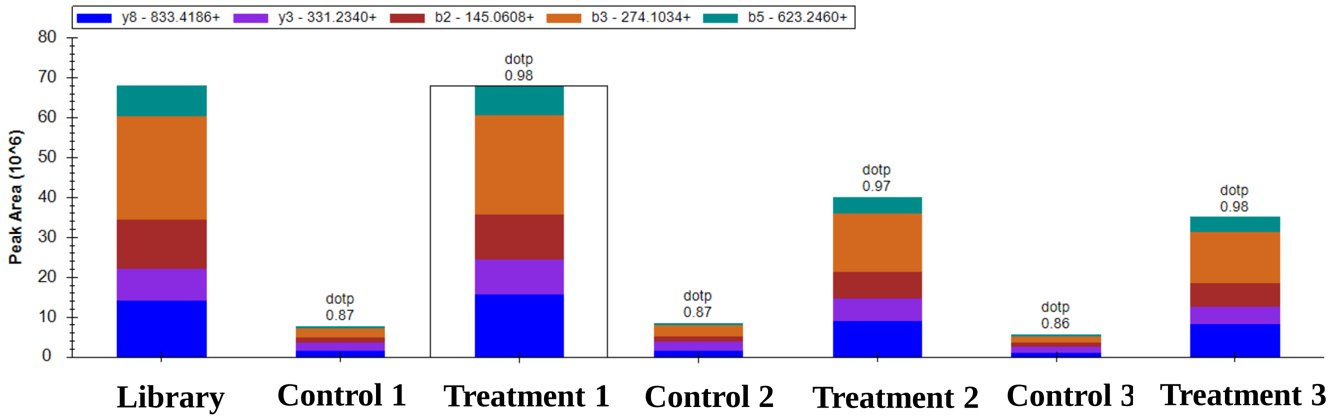

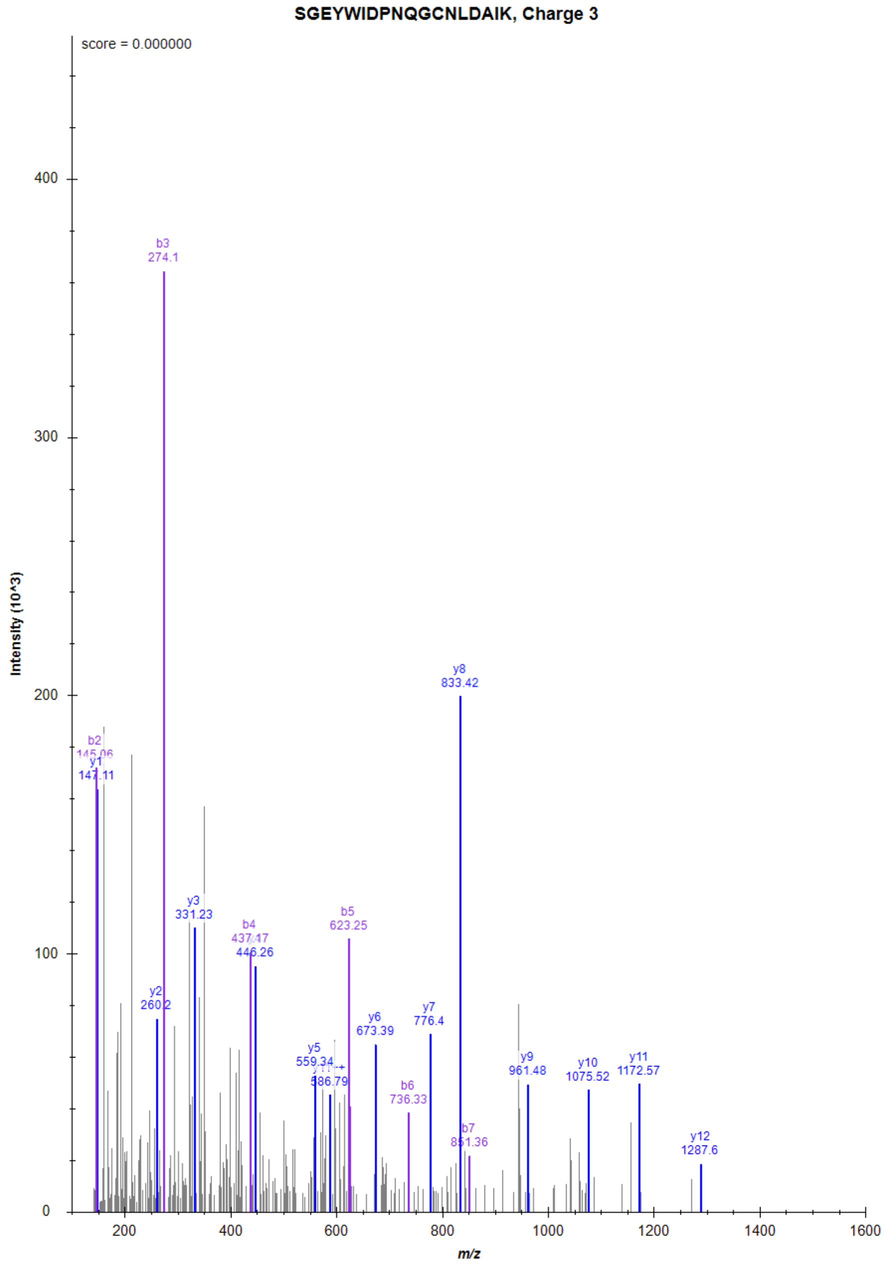

Collagen alpha-1(V) chain (COL5A1)

FLGSNDEEMSYDNNPYIR, Charge 3, m/z 721.98218

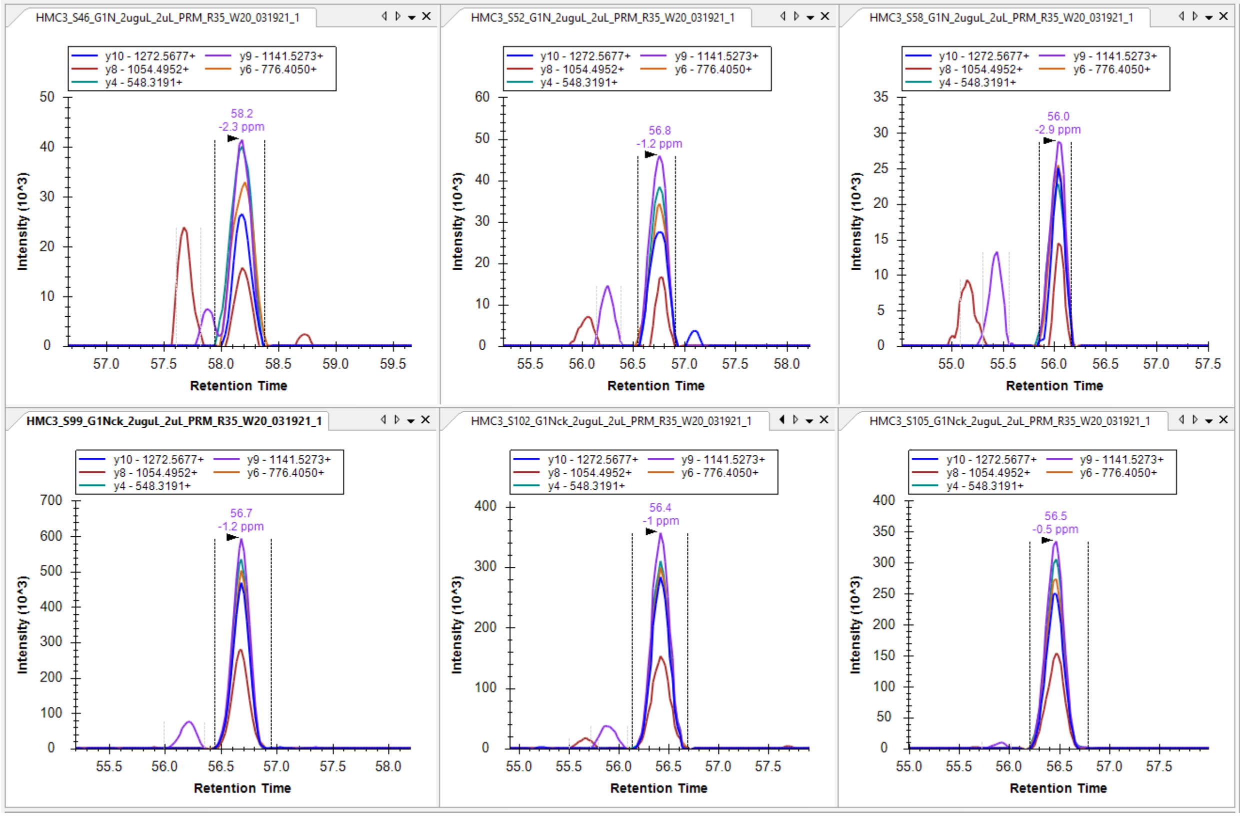

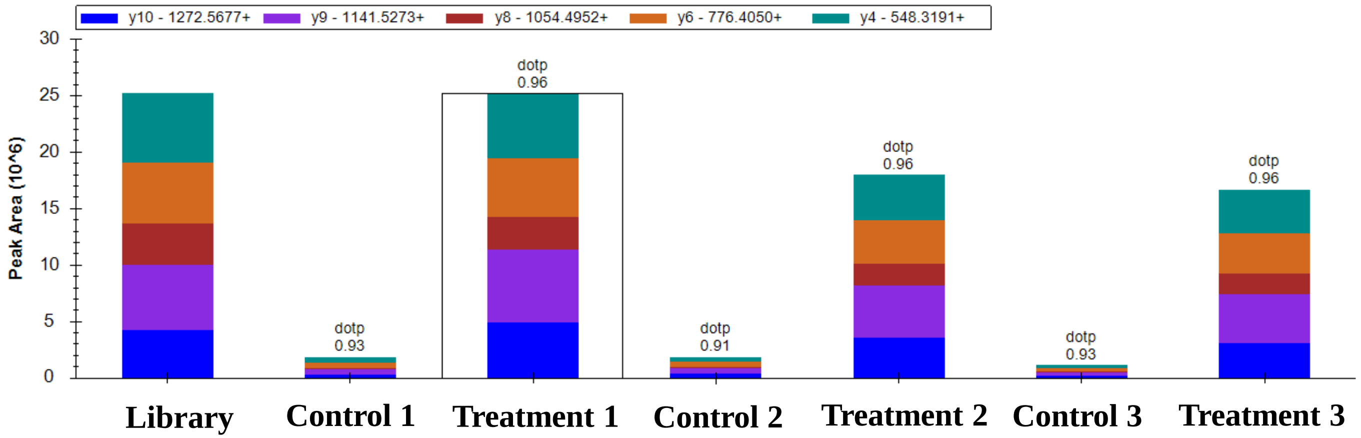

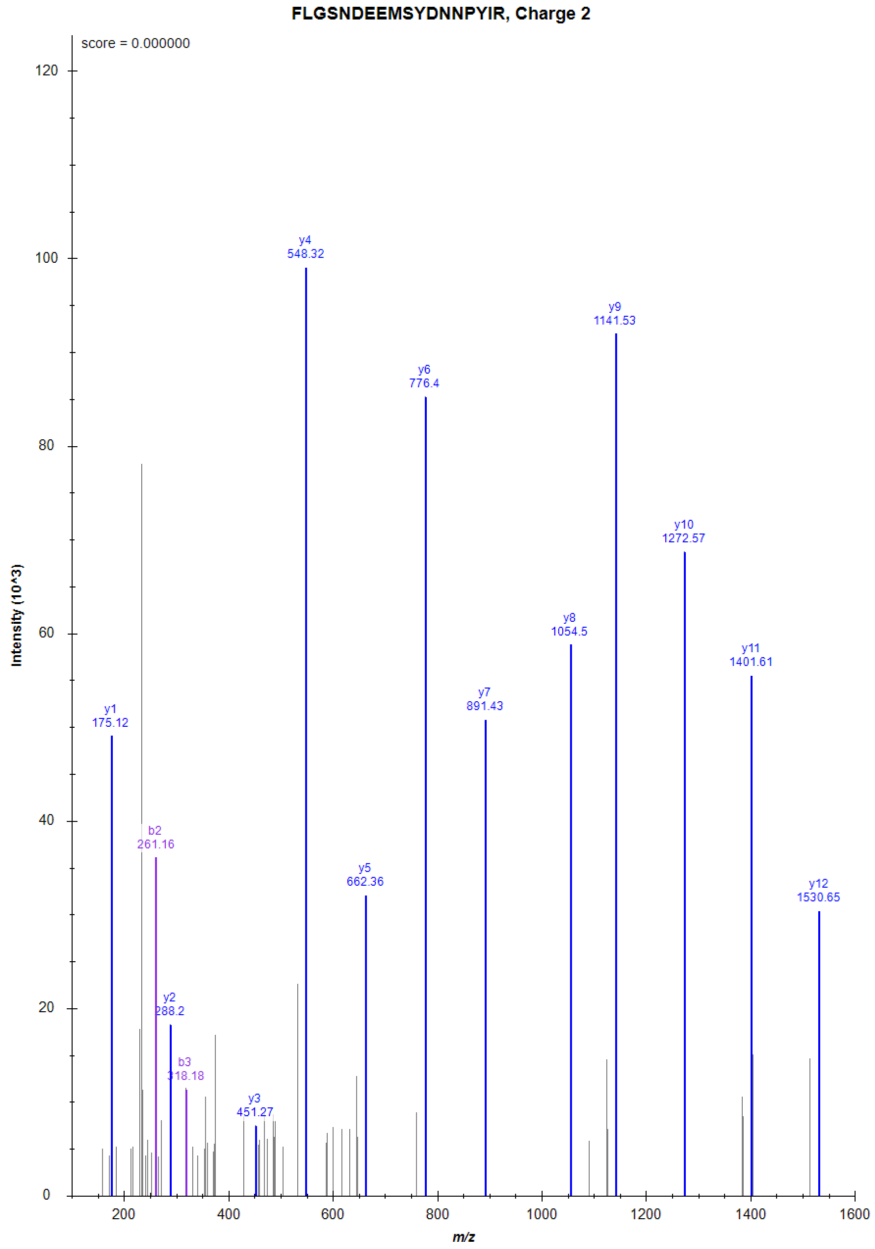

Tenascin C (TNC)

AVDIPGLEAATPYR, Charge 2, m/z 736.89502

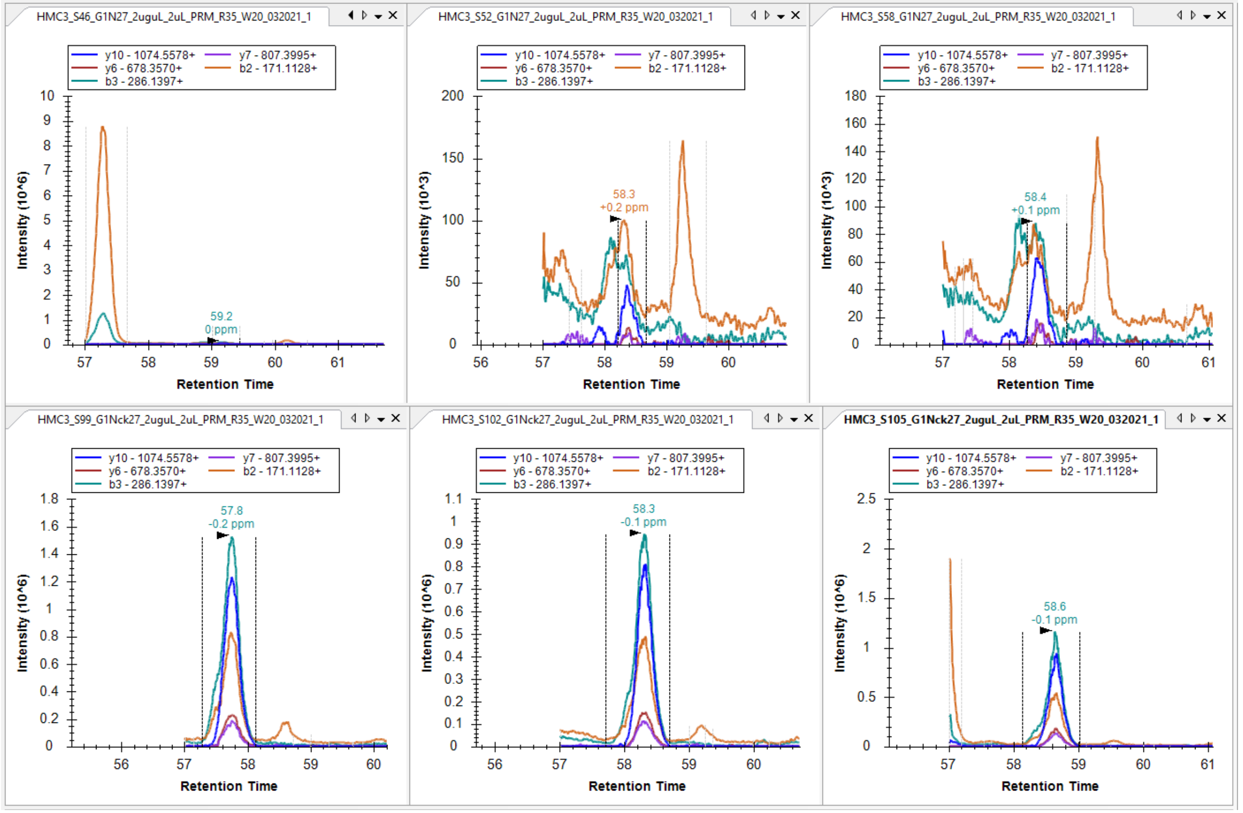

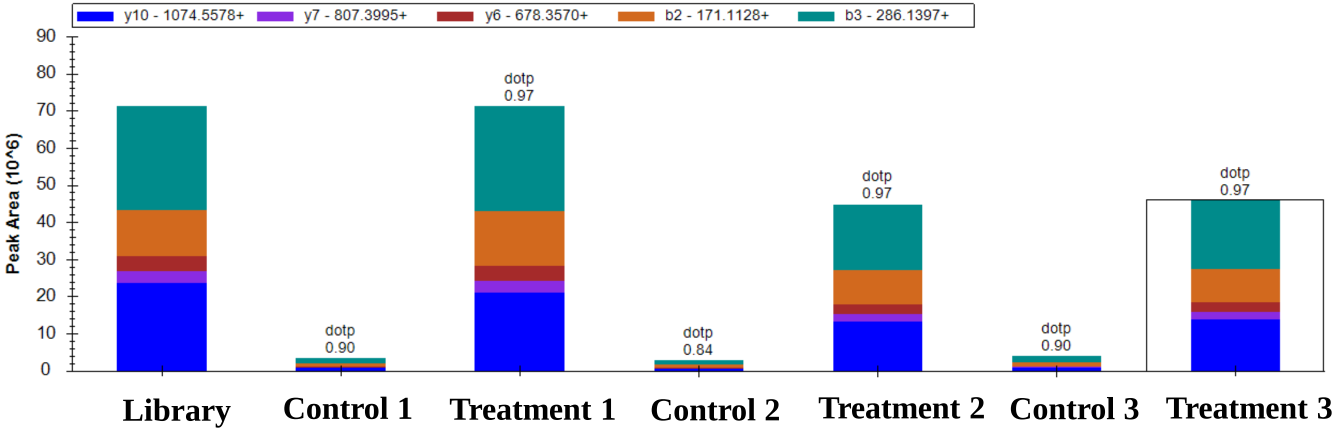

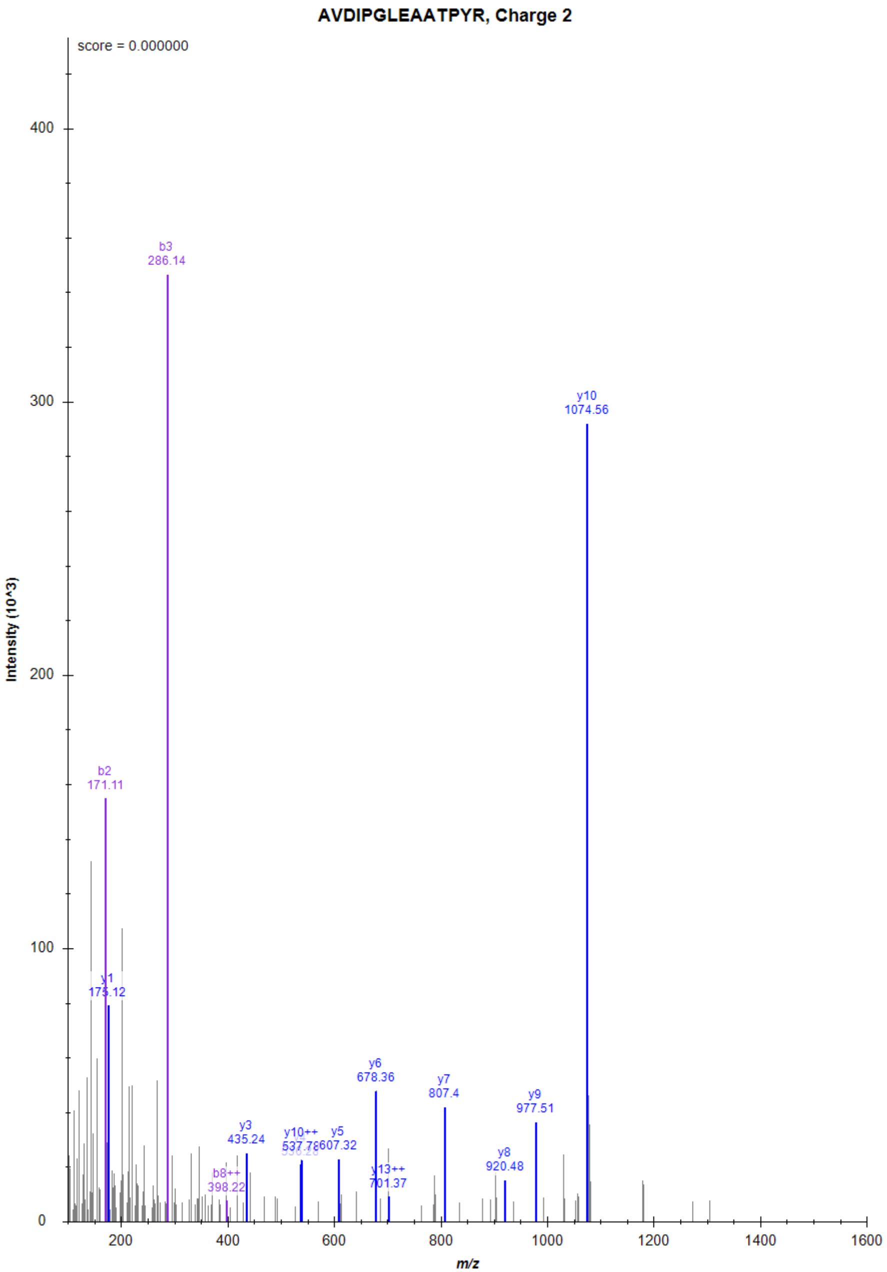

Transforming growth factor-beta-induced protein ig-h3 (TGFBI)

VISTITNNIQQIIEIEDTFETLR, Charge 3, m/z 897.48419

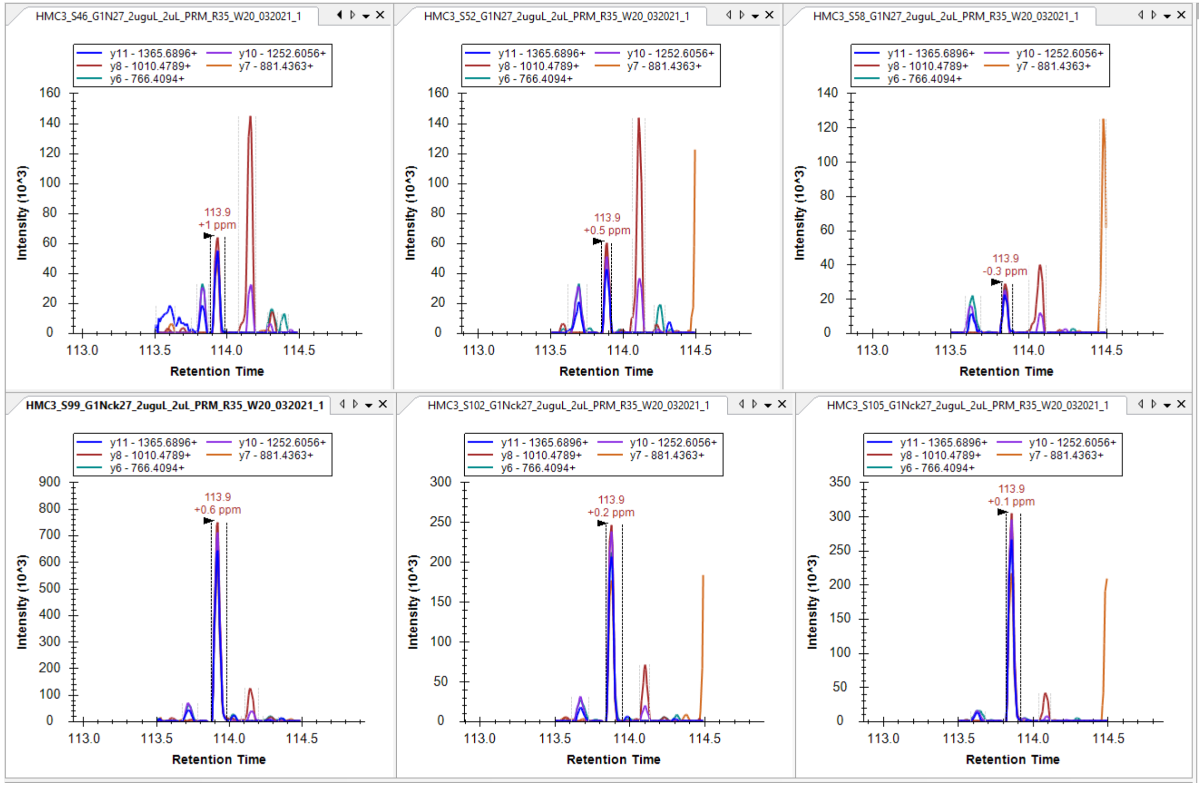

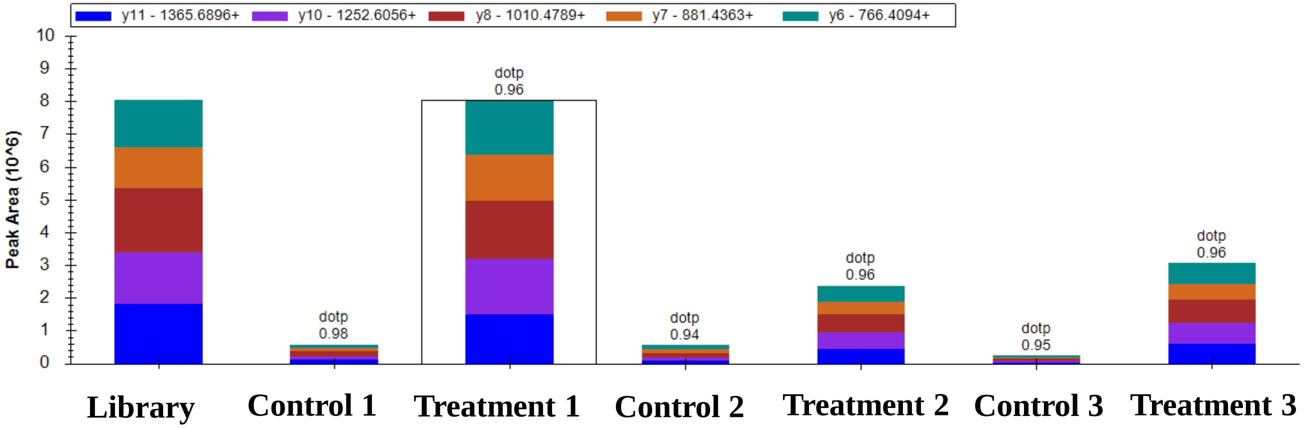

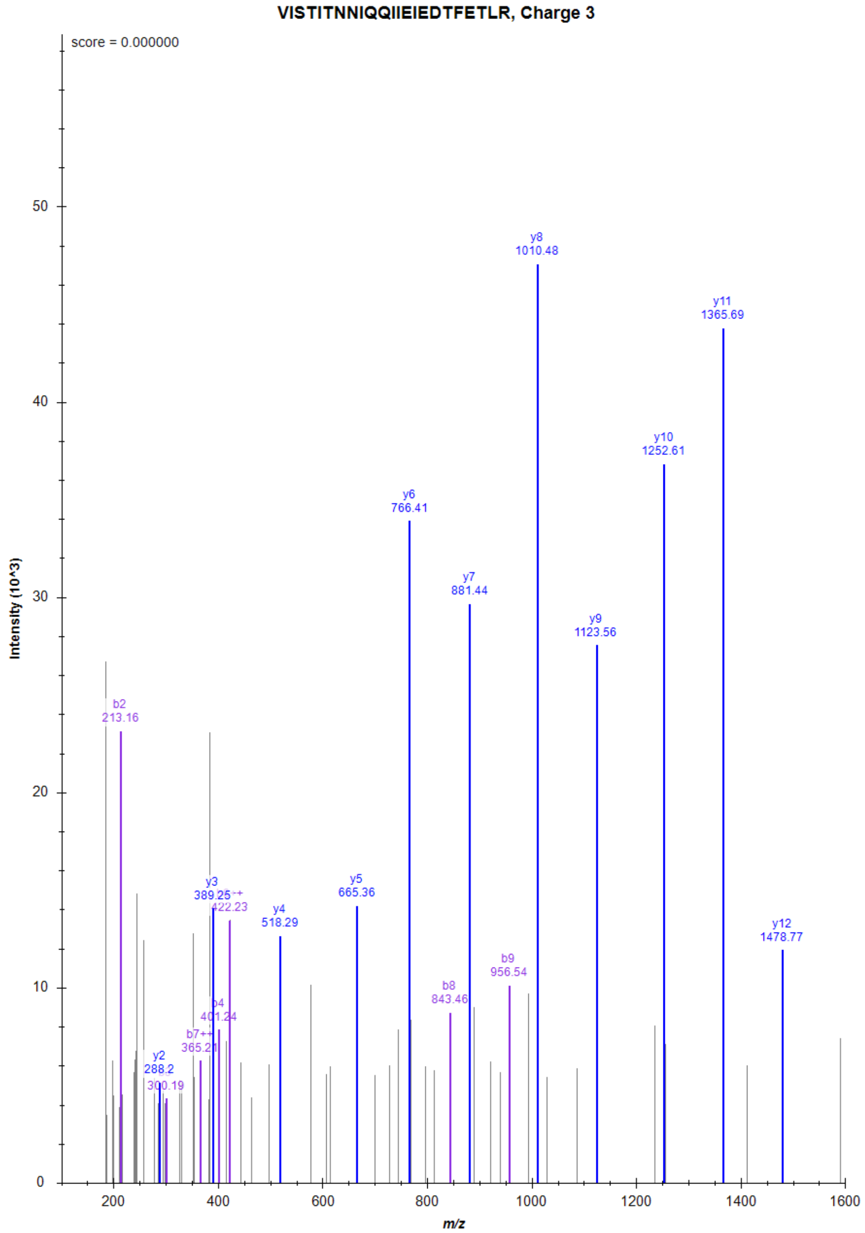

Cathepsin D (CTSD)

LVDQNIFSFYLSR, Charge 2, m/z 801.42249

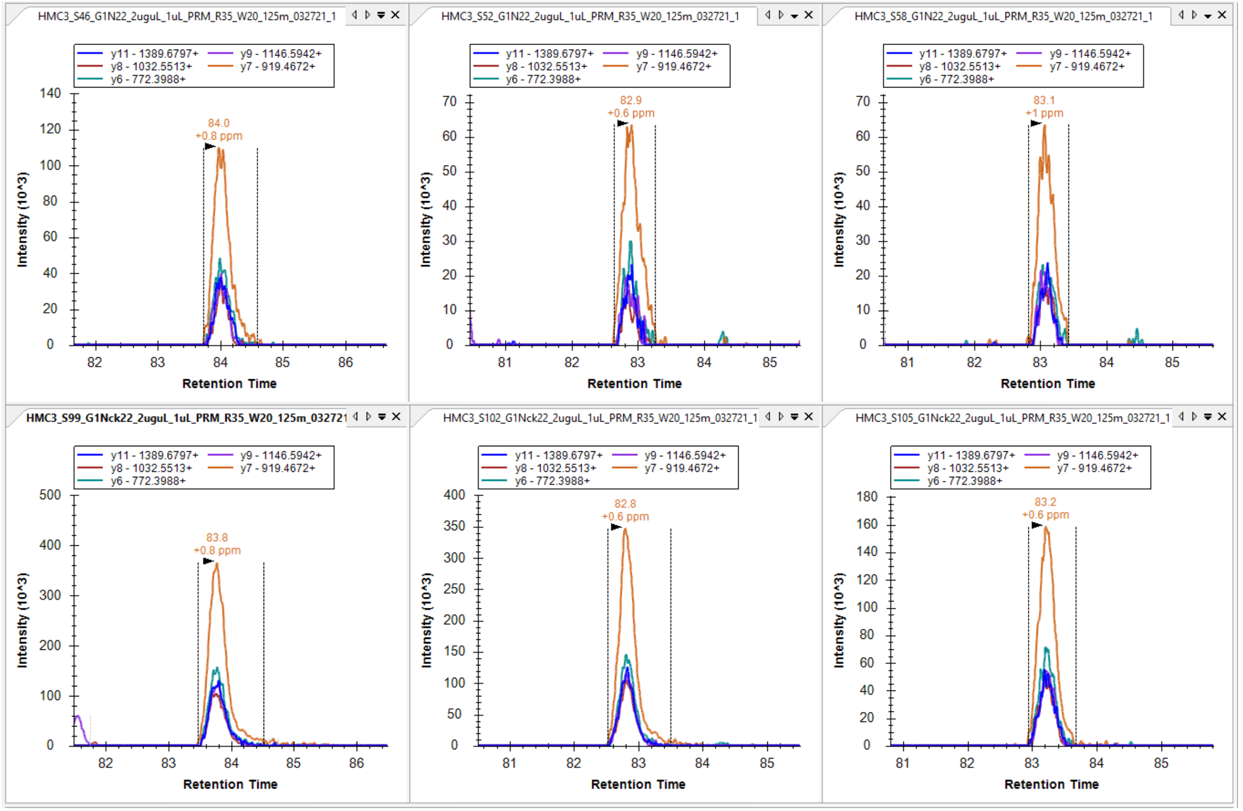

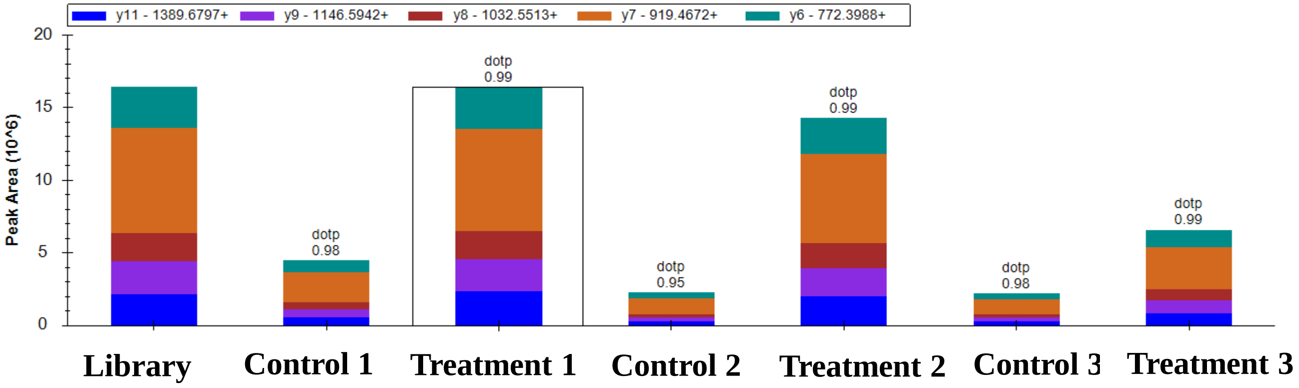

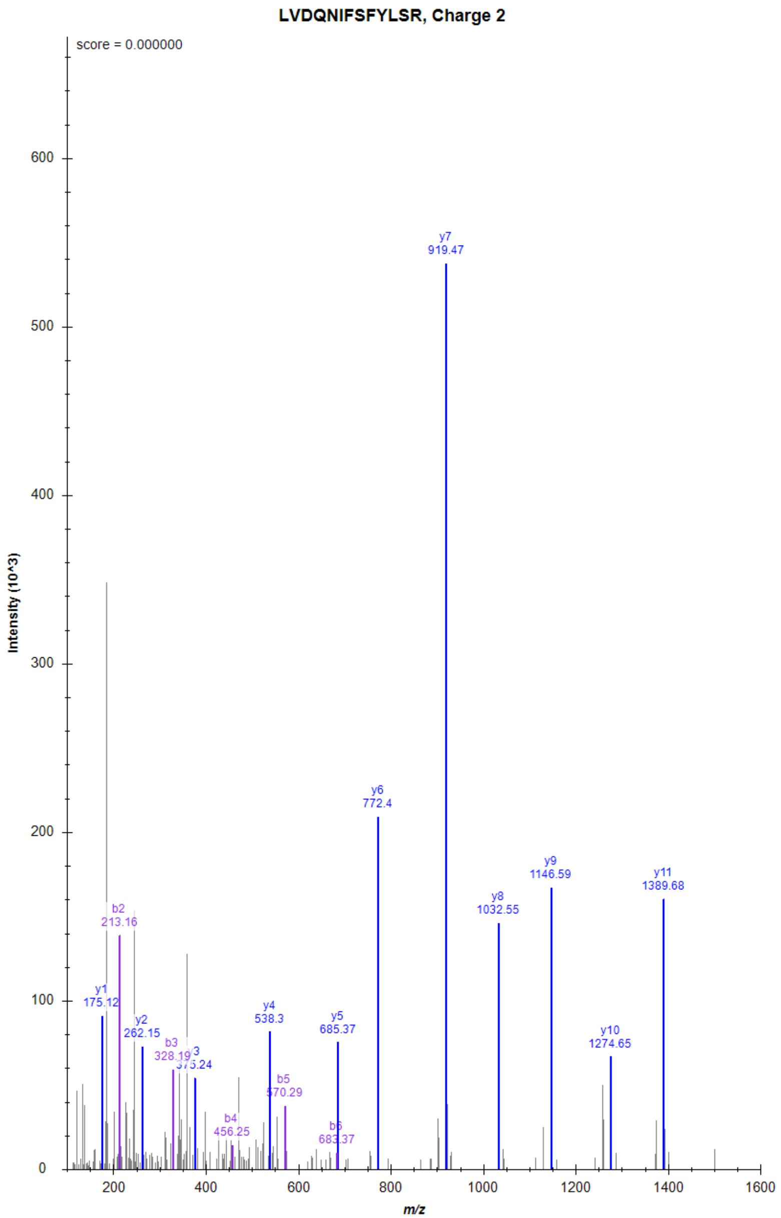

Cathepsin D (CTSD)

ISVNNVLPVFDNLMQQK, Charge 2, m/z 980.02466

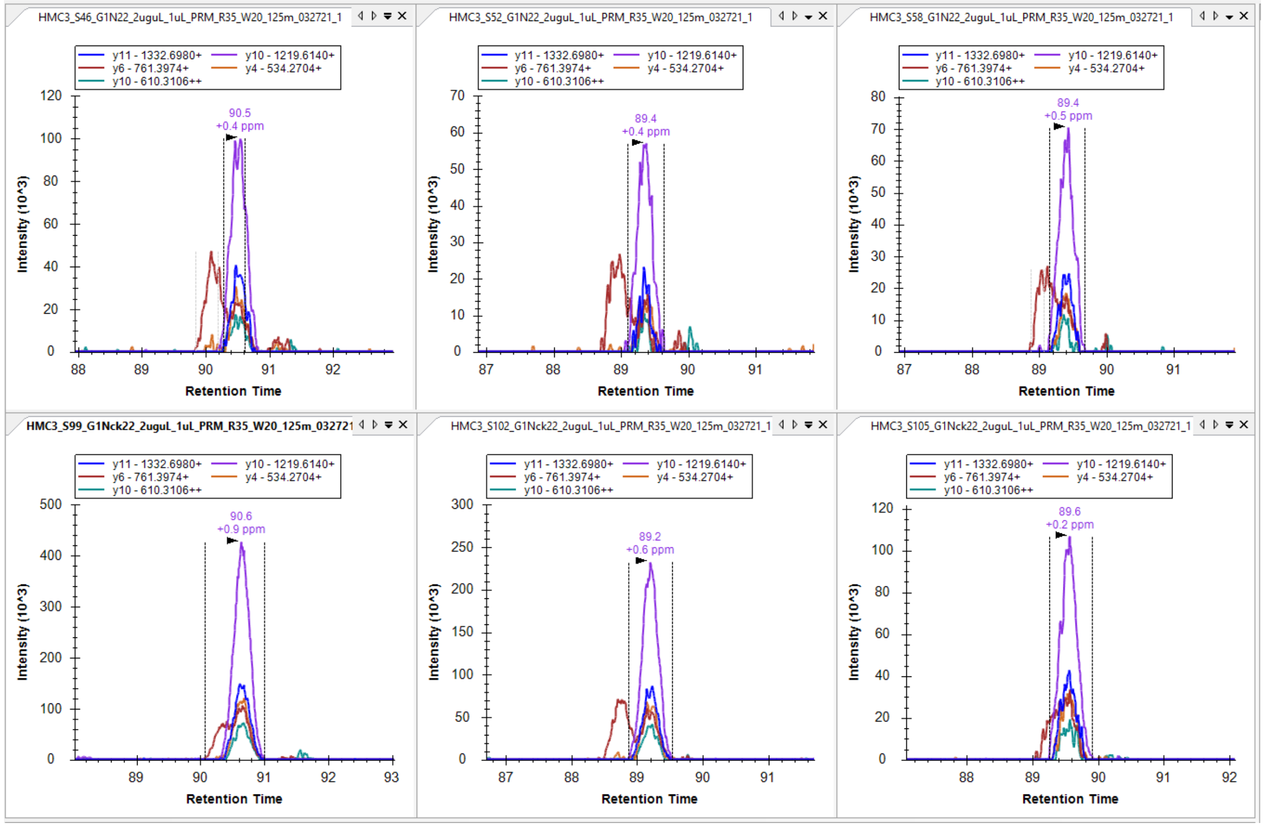

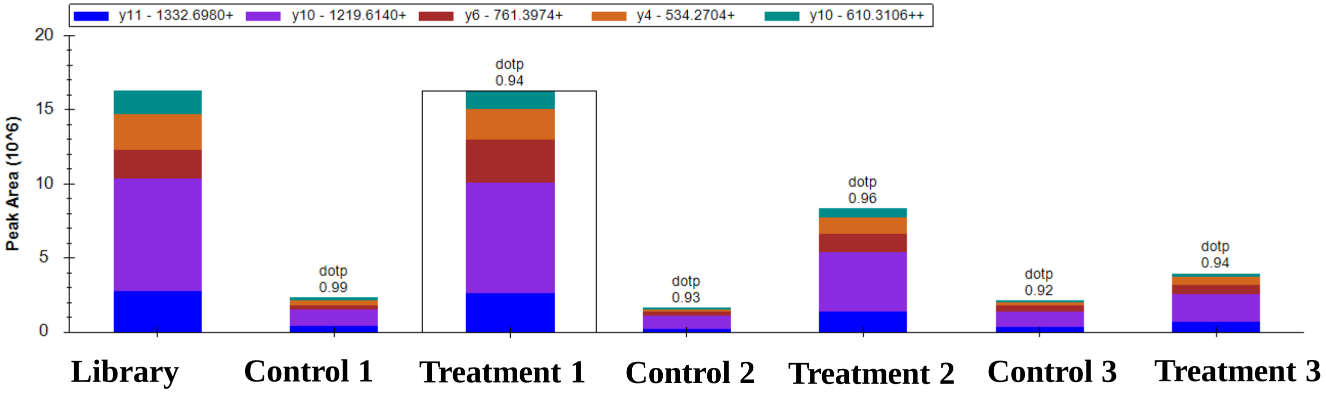

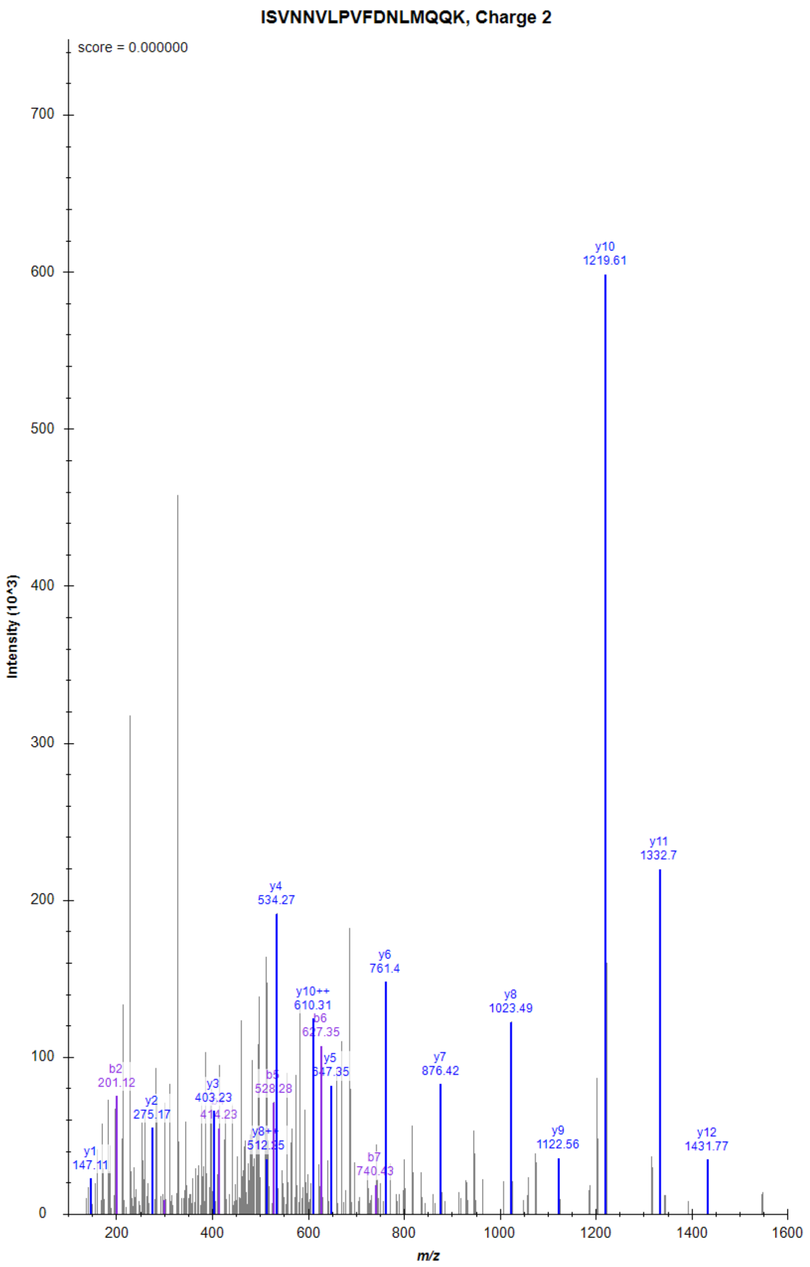

Cathepsin B (CTSB)

SGVYQHVTGEMMGGHAIR, Charge 3, m/z 643.9754

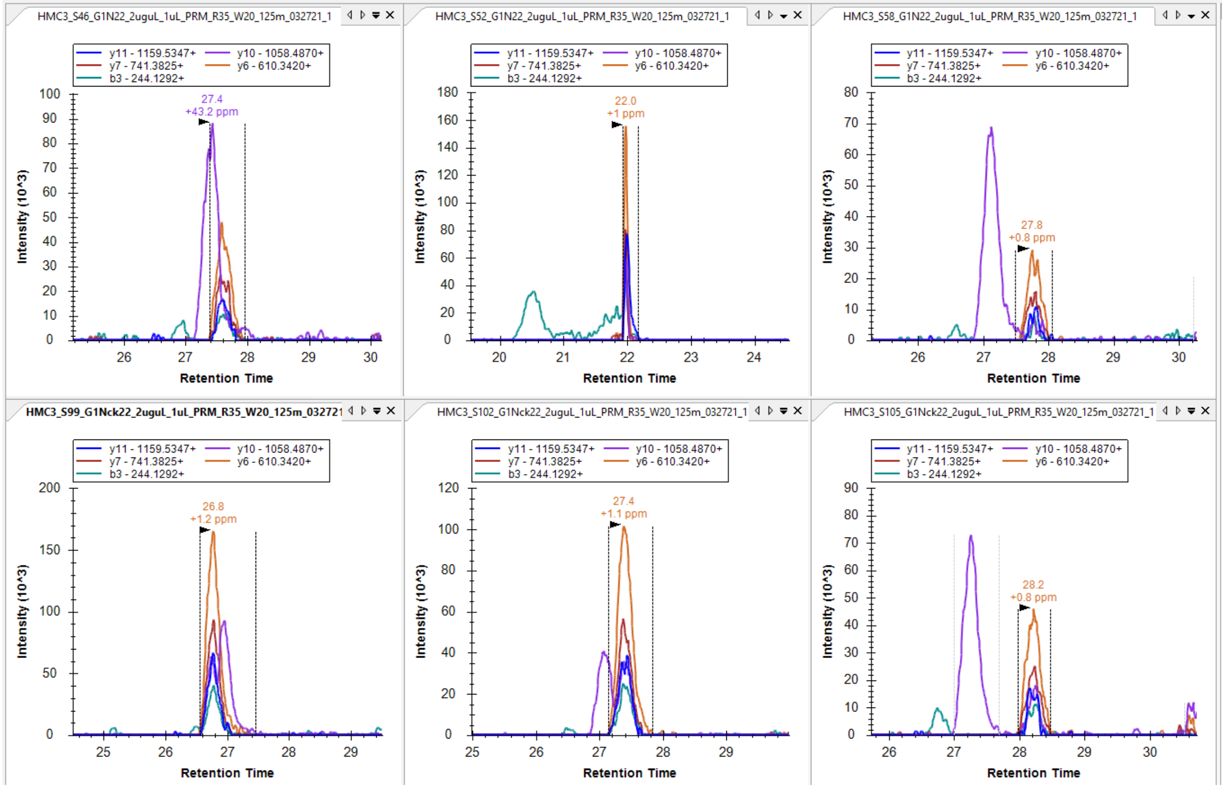

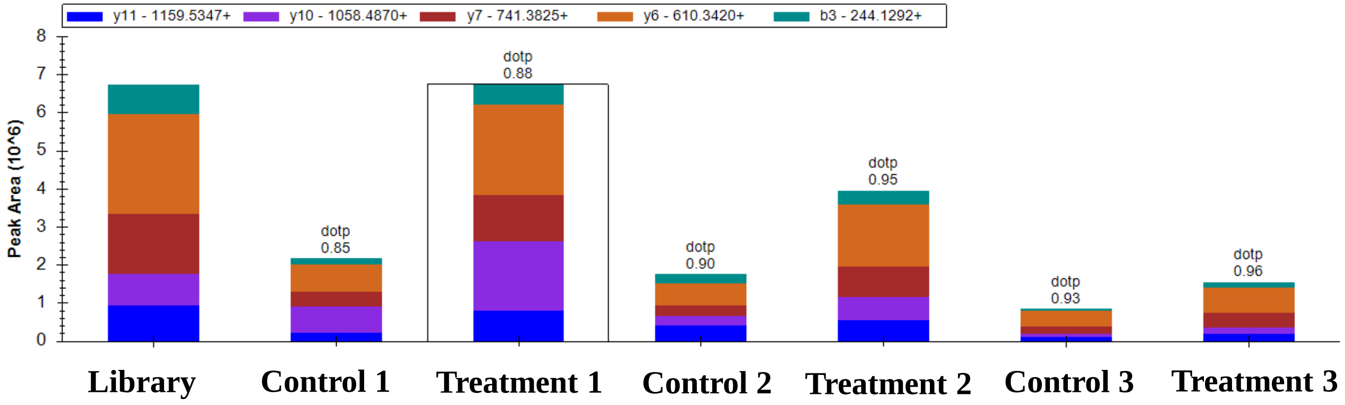

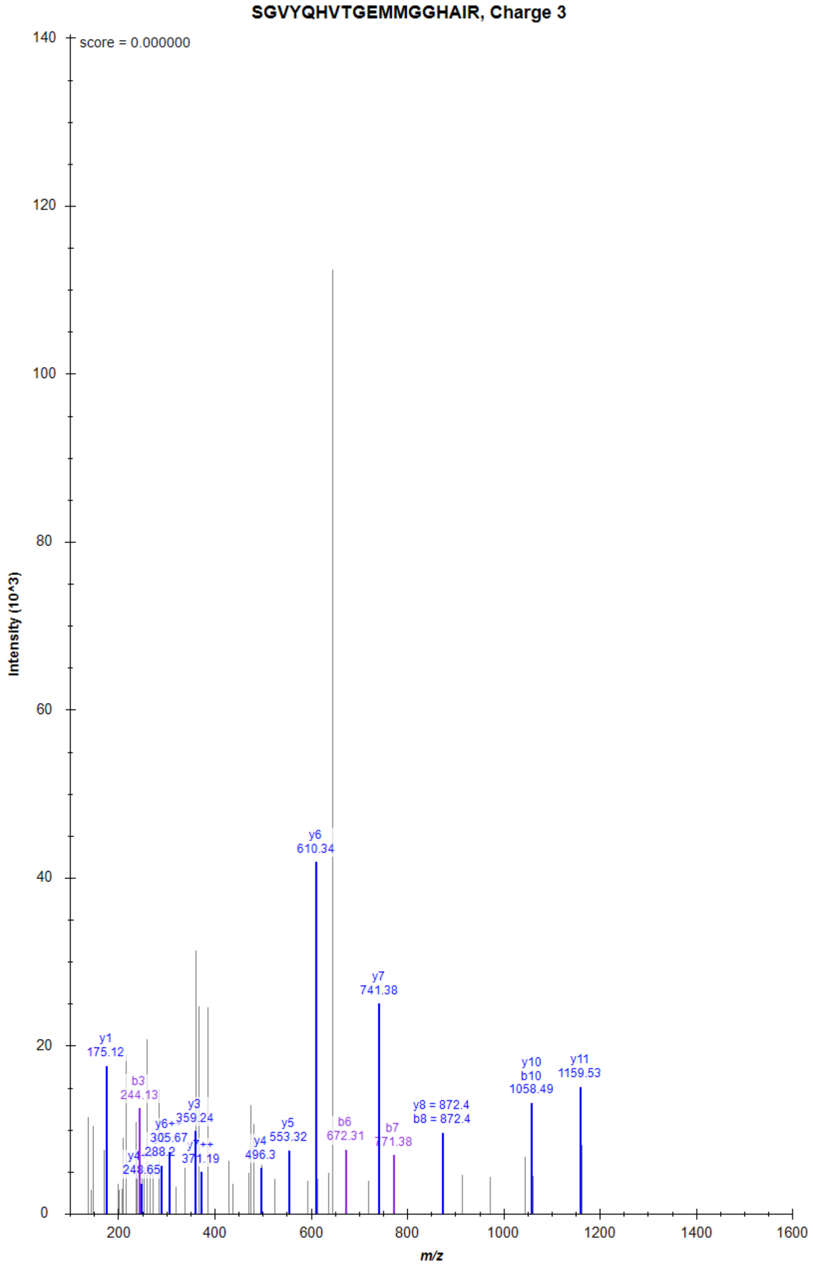

Transforming growth factor beta-1-induced transcript 1 protein (TGFB1I1)

GSLDTMLGLLQSDLSR, Charge 2, m/z 853.44556

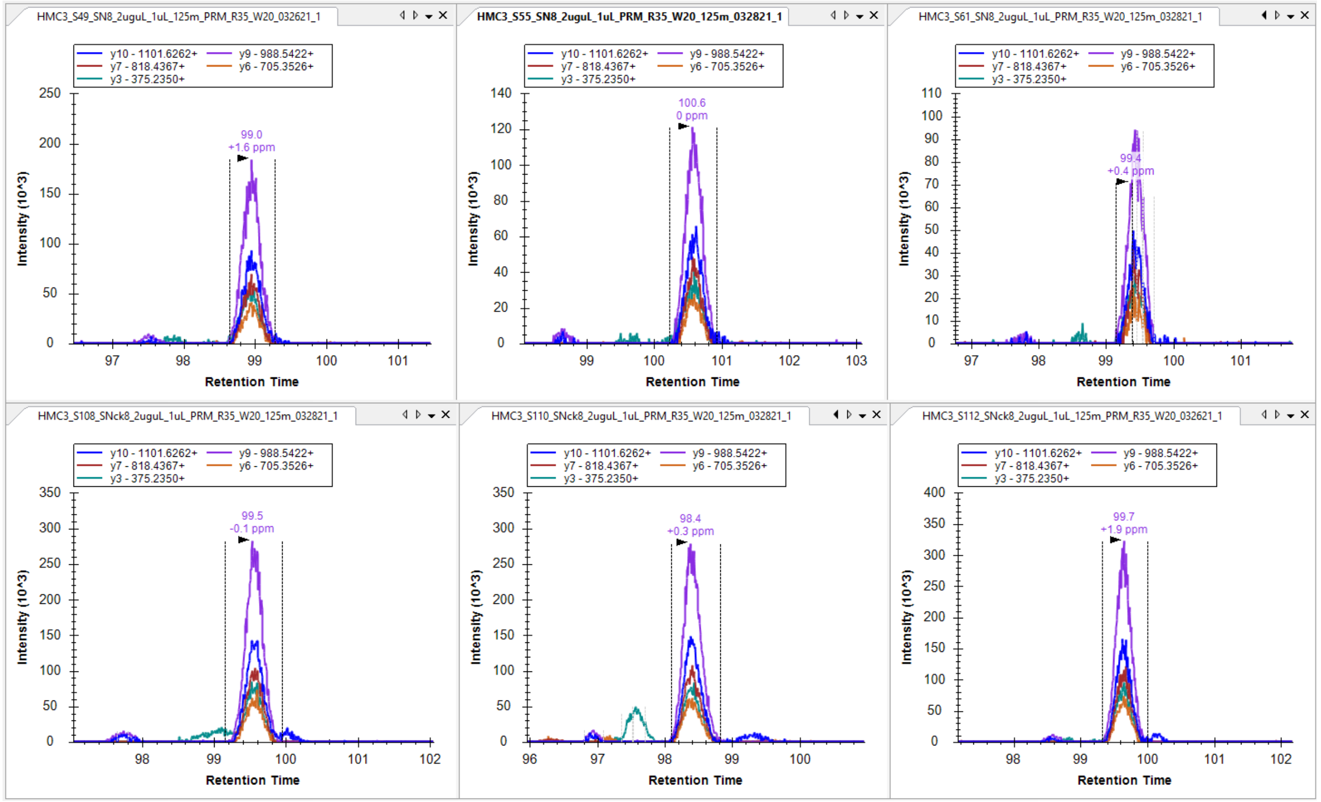

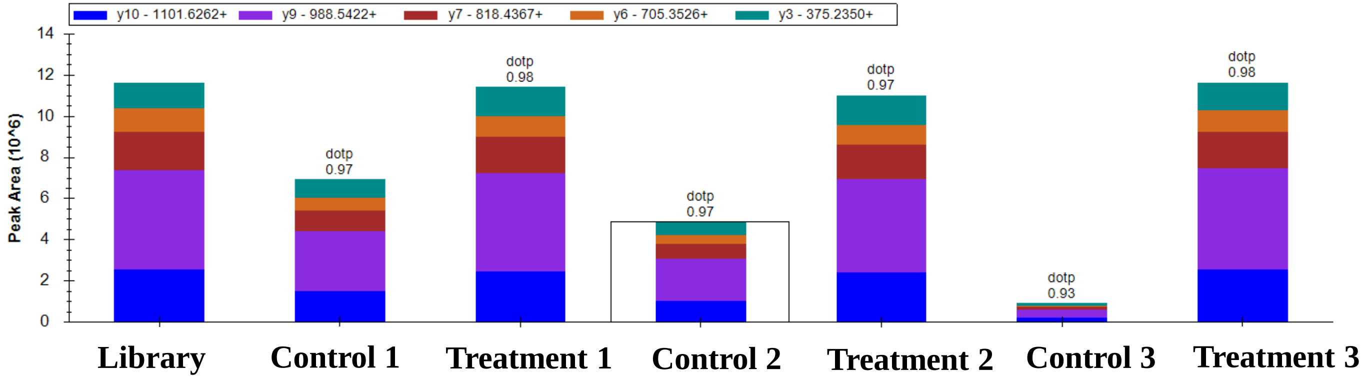

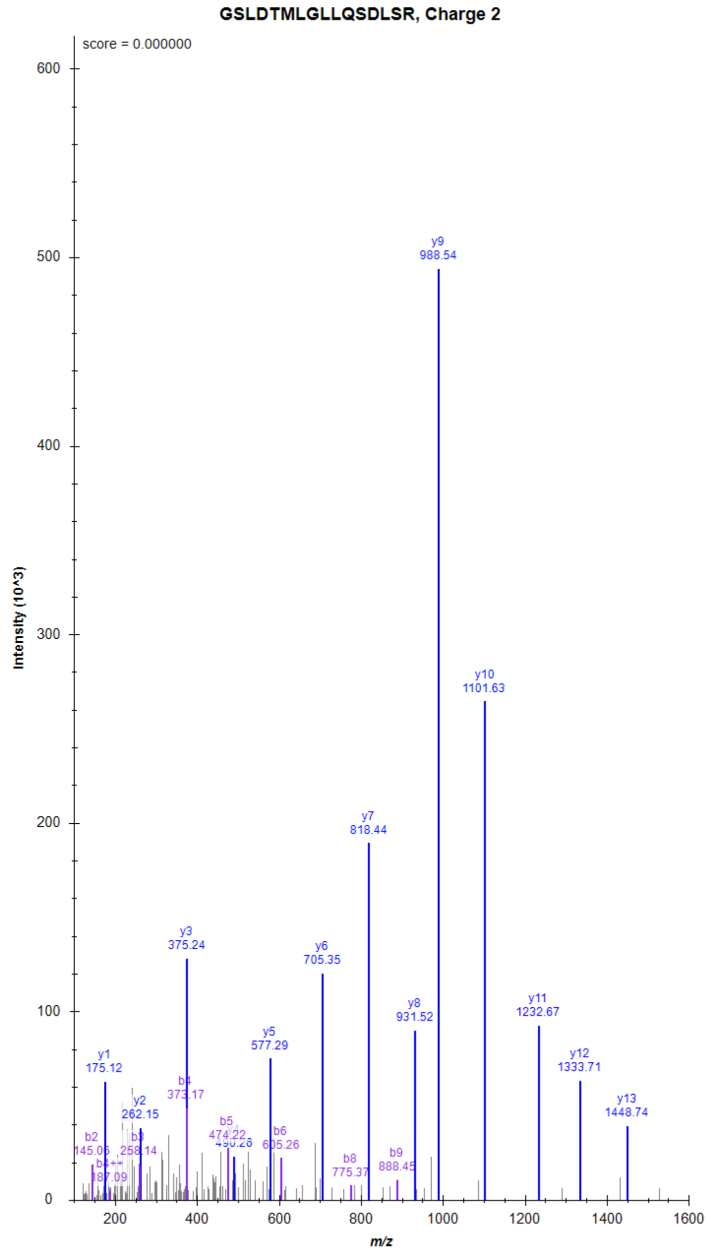

Integrin beta-1 (ITGB1)

LKPEDITQIQPQQLVLR, Charge 2, m/z 1010.08093

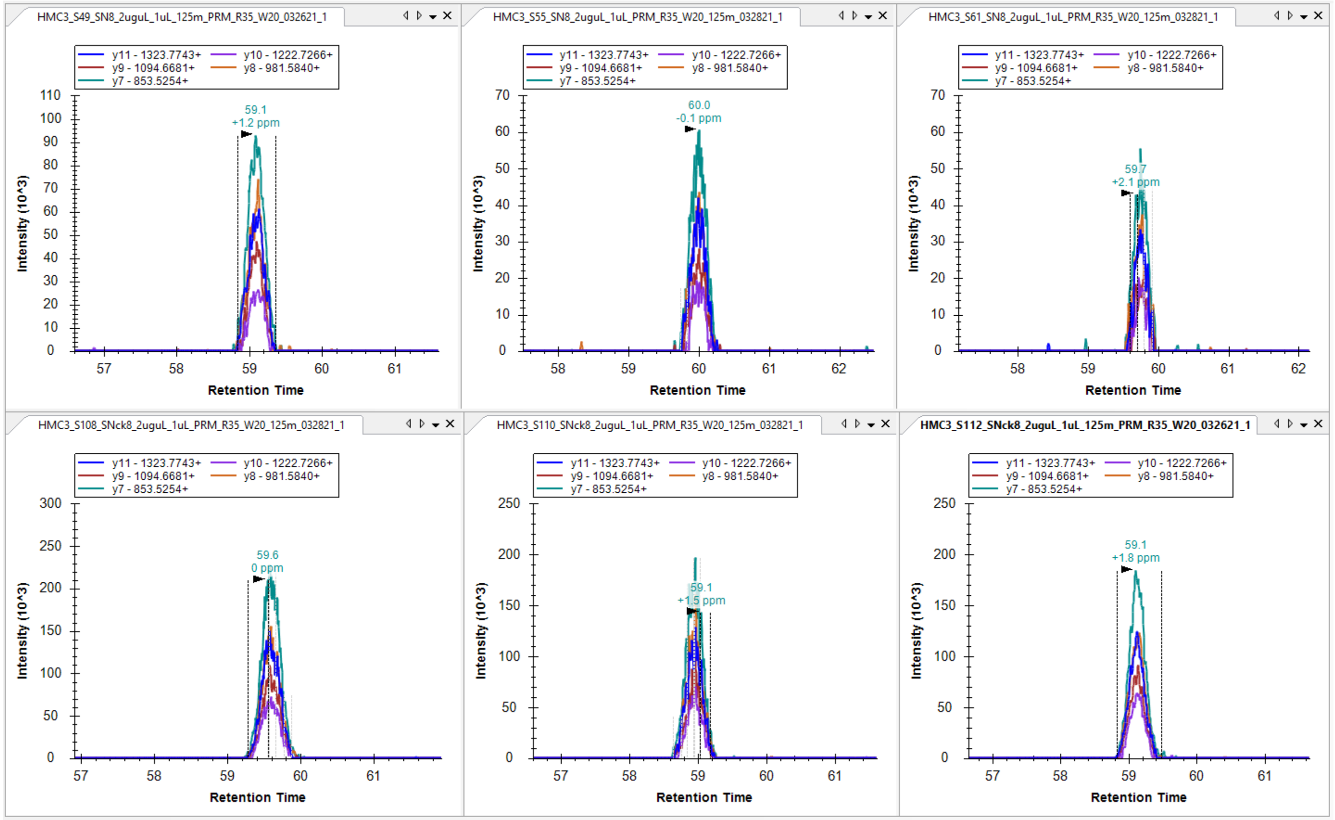

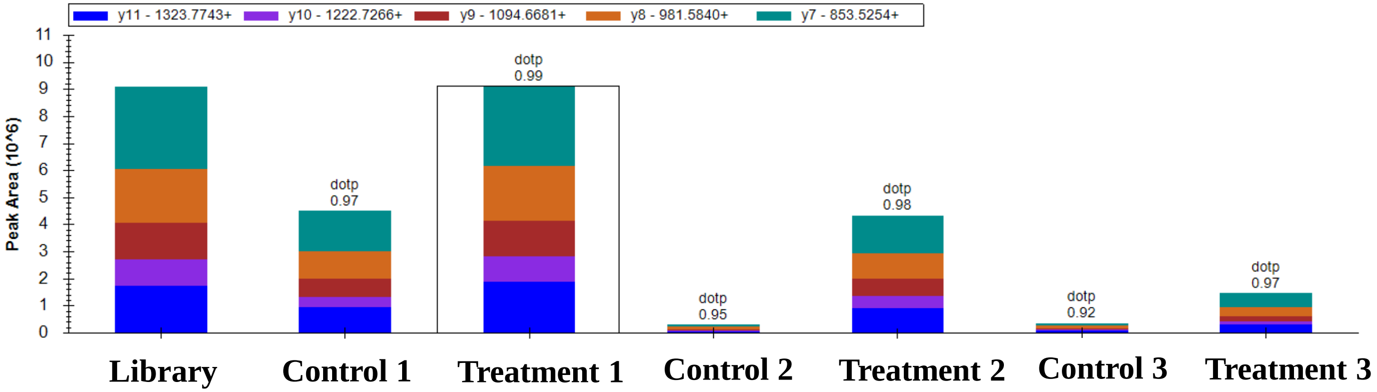

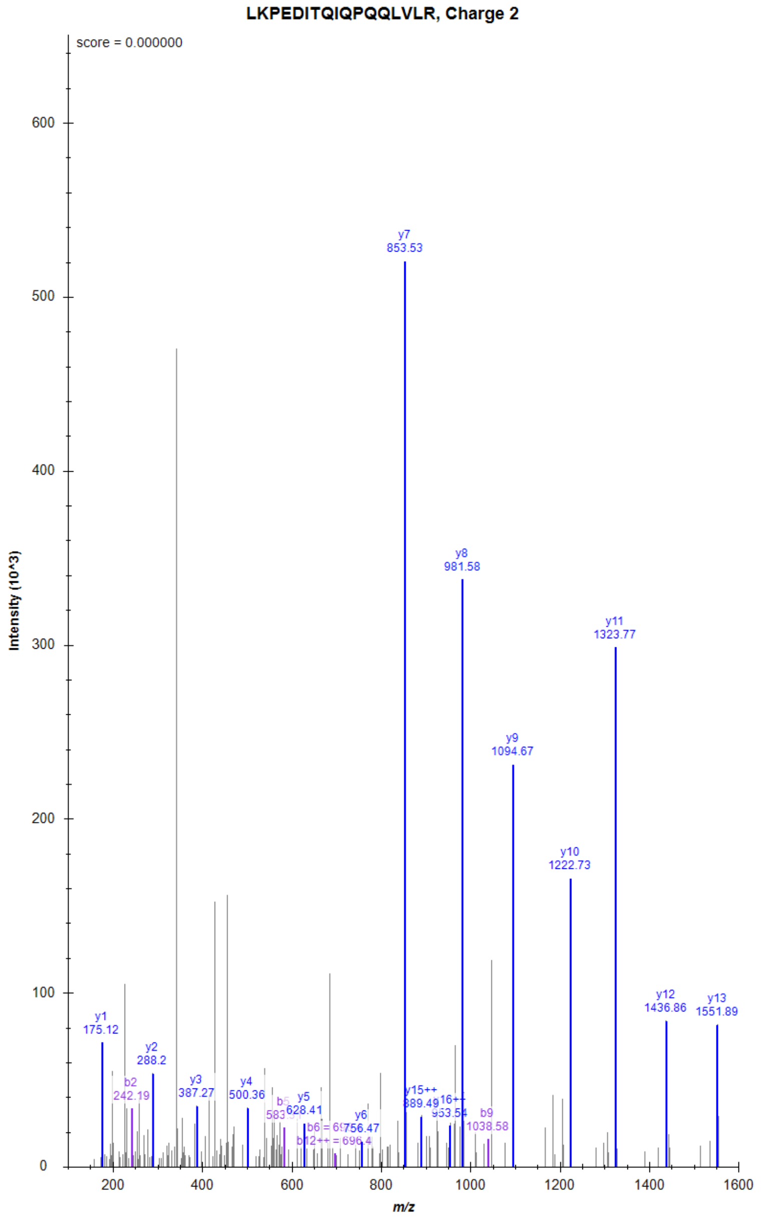

Integrin beta-1 (ITGB1)

SLGTDLMNEMR, Charge 2, m/z 633.79437

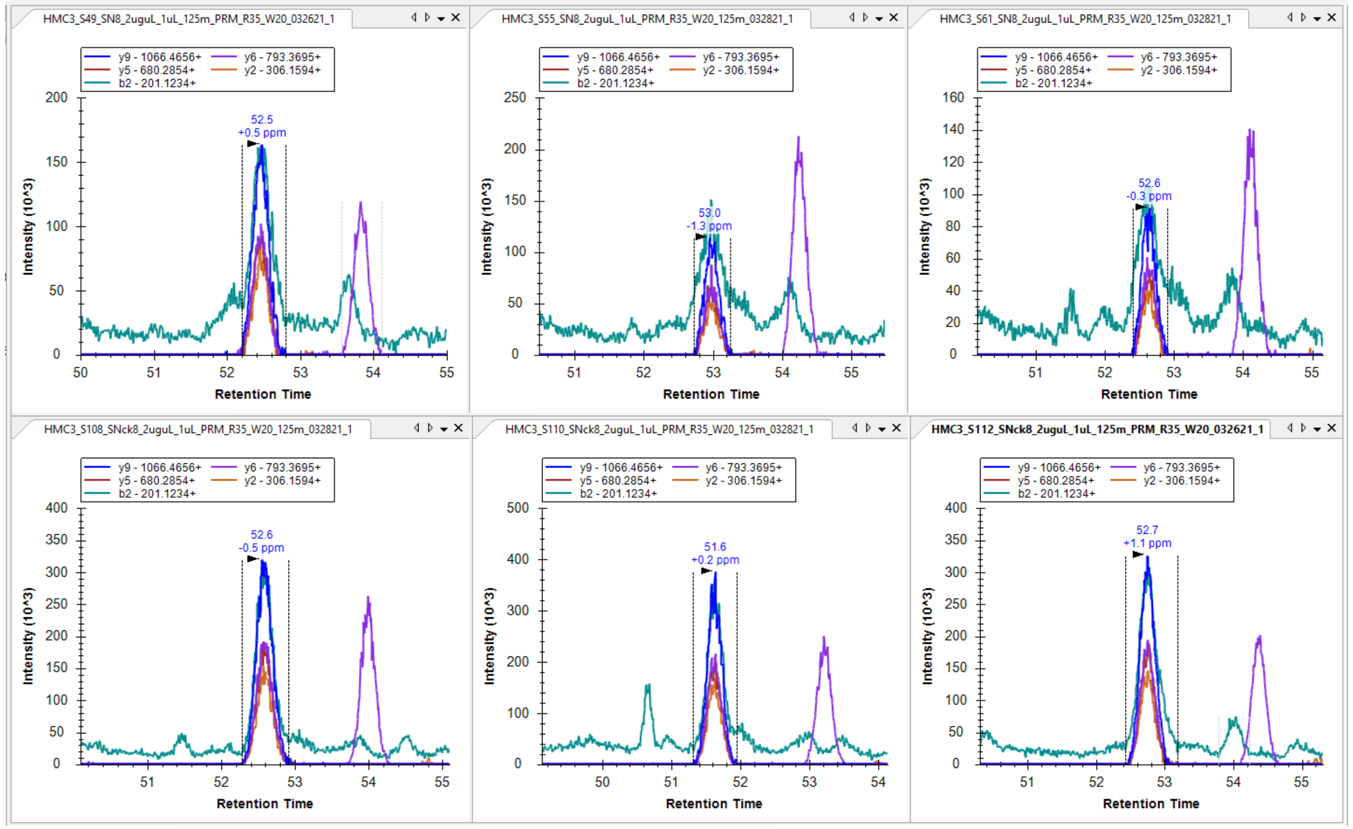

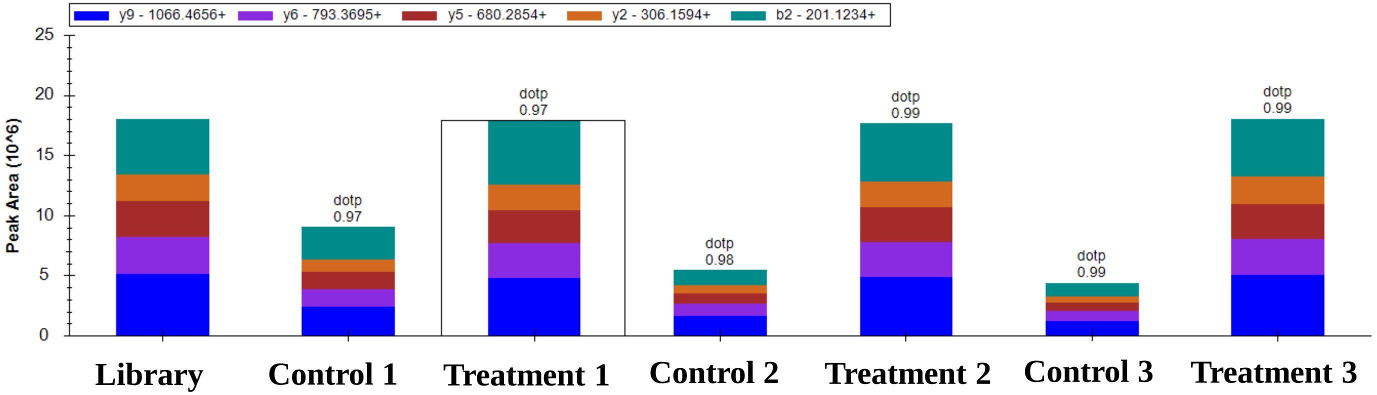

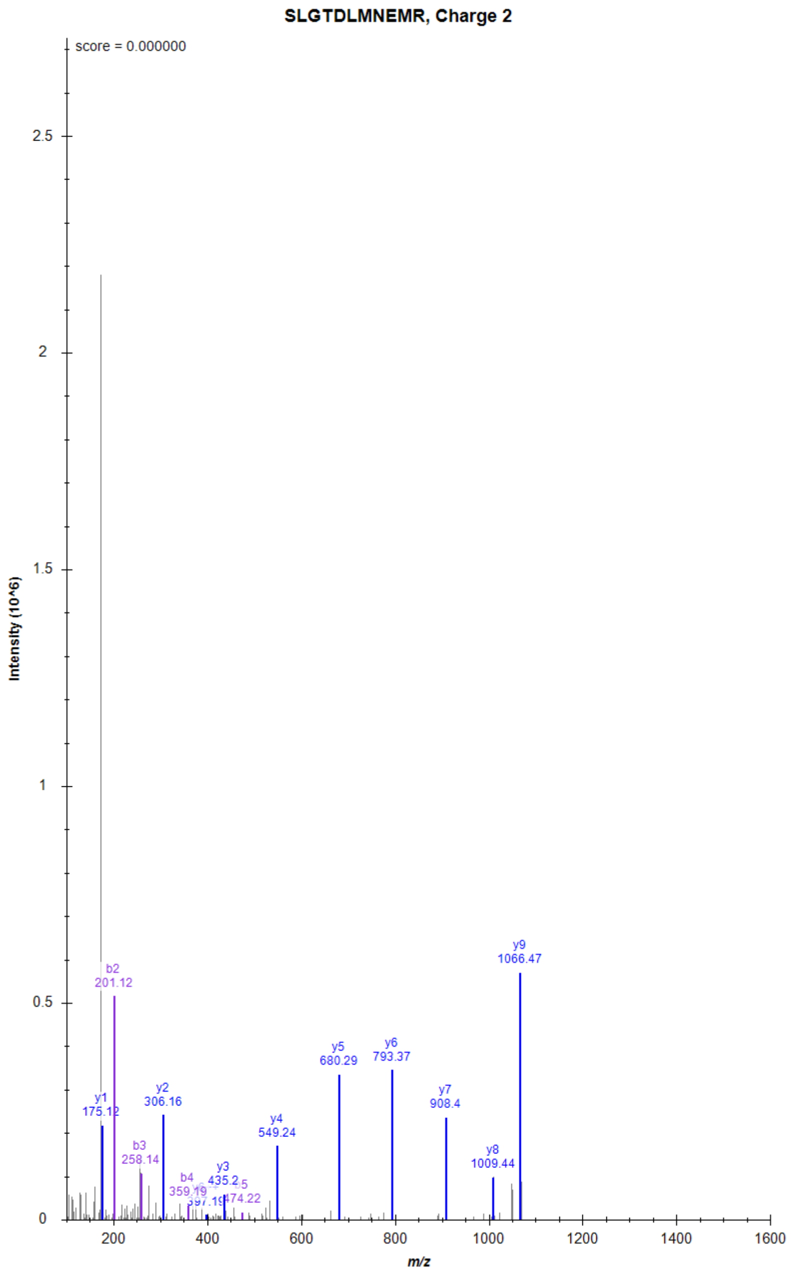

Integrin alpha-V (ITGAV)

SSASFNVIEFPYK, Charge 2, m/z 744.87372

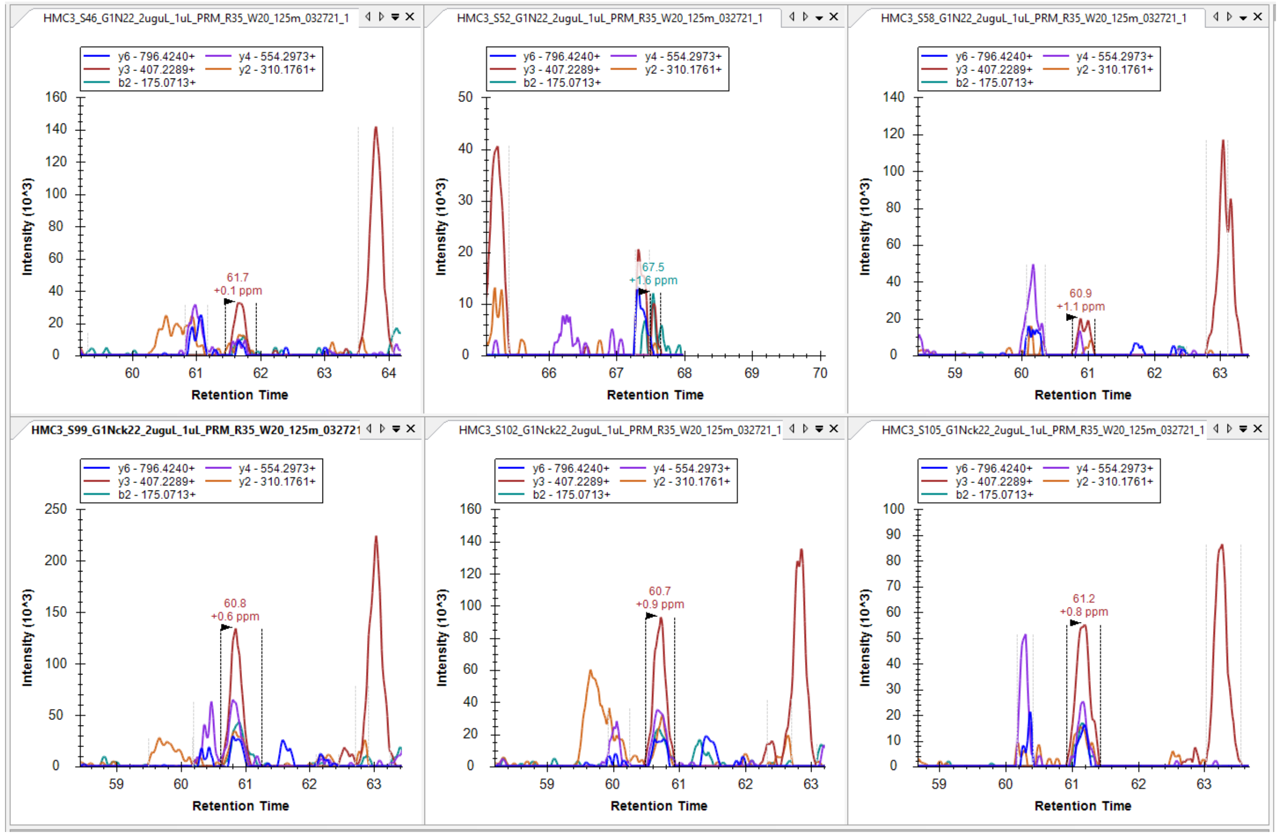

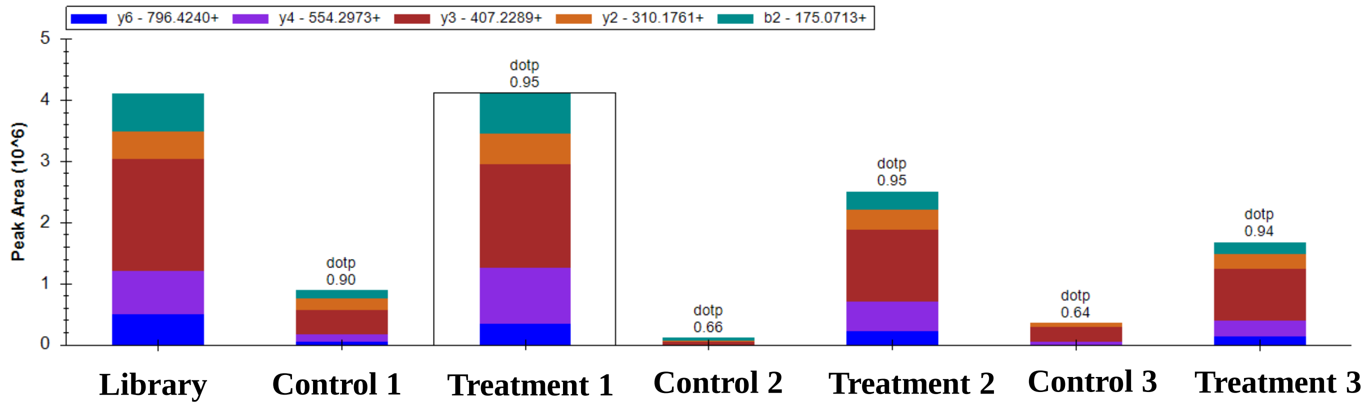

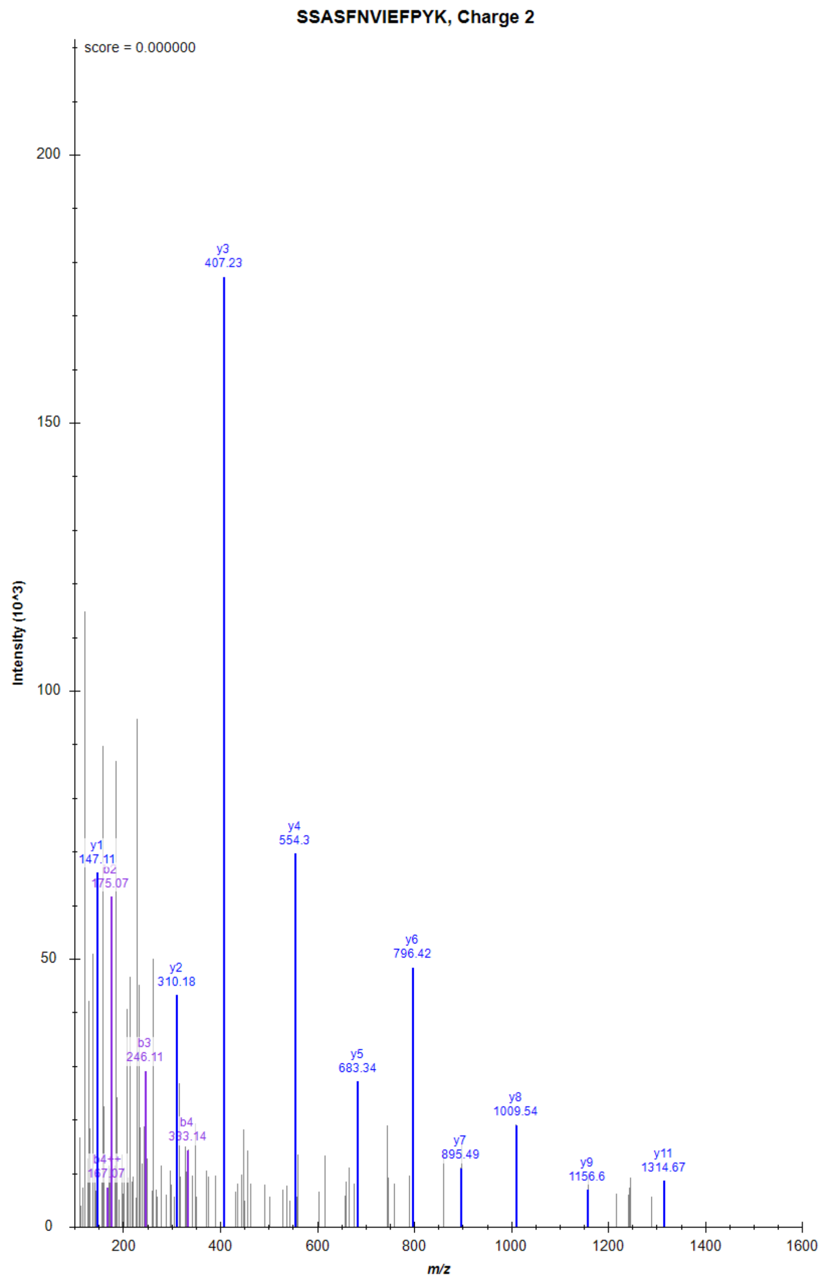

Integrin alpha-V (ITGAV)

LTPITIFMEYR, Charge 2, m/z 692.37231 (*20 min LC run*)

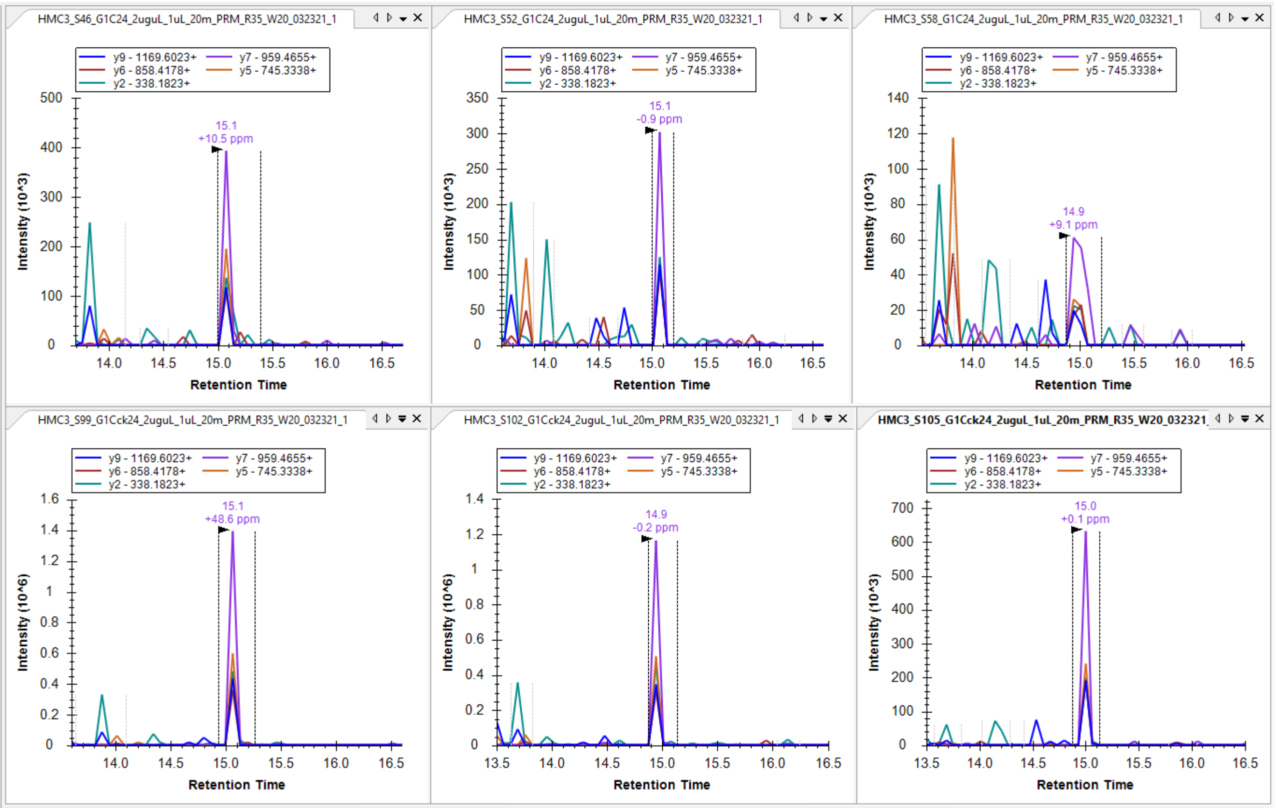

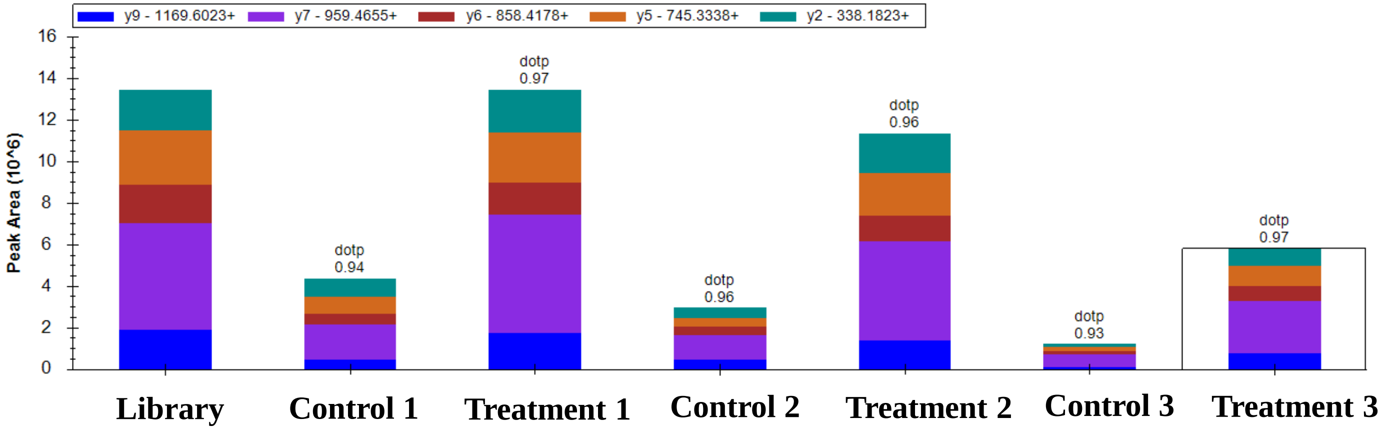

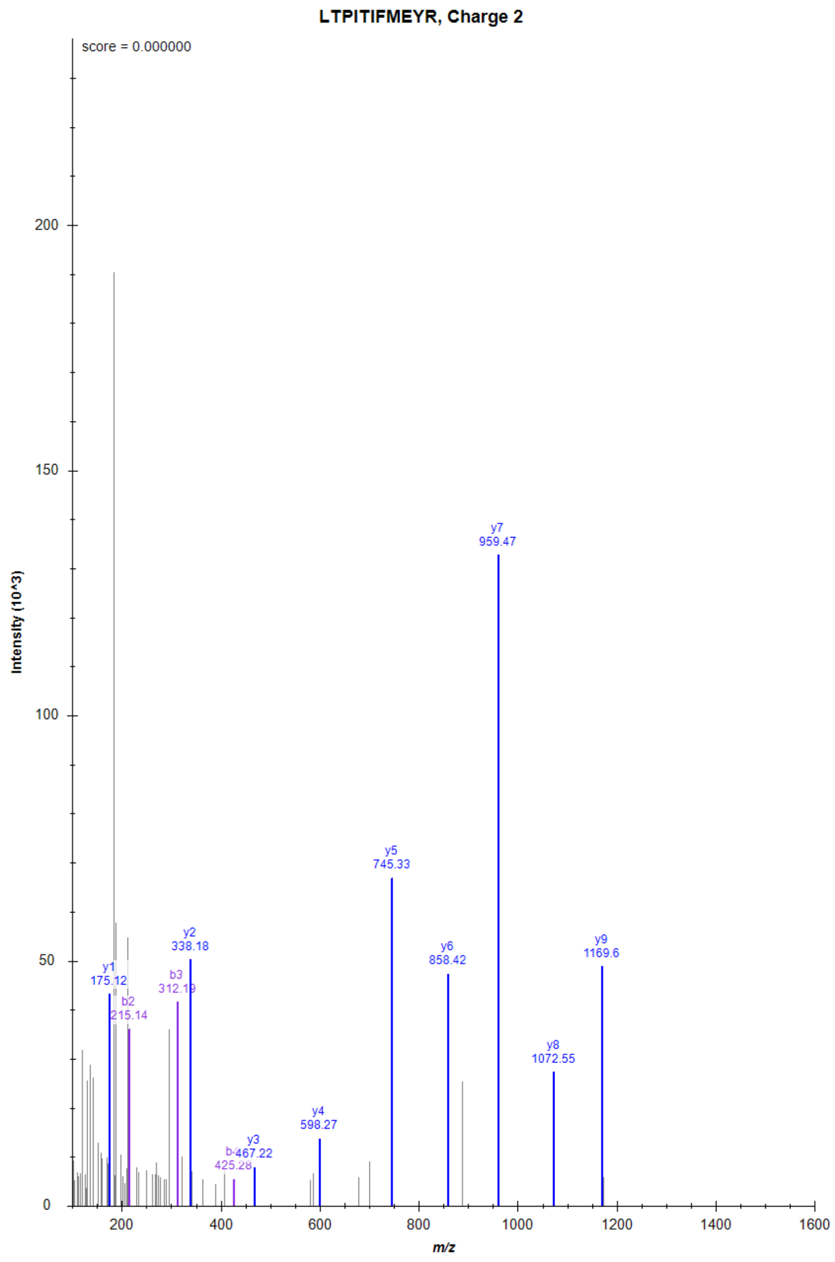

Hepatocyte growth factor receptor (MET)

DLIGFGLQVAK, Charge 2, m/z 580.83813

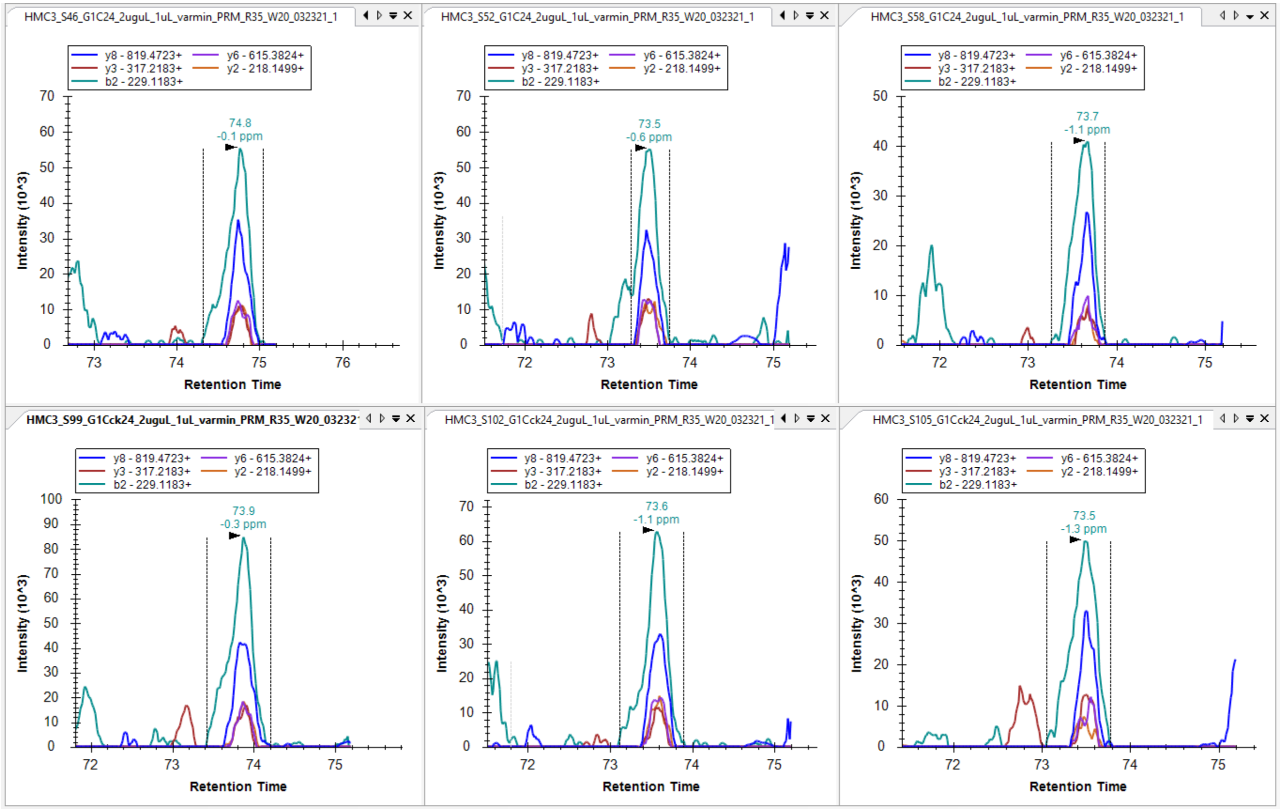

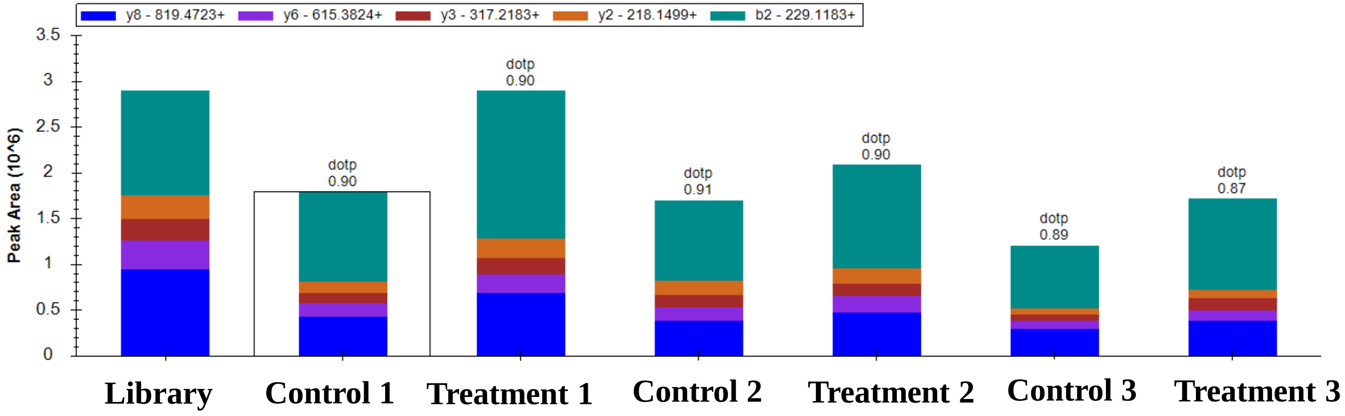

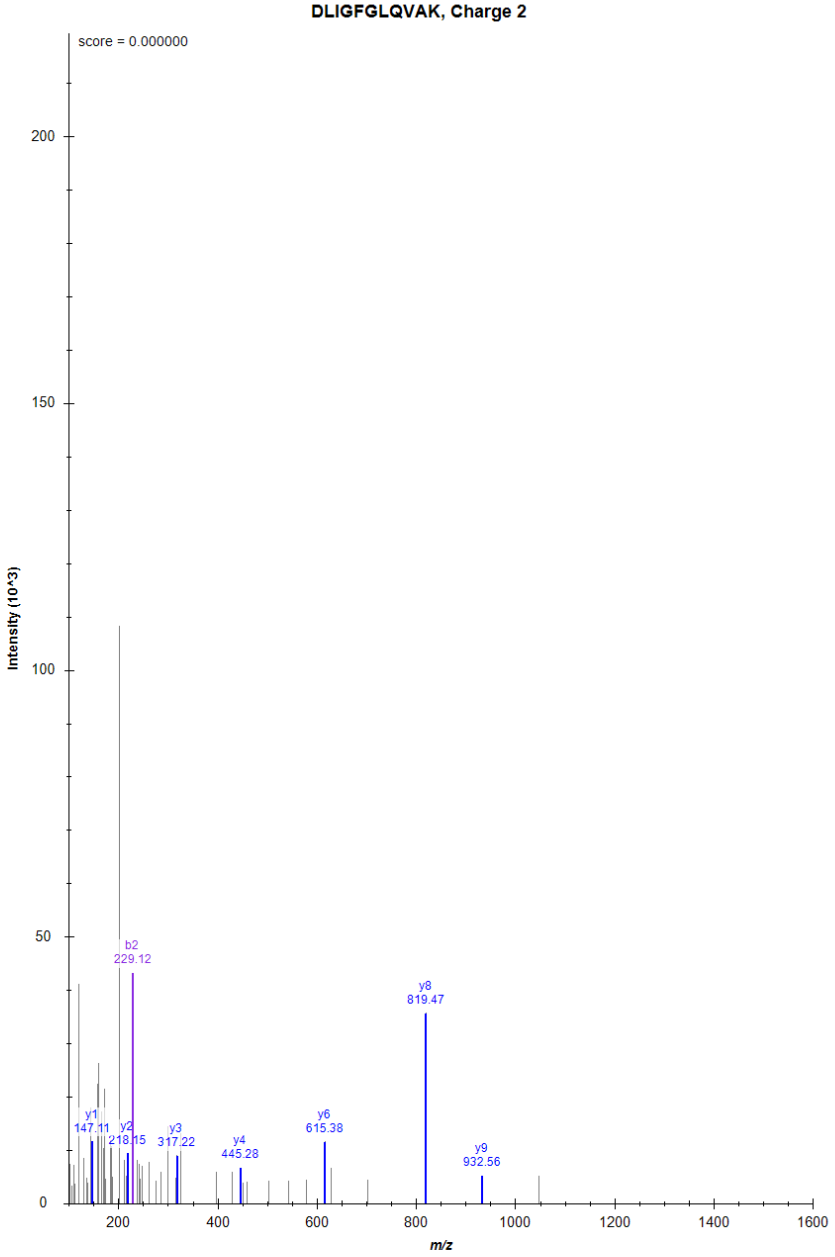

Category 3

Immune response related proteins

Complement component 1 Q subcomponent-binding protein, mitochondrial (C1QBP)

AFVDFLSDEIKEER, Charge 2, m/z 849.42389

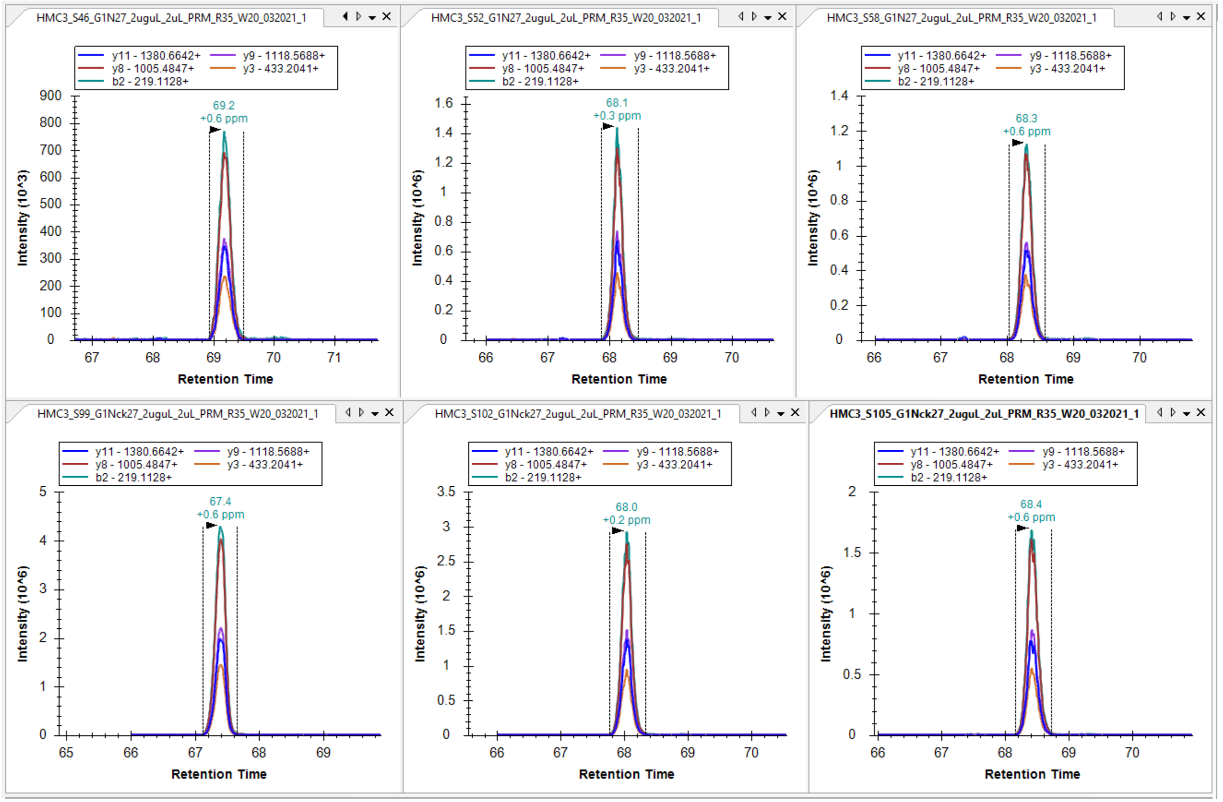

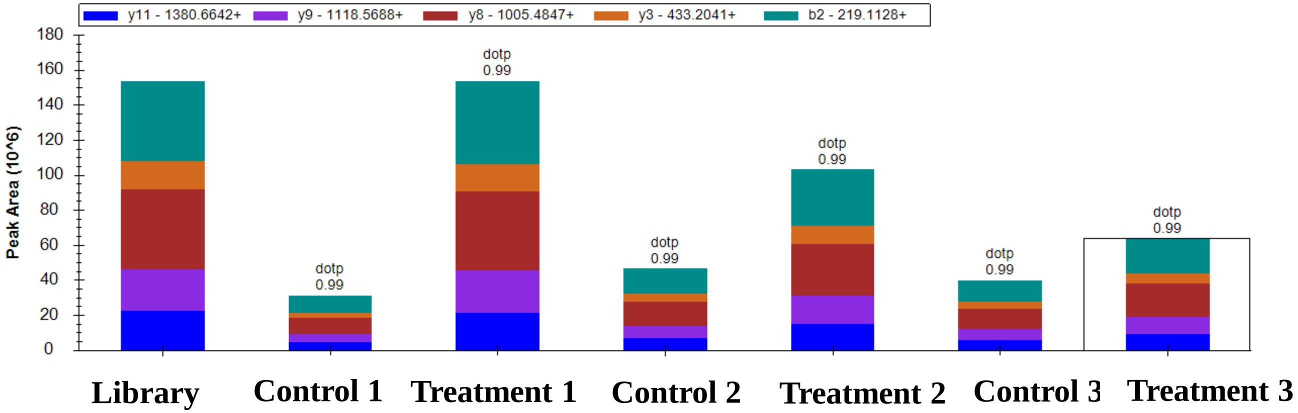

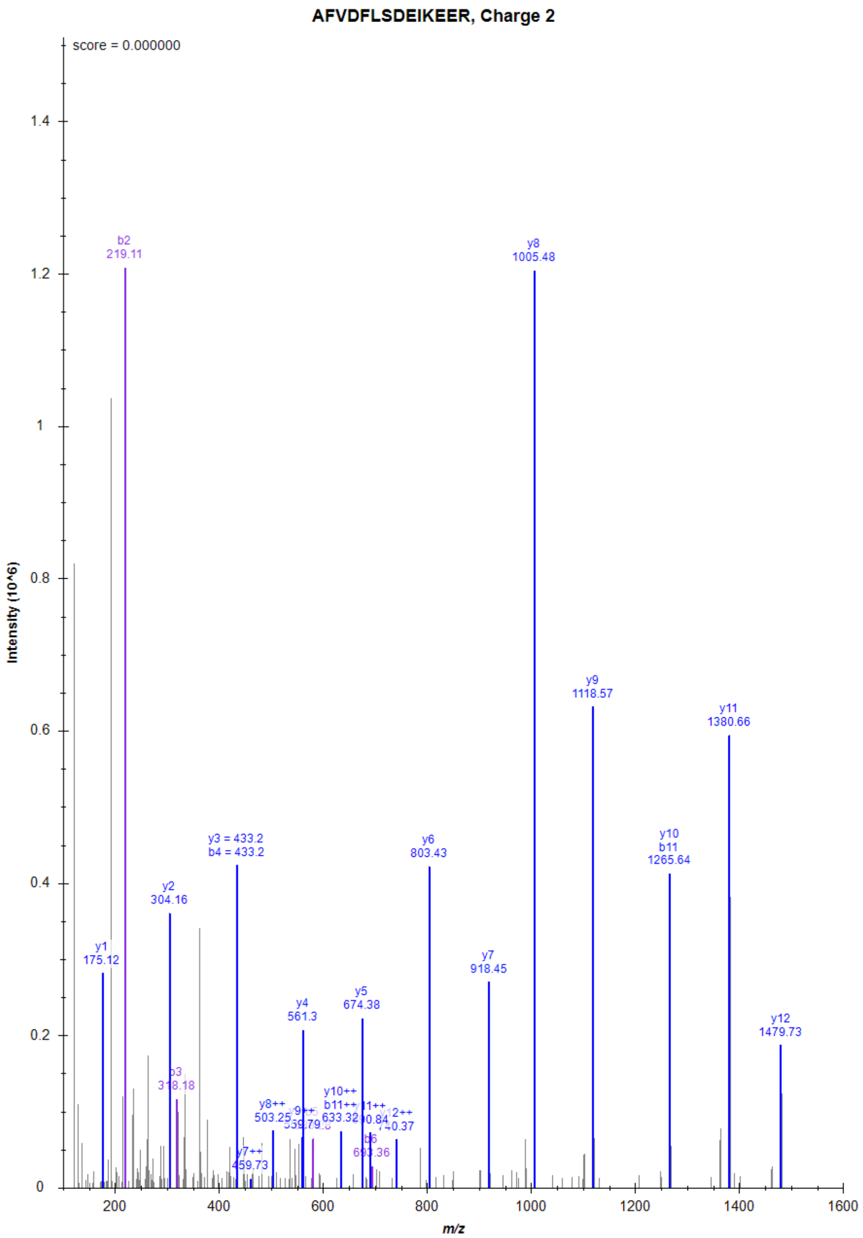

Complement component 1 Q subcomponent-binding protein, mitochondrial (C1QBP)

VEEQEPELTSTPNFVVEVIK, Charge 2, m/z 1144.08887

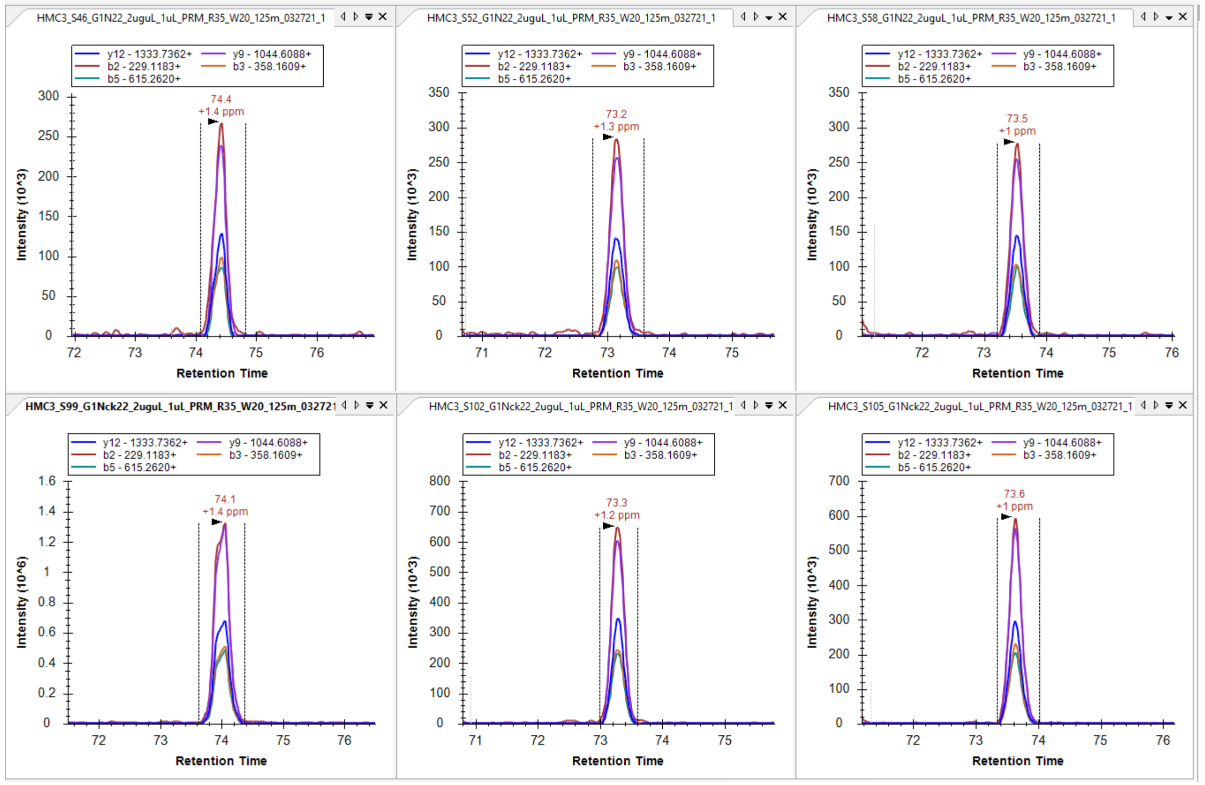

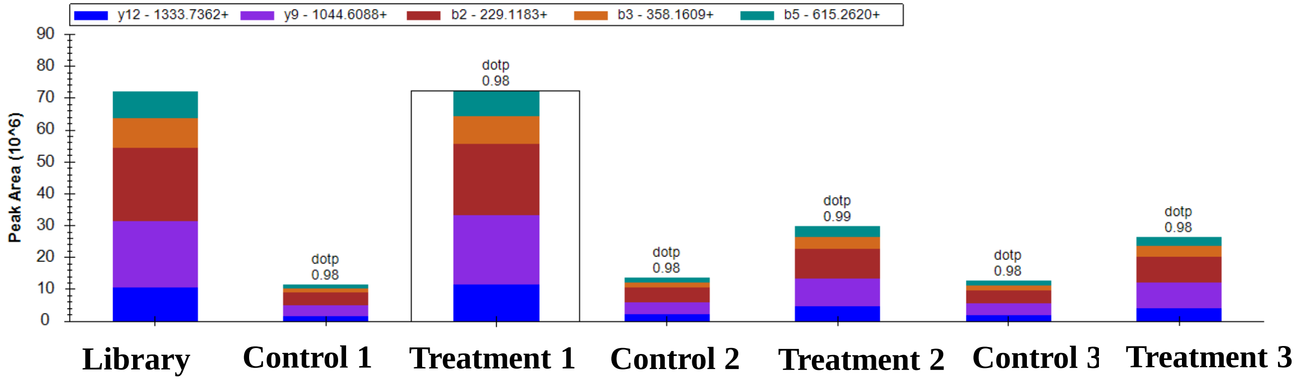

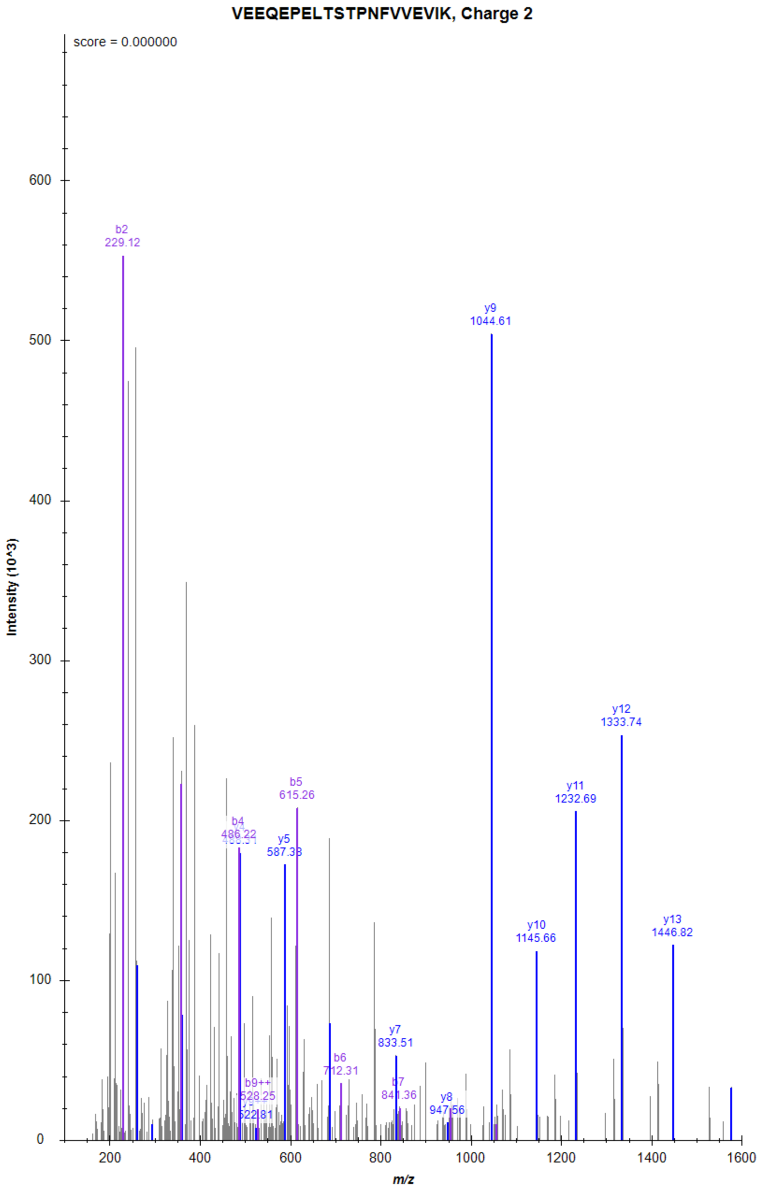

Complement component 1 Q subcomponent-binding protein, mitochondrial (C1QBP)

MSGGWELELNGTEAK, Charge 2, m/z 811.38141

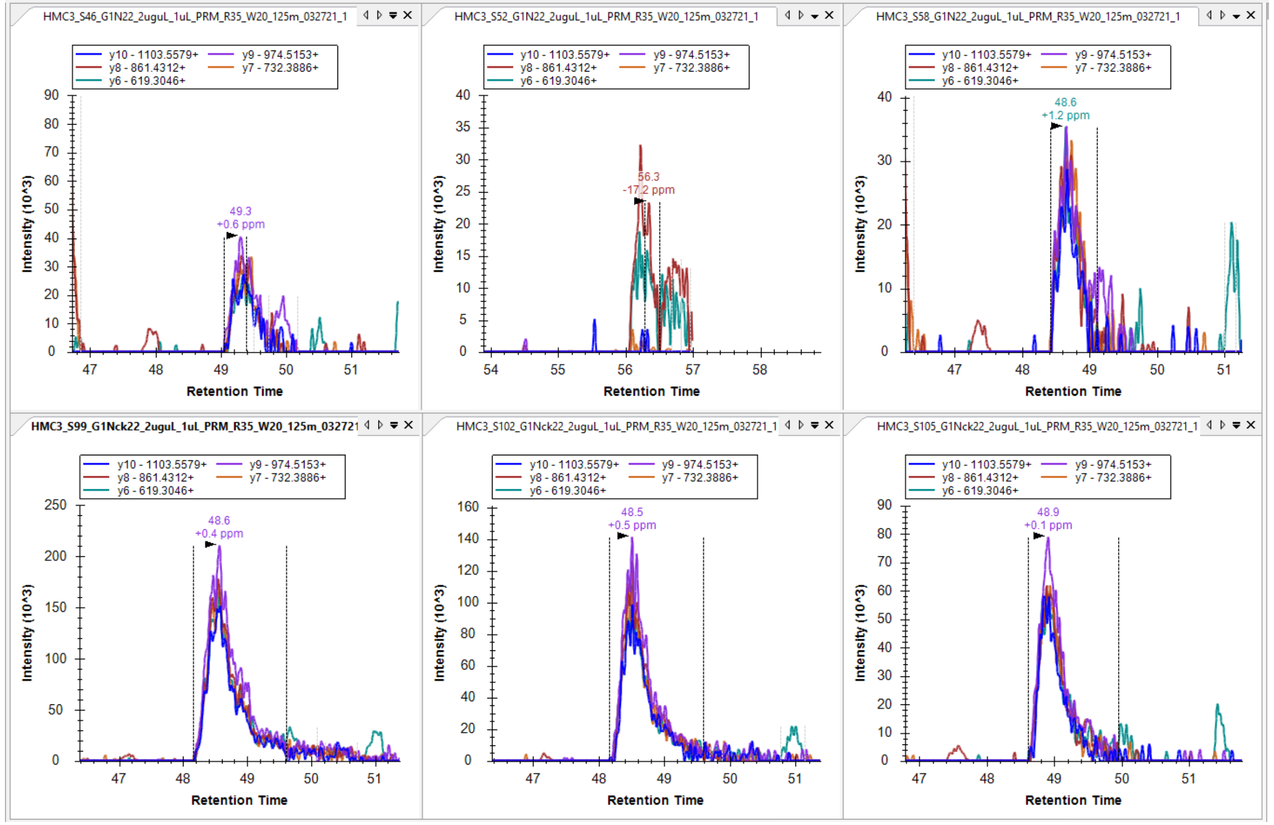

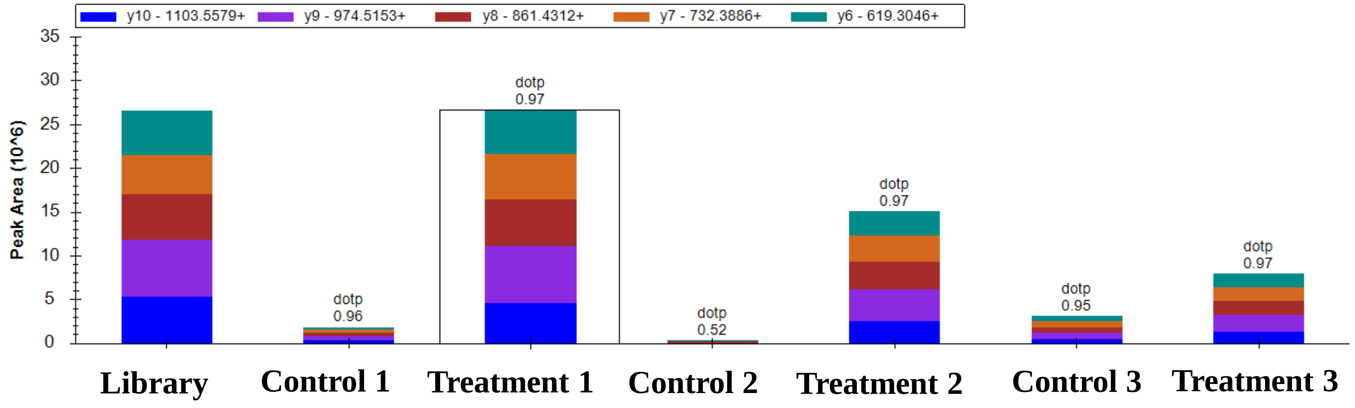

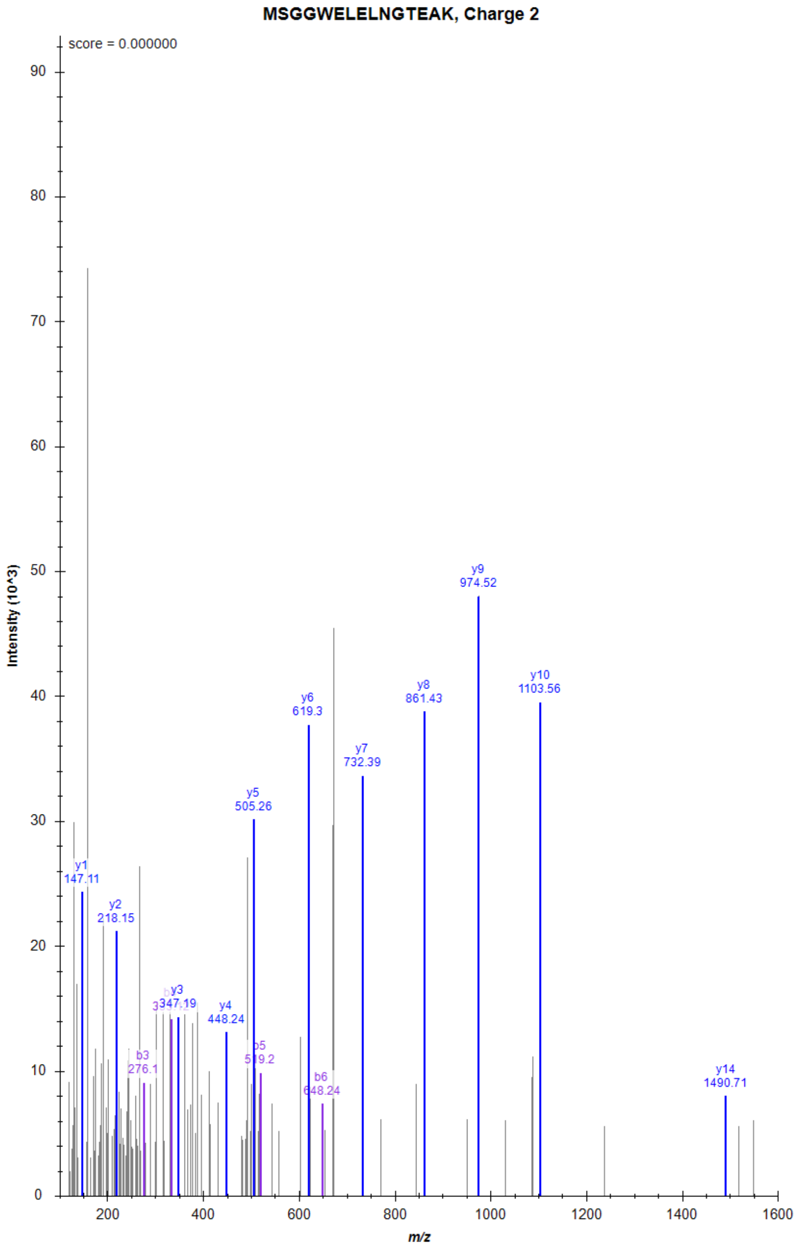

Nuclear factor NF-kappa-B p105 subunit (NFKB1)

GYNPGLLVHPDLAYLQAEGGGDR, Charge 3, m/z 804.73737

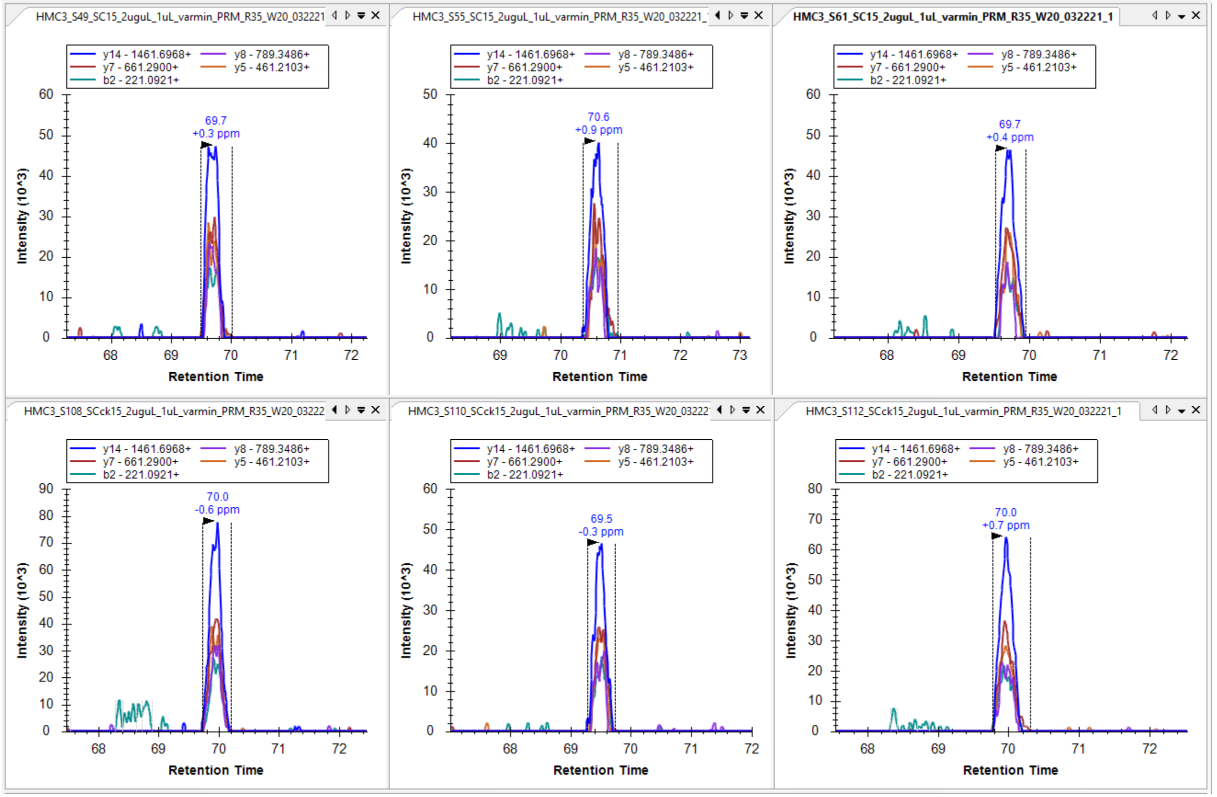

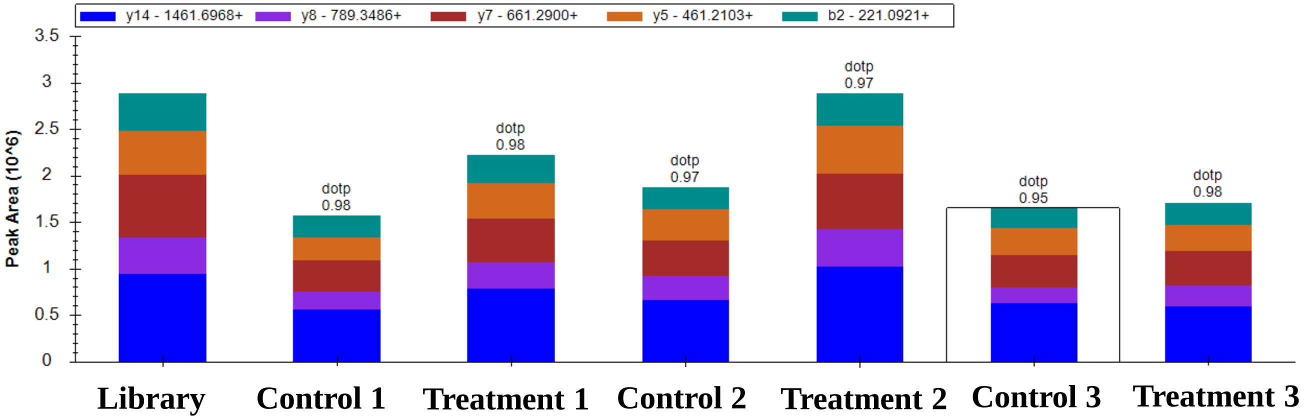

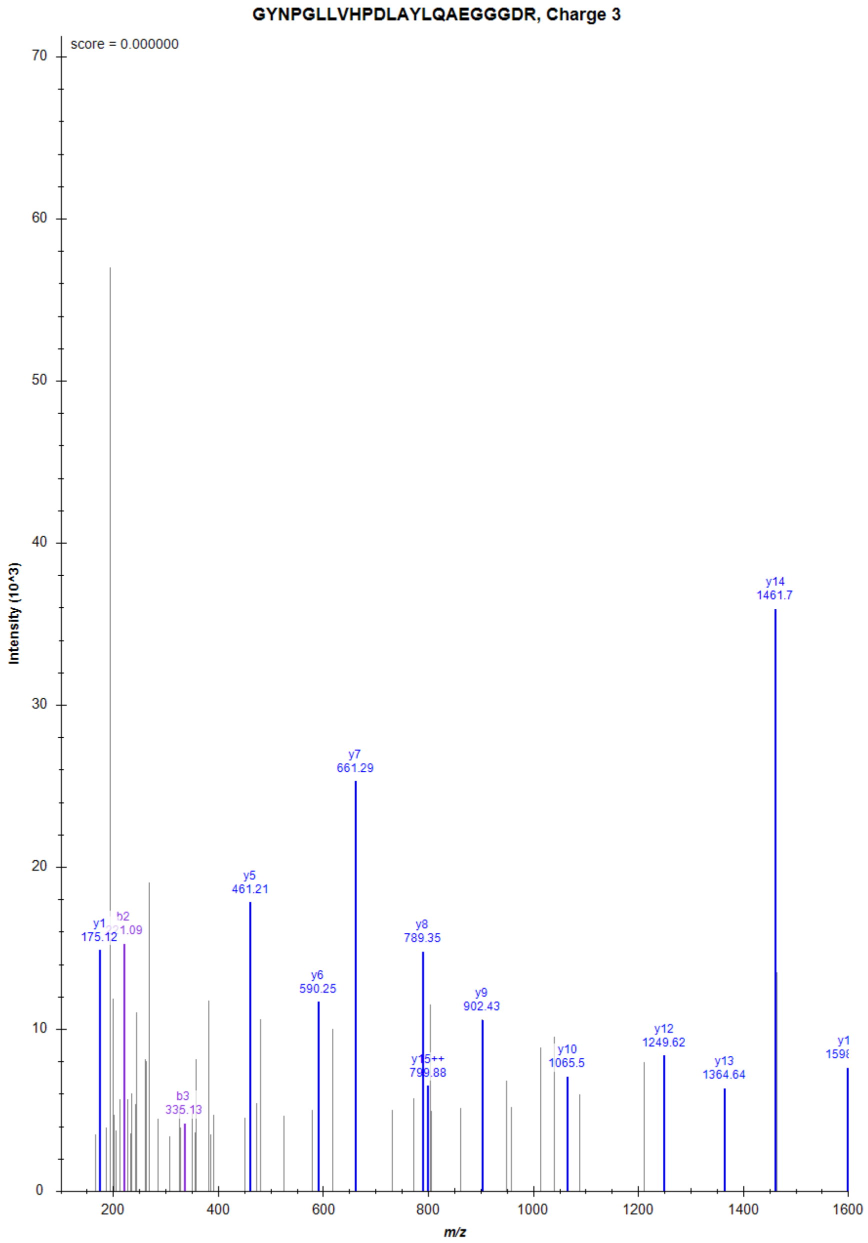

Nuclear factor NF-kappa-B p105 subunit (NFKB1)

LMFTAFLPDSTGSFTR, Charge 2, m/z 895.94464

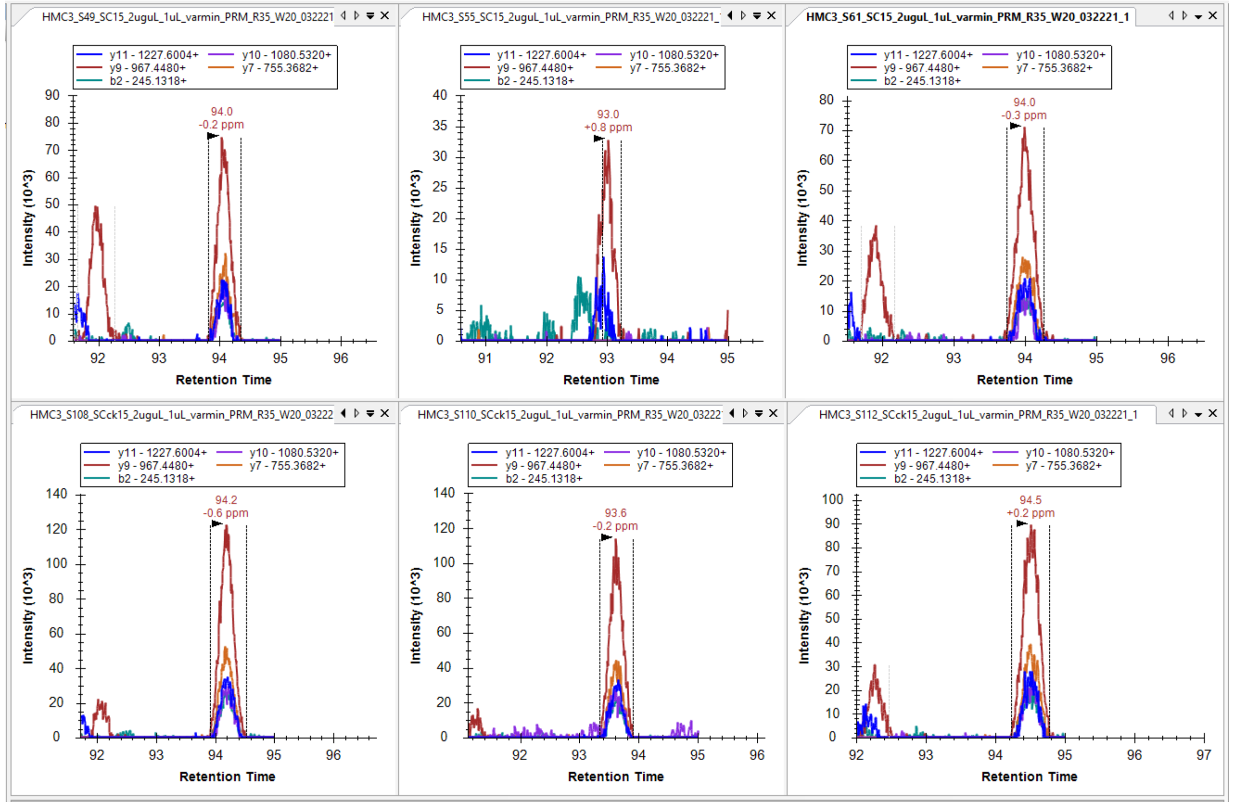

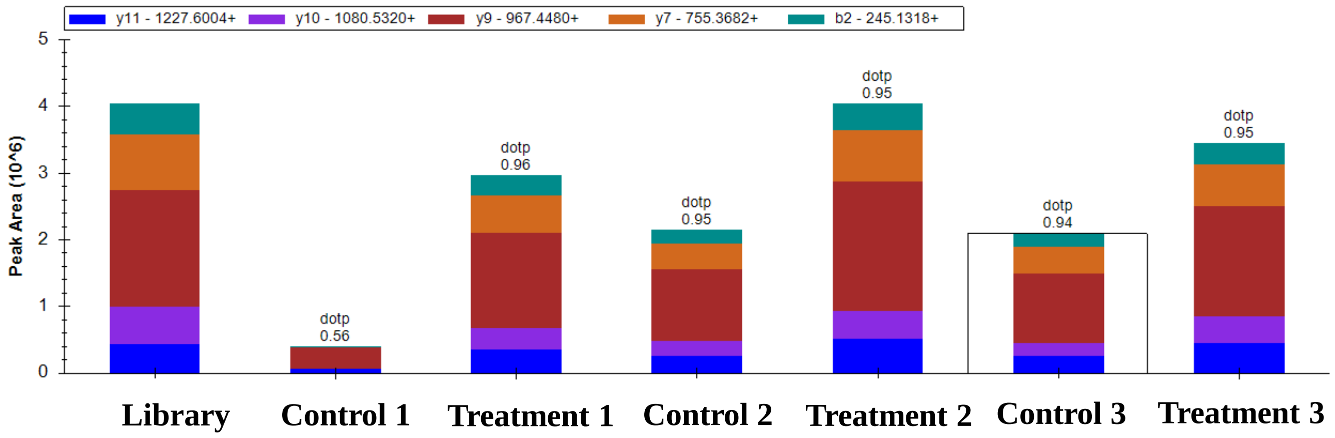

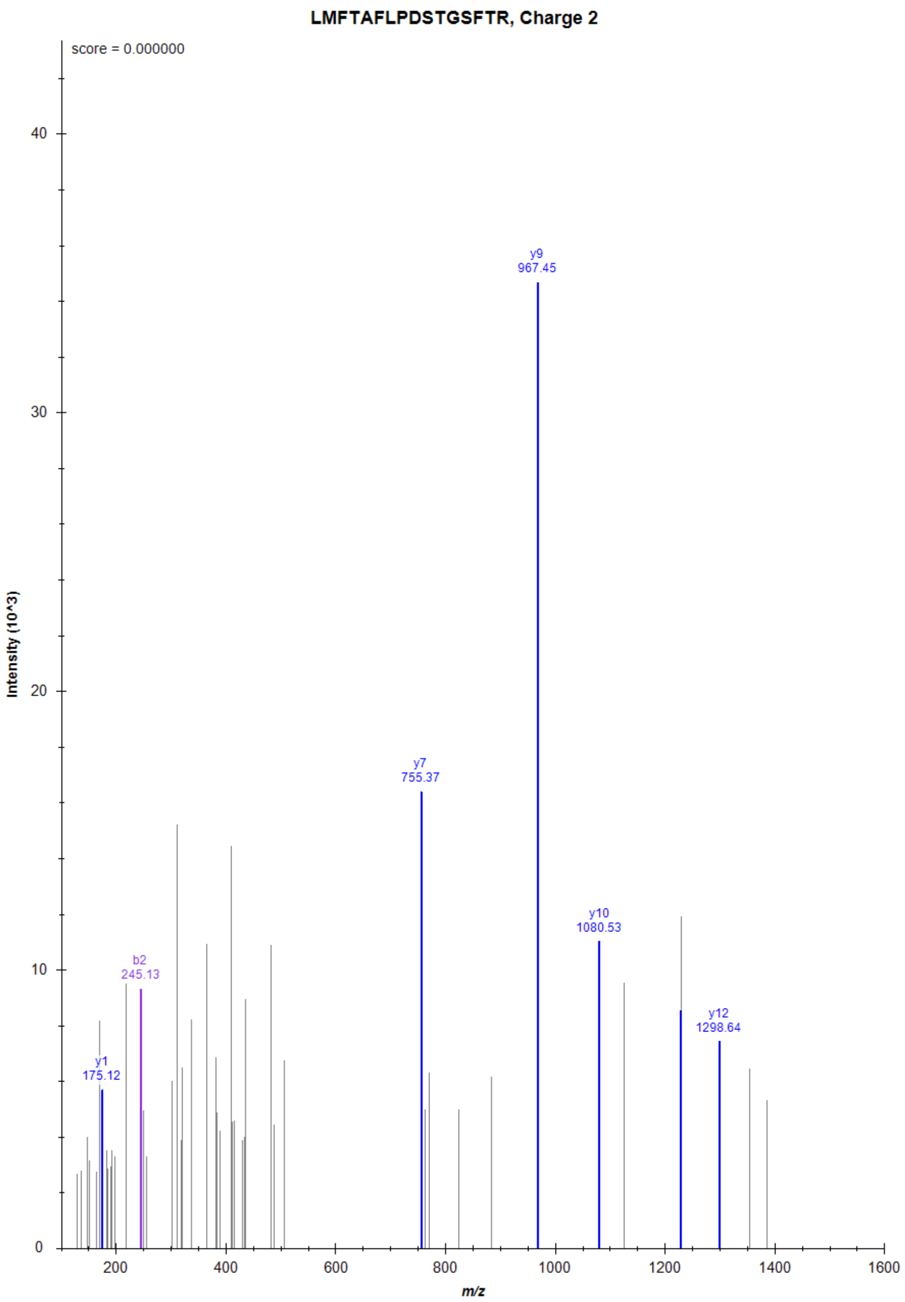

Signal transducer and activator of transcription 1-alpha/beta (STAT1)

FHDLLSQLDDQYSR, Charge 2, m/z 868.91266

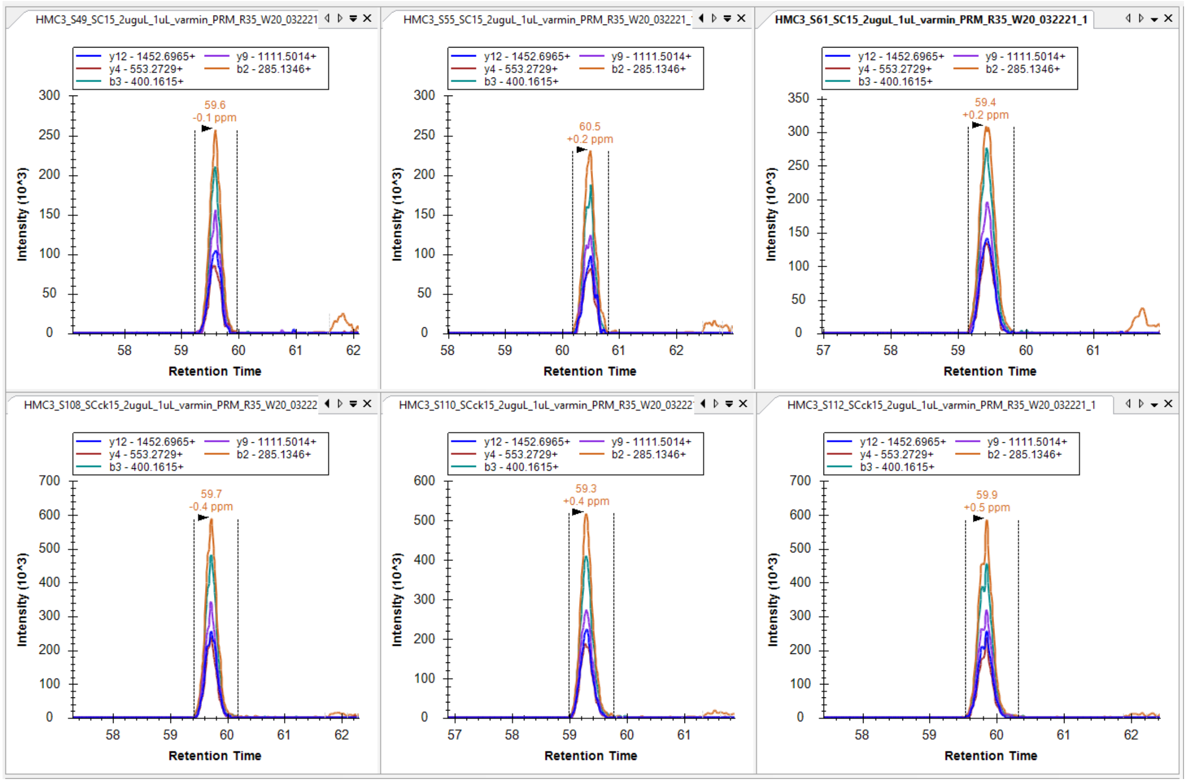

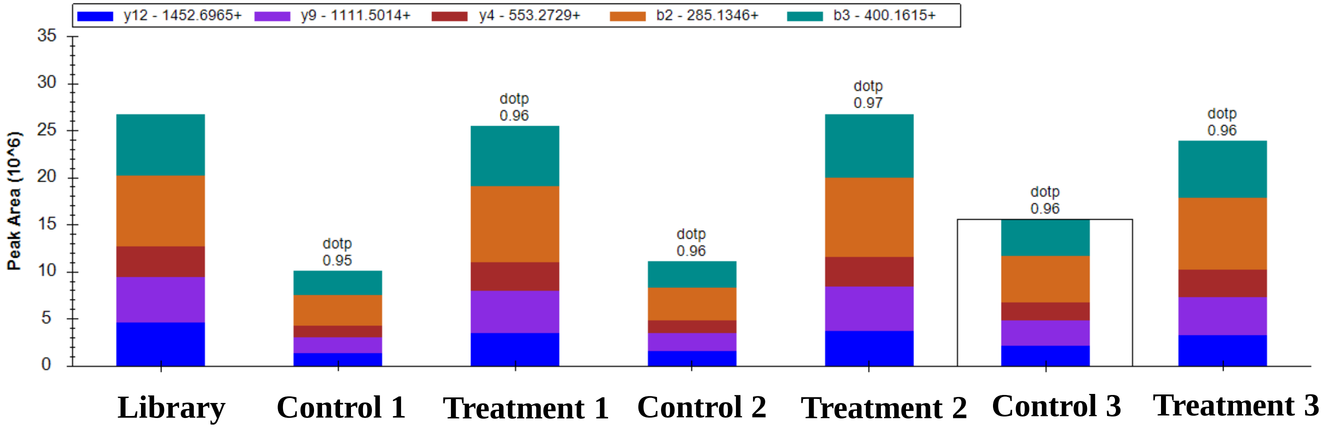

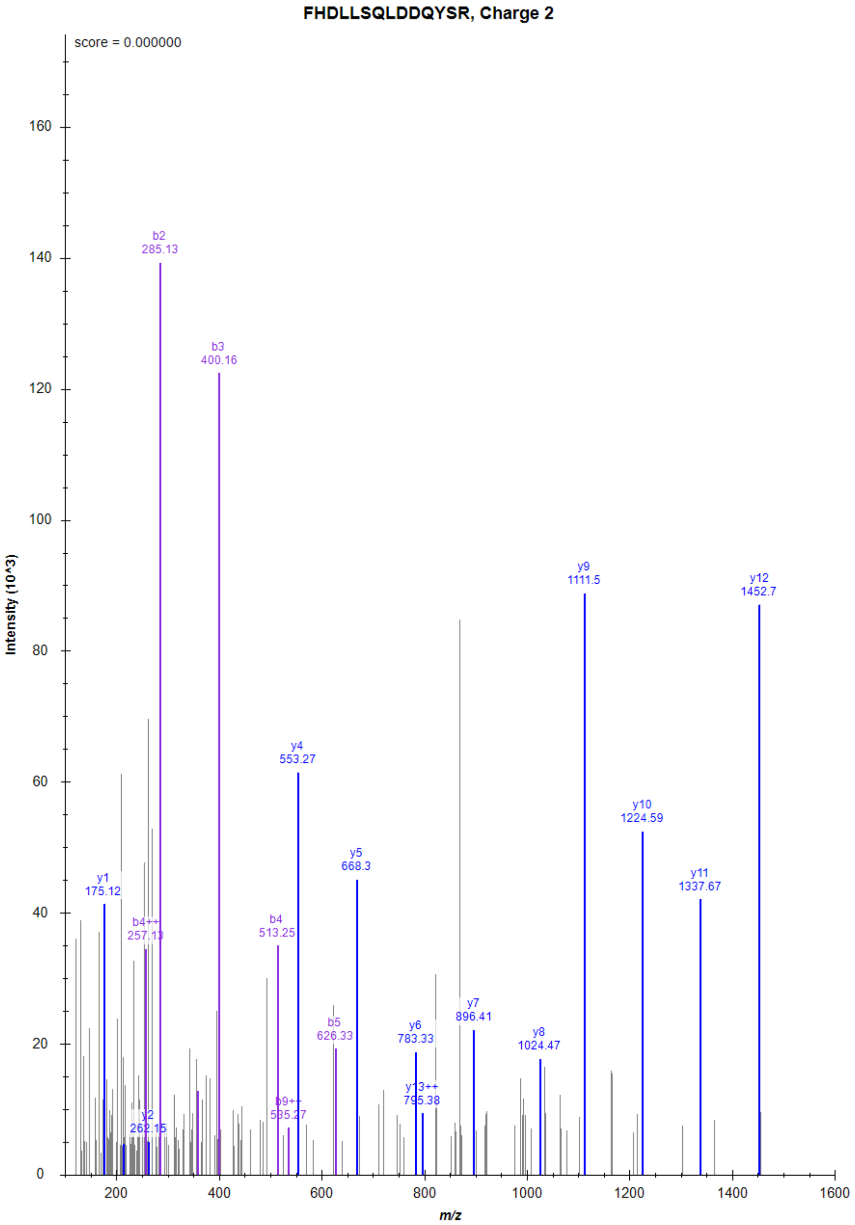

Signal transducer and activator of transcription 1-alpha/beta (STAT1)

LLGPNASPDGLIPWTR, Charge 2, m/z 853.96716

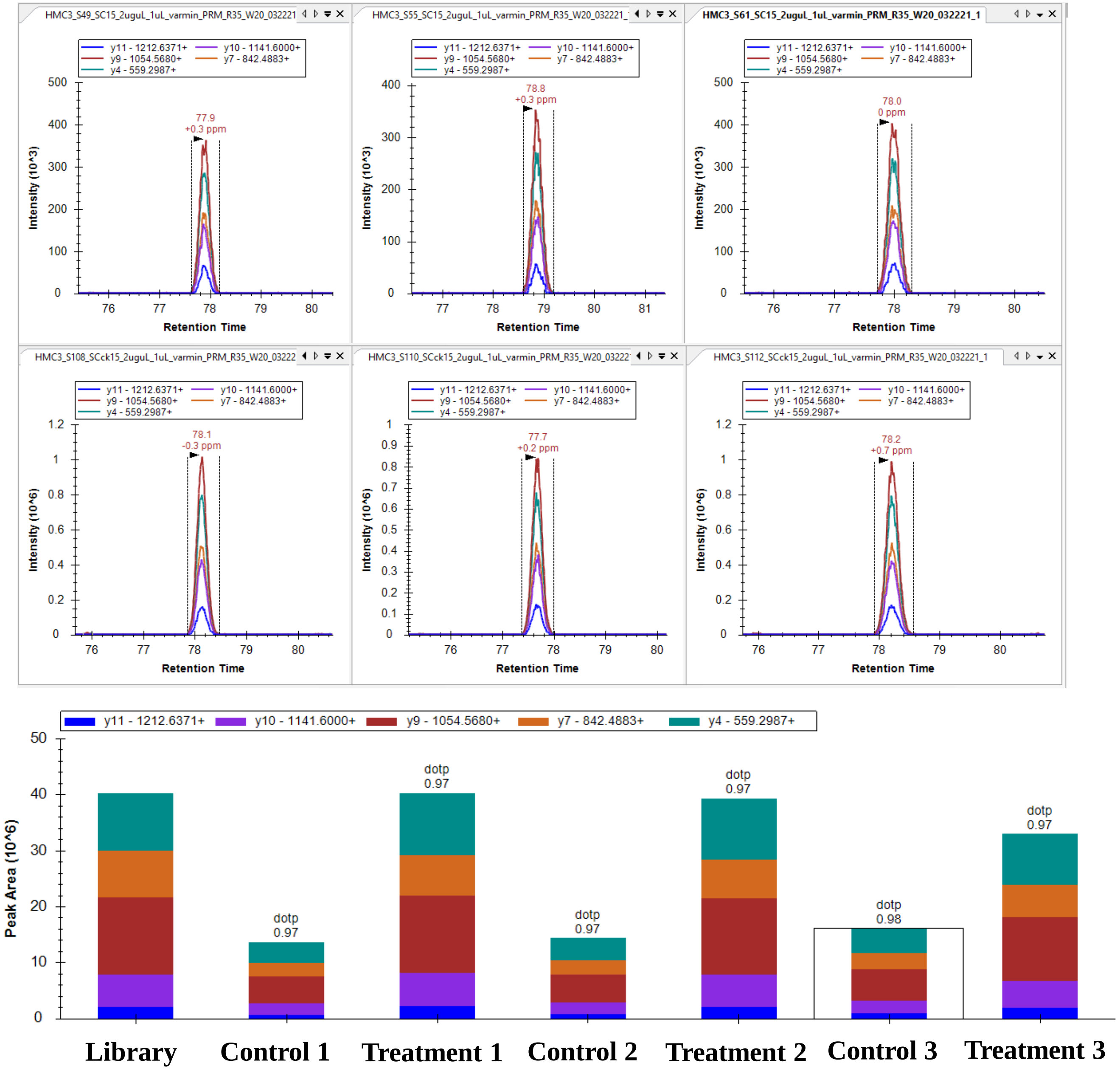

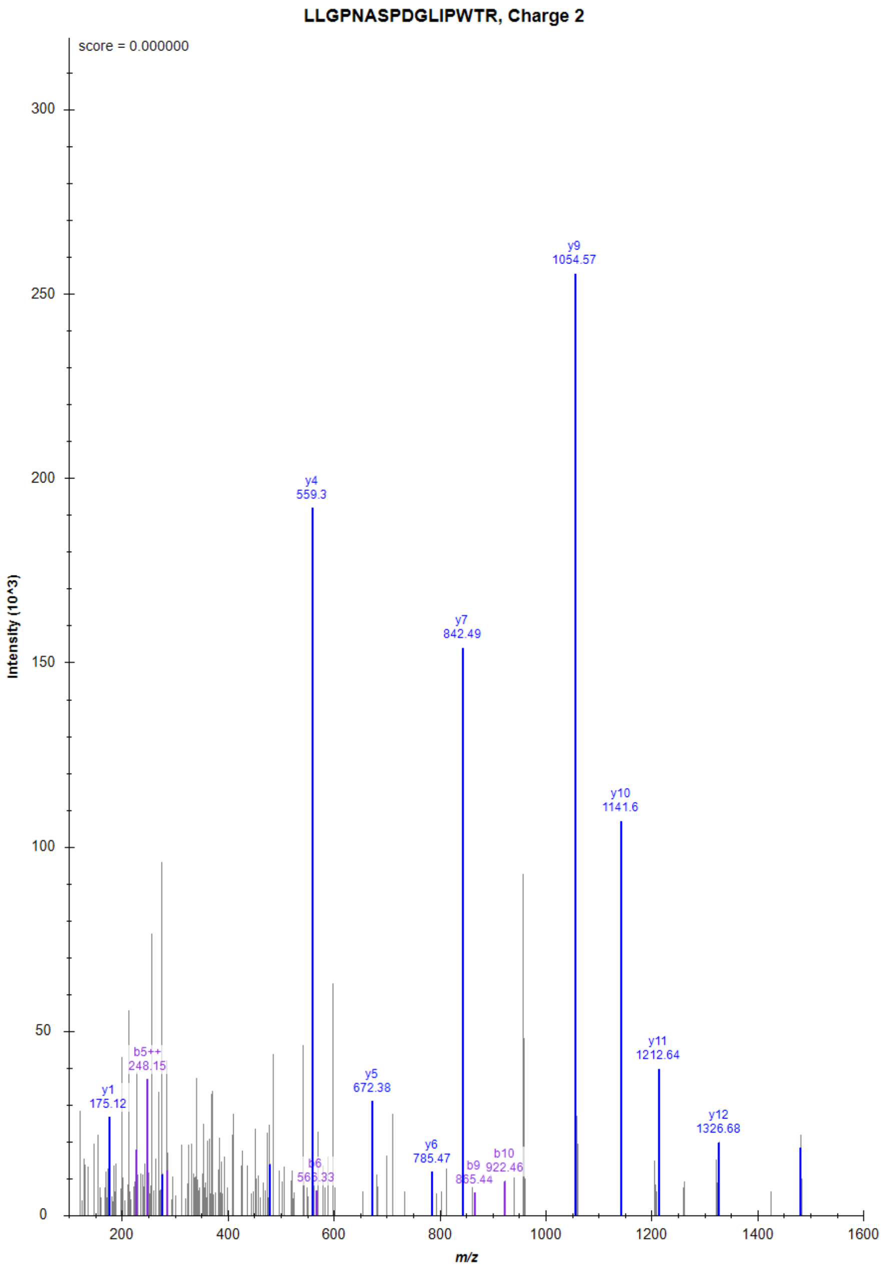

Ubiquitin-like protein ISG15 (ISG15)

LAVHPSGVALQDR, Charge 3, m/z 454.9221

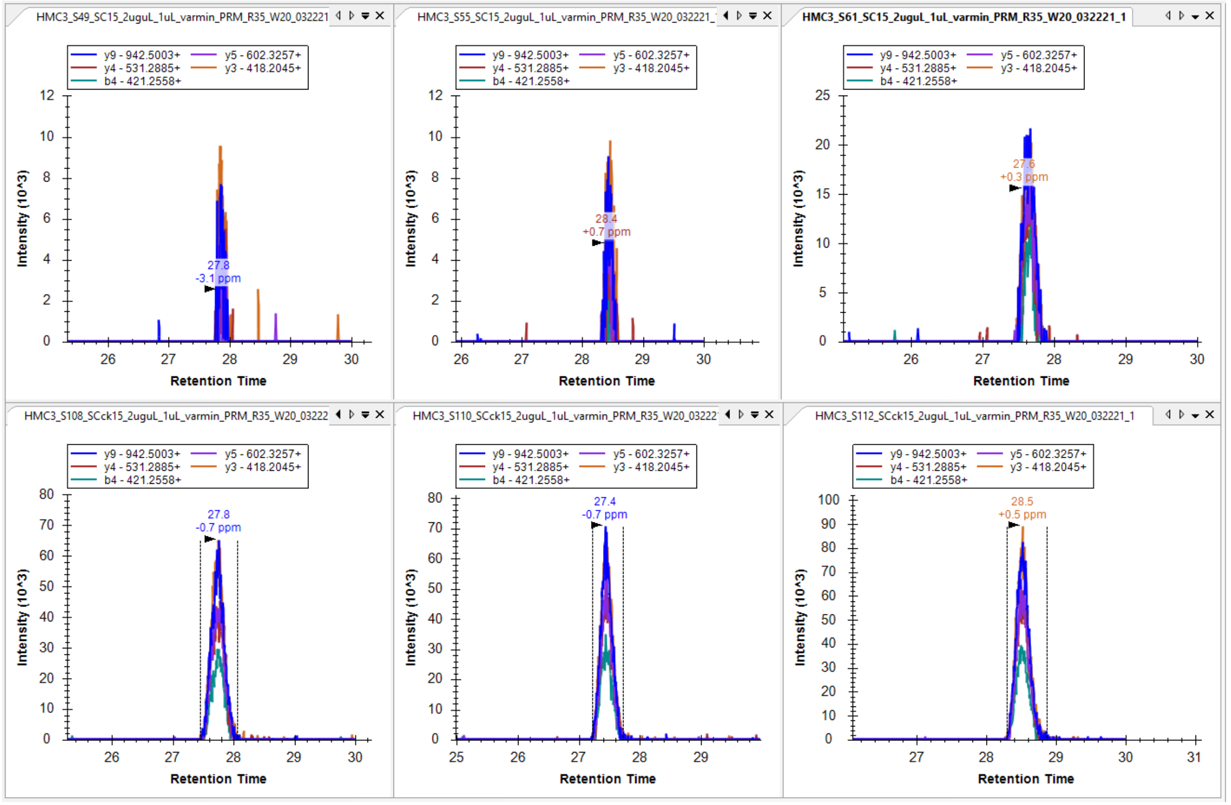

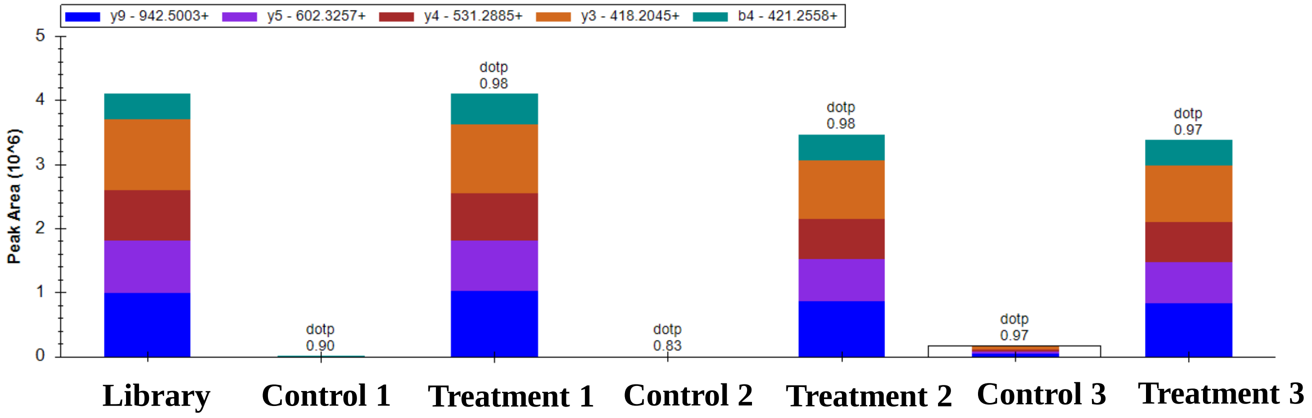

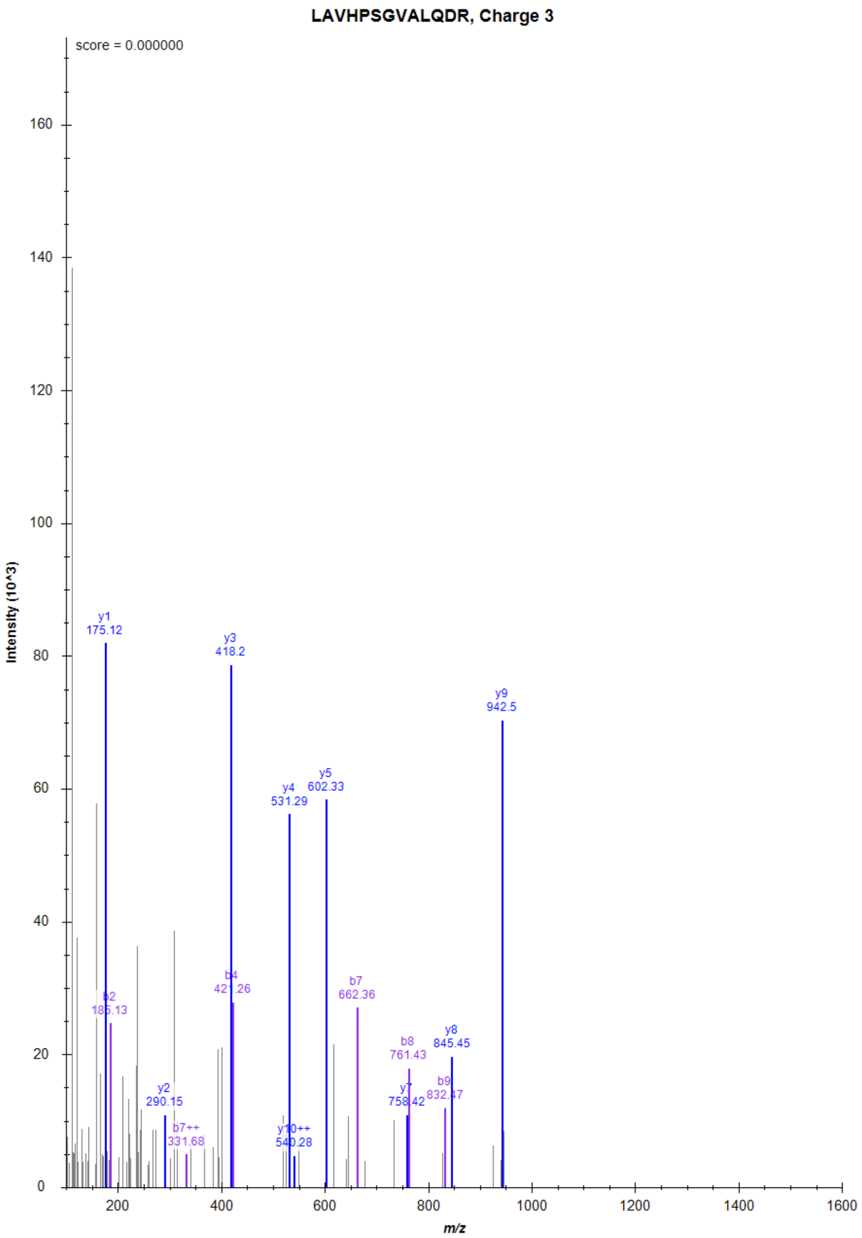

Ubiquitin-like protein ISG15 (ISG15)

VPLASQGLGPGSTVLLVVDK, Charge 3, m/z 650.7146 (*20 min LC run*)

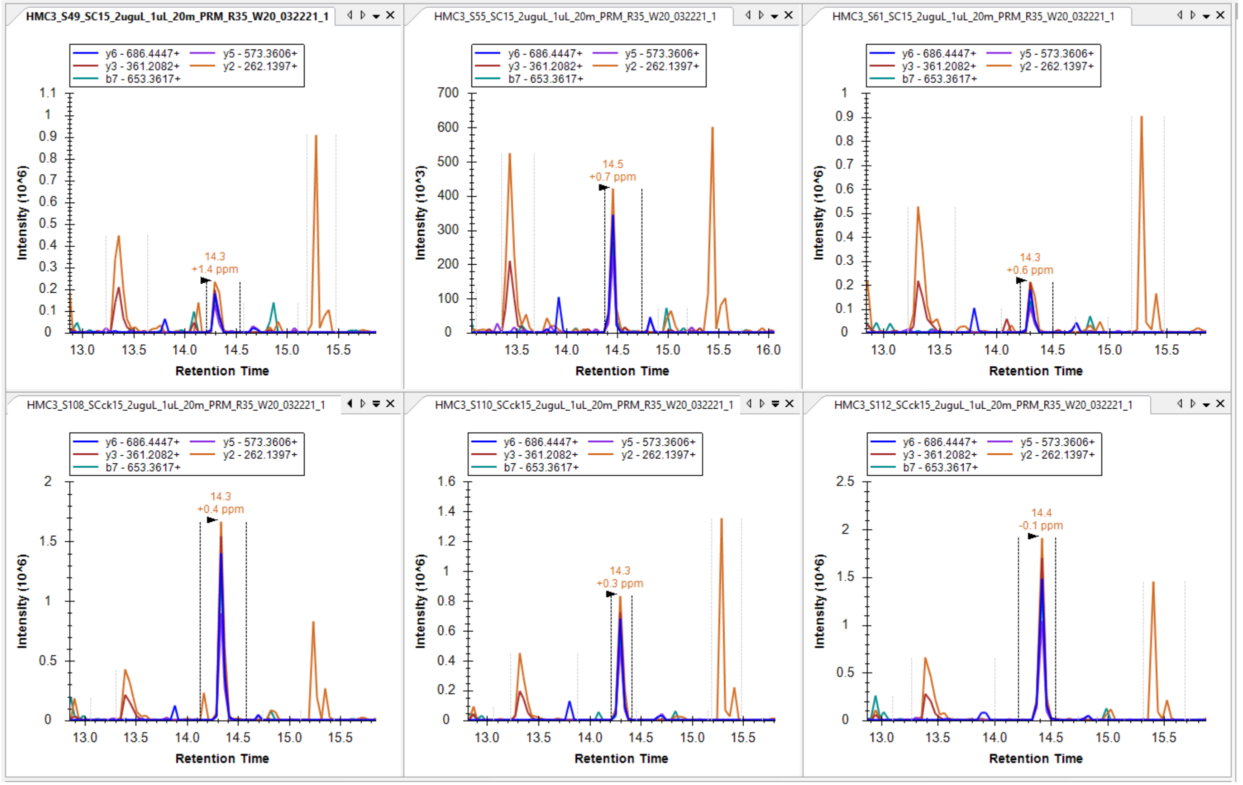

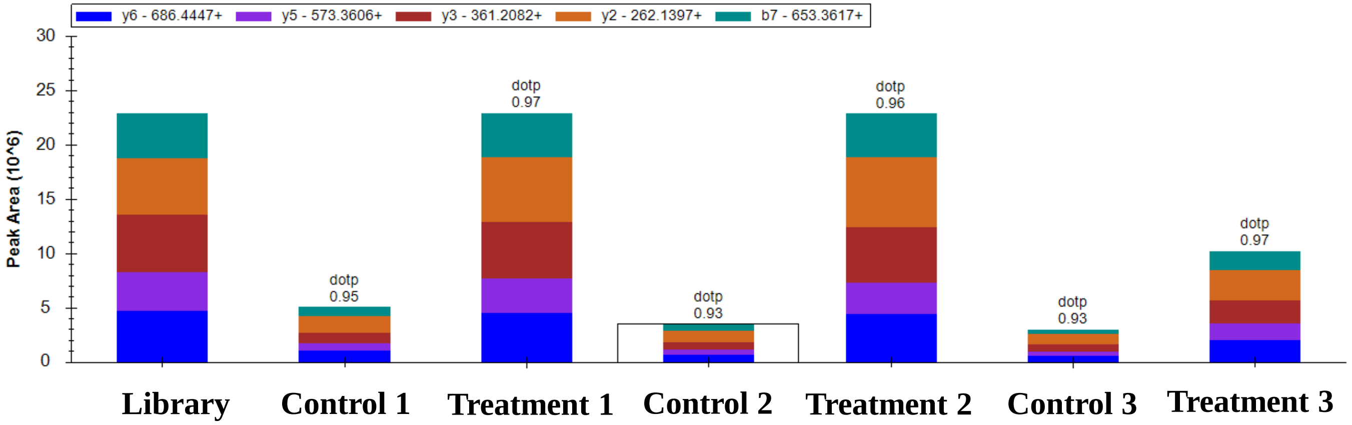

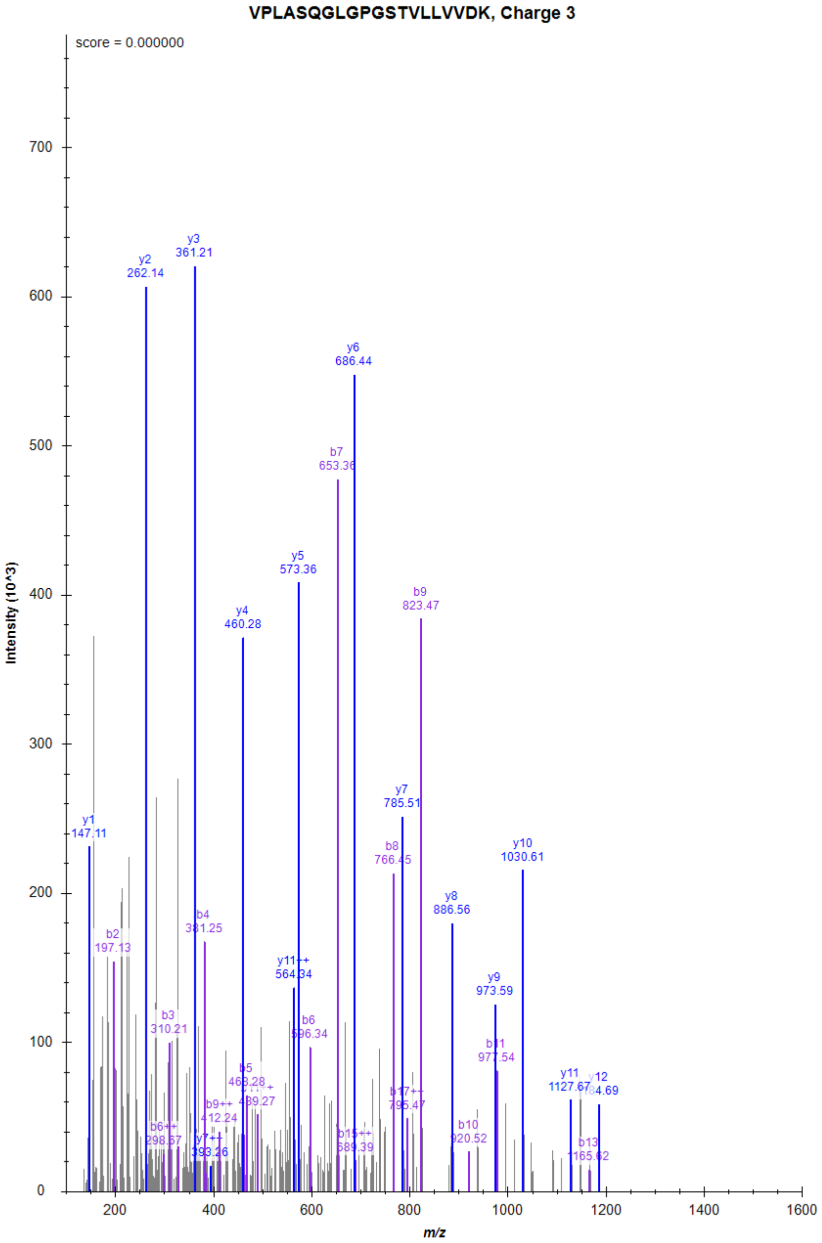

HLA class I histocompatibility antigen, Cw-8 (HLA-C)

WAAVVVPSGEEQR, Charge 2, m/z 714.36584

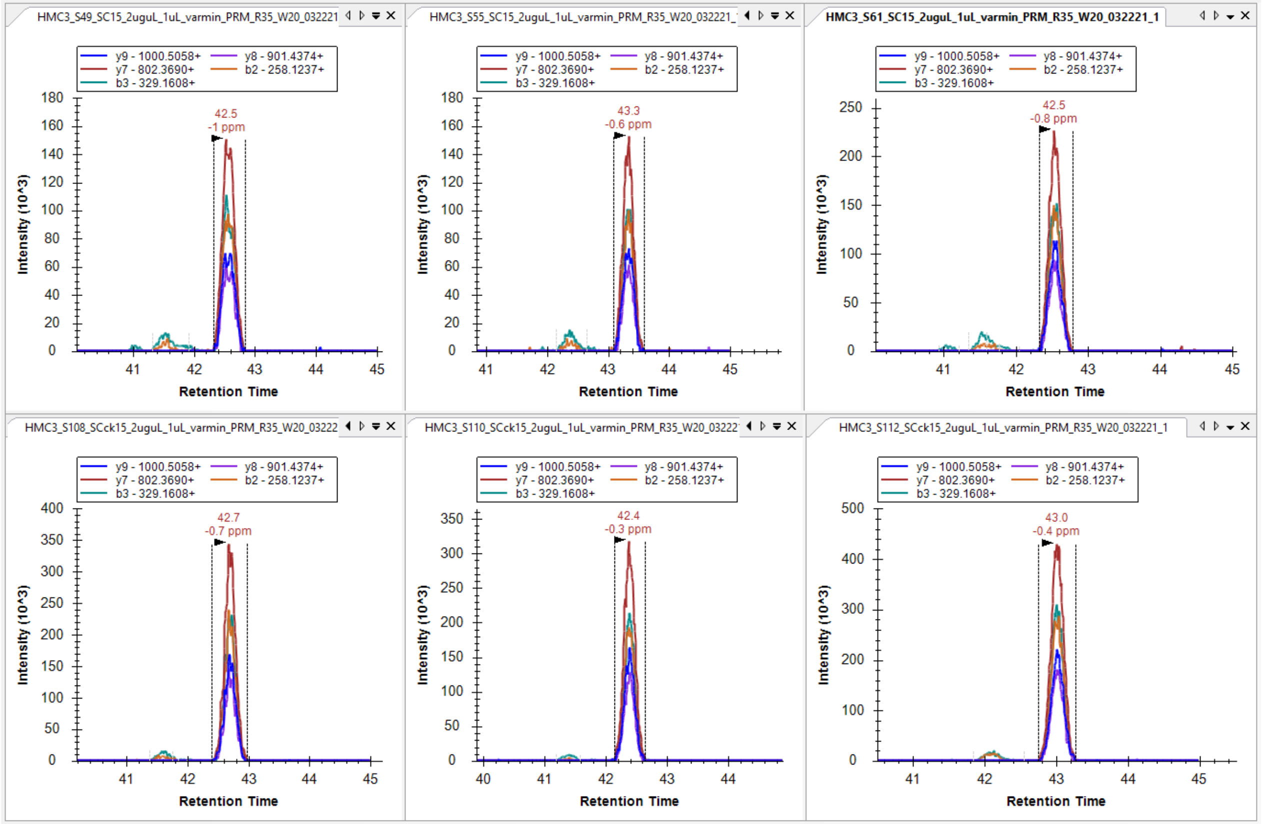

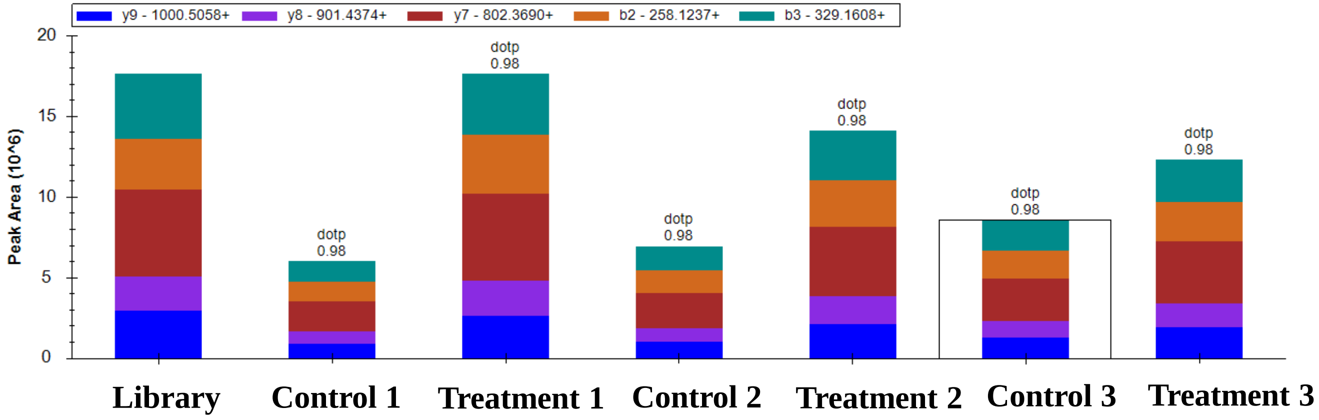

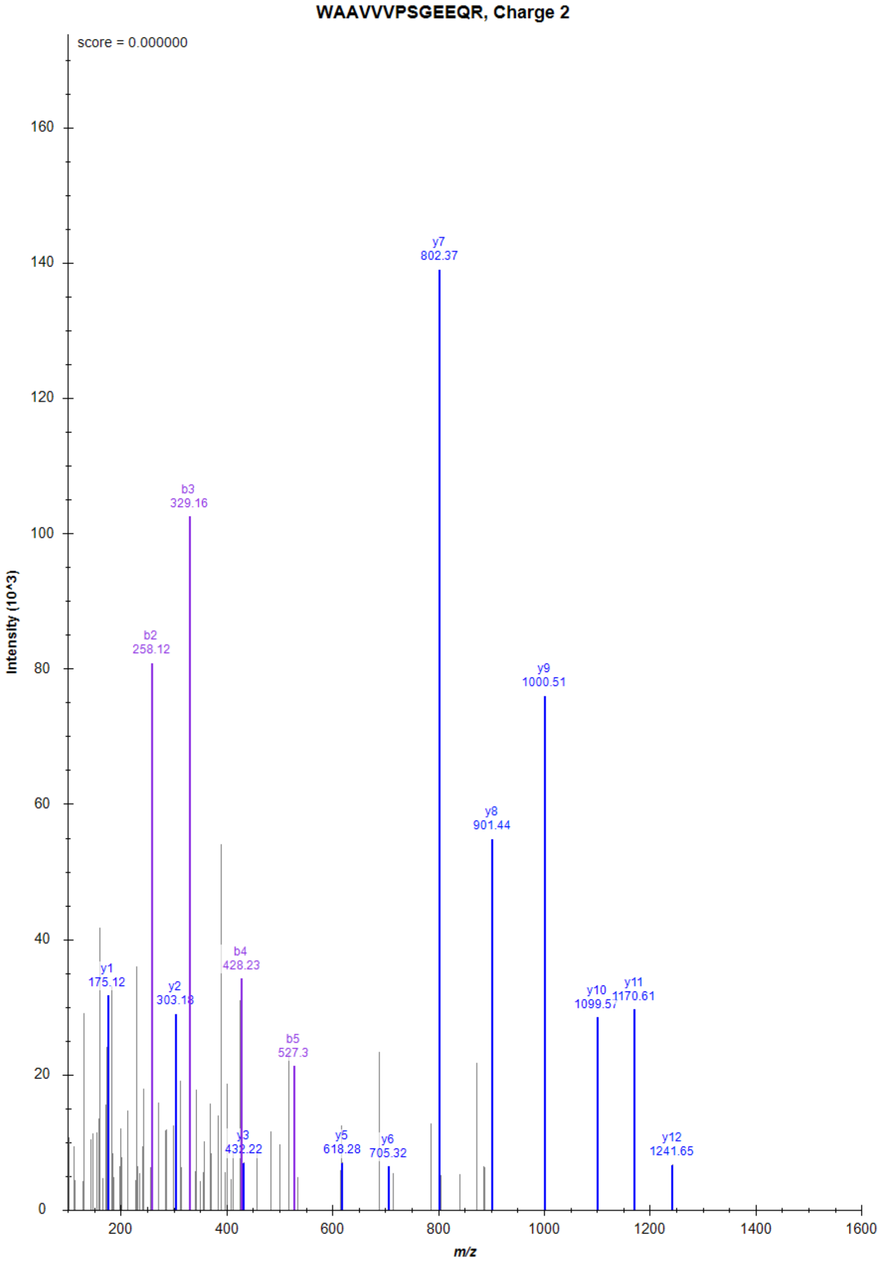

HLA class I histocompatibility antigen, Cw-8 (HLA-C)

APWVEQEGPEYWDR, Charge 2, m/z 881.39771

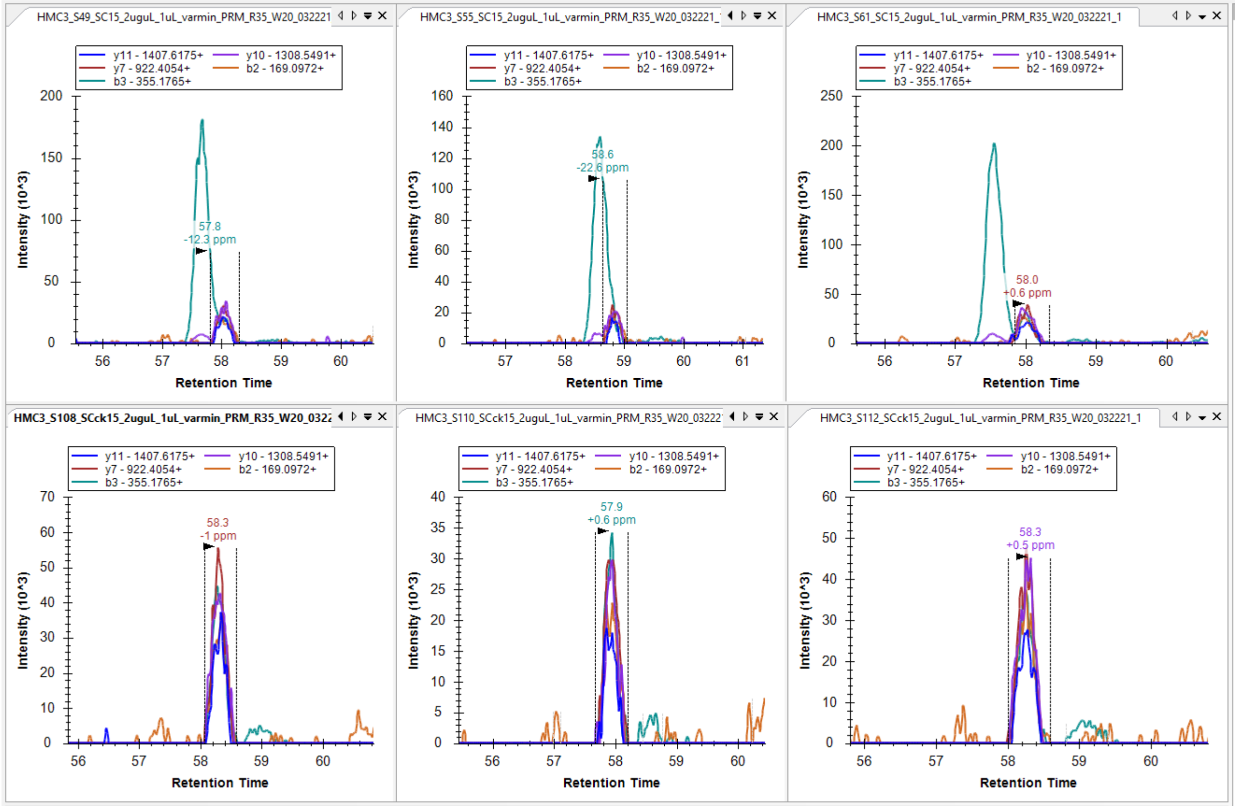

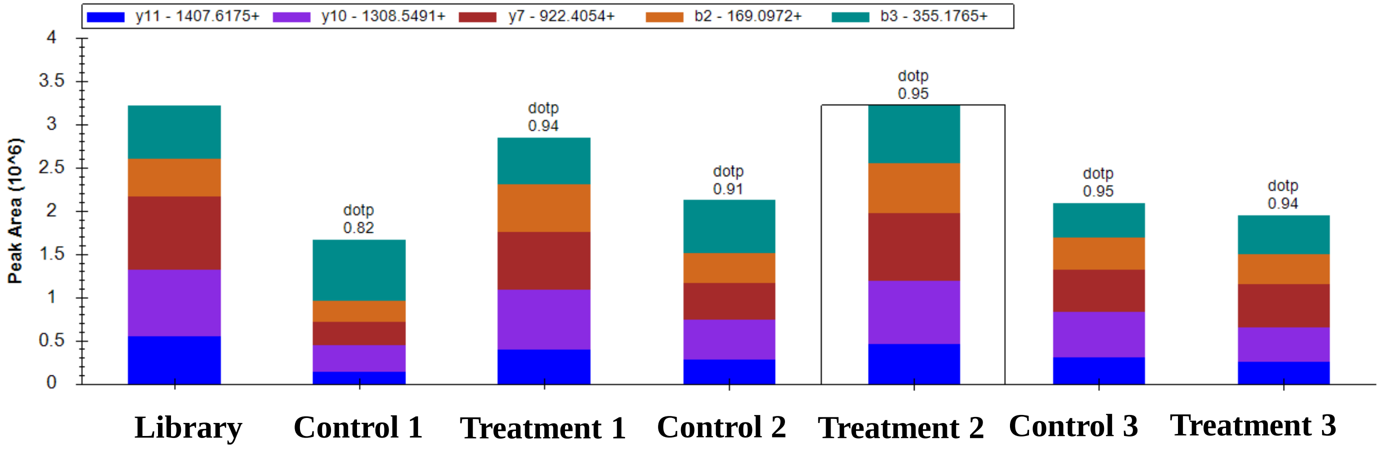

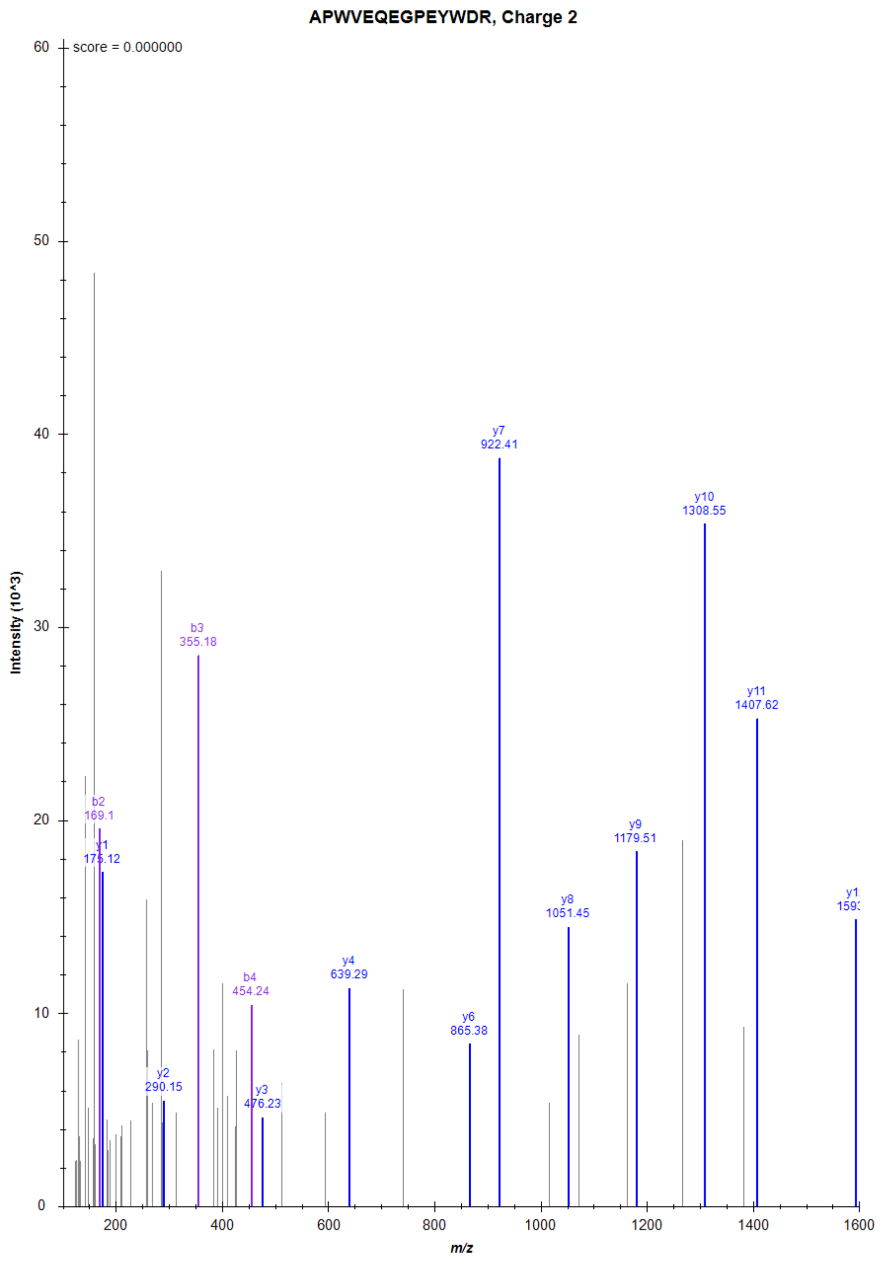

HLA class I histocompatibility antigen, Cw-8 (HLA-C)

SWTAADTAAQITQR, Charge 2, m/z 760.38116

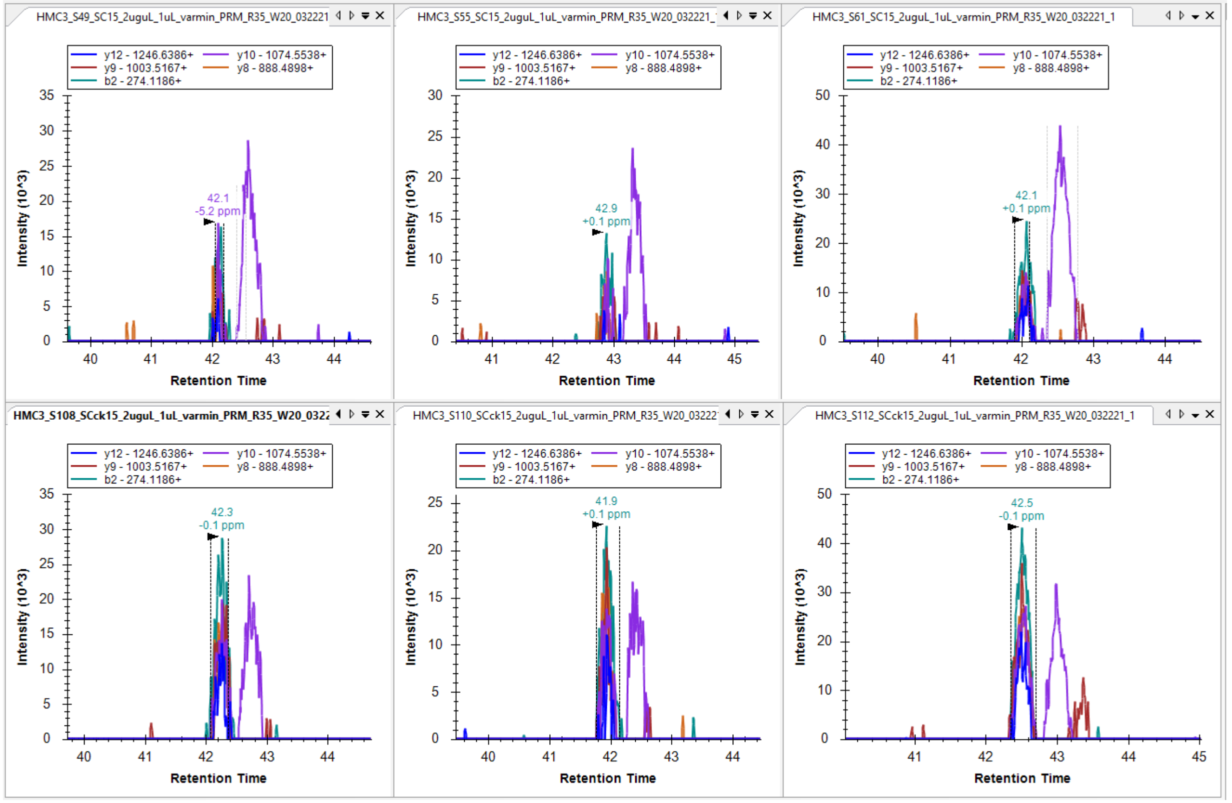

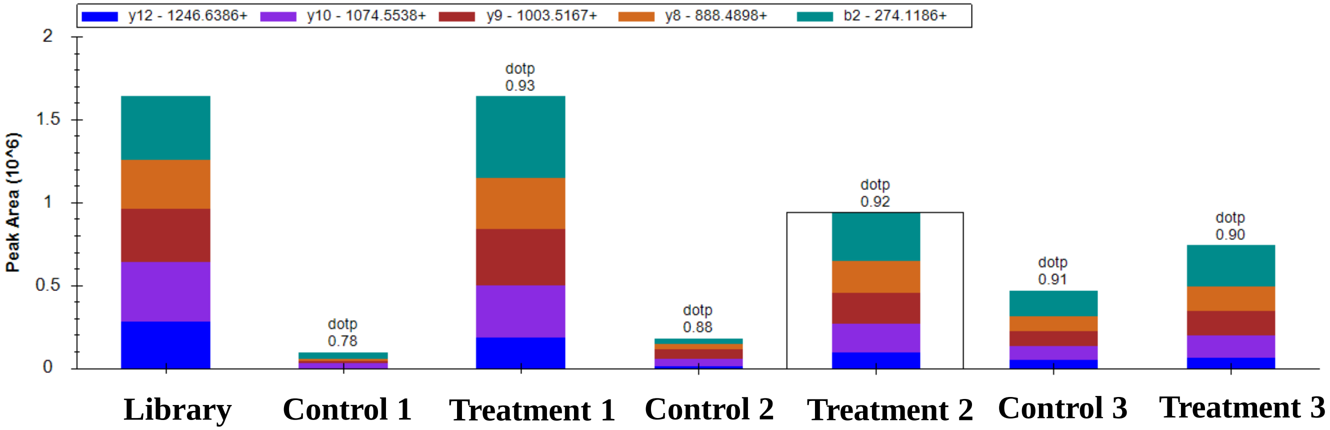

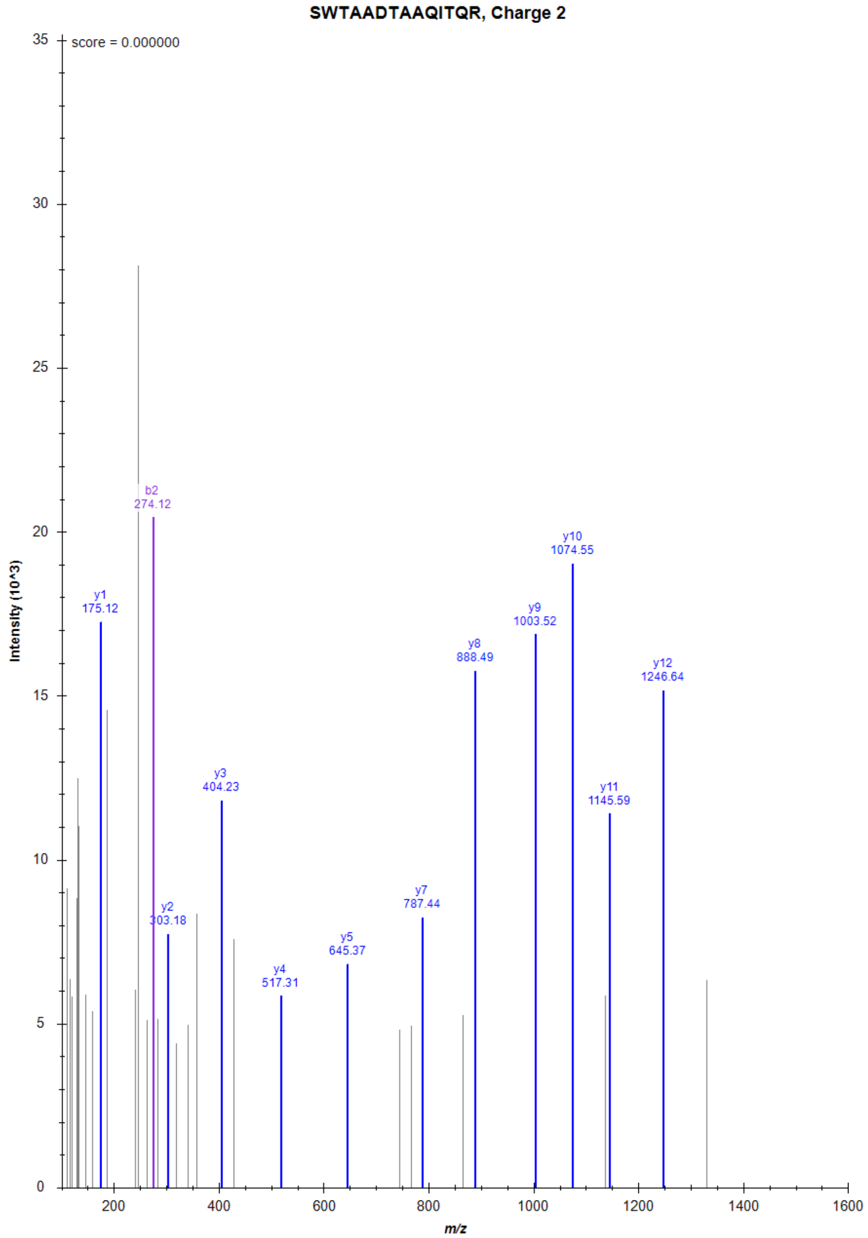

Category 4

Cell cycle related proteins

G2/mitotic-specific cyclin-B1 (CCNB1)

VPMLVPVPVSEPVPEPEPEPEPEPVKEEK, Charge 3, m/z 1068.55212

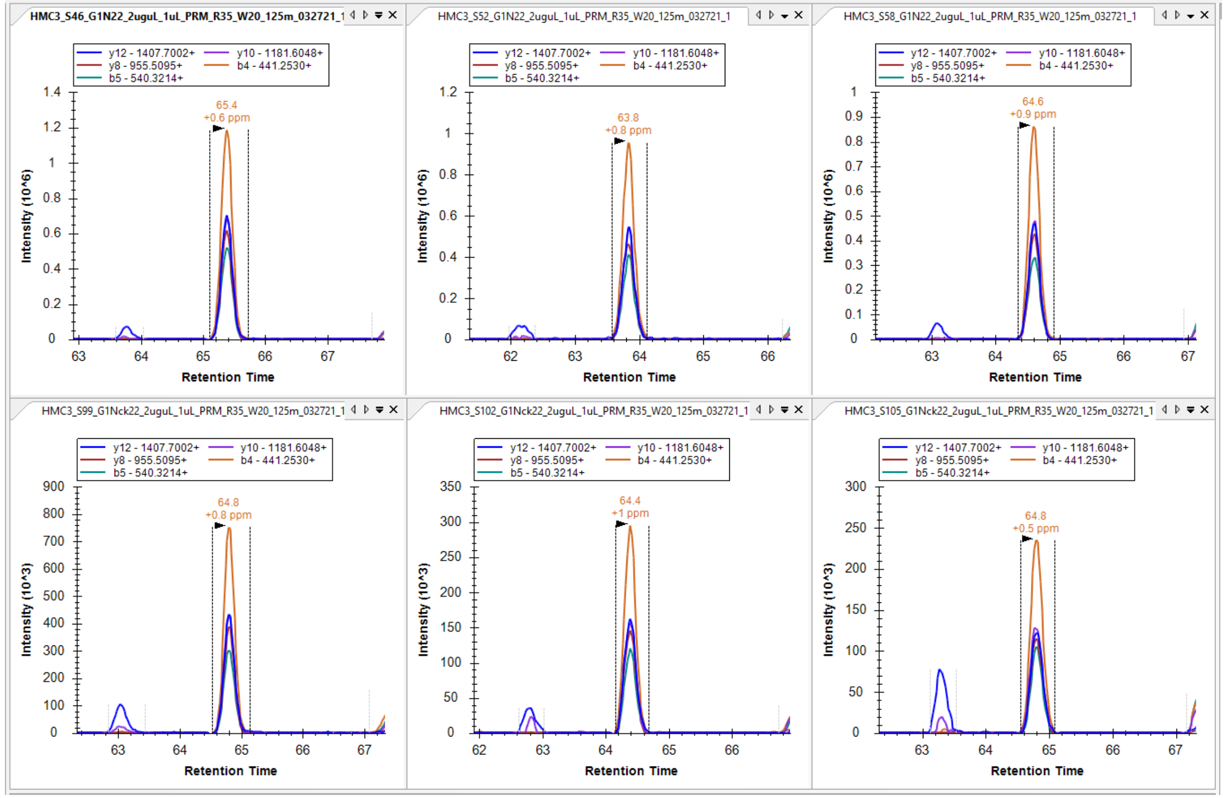

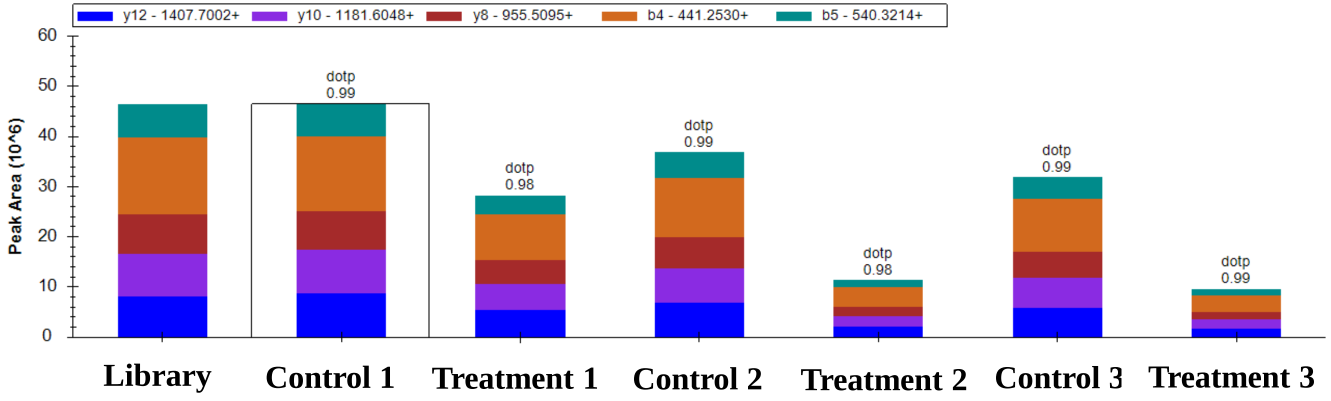

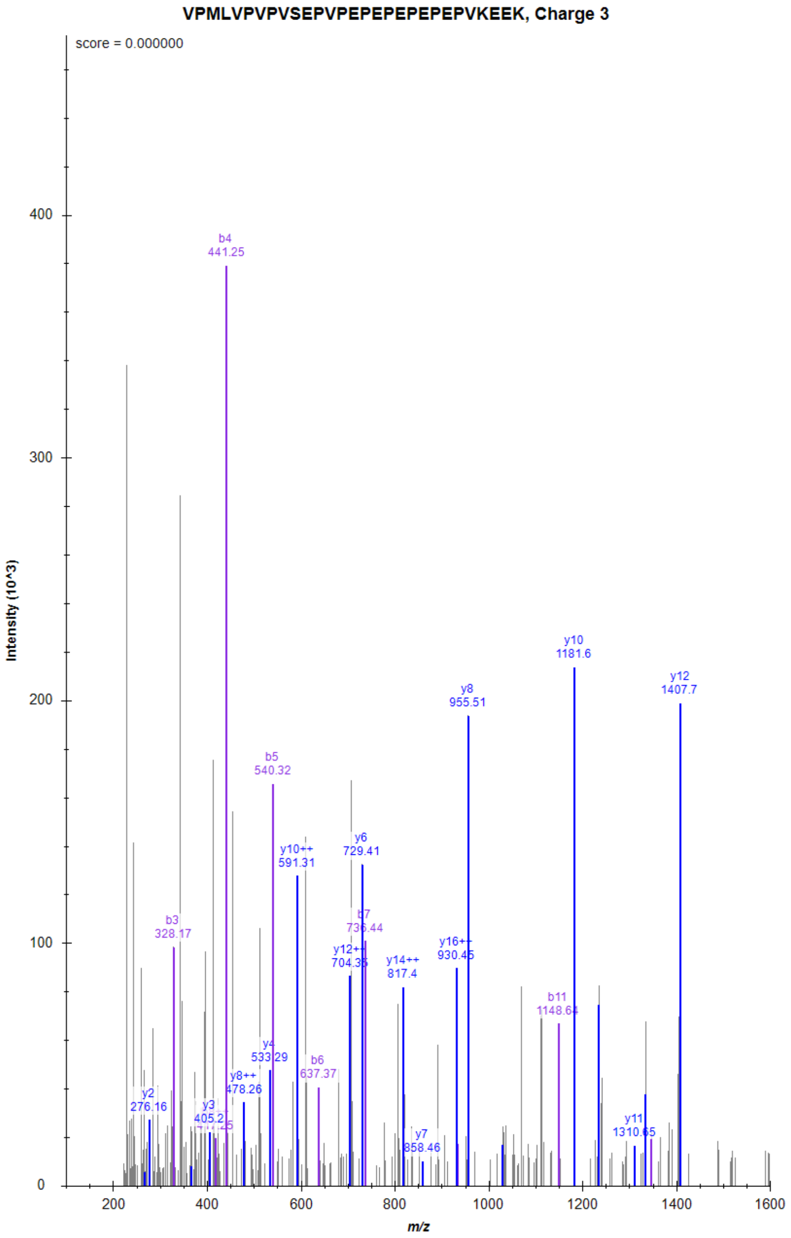

G2/mitotic-specific cyclin-B1 (CCNB1)

ISTLPQLNSALVQDLAK, Charge 2, m/z 906.01819

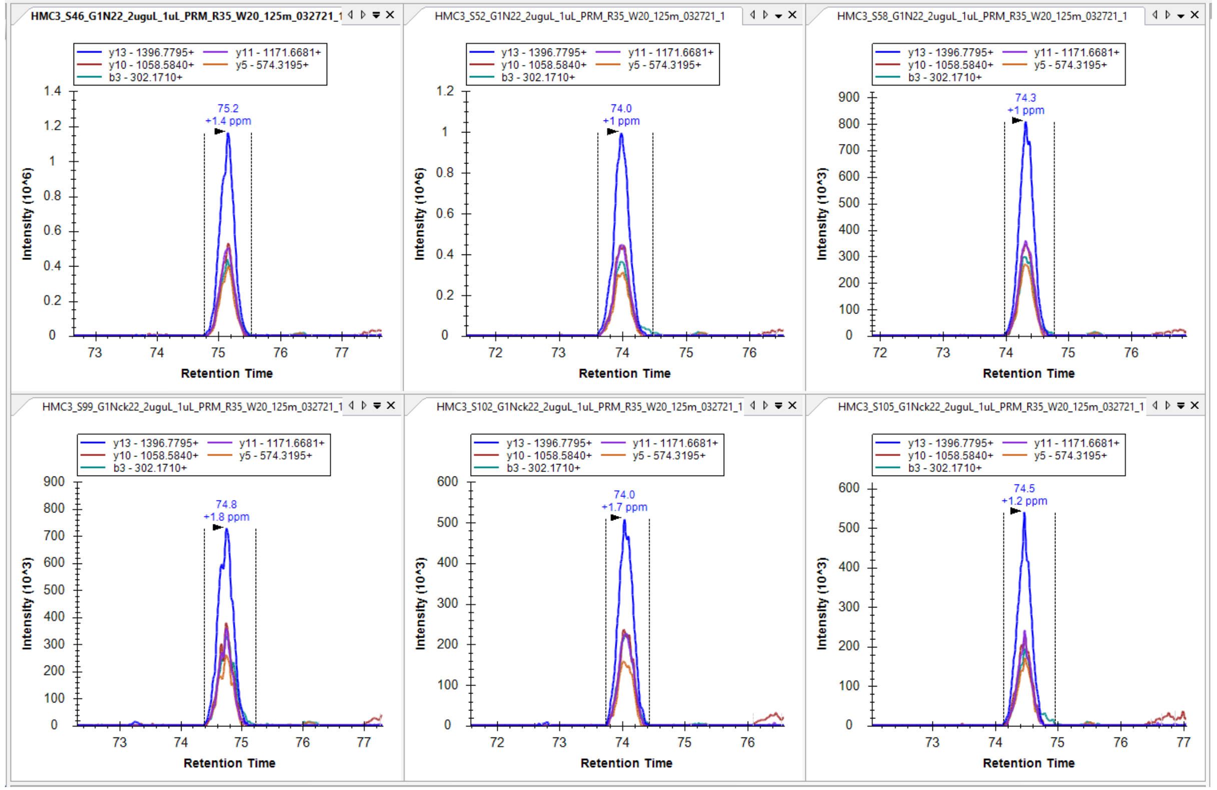

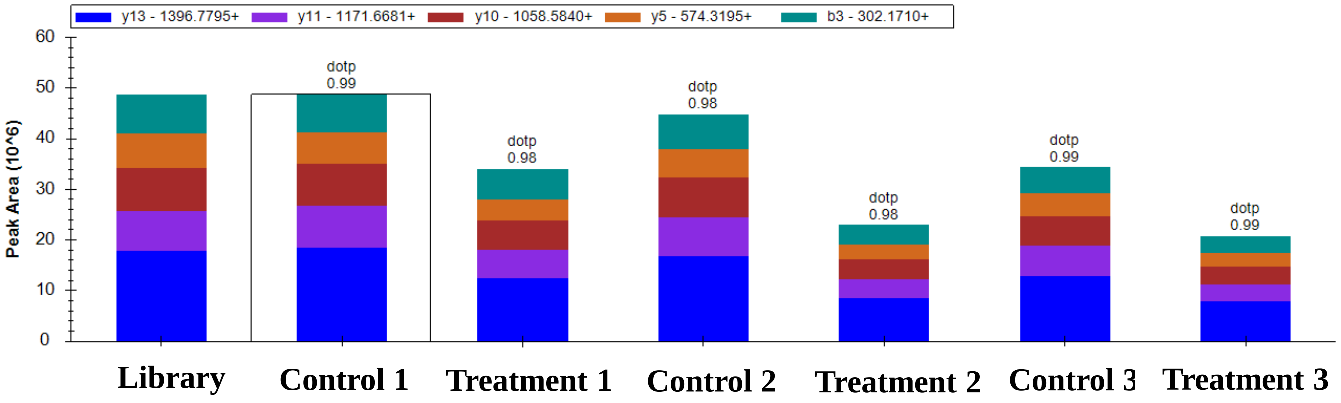

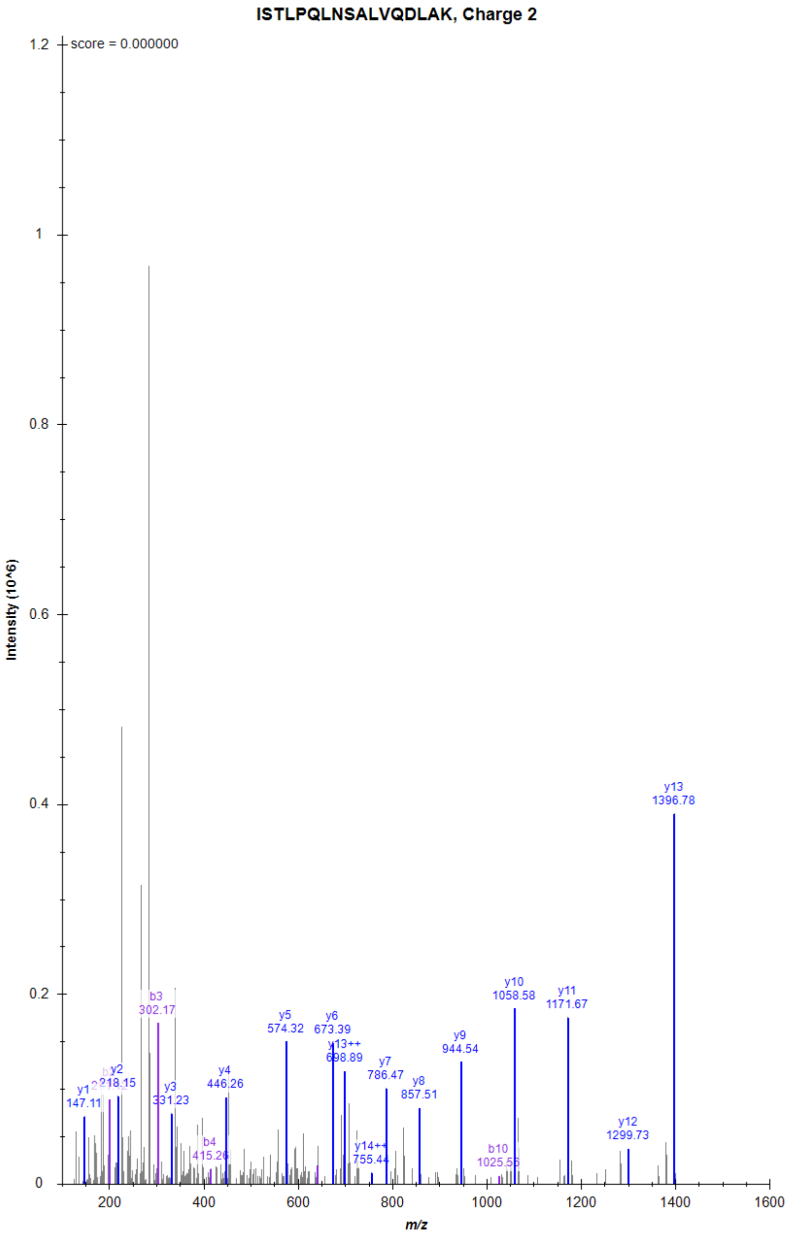

Cyclin-dependent kinase inhibitor 2A (CDKN2A)

LPVDLAEELGHR, Charge 2, m/z 674.86432

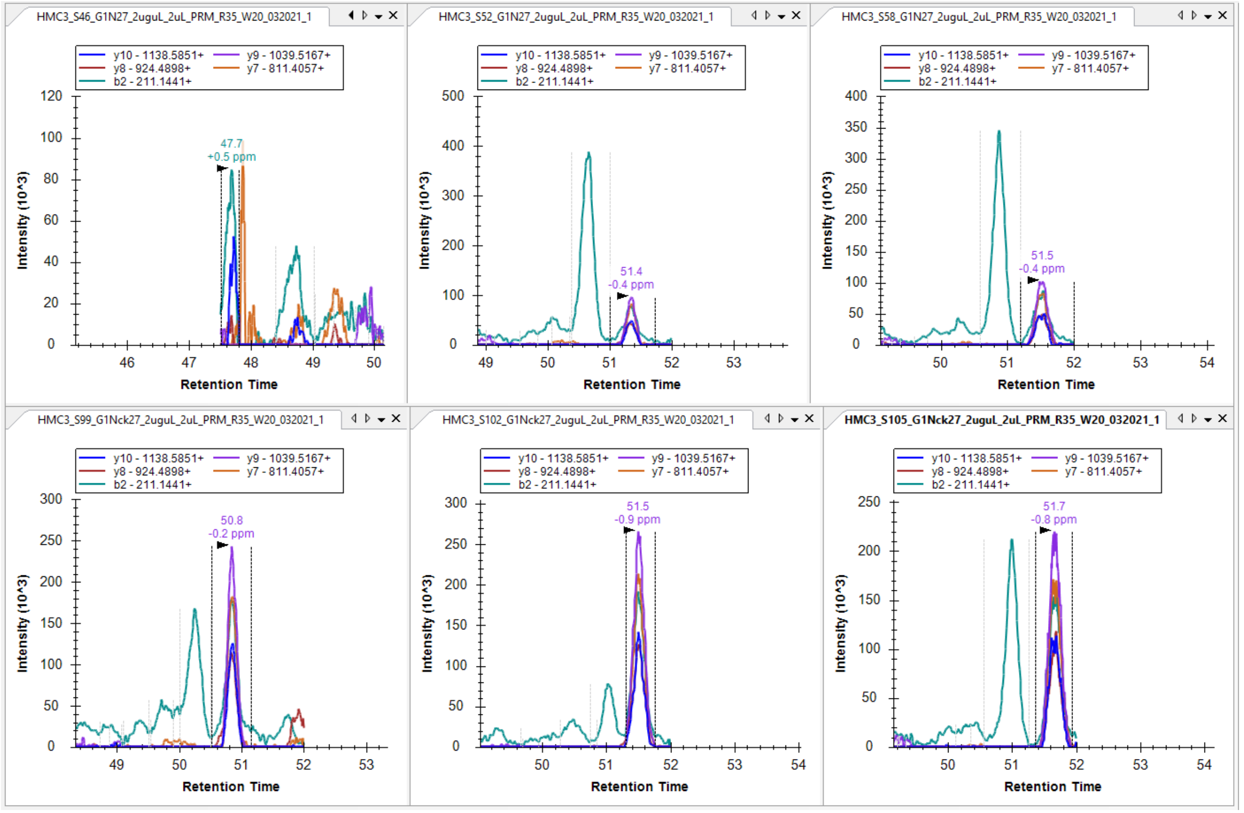

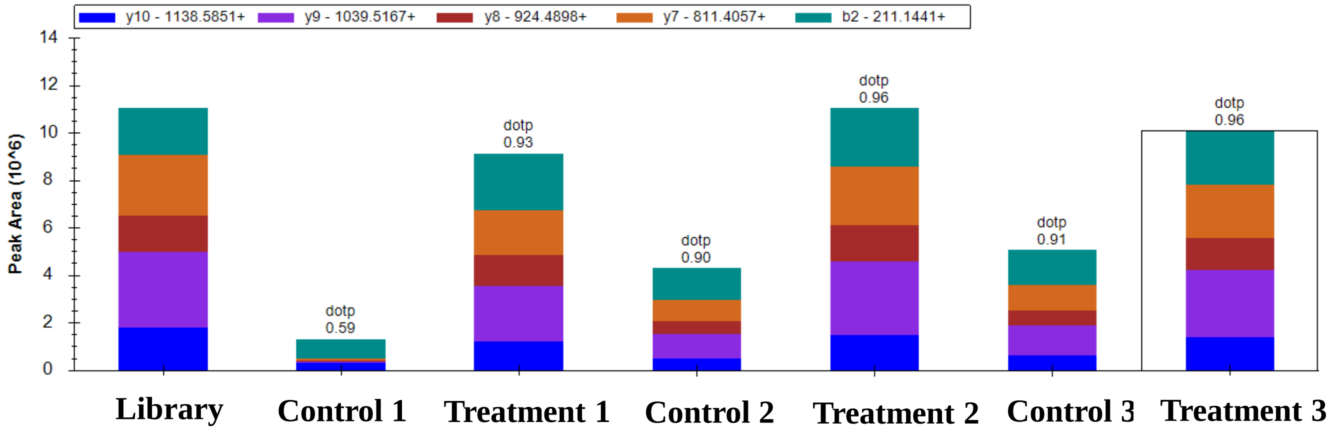

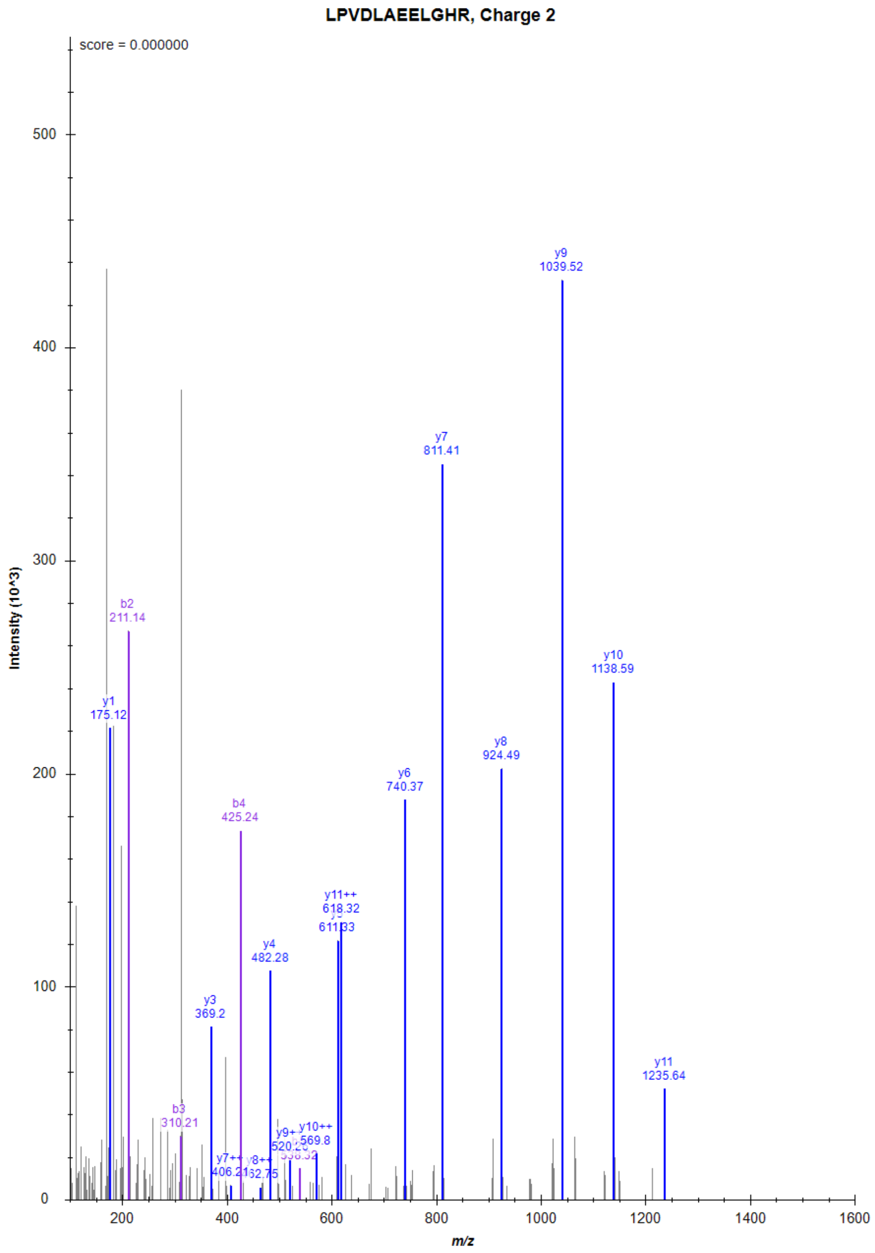

Cyclin-dependent kinase inhibitor 2A (CDKN2A)

ALLEAGALPNAPNSYGR, Charge 2, m/z 857.45227

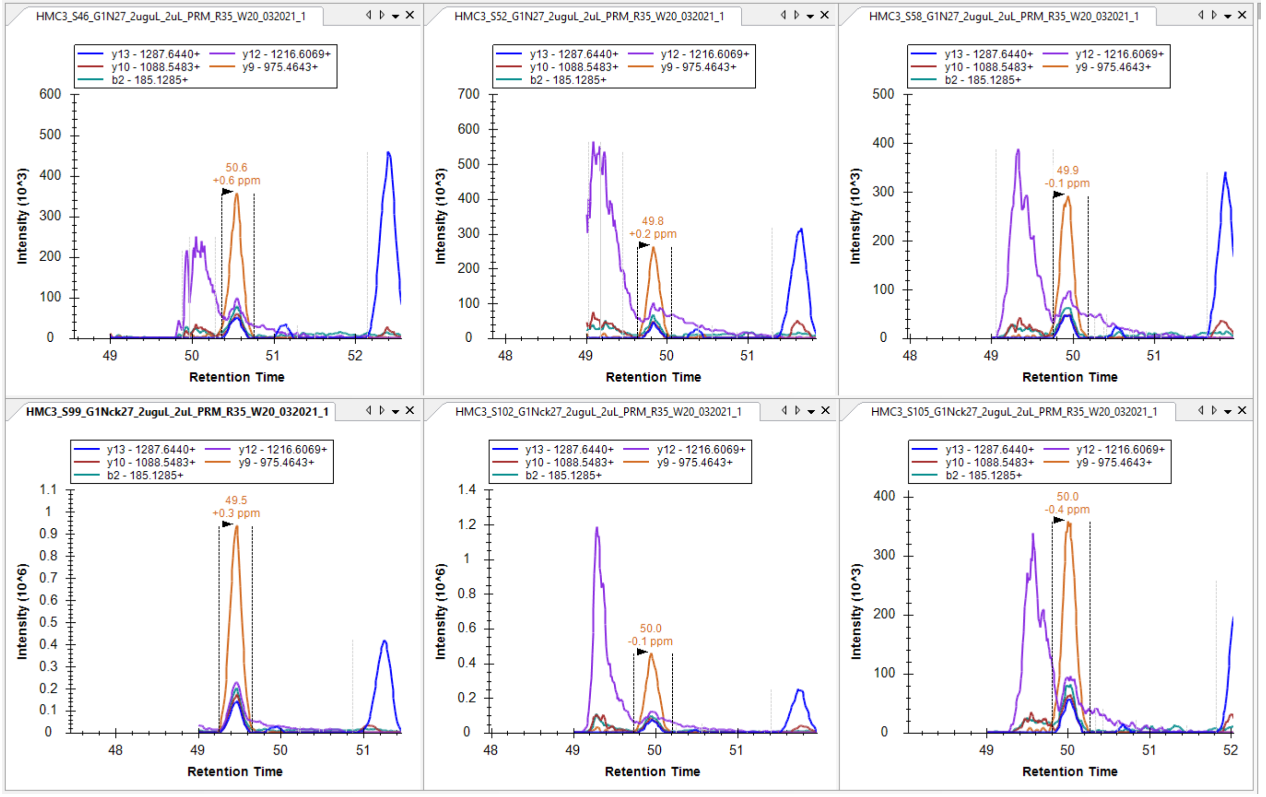

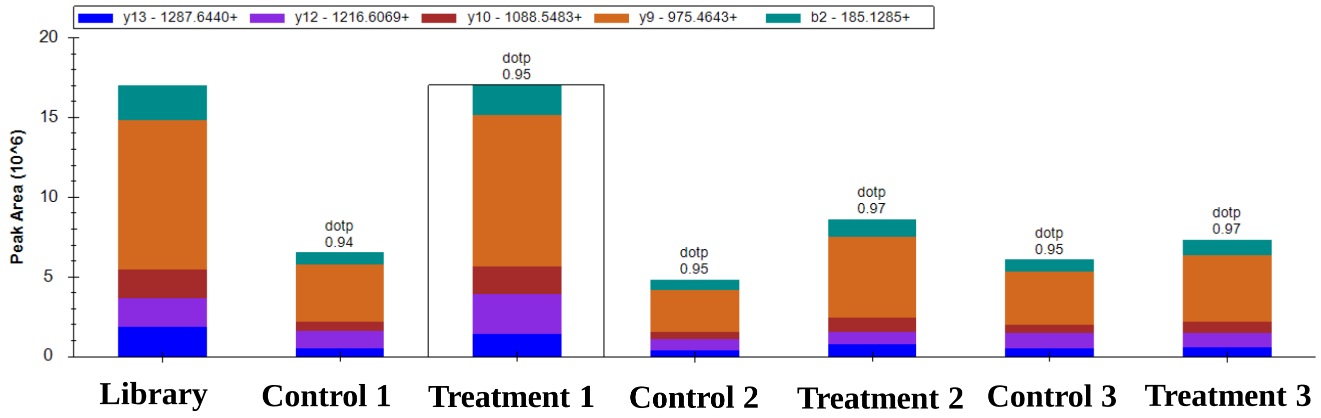

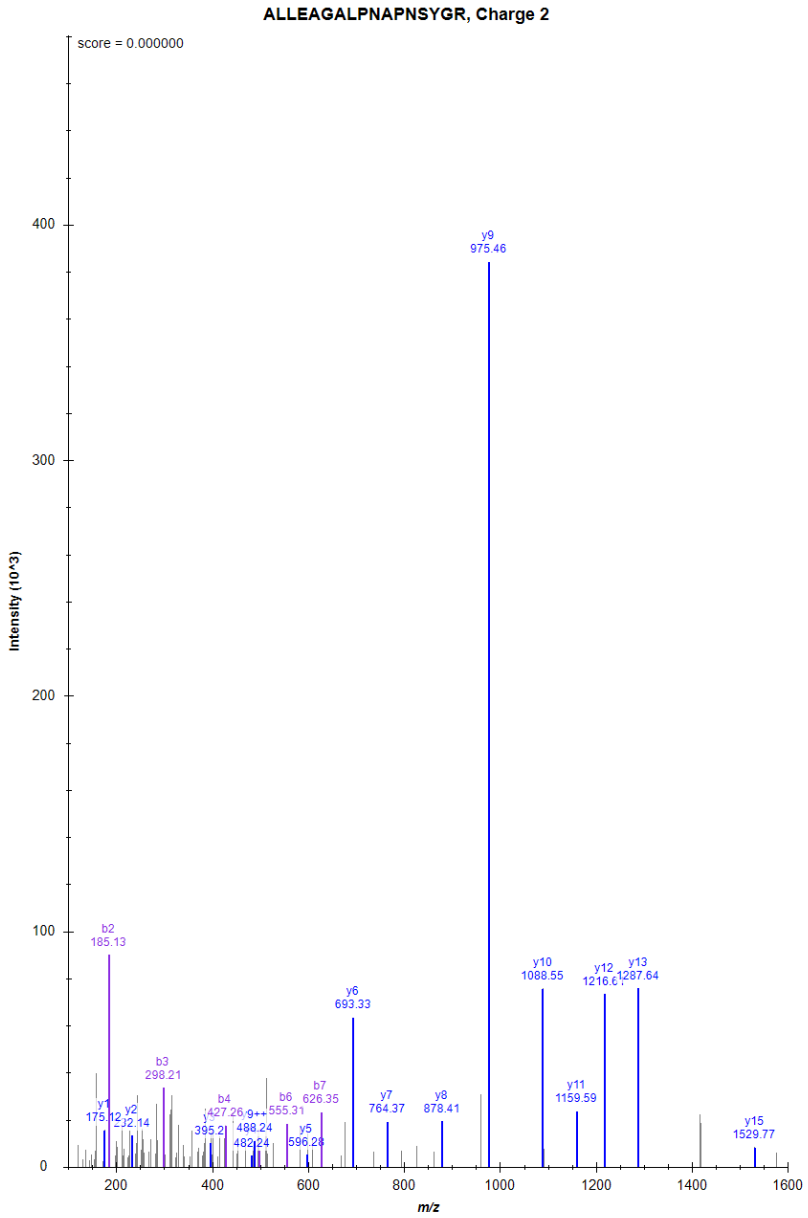

Cyclin-dependent kinase inhibitor 1 (CDKN1A)

LFGPVDSEQLSR, Charge 2, m/z 674.35016

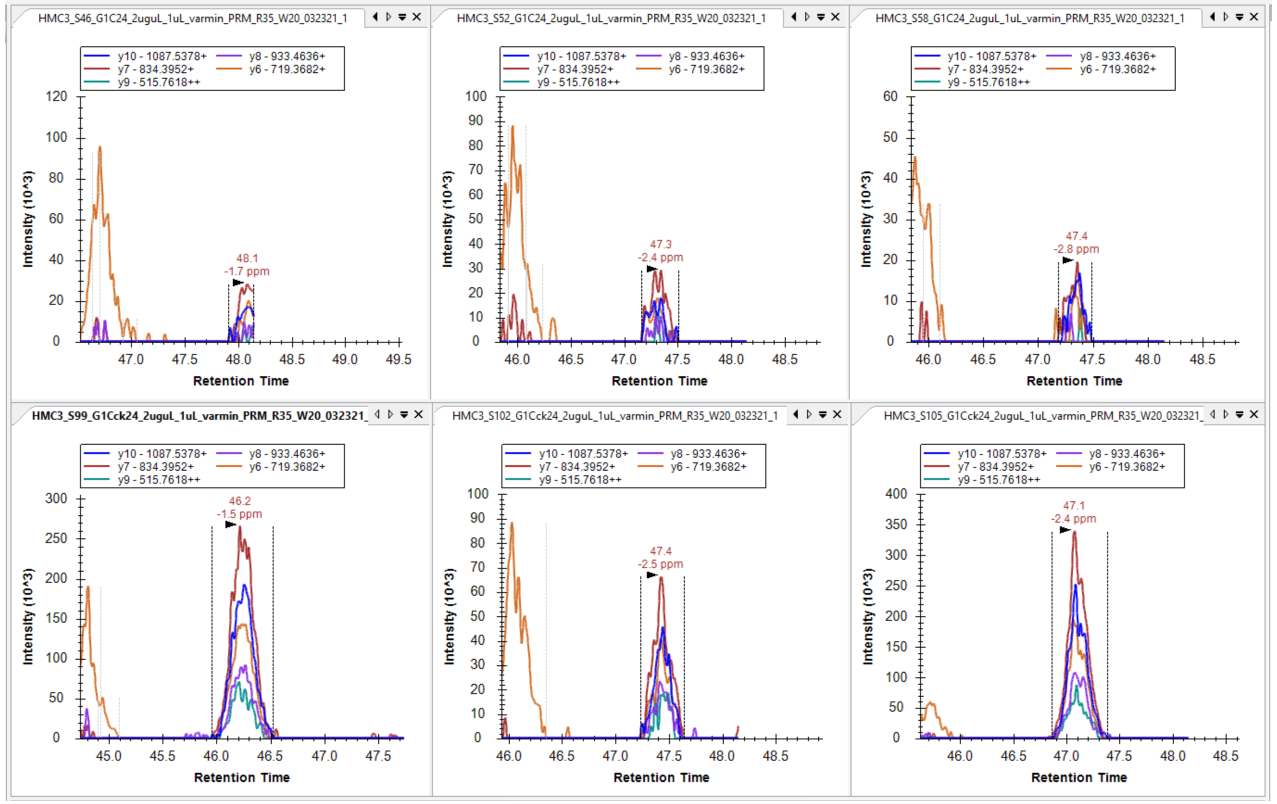

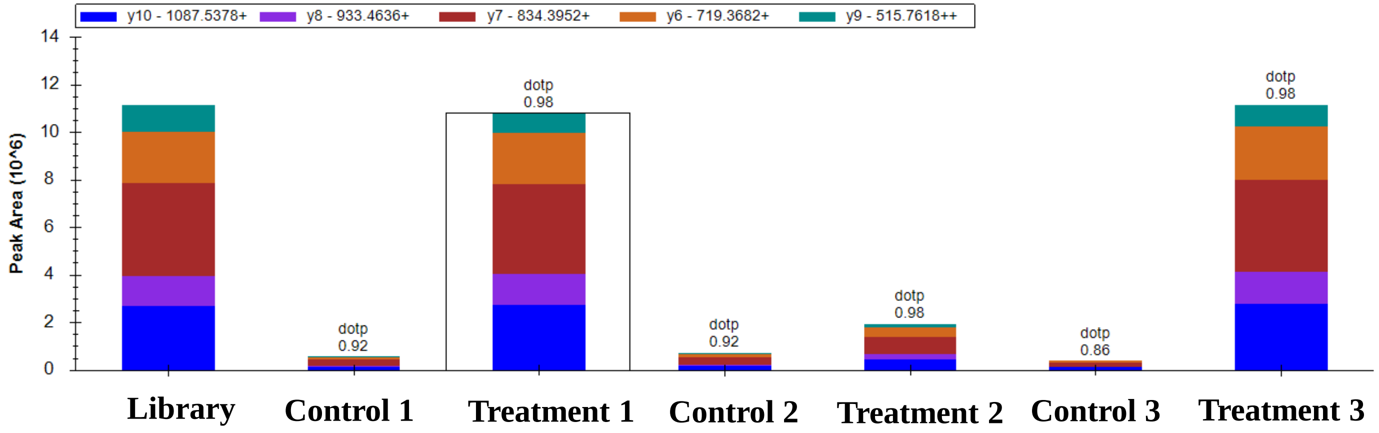

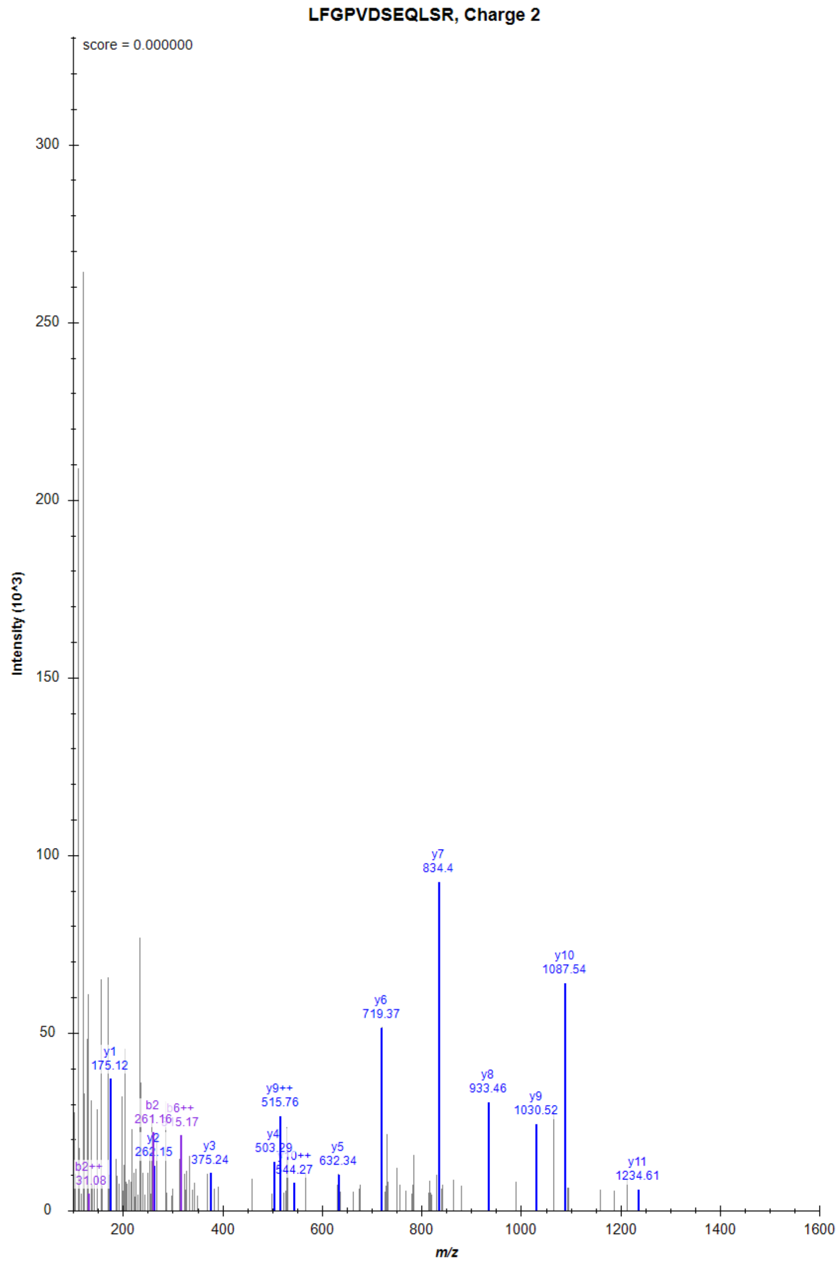

Transcription initiation factor TFIID subunit 1 (TAF1)

AINPLLDDDDQVAFSFILDNIVTQK, Charge 3, m/z 935.48328

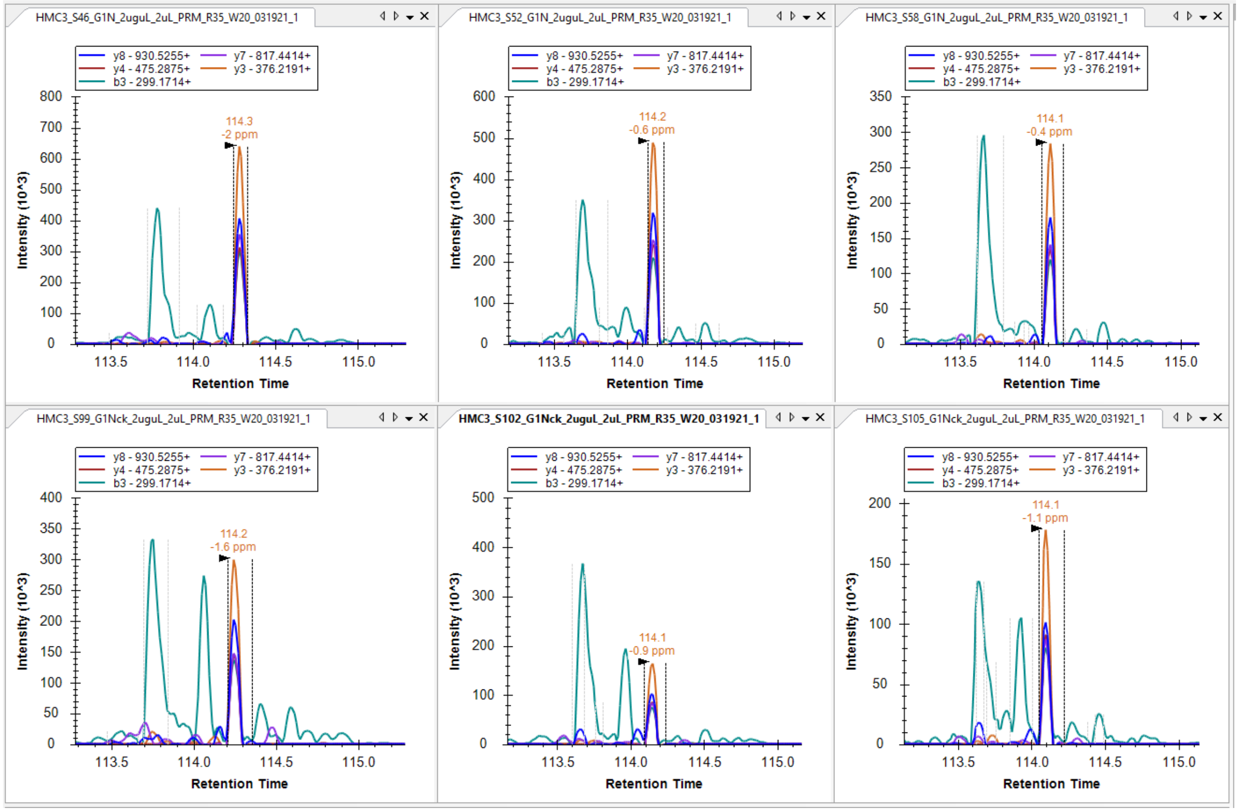

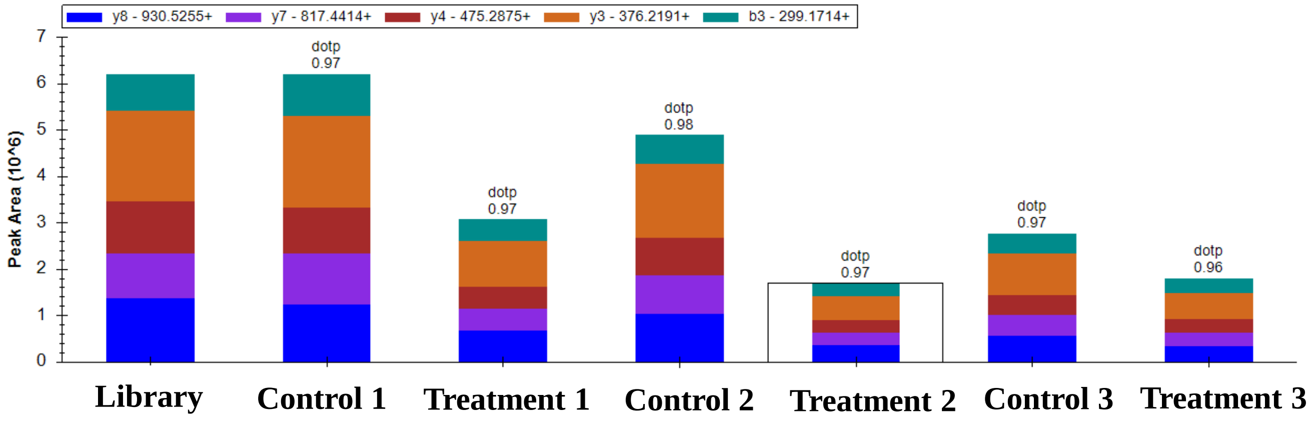

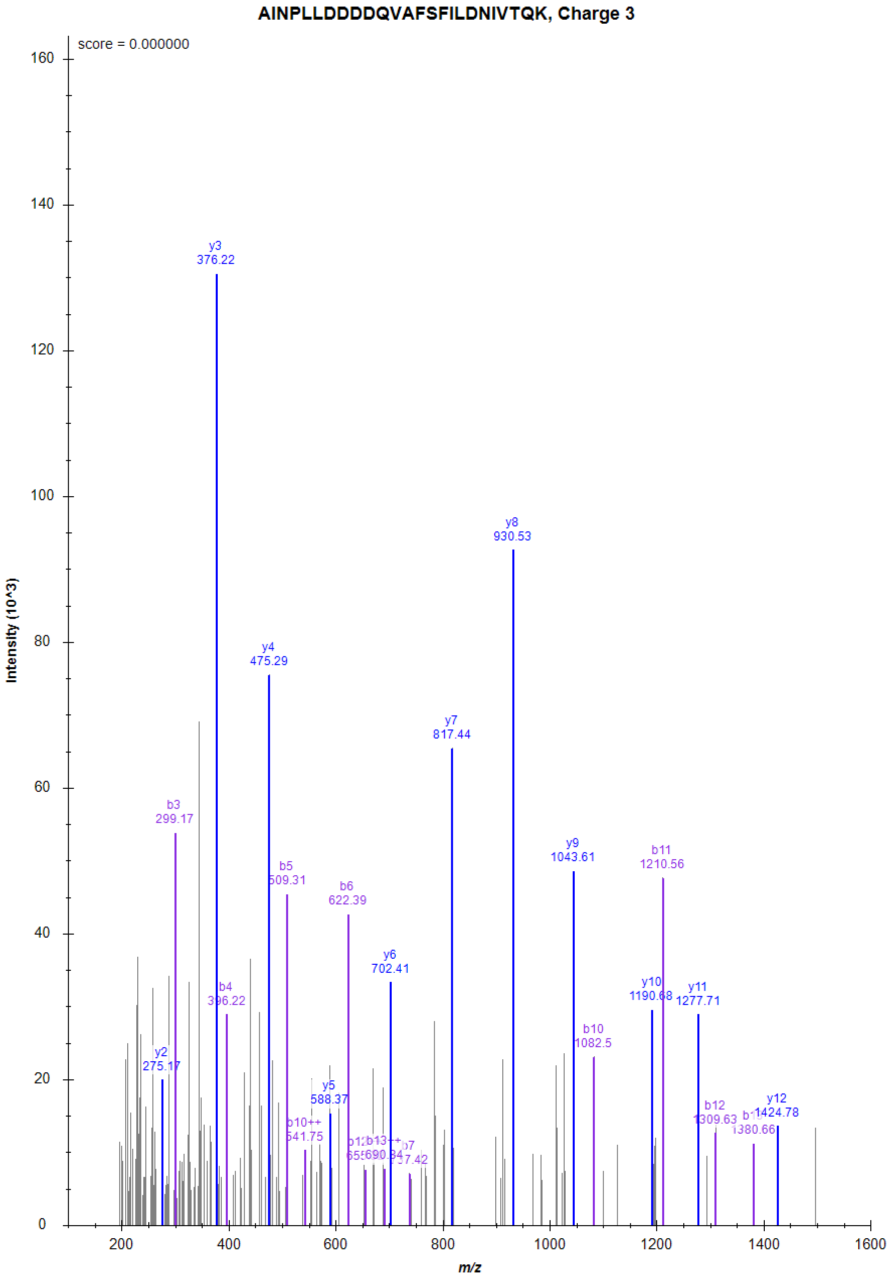

Replication factor C subunit 3 (RFC3)

GEVAQMAAYYEHR, Charge 2, m/z 762.84875

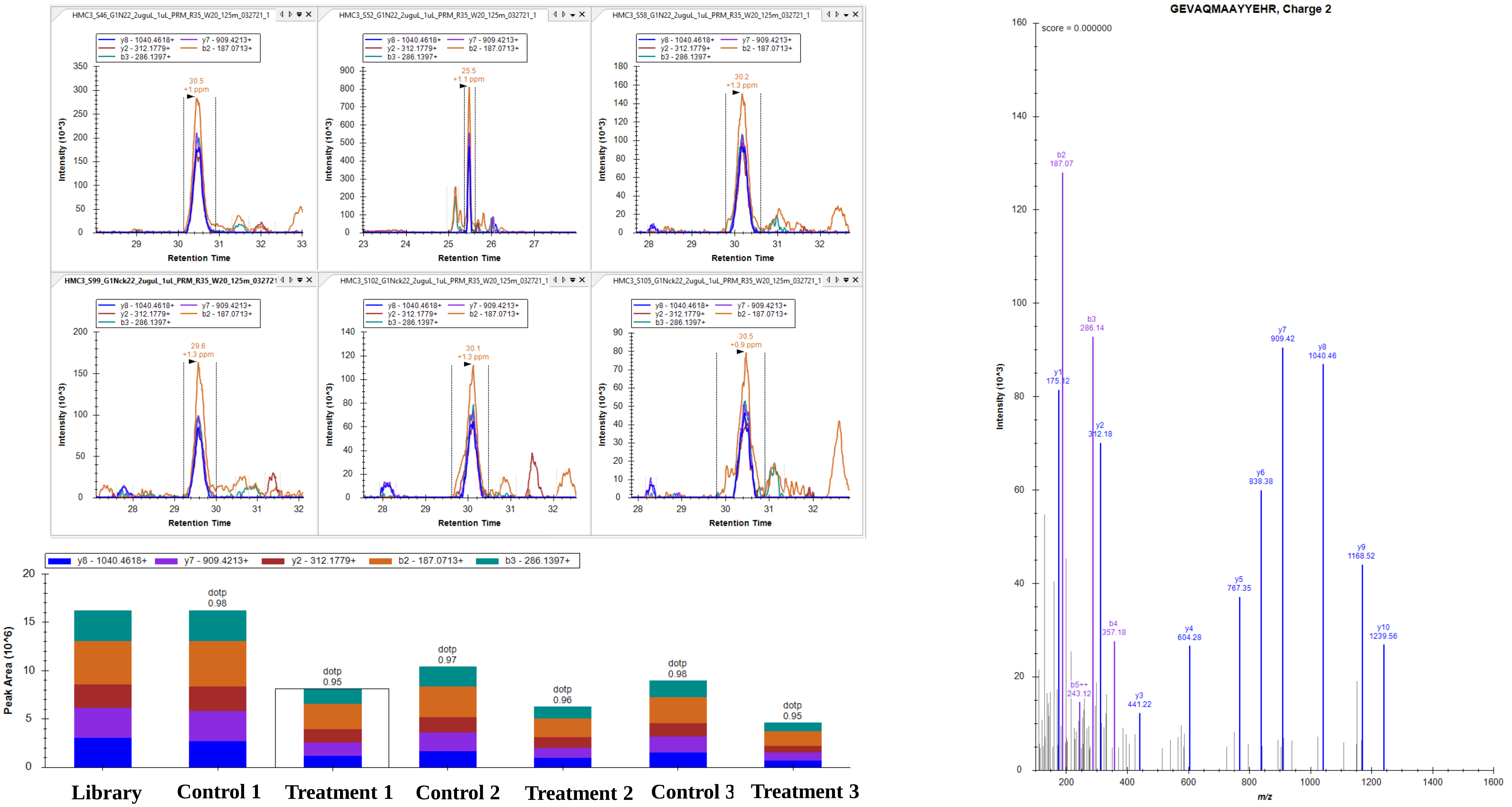

Prohibitin (PHB)

AATFGLILDDVSLTHLTFGK, Charge 2, m/z 1060.07739

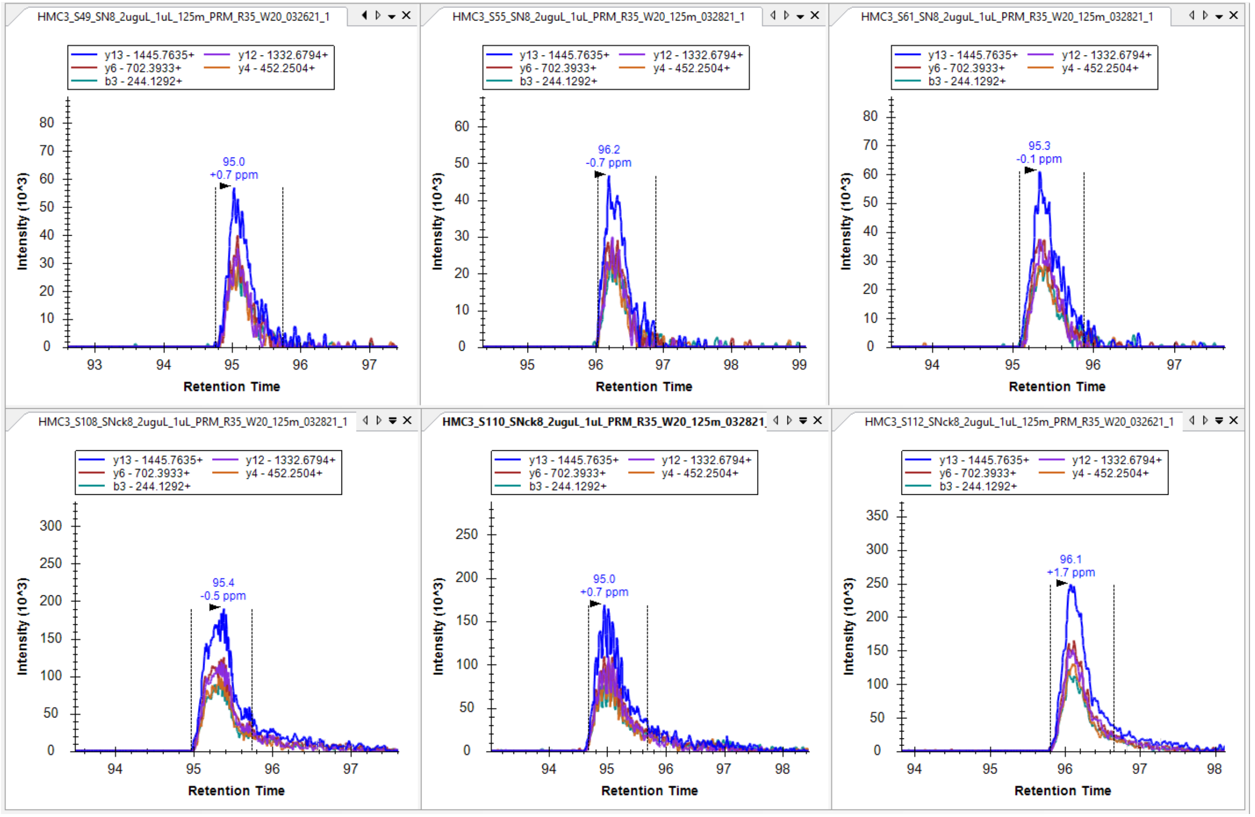

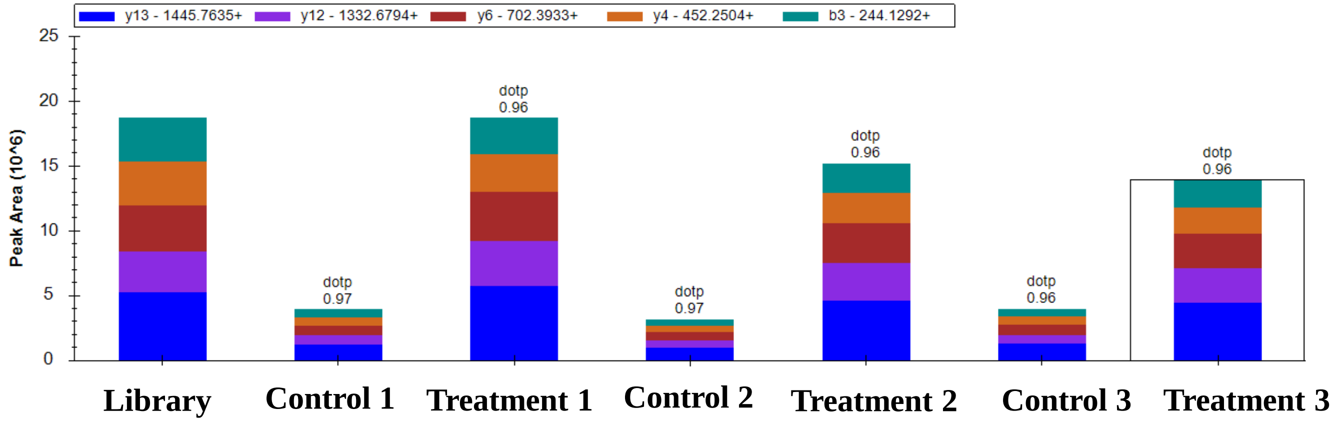

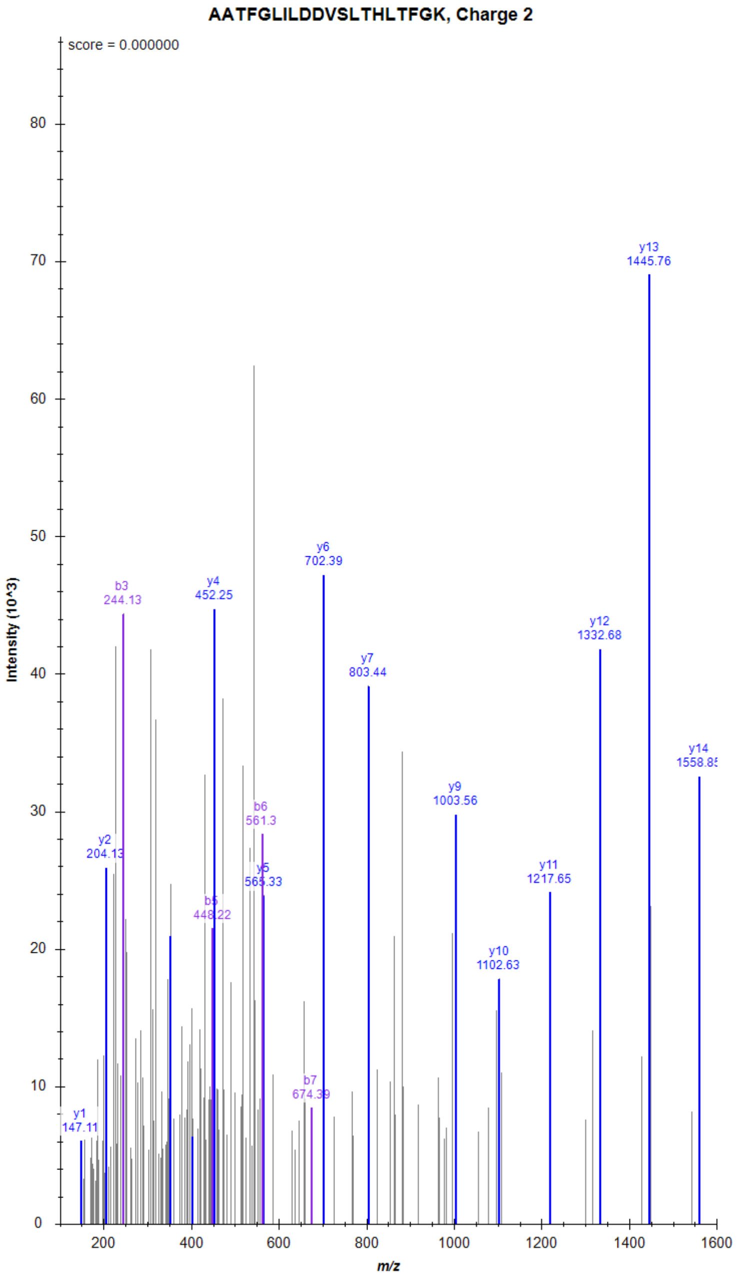

Prohibitin (PHB)

FGLALAVAGGVVNSALYNVDAGHR, Charge 3, m/z 791.09003

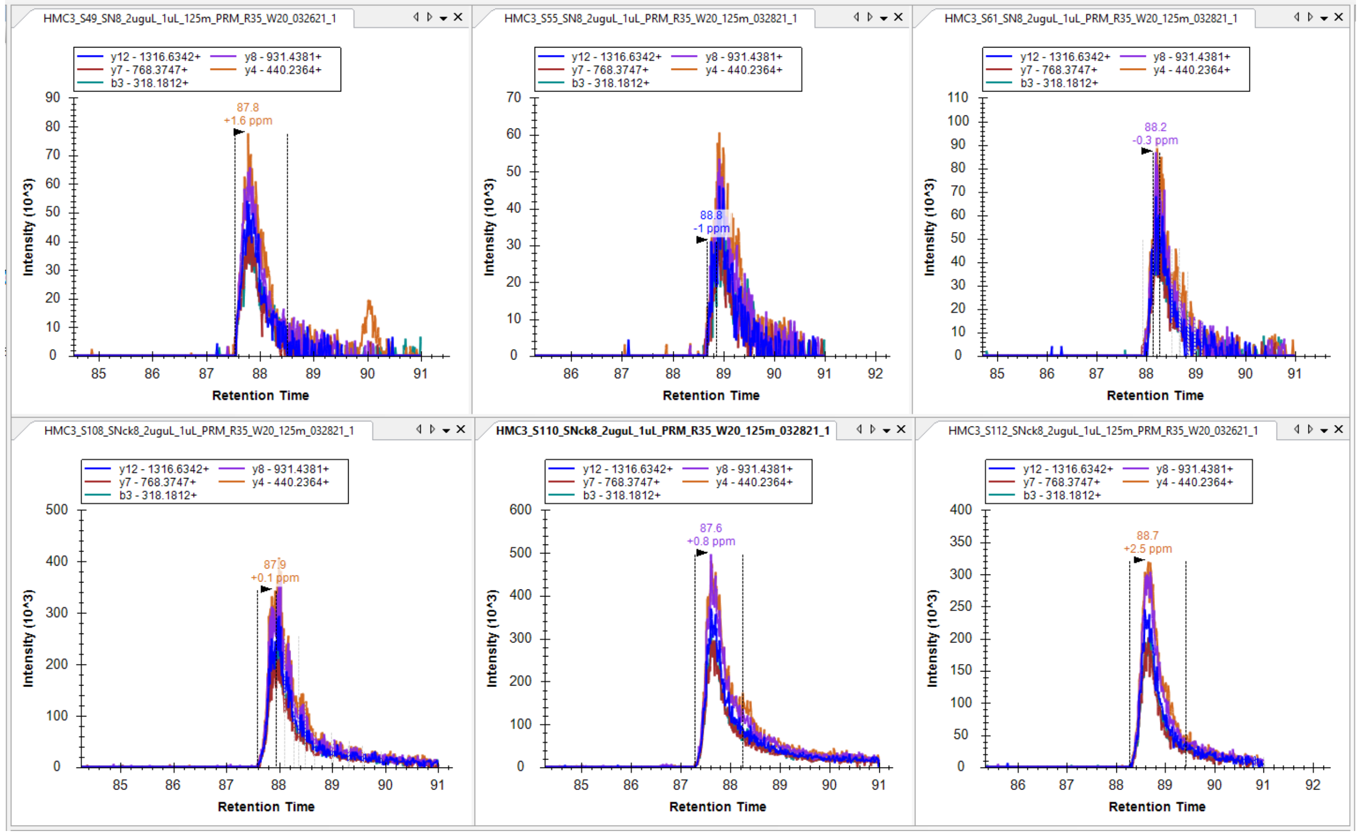

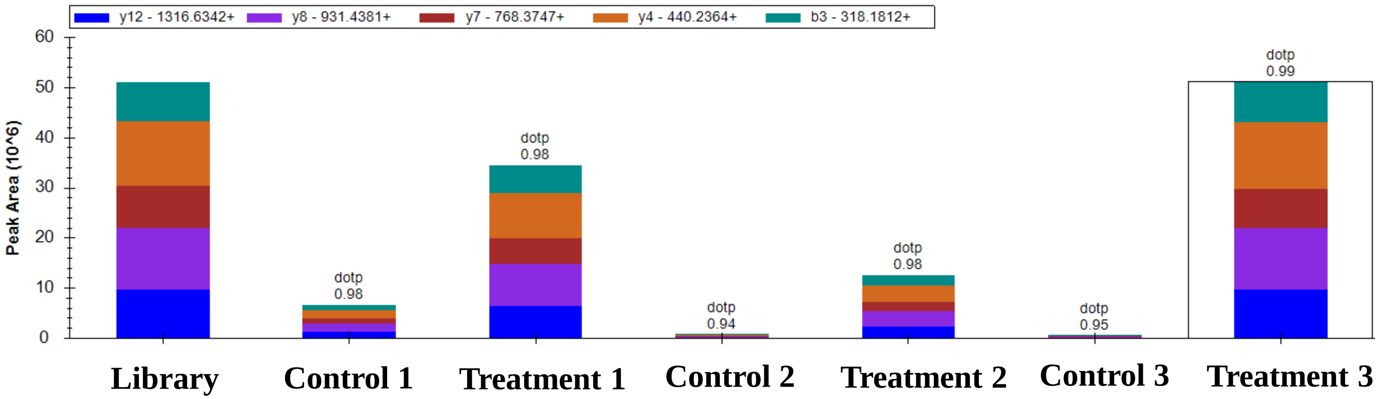

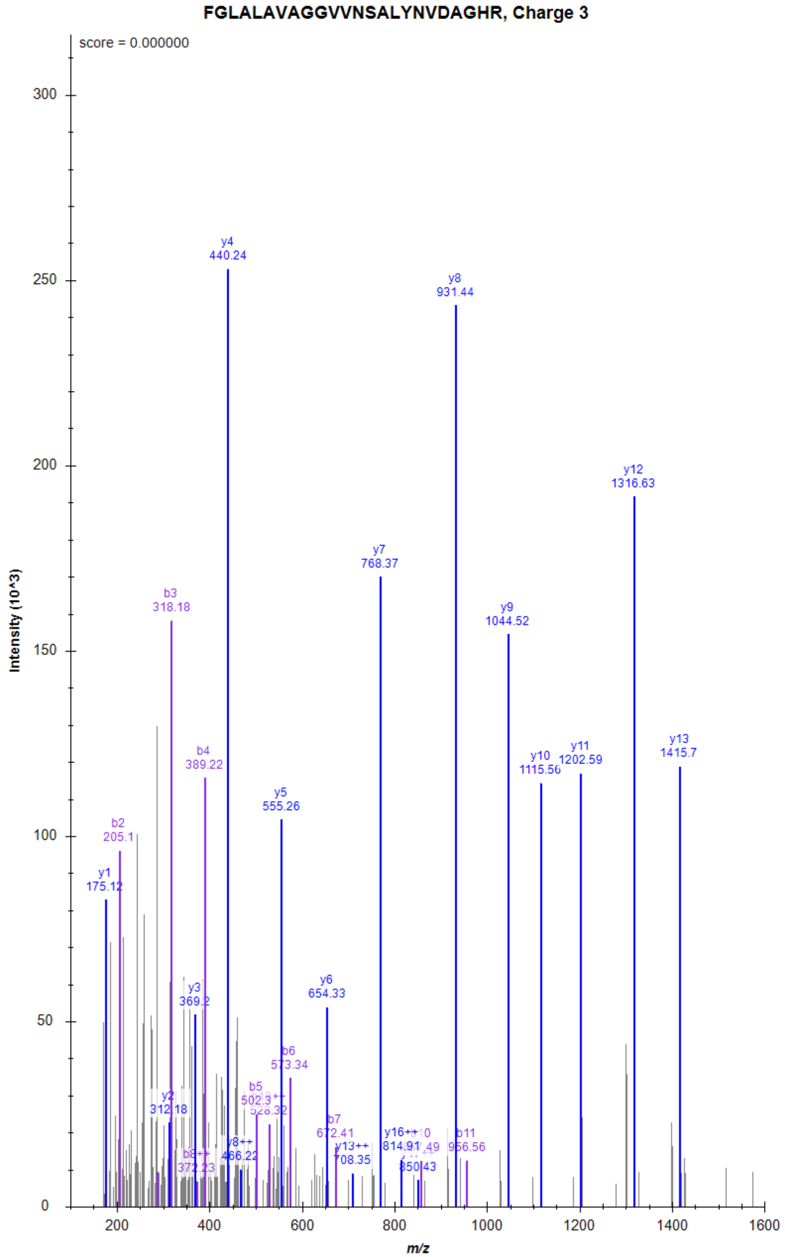

Proliferation marker protein Ki-67 (MKI67)

KVDVEEEFFALR, Charge 2, m/z 741.38544 (*20 min LC run*)

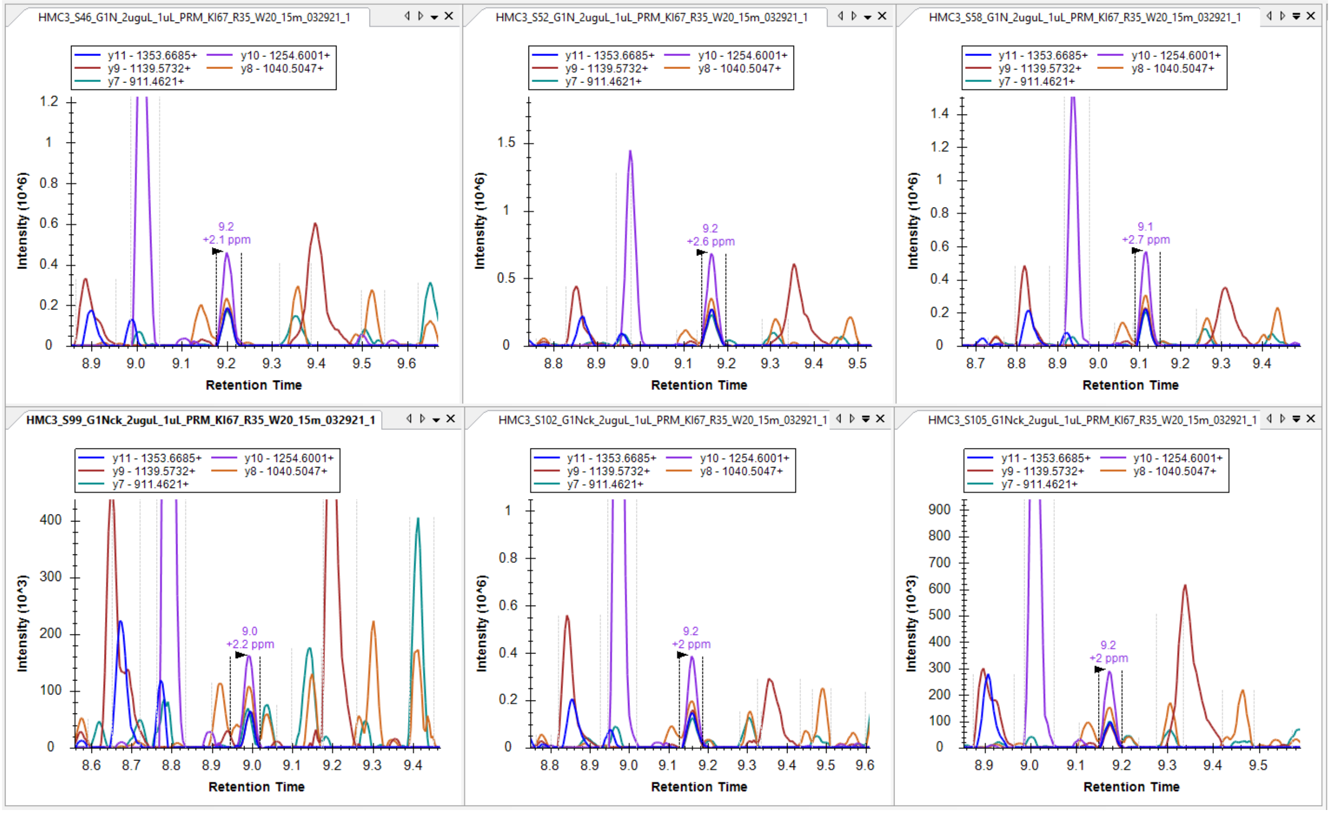

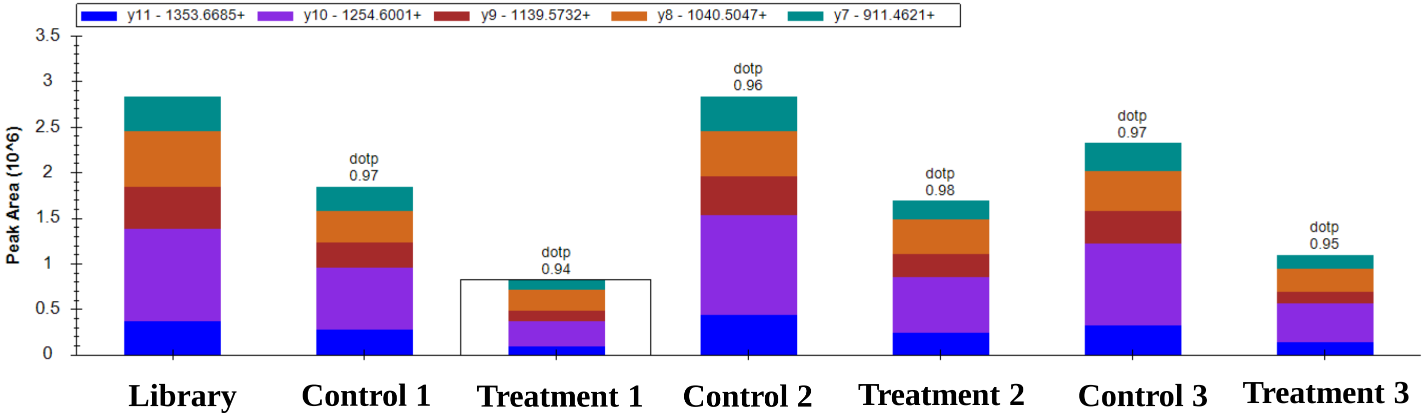

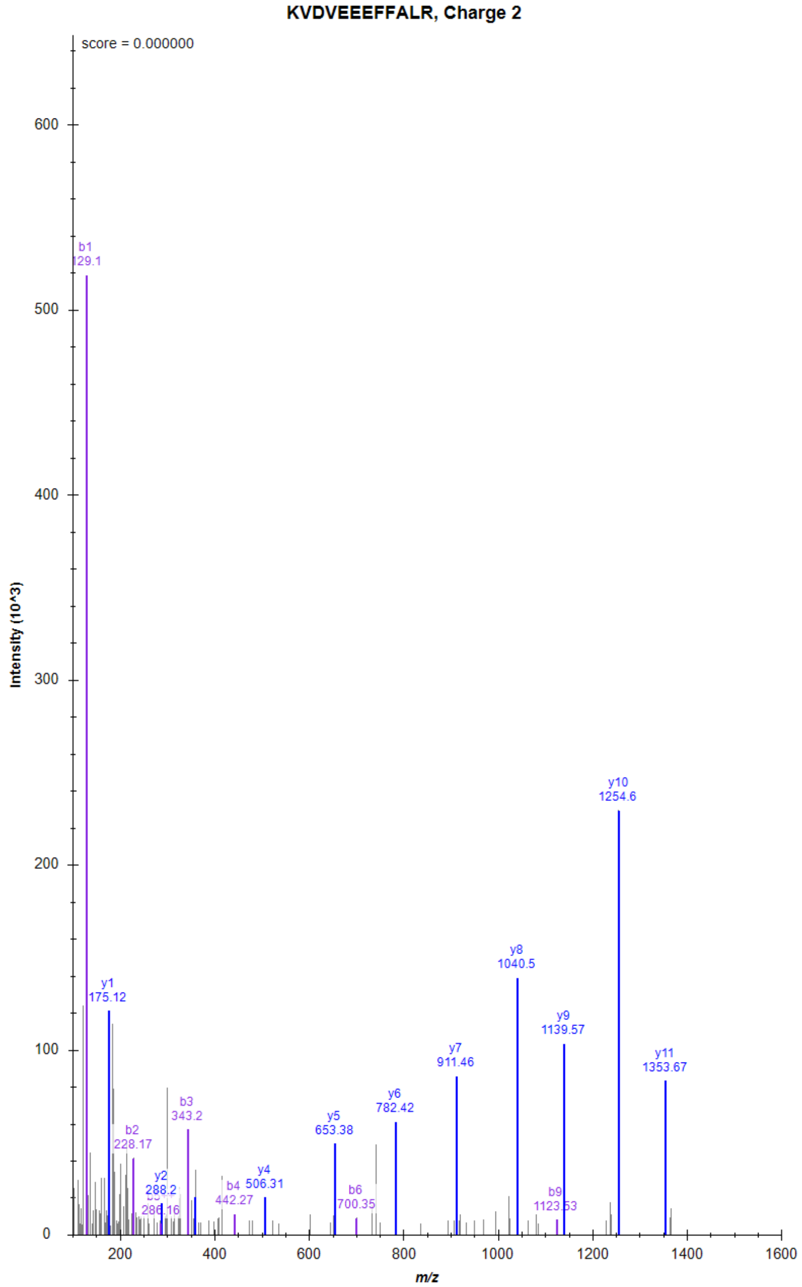

Housekeeping protein - Actin

Actin, cytoplasmic 1 (ACTB)

DLYANTVLSGGTTMYPGIADR, Charge 2, m/z 1108.03882 (*20 min LC run*)

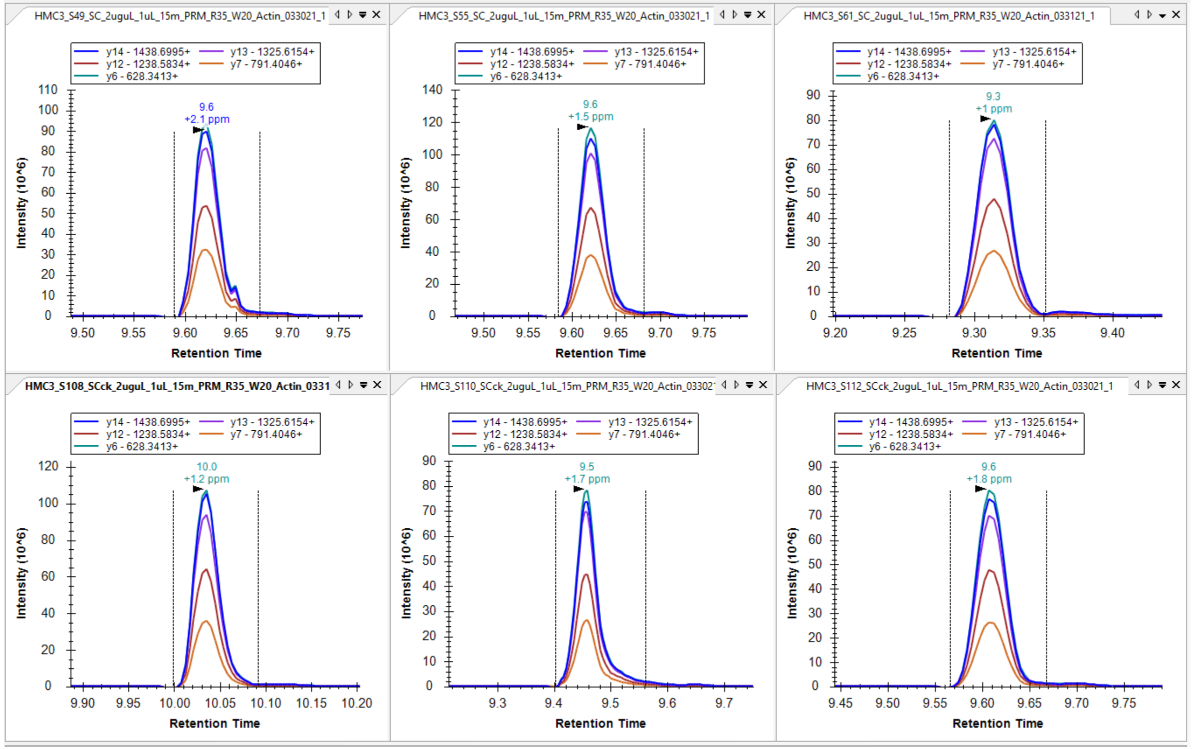

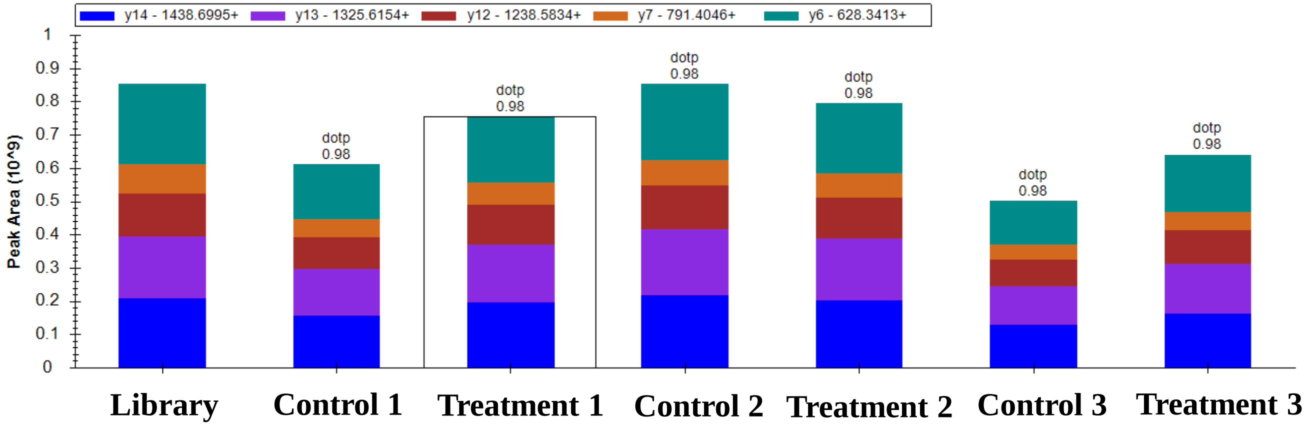

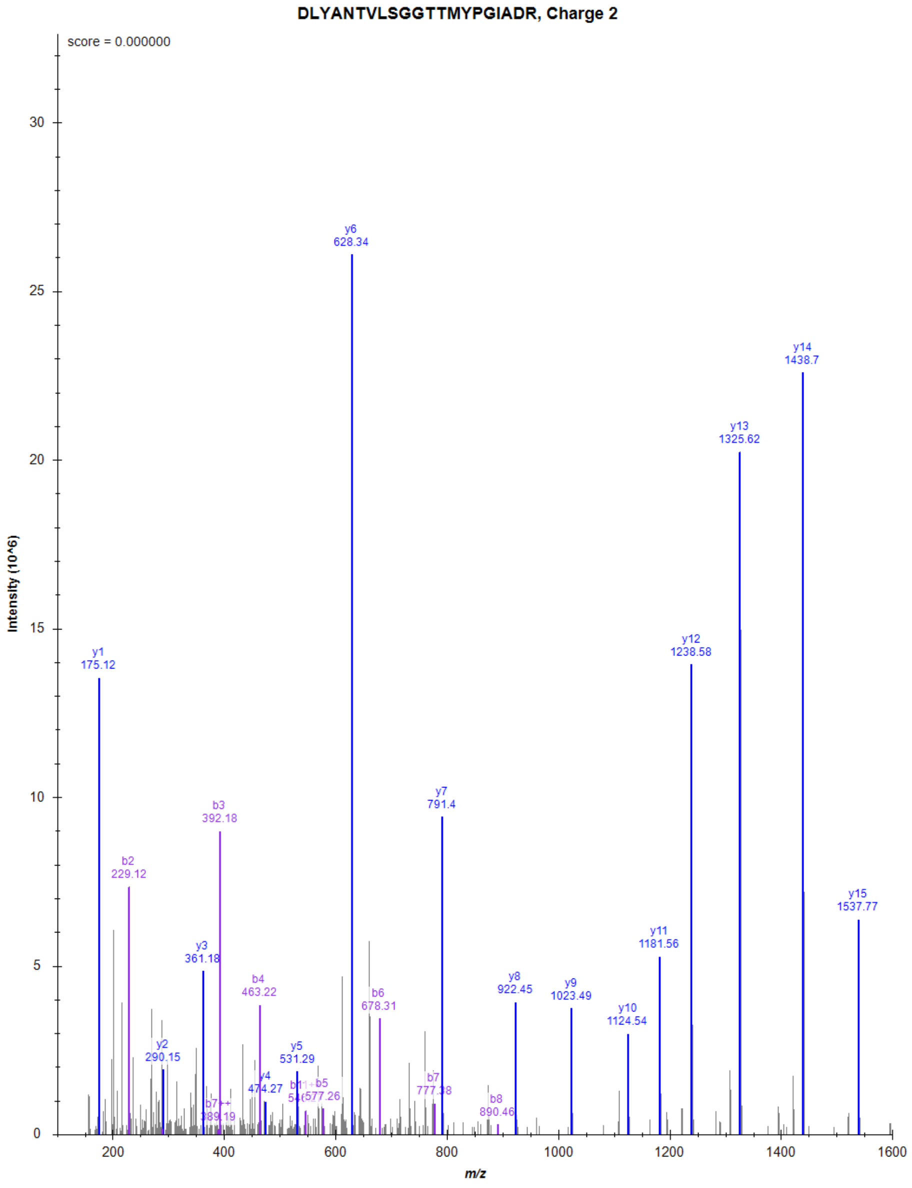

Actin, cytoplasmic 1 (ACTB)

YPIEHGIITNWDDMEK, Charge 2, m/z 980.9599 (*20 min full run search*)

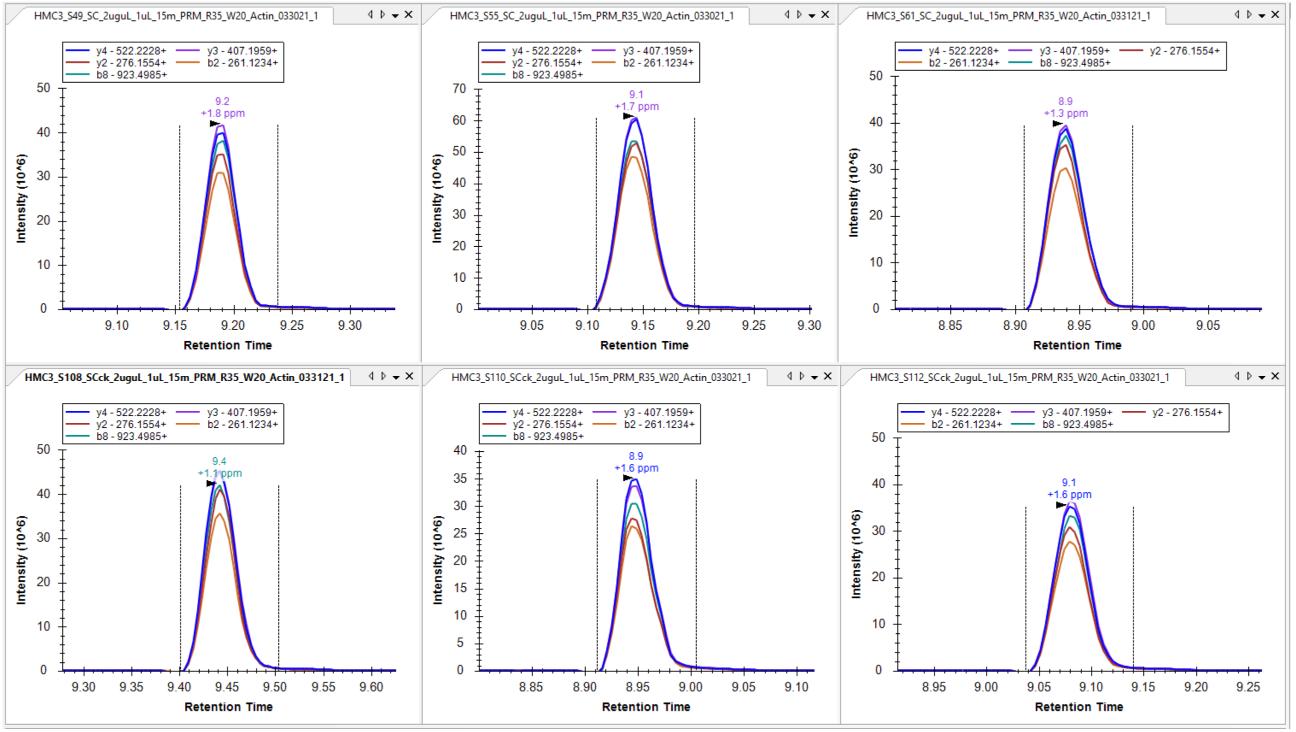

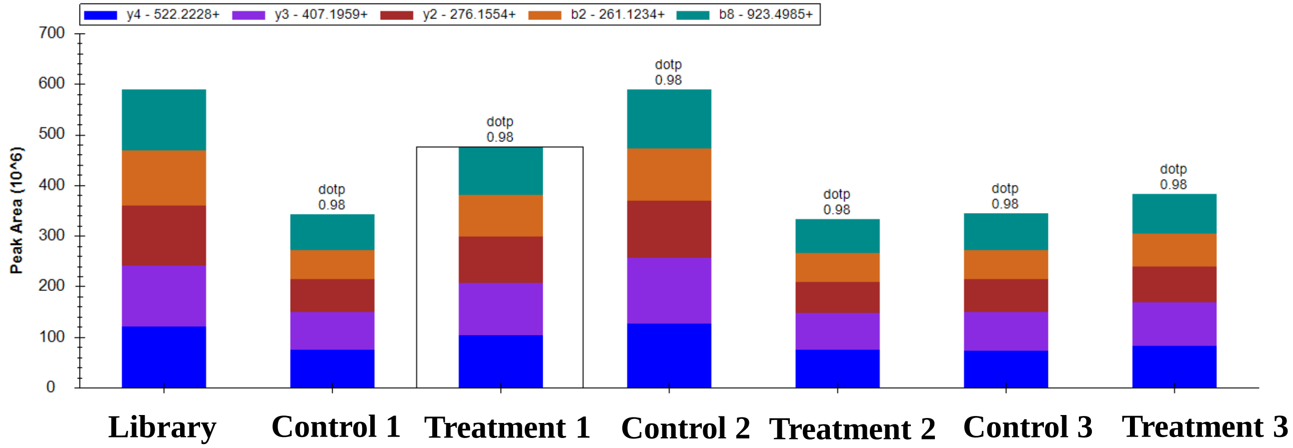

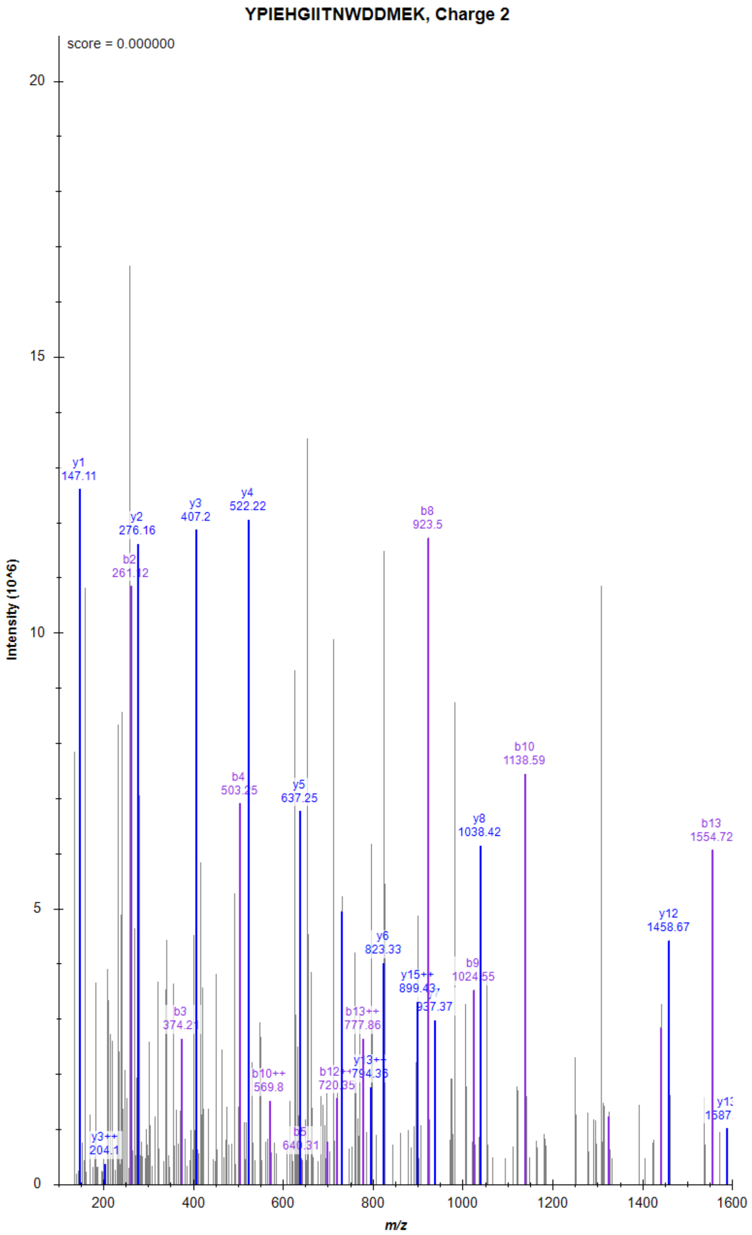

Supplement: Supplementary file 1 [file DataSheet_1.zip › Supplemental_file_1.pdf]
