# Supplementary material for: Systems-Level Proteomics Evaluation of Microglia Response to Tumor-Supportive Anti-Inflammatory Cytokines
Source: Front Immunol. 2021 Sep 9;12:646043. doi: 10.3389/fimmu.2021.646043 (PMC8458581; doi:10.3389/fimmu.2021.646043)
Supplement: Supplementary file 1 [file DataSheet_1.zip › Supplemental_file_2.pptx]

## Slide 1
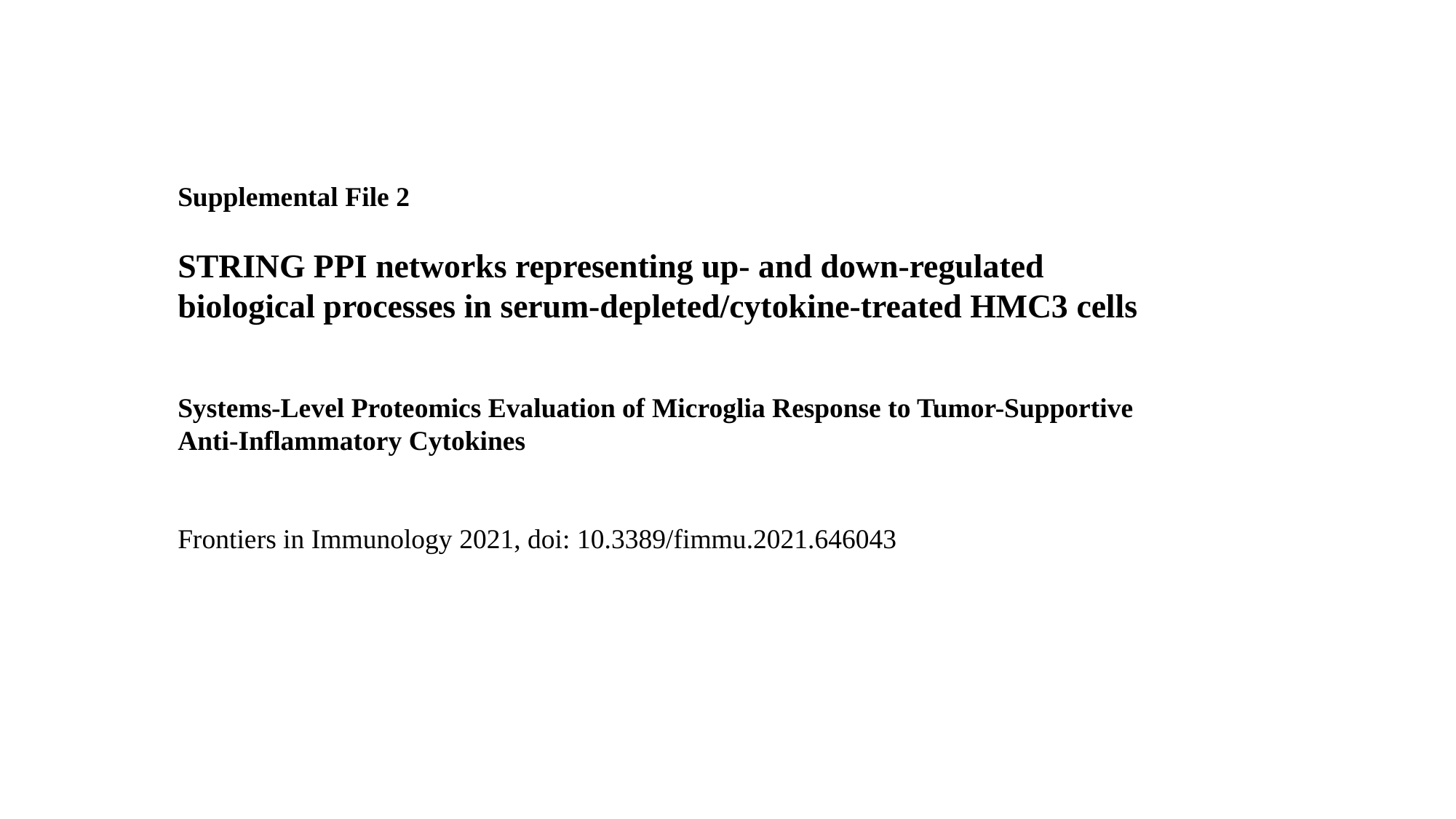

Supplemental File 2
STRING PPI networks representing up- and down-regulated biological processes in serum-depleted/cytokine-treated HMC3 cells
Systems-Level Proteomics Evaluation of Microglia Response to Tumor-Supportive
Anti-Inflammatory Cytokines
Frontiers in Immunology 2021, doi: 10.3389/fimmu.2021.646043

## Slide 2
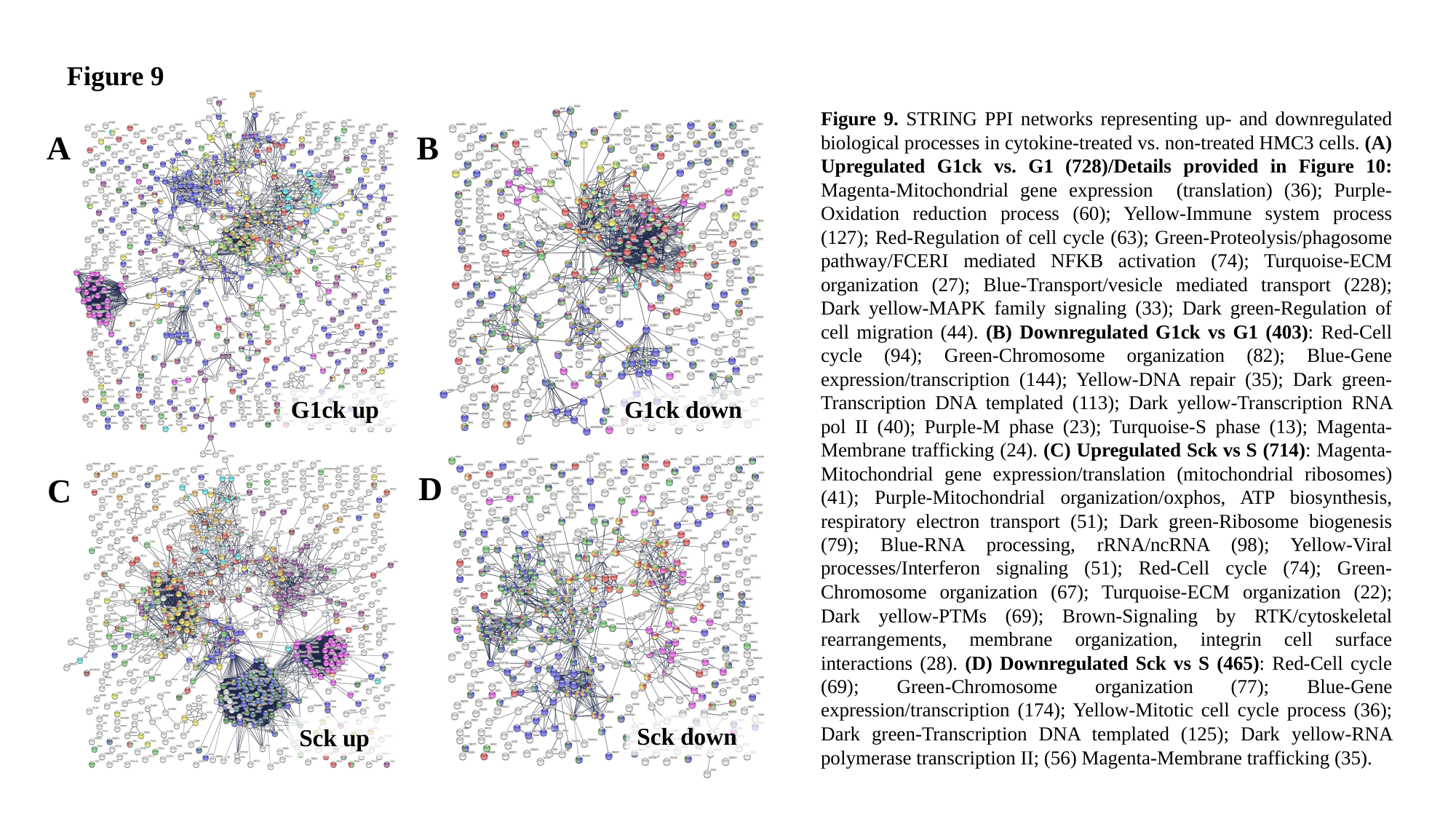

Figure 9
Figure 9. STRING PPI networks representing up- and downregulated biological processes in cytokine-treated vs. non-treated HMC3 cells. (A) Upregulated G1ck vs. G1 (728)/Details provided in Figure 10: Magenta-Mitochondrial gene expression (translation) (36); Purple-Oxidation reduction process (60); Yellow-Immune system process (127); Red-Regulation of cell cycle (63); Green-Proteolysis/phagosome pathway/FCERI mediated NFKB activation (74); Turquoise-ECM organization (27); Blue-Transport/vesicle mediated transport (228); Dark yellow-MAPK family signaling (33); Dark green-Regulation of cell migration (44). (B) Downregulated G1ck vs G1 (403): Red-Cell cycle (94); Green-Chromosome organization (82); Blue-Gene expression/transcription (144); Yellow-DNA repair (35); Dark green-Transcription DNA templated (113); Dark yellow-Transcription RNA pol II (40); Purple-M phase (23); Turquoise-S phase (13); Magenta-Membrane trafficking (24). (C) Upregulated Sck vs S (714): Magenta-Mitochondrial gene expression/translation (mitochondrial ribosomes) (41); Purple-Mitochondrial organization/oxphos, ATP biosynthesis, respiratory electron transport (51); Dark green-Ribosome biogenesis (79); Blue-RNA processing, rRNA/ncRNA (98); Yellow-Viral processes/Interferon signaling (51); Red-Cell cycle (74); Green-Chromosome organization (67); Turquoise-ECM organization (22); Dark yellow-PTMs (69); Brown-Signaling by RTK/cytoskeletal rearrangements, membrane organization, integrin cell surface interactions (28). (D) Downregulated Sck vs S (465): Red-Cell cycle (69); Green-Chromosome organization (77); Blue-Gene expression/transcription (174); Yellow-Mitotic cell cycle process (36); Dark green-Transcription DNA templated (125); Dark yellow-RNA polymerase transcription II; (56) Magenta-Membrane trafficking (35).
A
B
G1ck up
G1ck down
D
C
Sck down
Sck up

## Slide 3
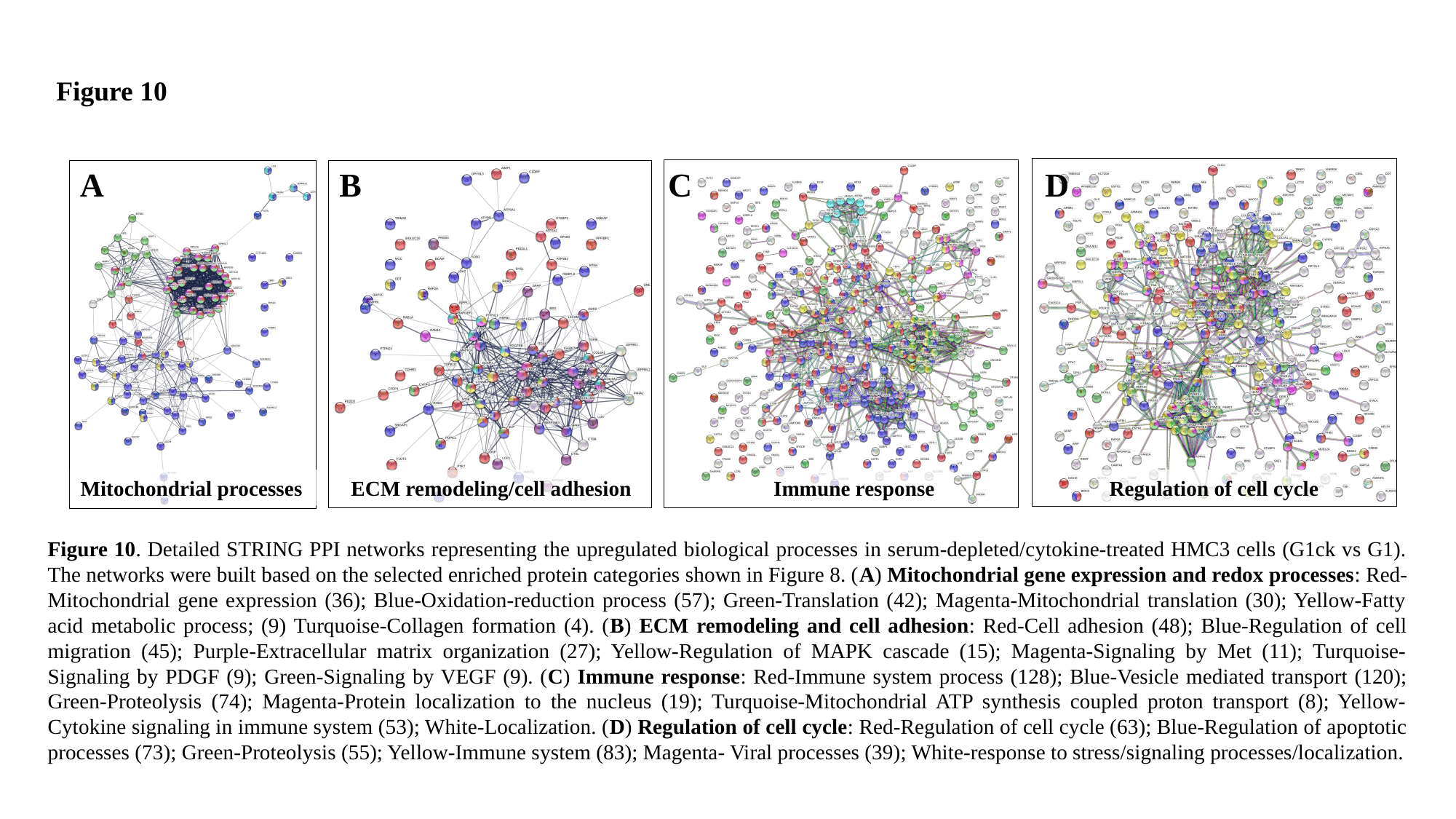

Figure 10
D
A
B
C
Mitochondrial processes
ECM remodeling/cell adhesion
Immune response
Regulation of cell cycle
Figure 10. Detailed STRING PPI networks representing the upregulated biological processes in serum-depleted/cytokine-treated HMC3 cells (G1ck vs G1). The networks were built based on the selected enriched protein categories shown in Figure 8. (A) Mitochondrial gene expression and redox processes: Red-Mitochondrial gene expression (36); Blue-Oxidation-reduction process (57); Green-Translation (42); Magenta-Mitochondrial translation (30); Yellow-Fatty acid metabolic process; (9) Turquoise-Collagen formation (4). (B) ECM remodeling and cell adhesion: Red-Cell adhesion (48); Blue-Regulation of cell migration (45); Purple-Extracellular matrix organization (27); Yellow-Regulation of MAPK cascade (15); Magenta-Signaling by Met (11); Turquoise-Signaling by PDGF (9); Green-Signaling by VEGF (9). (C) Immune response: Red-Immune system process (128); Blue-Vesicle mediated transport (120); Green-Proteolysis (74); Magenta-Protein localization to the nucleus (19); Turquoise-Mitochondrial ATP synthesis coupled proton transport (8); Yellow-Cytokine signaling in immune system (53); White-Localization. (D) Regulation of cell cycle: Red-Regulation of cell cycle (63); Blue-Regulation of apoptotic processes (73); Green-Proteolysis (55); Yellow-Immune system (83); Magenta- Viral processes (39); White-response to stress/signaling processes/localization.

## Slide 4
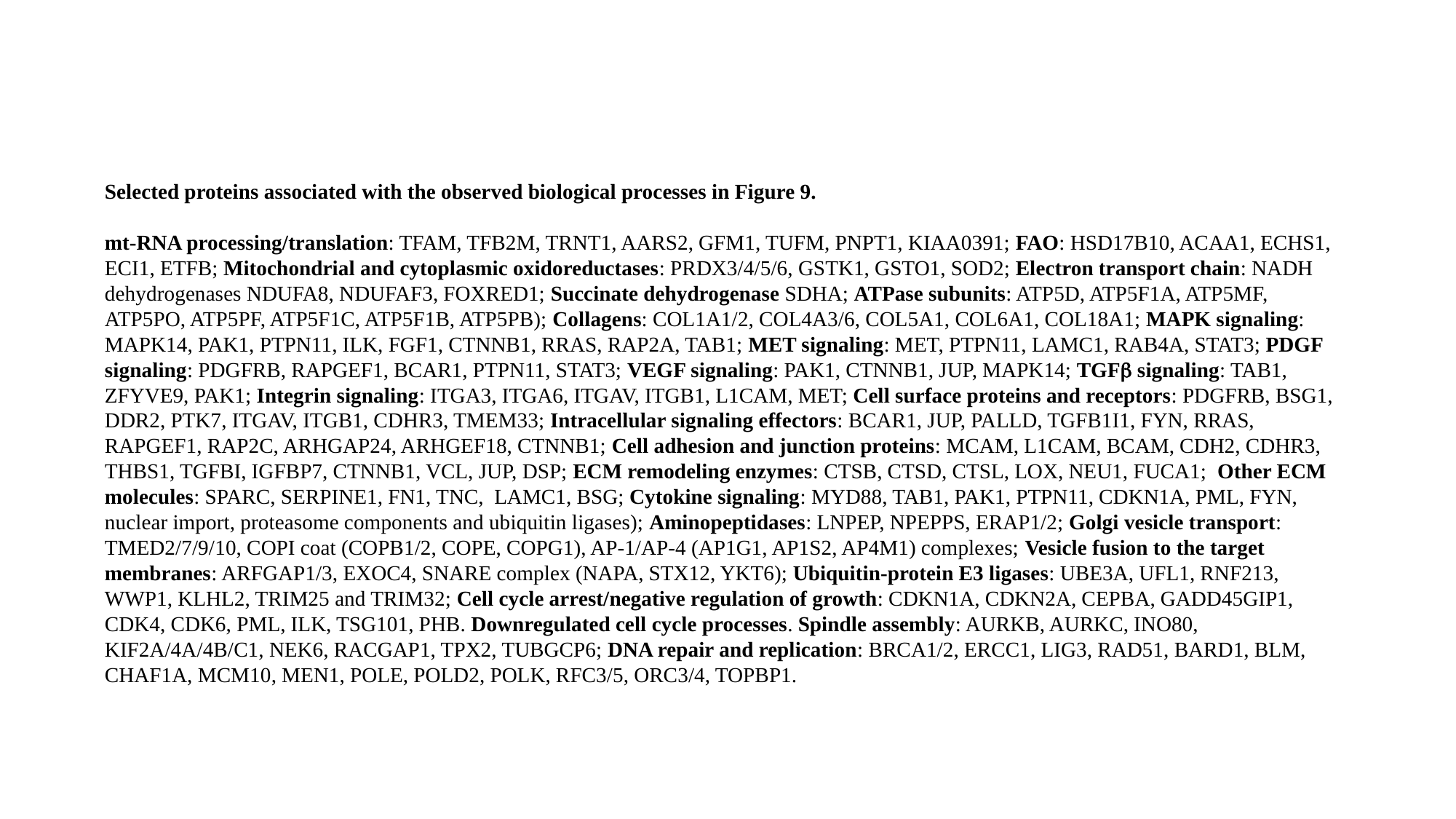

Selected proteins associated with the observed biological processes in Figure 9.
mt-RNA processing/translation: TFAM, TFB2M, TRNT1, AARS2, GFM1, TUFM, PNPT1, KIAA0391; FAO: HSD17B10, ACAA1, ECHS1, ECI1, ETFB; Mitochondrial and cytoplasmic oxidoreductases: PRDX3/4/5/6, GSTK1, GSTO1, SOD2; Electron transport chain: NADH dehydrogenases NDUFA8, NDUFAF3, FOXRED1; Succinate dehydrogenase SDHA; ATPase subunits: ATP5D, ATP5F1A, ATP5MF, ATP5PO, ATP5PF, ATP5F1C, ATP5F1B, ATP5PB); Collagens: COL1A1/2, COL4A3/6, COL5A1, COL6A1, COL18A1; MAPK signaling: MAPK14, PAK1, PTPN11, ILK, FGF1, CTNNB1, RRAS, RAP2A, TAB1; MET signaling: MET, PTPN11, LAMC1, RAB4A, STAT3; PDGF signaling: PDGFRB, RAPGEF1, BCAR1, PTPN11, STAT3; VEGF signaling: PAK1, CTNNB1, JUP, MAPK14; TGFβ signaling: TAB1, ZFYVE9, PAK1; Integrin signaling: ITGA3, ITGA6, ITGAV, ITGB1, L1CAM, MET; Cell surface proteins and receptors: PDGFRB, BSG1, DDR2, PTK7, ITGAV, ITGB1, CDHR3, TMEM33; Intracellular signaling effectors: BCAR1, JUP, PALLD, TGFB1I1, FYN, RRAS, RAPGEF1, RAP2C, ARHGAP24, ARHGEF18, CTNNB1; Cell adhesion and junction proteins: MCAM, L1CAM, BCAM, CDH2, CDHR3, THBS1, TGFBI, IGFBP7, CTNNB1, VCL, JUP, DSP; ECM remodeling enzymes: CTSB, CTSD, CTSL, LOX, NEU1, FUCA1; Other ECM molecules: SPARC, SERPINE1, FN1, TNC, LAMC1, BSG; Cytokine signaling: MYD88, TAB1, PAK1, PTPN11, CDKN1A, PML, FYN, nuclear import, proteasome components and ubiquitin ligases); Aminopeptidases: LNPEP, NPEPPS, ERAP1/2; Golgi vesicle transport: TMED2/7/9/10, COPI coat (COPB1/2, COPE, COPG1), AP-1/AP-4 (AP1G1, AP1S2, AP4M1) complexes; Vesicle fusion to the target membranes: ARFGAP1/3, EXOC4, SNARE complex (NAPA, STX12, YKT6); Ubiquitin-protein E3 ligases: UBE3A, UFL1, RNF213, WWP1, KLHL2, TRIM25 and TRIM32; Cell cycle arrest/negative regulation of growth: CDKN1A, CDKN2A, CEPBA, GADD45GIP1, CDK4, CDK6, PML, ILK, TSG101, PHB. Downregulated cell cycle processes. Spindle assembly: AURKB, AURKC, INO80, KIF2A/4A/4B/C1, NEK6, RACGAP1, TPX2, TUBGCP6; DNA repair and replication: BRCA1/2, ERCC1, LIG3, RAD51, BARD1, BLM, CHAF1A, MCM10, MEN1, POLE, POLD2, POLK, RFC3/5, ORC3/4, TOPBP1.
