# Supplementary material for: Systems-Level Proteomics Evaluation of Microglia Response to Tumor-Supportive Anti-Inflammatory Cytokines
Source: Front Immunol. 2021 Sep 9;12:646043. doi: 10.3389/fimmu.2021.646043 (PMC8458581; doi:10.3389/fimmu.2021.646043)
Supplement: Supplementary file 1 [file DataSheet_1.zip › Supplemental_file_3.pdf]

Supplemental File 3: Cytokine array analysis of cell culture supernatants from serum-free untreated and cytokine-treated HMC3 cells (G1 and G1ck)

Systems-Level Proteomics Evaluation of Microglia Response to Tumor-Supportive Anti-Inflammatory Cytokines

Frontiers in Immunology 2021, doi: 10.3389/fimmu.2021.646043

Components with increased abundance in the cell culture supernatant

- *Anti-inflammatory cytokines*: GDF15
- *Cytokines with dual inflammatory/anti-inflammatory function:* IL-6, LIF, IL-11, LCN2, PTX3, THBS1
- *Inflammatory cytokines and factors:* IL-5, IFN-γ, OPN, MPO
- *Other factors: chemokines (*CXCL12/SDF1α, OPN), cytokine activity (FLT3LG), growth factor activity (PDGFAA, ANGPT2, ENG, DKK1), endocytic factor (TFRC), protease (KLK3), vitamin D

Components with decreased abundance in the cell culture supernatant

- *Pro-inflammatory cytokines: IL-17A, IL-8*
- *Other factors: FGF-19 (cancer promoting growth factor), angiogenin (angiogenesis)*

Components with unchanged abundance in the cell culture supernatant

- *SERPINE1, BSG, BDNF, MIF, IGFBP2, VEGFA, UPAR*

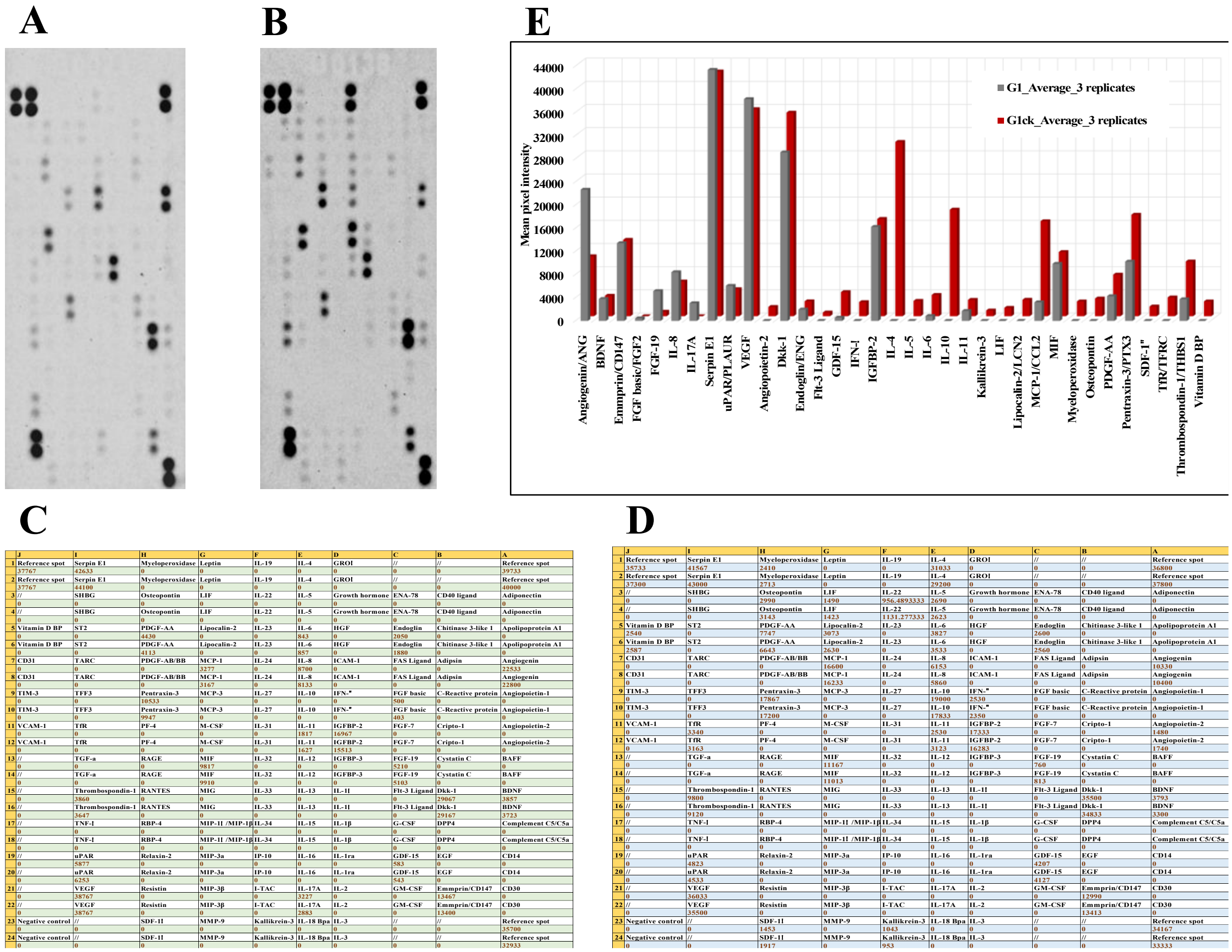

Supplemental figure 1: Cytokine array analysis of serum-free HMC3 cell culture supernatant (105 cytokines spotted on the membrane in duplicates). (**A**) Cytokines detected in the supernatant of the serum-free HMC3 cell cultures (G1). (**B**) Cytokines detected in the supernatant of the serum-free/cytokine-treated HMC3 cell cultures (G1ck). (**C**) Cytokine array matrix representing the average densitometry data for cytokines detected in three replicates of HMC3/G1 cells. (**D**) Cytokine array matrix representing the average densitometry data cytokines detected in three replicates of HMC3/G1ck cells. (**E**) Bar chart representing the mean pixel intensities of cytokines that were detected in the G1 (red) vs G1ck (grey) HMC3 cells. Differences in spot intensities for IL-4, IL-10 and MCP1 (CCL2) were not considered meaningful because these cytokines were added to the cocktail used to the treat the cells.

Selected proteins displaying increased expression in the supernatant of the G1ck cells

1. **GDF15** – Member of the TGFβ family and has been shown to induce anti-inflammatory effects by polarizing macrophages into the M2 phenotype. It also plays a role in enhancing tumor proliferation and metastasis (*Chang, J. Y., Hong, H. J., Kang, S. G., Kim, J. T., Zhang, B. Y., and Shong, M. (2020). The Role of Growth Differentiation Factor 15 in Energy Metabolism. Diabetes Metab J., 44(3), 363-371.*)

2. **Interleukin 6 (IL-6), Interleukin 11 (IL-11), Leukemia inhibitory factor (LIF)** – IL-6, IL-11 and LIF belong to the IL-6 family of inflammatory cytokines. IL-6 and LIF, together, have been shown to induce the differentiation of macrophages into tumor-associated macrophages and

promote the expression of M-CSF which recruits monocytes to the site of tumorigenesis (*Duluc, D., Delneste, Y., Tan, F., Moles, M. P., Grimaud, L., Lenoir, J et al. (2007). Tumor-associated leukemia inhibitory factor and IL-6 skew monocyte differentiation into tumor-associated macrophage-like cells. Blood, 110(13), 4319-4330.*). IL-11 too plays a role in pro-tumorigenic activities by contributing to cancer related inflammation (*Jones, S. A., and Jenkins, B. J. (2018). Recent insights into targeting the IL-6 cytokine family in inflammatory diseases and cancer. Nat. Rev. Immunol., 18(12), 773-789.*)

3. **Platelet derived growth factor (PDGFAA)** – PDGFR signaling is associated with increased cell migration, invasion, cell growth and metastasis. Ligand for the PDGF receptor that corroborated the upregulation of PDGFR signaling proteins in the G1ck cells.

4. **Thrombospondin (THBS1)** – This protein mediates cell-cell and cell-matrix interactions. (www.genecards.org). It was found to be upregulated in the G1ck cells by LC/MS analysis.

5. **Interferon gamma (IFNγ)** – Upregulation of interferon induced proteins (TRIM25, ISG15) observed with LC/MS analysis was corroborated by an increased intensity of the IFNγ spot on the cytokine array.

6. **Lipocalin 2 (LCN2)** – LCN2 promotes cell growth, regeneration and dampens inflammatory conditions. LCN2 was shown to be secreted by tumor-associated macrophages in response to stimulation by tumor cells and had a direct impact on lymphangiogenesis which enhances metastatic progression of cancer cells. (*Jung, M., Ören, B., Mora, J., Mertens, C., Dziumbla, S., Popp, R. et al. (2016). Lipocalin 2 from macrophages stimulated by tumor cell–derived sphingosine 1-phosphate promotes lymphangiogenesis and tumor metastasis. Sci. Signal. 9(434), ra64-ra64.*)

7. **Interleukin 5 (IL-5)** – Like the anti-inflammatory cytokines, IL-4, IL-13 and IL-10, interleukin 5 (IL-5) is a Th2 cytokine involved in countering the inflammatory responses in the body (*Berger, A. (2000). Th1 and Th2 responses: what are they? Bmj, 321(7258), 424.*). It's role in cancer progression is not entirely clear but there is evidence that IL-5 facilitates metastatic colonization in certain cancers (*Zaynagetdinov, R., Sherrill, T. P., Gleaves, L. A., McLoed, A. G., Saxon, J. A., Habermann, A. C. et al. (2015). Interleukin-5 facilitates lung metastasis by modulating the immune microenvironment. Cancer Res. 75(8), 1624-1634.*).

8. **Myeloperoxidase (MPO) –** The role of MPO in cancer progression has not been elucidated to a large extent. However, it has been implicated to play a role in the recruitment of immune cells to the tumorigenic site *(Kim, J., and Bae, J. S. (2016). Tumor-associated macrophages and neutrophils in tumor microenvironment. Mediators Inflamm. 2016).*

9. **Osteopontin (OPN)** – OPN is an extracellular matrix molecule which is primarily associated with inflammatory conditions. It plays a crucial role in cancer progression by promoting extracellular matrix remodeling by inducing the expression of MMPs. It also has a positive effect on cancer cell proliferation, invasion and survival. (*Castello, L. M., Raineri, D., Salmi, L., Clemente, N., Vaschetto, R., Quaglia, M., et al. (2017). Osteopontin at the crossroads of inflammation and tumor progression. Mediators Inflamm. 2017*). There is also evidence for a role of OPN in recruiting macrophages towards the site of tumorigenesis in glioblastoma. *(Wei, J., Marisetty, A., Schrand, B., Gabrusiewicz, K., Hashimoto, Y., Ott, M. et al. (2019). Osteopontin mediates glioblastoma-associated macrophage infiltration and is a potential therapeutic target. J. Clin. Investig. 129(1), 137-149.)*

10. **Transferrin receptor (TFRC)** – Involved in receptor-mediated endocytosis. TFRC upregulation was also demonstrated in microglia/macrophages isolated from adult and pediatric glioblastoma tumors. *(Engler, J. R., Robinson, A. E., Smirnov, I., Hodgson, J. G., Berger, M. S., Gupta, N. et al. (2012). Increased microglia/macrophage gene expression in a subset of adult and pediatric astrocytomas. PloS one, 7(8), e43339.).* The upregulation of TFRC in G1ck cells was also observed by LC/MS analysis.

11. **Pentraxin 3 (PTX3)** – Stimulation with IL-10 induces pentraxin-3 expression which promotes extracellular remodeling in M2c macrophages *(Mantovani, A., Sica, A., Sozzani, S., Allavena, P., Vecchi, A., and Locati, M. (2004). The chemokine system in diverse forms of macrophage activation and polarization. Trends Immunol. 25(12), 677-686.)*

12. **SDF1α/CXCL12** – Chemoattractant molecule with multifunctional roles in inflammatory response, cancer metastasis, and immune cell recruitment (www.genecards.org).

13. **Fms-related tyrosine kinase 3 ligand (FLT3LG)** – Structurally analogous to the colony stimulating factor (CSF1), this cytokine has a role to play in instigating immune response against pathogens (www.genecards.org). FLT3LG is expressed at high levels by tumor associated macrophages which provides chemoresistance to tumor cells (Guo, Y., Huang, J., Zhang, D., Zhang, L., Zhao, Y., Yu, L., et al. (2020). Tumor Associated Macrophages Express High-Levels of FLT3 Ligand, Which Induces Activation of FLT3 Signaling That Promotes Survival of Neoplastic Cells in B-Cell Acute Lymphoblastic Leukemia. Blood, Vol. 136). However, FLT3LG, which controls the development of dendritic cells (DCs) has also been shown to kill and shrink tumor cells by increasing the numbers of DCs at the tumorigenic site (Bhardwaj, N., Friedlander, P. A., Pavlick, A. C., Ernstoff, M. S., Gastman, B. R., Hanks, B. A. et al. (2020). Flt3 ligand augments immune responses to anti-DEC-205-NY-ESO-1 vaccine through expansion of dendritic cell subsets. Nat. Cancer, 1(12), 1204-1217.)

14. **Platelet derived growth factor (PDGFAA)** – PDGFR signaling is associated with increased cell migration, invasion, cell growth and metastasis. Ligand for the PDGF receptor that corroborated the upregulation of PDGFR signaling proteins in the G1ck cells.

15. **Angiopoietin-2 (ANGPT2)** – Angiopoietins are a family of growth factors that regulate angiogenesis and are associated with the M2 polarized macrophages. Targeting the ANG2 and TIE2 (receptor for ANG2) signaling has been shown to suppress tumorigenesis. (Mazzieri, R., Pucci, F., Moi, D., Zonari, E., Ranghetti, A., Berti, A. et al. (2011). Targeting the ANG2/TIE2 axis inhibits tumor growth and metastasis by impairing angiogenesis and disabling rebounds of proangiogenic myeloid cells. Cancer cell, 19(4), 512-526.)

16. **Endoglin (ENG)** – It is a glycoprotein found on the surface of cells which constitutes the TGFβ receptor complex. The expression of this protein increases in the presence of the TGFβ ligand. It stimulates angiogenesis and is associated with a number of malignancies. (Fonsatti, E., Vecchio, L. D., Altomonte, M., Sigalotti, L., Nicotra, M. R., Coral, S., et al. (2001). Endoglin: An accessory component of the TGF-β-binding receptor-complex with diagnostic, prognostic, and bioimmunotherapeutic potential in human malignancies. J. Cell. Physiol. 188(1), 1-7.)

17. **Dickkopf WNT Signaling Pathway Inhibitor 1 (DKK1)** – This protein antagonizes the β-catenin mediated Wnt signaling pathway. It has been discussed in detail in the manuscript that both positive and negative regulators of the Wnt pathway were present in the cytokine-treated microglia cells. Nevertheless, overexpression of this protein is implicated in increased cancer cell proliferation and invasion (www.genecards.org).

18. **Kallikrein-3 (KLK3)** – Kallikreins are serine proteases with important implications in cancer invasion and progression by promoting proteolysis of the extracellular matrix (ECM). KLK3 can also degrade IGFBP2 to release insulin-like growth factors which induces cancer cell proliferation. (Borgoño, C. A., and Diamandis, E. P. (2004). The emerging roles of human tissue kallikreins in cancer. Nat. Rev. Cancer, 4(11), 876-890.)

19. **Vitamin D binding protein (DBP/GC/GC-MAF)** – Binds, transports and stores vitamin D and plays a role in macrophage activation (www.genecards.org). Vitamin D promotes immunosuppressive effects and promotes M2 polarization (Wei, J., Gabrusiewicz, K., and Heimberger, A. (2013). The controversial role of microglia in malignant gliomas. Clin. Dev. Immunol.). DBP can also be converted to a macrophage activating factor (DBP-MAF) which is anti-tumorigenic as it suppresses angiogenesis. (Kisker, O., Onizuka, S., Becker, C. M., Fannon, M., Flynn, E., D'Amato, R. et al. (2003). Vitamin D binding protein-macrophage activating factor (DBP-maf) inhibits angiogenesis and tumor growth in mice. Neoplasia, 5(1), 32-40.)

Selected proteins displaying increased expression in the supernatant of the G1 cells

1. **Interleukin 17 (IL-17)** – Pro-inflammatory cytokine which mediates inflammatory functions via NFkB activation. (*Jin, W., and Dong, C. (2013). IL-17 cytokines in immunity and inflammation. Emerging microbes & infections, 2(1), 1-5.)*

2. **Fibroblast growth factor (FGF-19)** - Plays an important role in the regulation of cell survival, cell growth, cell differentiation and cell migration (*www.genecards.org*).

3. **Angiogenin (ANG) –** Angiogenin, belonging to the RNase superfamily; mediates angiogenesis. (*www.genecards.org*).

4. **Interleukin-8 (IL-8/CXCL8)** – IL8 is a pro-inflammatory cytokine which serves as a chemoattractant cytokine for many immune cells such as neutrophils. (*Bickel, M. (1993). The role of interleukin-8 in inflammation and mechanisms of regulation. J. Periodontol. 64(5 Suppl), 456-460.*)

Selected proteins displaying no change in expression in the supernatants of the G1 and G1ck cells

1. **SERPINE1/PAI-1** – This protein was observed to be upregulated in the cytokine-treated HMC3 cells by MS analysis. It is an inhibitor of the serine proteases that mediates cell adhesion and spreading. It promotes tumorigenesis by polarizing and recruiting M2 macrophages to the site of tumorigenesis. SERPINE1 was also shown to induce the expression of IL-6, which facilitated M2 polarization via STAT3 signaling axis (*Kubala, M. H., Punj, V., Placencio-Hickok, V. R., Fang, H., Fernandez, G. E., Sposto, R. et al. (2018). Plasminogen activator inhibitor-1 promotes the recruitment and polarization of macrophages in cancer. Cell Rep. 25(8), 2177-2191.*)

2. **Emmprin/CD147/Basigin (BSG)** – Plasma membrane protein which induces the production and release of matrix metalloproteases. Therefore, it plays significant roles in tumor growth, invasion and metastasis. (www.genecards.org). BSG, especially plays a crucial role in the brain as it controls ECM remodeling and neuroprotective functions during CNS pathologies like brain tumors. *(Kaushik, D. K., Hahn, J. N., and Yong, V. W. (2015). EMMPRIN, an upstream regulator of MMPs, in CNS biology. Matrix Biol. 44, 138-146.)*

3. **Brain-derived neurotrophic factor (BDNF) –** Microglia are an important source of BDNF in the CNS, a neurotrophin involved in the development, differentiation and survival of neurons under homeostatic as well as neuropathological conditions. BDNF expression is mediated by purinergic receptors (which have been discussed in the manuscript) whose expression is induced in microglial cells in the presence of CCL2. (*Ferrini, F., and De Koninck, Y. (2013). Microglia control neuronal network excitability via BDNF signalling. Neural Plast.*)

4. **Macrophage migration inhibitory factor (MIF)** – Pro-inflammatory cytokine involved in immunoregulation and response to pathogens (*www.genecards.org*). MIF expression is regulated by hypoxia and is correlated with increased angiogenesis and tumor progression. (*Conroy, H., Mawhinney, L., and Donnelly, S. C. (2010). Inflammation and cancer: macrophage migration inhibitory factor (MIF)—the potential missing link. QJM. 103(11), 831-836.*)

5. **Insulin-like growth factor-binding protein 2 (IGFBP2)** – Secreted protein that modulates the activity of insulin-like growth factors by binding them. It also has domains for binding integrins and heparin proteins. IGFBP2 is abundantly expressed in the central nervous system and mediates a number of developmental and neuroprotective functions in the embryonic and post-natal CNS (*Khan, S. (2019). IGFBP-2 Signaling in the brain: from brain development to higher order brain functions. Front. Endocrinol., 10, 822*). IGFBP2 has also been identified to play a crucial role in many cancer metastases events. (*Li, T., Forbes, M. E., Fuller, G. N., Li, J., Yang, X., and Zhang, W. (2020). IGFBP2: integrative hub of developmental and oncogenic signaling network. Oncogene, 39(11), 2243-2257.*).

6. **Vascular endothelial growth factor (VEGFA) –** Growth factor that regulates angiogenesis and blood vessel development. Tumor associated microglia/macrophages elicit VEGFA expression to induce vascularization in the brain tumor microenvironment (*Roesch, S., Rapp, C., Dettling, S., and Herold-Mende, C. (2018). When immune cells turn bad—tumor-associated microglia/macrophages in glioma. Int. J. Mol. Sci. 19(2), 436*).
